# BRCA1 orchestrates the response to BI-2536 and its combination with alisertib in MYC-driven small cell lung cancer

**DOI:** 10.1038/s41419-024-06950-w

**Published:** 2024-07-31

**Authors:** Jiahui Zhang, Xiaoli Liu, Peng Hou, Yang Lv, Gongfeng Li, Guozhen Cao, Huogang Wang, Wenchu Lin

**Affiliations:** 1 High Magnetic Field Laboratory, Hefei Institutes of Physical Science, Chinese Academy of Sciences, Hefei, 230031 Anhui P.R. China; 2 University of Science and Technology of China, Hefei, 230026 Anhui P.R. China; 3 The Second Affiliated Hospital, School of Medicine, The Chinese University of Hong Kong, Shenzhen & Longgang District People’s Hospital of Shenzhen, Shenzhen, 518172 P.R. China

**Keywords:** Cancer therapy, Cell death, Lung cancer

## Abstract

PLK1 is currently at the forefront of mitotic research and has emerged as a potential target for small cell lung cancer (SCLC) therapy. However, the factors influencing the efficacy of PLK1 inhibitors remain unclear. Herein, *BRCA1* was identified as a key factor affecting the response of SCLC cells to BI-2536. Targeting AURKA with alisertib, at a non-toxic concentration, reduced the BI-2536-induced accumulation of BRCA1 and RAD51, leading to DNA repair defects and mitotic cell death in SCLC cells. In vivo experiments confirmed that combining BI-2536 with alisertib impaired DNA repair capacity and significantly delayed tumor growth. Additionally, GSEA analysis and loss- and gain-of-function assays demonstrated that MYC/MYCN signaling is crucial for determining the sensitivity of SCLC cells to BI-2536 and its combination with alisertib. The study further revealed a positive correlation between *RAD51* expression and *PLK1*/*AURKA* expression, and a negative correlation with the IC_50_ values of BI-2536. Manipulating *RAD51* expression significantly influenced the efficacy of BI-2536 and restored the MYC/MYCN-induced enhancement of BI-2536 sensitivity in SCLC cells. Our findings indicate that the BRCA1 and MYC/MYCN-RAD51 axes govern the response of small cell lung cancer to BI-2536 and its combination with alisertib. This study propose the combined use of BI-2536 and alisertib as a novel therapeutic strategy for the treatment of SCLC patients with MYC/MYCN activation.

## Background

Small cell lung cancer (SCLC) is recognized as the most malignant form of lung cancer [1, 2]. Combination chemotherapy and radiation therapy have been established as the standard management for patients with SCLC [3]. Despite a frequent dramatic initial response to cytotoxic chemotherapy, platinum-based treatment has proven ineffective in the late stages of SCLC due to acquired resistance. Consequently, the prognosis of SCLC patients remains bleak, with a low 5-year survival rate lingering below 7% [4–6]. Recently, immunotherapy combined with chemotherapy has been approved for the first-line treatment of SCLC [3, 7]. However, its therapeutic efficacy and scope of clinical application remain constrained. Thus, the development of novel therapeutic strategies to enhance patient survival rates remains a critical and urgent challenge in the treatment of SCLC.

Cell cycle progression is a fundamental biological process in mammalian cells, where each phase must be tightly regulated by kinases and other factors to ensure accurate cell replication [8, 9]. Loss of normal cell-cycle control is a common feature of cancer cells. Chemotherapeutic agents targeting cell cycle progression are extensively used in clinical practice [10, 11]. Nonetheless, many of these agents act on both malignant and normal cells, leading to severe side effects. Strategies targeting tumor-specific cell cycle characteristics might achieve a robust response against cancer cells while minimizing cytotoxic effects on normal cells.

Cell cycle checkpoints are critical for maintaining genome integrity. However, these checkpoints are frequently deregulated in cancer cells, creating cancer vulnerabilities that could be exploited by small molecules. Polo-like kinases (PLKs), master regulators of the progression of mitosis and the G2/M checkpoint, encompass a family of five evolutionary conserved serine/threonine protein kinases that orchestrate mitotic progression [12]. Among the PLK family (PLK1-5), PLK1 is the most extensively studied member [13]. *PLK1* is often elevated in a wide variety of human cancers compared to normal tissues [14], giving these cancer cells a growth and invasion advantage [15–17]. Additionally, *PLK1* overexpression is commonly linked to a poor cancer prognosis [17]. Integrated bioinformatics analyses and functional investigation have identified PLK1 as a promising therapeutic target in various cancer types, including SCLC. Several studies have explored PLK1 inhibitors (PLK1i) [13, 18], including BI-2536 & BI-6727, for treating advanced metastatic tumors, including prostate cancer, lung cancer, neuroblastoma, and non-Hodgkin’s lymphoma [2, 13, 19]. The inhibition of PLK-1 using small molecules at nanomolar concentrations has shown encouraging preclinical outcomes in the aforementioned solid tumors and hematopoietic malignancies. However, clinical trials of PLK1 inhibitors as monotherapy have shown minimal or no clinical activity in SCLC [20].

The determinants influencing the response of PLK1 inhibitors (PLK1i) are not well understood. Several possibilities have been proposed to explain the limited antitumor activity of PLK1 inhibitors in clinical studies, including the short half-life and low accumulation of PLK1 inhibitors in tumor tissues. Additionally, the complexity of regulatory complexes that precisely control mitotic progression may pose another challenge in the effective use of PLK1 inhibitors. Aurora Kinase and PLK1, both pivotal in mitosis research, play essential roles in mitotic progression and DNA damage checkpoints [21–24]. Several studies have demonstrated that the Aurora-PLK1 cascade acts synergistically with cyclin B-CDK1, the fundamental signal module in mitosis [17, 24, 25]. Moreover, Aurora A kinase might play redundant roles with PLK1 in G2/M checkpoint and mitotic progression and exert PLK1-independent functions during the cell cycle [26–28]. Understanding the underlying mechanistic basis causing the ineffectiveness of PLK1i might facilitate specific therapeutic strategies for re-sensitizing or enhancing cancer cells to PLK1i and allow the design of rational combination therapies in treating advanced solid tumors, including SCLC.

Given that Aurora A and PLK1 are deregulated in a broad spectrum of human tumors and targeting PLK1 or Aurora A alone has yielded favorable outcomes, co-targeting AURKA and PLK1 might intensify defects in G2/M transition and mitotic progression [3, 17, 24, 29–32]. This intensified action could lead to more pronounced defects and subsequent cell death. Indeed, nasopharyngeal carcinoma and diffuse midline glioma cells have shown increased sensitivity to co-inhibition of PLK1 and AURKA compared to BI-2536 or AURKA inhibitors as a single agent [33–35]. However, co-targeting PLK1 and Aurora A kinases have not yet been explored in SCLC.

BRCA1 is pivotal in the repair of DNA double strand break, primarily through its regulation of homologous recombination. Numerous studies have established that BRCA1 is essential for the activation of Chk1 and RAD51 [36, 37], which are crucial during the DNA damage response. Consequently, inhibiting ATR-Chk1 signaling and RAD51 activation seriously disrupts DNA repair mechanisms. Additionally, BRCA1 is associated with drug resistance, particularly to DNA damage agents such as PARP inhibitors and platinum-based therapies. Despite these findings, BRCA1’s role in BI-2536 resistance has not been explored, even though there is a functional link between BRCA1 and PLK1 during the mitotic cell cycle transition [38]. In this study, we observed that treatment with BI-2536 elicited time- and concentration-dependent increase in BRCA1 levels, potentially contributing to resistance in SCLC cells to BI-2536. Suppression of BRCA1 with siRNA or Bractoppin enhanced the sensitivity of SCLC cells to BI-2536. Additionally, targeting AURKA with alisertib markedly reduced BI-2536-induced BRCA1 and RAD51 expression, thereby impairing DNA repair pathways and inducing G2/M arrest. This dramatically leveraging SCLC cell sensitivity to BI-2536. Furthermore, combined treatment with BI-2536 and alisertib exhibited potent synergistic antitumor efficacy in vivo. Gene set enrichment analysis (GSEA) of differentially expressed genes uncovered significant enrichment of c-MYC/MYCN-related gene sets in BI-2536-sensitive SCLC cells. Subsequent functional studies confirmed that high c-*MYC*/*MYCN* expression was a positive predictor of BI-2536 sensitivity. Moreover, RAD51, a well-recognized MYC/MYCN target, was identified as a critical determinant in the cellular response to BI-2536 and the dual combination of BI-2536 and alisertib.

## Results

### BRCA1 negatively modulates the sensitivity of BI-2536 alone and its combination with alisertib

**Fig. 1:**
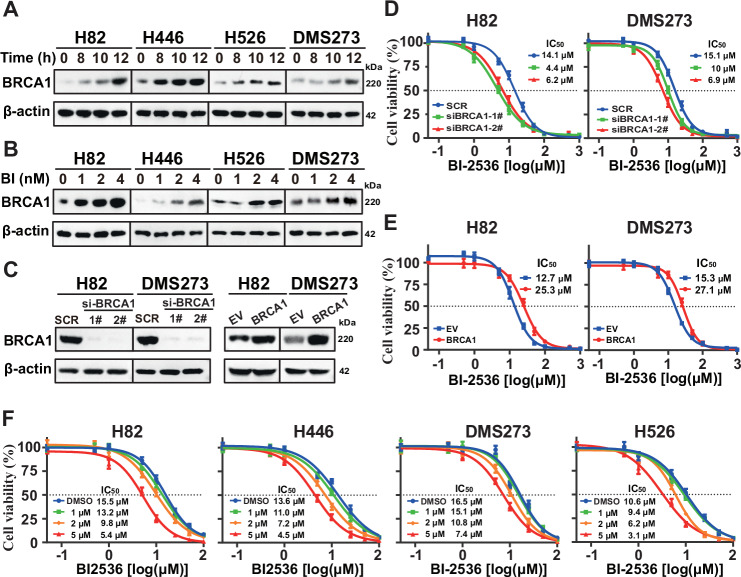
BRCA1 determines BI-2536 activity. **A** Representative time-course western blot analysis showing BRCA1 expression in SCLC cells treated with 1 nM BI-2536. **B** Representative dose-response western blot analysis for BRCA1 in SCLC cells treated with various concentrations of BI-2536 for 24 h. **C** Western blot analysis of BRCA1 expression in SCLC cells following BRCA1 silencing or overexpression. β-Actin was used as a loading control. Growth inhibition curves of BI-2536 monotherapy following BRCA1 silencing (**D**) or overexpression (**E**). **F** Growth inhibition curves of BI-2536 monotherapy in SCLC cells treated with various concentrations of Bractoppin for 24 h. Cells were treated with different concentrations of BI-2536 for 24 h. The cell viability was assessed using the CellTiter-Glo assay. The IC_50_ values were determined from the sigmoidal dose-response curves.

Prior research have unraveled a synthetic lethality interaction between PLK1 and BRCA1 [38]. Therefore, we investigated the potential role of BRCA1 as a negative regulator in the context of PLK1 inhibition. We assessed *BRCA1* expression following BI-2536 treatment in four SCLC cell lines. As depicted in Fig. 1A, B, both time-course and dose-response analysis demonstrated that BI-2536 resulted in marked induction of BRCA1 protein. Additionally, *BRCA1* silencing with two independent short interfering RNAs (siRNAs) in H82 and DMS273 cells led to enhanced sensitivity to BI-2536 (Fig. 1C, D), while ectopic expression of *BRCA1* had an opposite effect compared to *BRCA1* knockdown on BI-2536 activity (Fig. 1E). Moreover, inhibition of BRCA1 with Bractoppin, an inhibitor of phosphopeptide recognition by the BRCA1 tBRCT, resulted in a dose-dependent leftward shift in the cell viability curve, underscoring the significance of BRCA1 in modulating BI-2536 sensitivity (Fig. 1F). Collectively, these data highlight BRCA1 as a potentially pivotal determinant in the anti-cancer efficacy of BI-2536.

### Alisertib cooperates with BI-2536 to induce cell cycle and DNA double-strand breaks

**Fig. 2:**
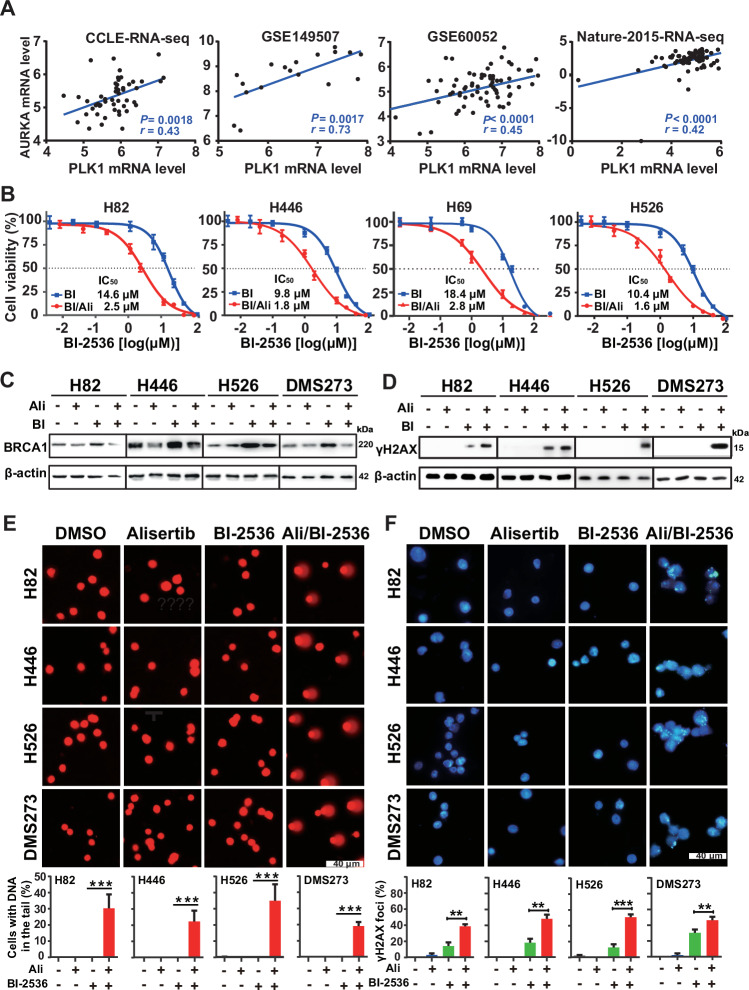
Combined PLK1 and AURKA inhibition affects DNA damage. **A** Pearson correlation analysis of PLK1 and AURKA transcription levels across different SCLC datasets, including CCLE RNA-seq, GSE149507, GSE60052, and Nature-2015 RNA-seq. **B** Growth inhibition curves for BI-2536 monotherapy and its combination with alisertib in four different SCLC cell lines. Cells were treated with varying concentrations of BI-2536 alone or combined with 20 nM alisertib for 24 h. The cell viability was determined by the CellTiter-Glo assay. **C**, **D** Western blot analysis of BRCA1 (**B**) and γH2AX (**C**) following treatment with BI-2536 and alisertib, alone or in combination, in SCLC cells. β-Actin was used as a loading control. Cells were treated with the drugs for 14 h (**B**) and 24 h (**C**), respectively. **E** Comet assay showing DNA damage in SCLC cells upon treated with BI-2536 and alisertib, alone or in combination for 24 h. DNA in the tail was used to assess DNA damage. DNA damage quantification shown as mean ± SD; ***p < 0.001 (unpaired Student’s *t* test). Scale bar, 40 μm. **F** Immunofluorescence staining of γH2AX in SCLC cells treated with indicated drugs for 24 h. Cells with more than 3 foci were considered as positive for DNA damage. **p < 0.01; ***p < 0.001 (unpaired Student’s *t* test). Scale bar, 40 μm.

We next explored the potential of a combination therapy involving BI-2536 for SCLC. Initially, we performed a genomic analysis of 249 SCLC clinical specimens with *PLK1* alterations using the cBioPortal database. The result indicated that *PLK1* was mutated at a very low rate in SCLC. Similarly, genomic profiling of 50 SCLC cell lines has also revealed a relatively low mutation frequency, although it was higher than in clinical samples (Fig. 2C, D). Subsequently, we carried out a comprehensive analysis of *PLK1* expression in multiple microarray and RNA-seq datasets. The analysis demonstrated that *PLK1* mRNA expression was significantly higher in SCLC cells compared to lung adenocarcinoma (LUAD) cells (*p* < 0.01) (Fig. S2A). Furthermore, *PLK1* mRNA expression in SCLC clinical specimens was remarkably higher than in adjacent noncancerous tissues (Fig. S2A). Kaplan-Meier analysis indicated that higher PLK1 mRNA expression levels correlated with better overall and progression-free survival in SCLC (Fig. S2B), suggesting that *PLK1* expression serves as an independent prognostic factor in SCLC. Given that PLK1 is a known target of AURKA, which was also overexpressed rather than mutated in SCLC (Fig. S3C), we investigated the potential of co-targeting PLK1 and AURKA. Analysis of various RNA-seq datasets from the CCLE database and others revealed a direct correlation between *AURKA* and *PLK1* mRNA expression in SCLC cells (Fig. 2A). Subsequently, pharmacological inhibition of PLK1 with BI-2536 and AURKA with alisertib resulted in a 5 to 9-fold decrease in BI-2536 IC_50_ values in SCLC cell lines (Fig. 2B).

To investigate the synergistic effect of the combination regimen, we examined their impact on cell cycle progression. The results showed that that the combined treatment induced a more pronounced G2/M arrest than BI-2536 alone (Fig. S4A). We assessed apoptosis by monitoring cleaved PARP protein levels via western blotting (Fig. S4B, C). Both BI-2536 and alisertib independently induced cleaved PARP protein accumulation, no enhanced apoptosis was observed 14 h after their combination (Fig. S4B). Additionally, there was no detectable increase in cleaved PARP 24 h after treatment, despite alisertib substantially enhanced BI-2536 cytotoxicity at this time point (Fig. S4C). These findings suggest that alisertib exhibits a cell-killing effect via mechanisms rather than apoptosis, even though co-targeting PLK1 and AURKA induced a G2/M arrest.

Given the enhanced anticancer effect of BI-2536 by alisertib-mediated AURKA inhibition, we speculated that BI-2536-induced BRCA1 might influence the effect of alisertib on cell viability in the presence of BI-2536. A panel of SCLC cell lines were treated with alisertib and BI-2536 as single-agents or in combination, and western blot analysis was performed to assess BRCA1 expression. Consistent with previous results, BI-2536 alone induced substantial BRCA1 accumulation, whereas alisertib showed minimal effect on *BRCA1* expression. Notably, co-inhibition of PLK1 and AURKA with BI-2536 and alisertib effectively downregulated BI-2536-evoked *BRCA1* increase (Fig. 2C).

It has been reported that AURKA and PLK1 are involved in the key processes of DNA damage repair besides cell cycle phase transition [39]. Therefore, we evaluated the extent of DNA damage using a comet assay to further delineate the underlying mechanism leading to enhanced cell killing. Compared to the control, the single-drug treatment group did not exhibit noticeable DNA damage up to 20 h. However, the combined treatment led to significantly increased DNA damage (Fig. 2D–F). Supporting the comet assay results, immunofluorescence staining confirmed a higher number of γH2AX foci in SCLC cells co-treated with alisertib and BI-2536 than in those treated by either drug alone (Fig. 2E). Moreover, western blot analysis showed that 20 nM alisertib treatment augmented BI-2536-induced γH2AX accumulation as early as 14 h post-combination treatment in all cell lines tested (Fig. S4D). In contrast, BI-2536 alone did not induce noticeable γH2AX accumulation in H526 and DMS273 cells and was only detectable after 24 h post-treatment in H82 and H446 cells (Fig. 2D). In summary, alisertib enhances BI-2536-mediated DNA double-strand breaks in SCLC cells.

### Alisertib attenuates homologous recombination competency in SCLC cells

**Fig. 3:**
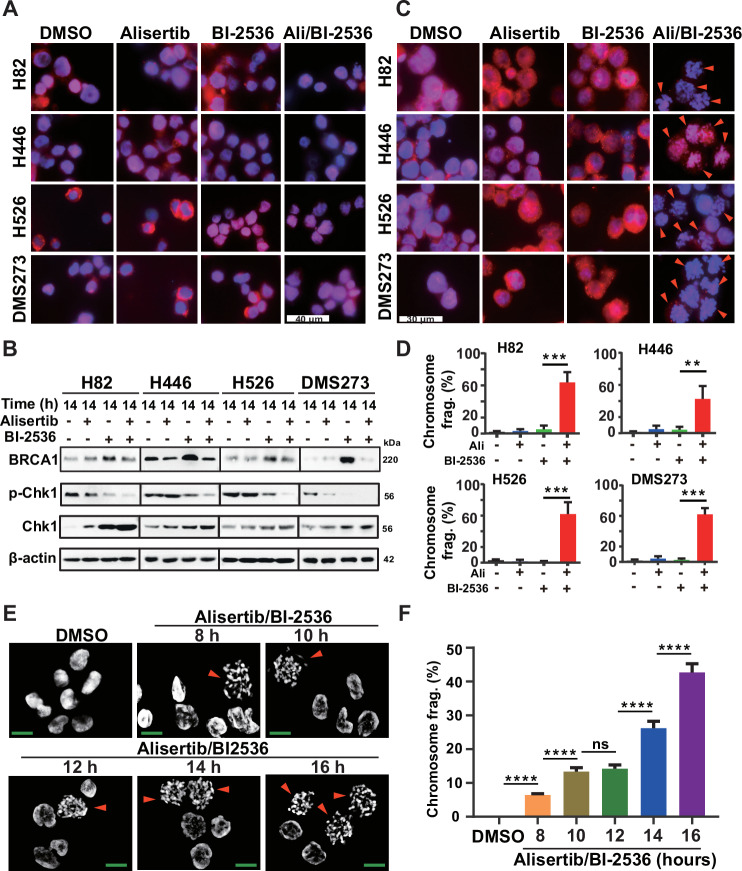
Impact of BI-2536 and alisertib on DNA damage repair pathway in SCLC cells. **A**, **C** RAD51 immunofluorescence in SCLC cells treated with indicated drugs for 14 h (**A**) and 24 h (**C**). Scale bars, 40 μm (**A**) and 30 μm (**C**). Quantification of RAD51 fluorescence intensities from three experiments shown as mean ± SD; **p < 0.01; ***p < 0.001 (unpaired Student’s *t* test). **B** Western blot of phosphorylated-ChK1, ChK1, and BRCA1 in SCLC cells treated with indicated drugs for 24 h. β-Actin was served as a loading control. **D** Proportion statistics of abnormal nuclear morphology. In order to evaluate the proportion of abnormal nuclear morphology and the difference between different drug treatment groups, at least 3 randomly selected fields of view in each sample were analyzed and the proportion of abnormal nuclear morphology to the total number of cells was calculated. **p < 0.01; ***p < 0.001 (unpaired Student’s *t* test). **E** Representative time-course analysis of abnormal nuclear morphology. H82 cells were treated with 1 nM BI-2536 and 20 nM alisertib for the indicated times, fixed with 4% paraformaldehyde, stained with DAPI, and observed under a confocal microscope. Scale bar, 10 μm. **F** The proportion of nuclear fragmentation was counted, and the proportion of the six visual fields in the drug treatment group at different time points was analyzed and plotted. The Quantification of nuclear fragmentation from three experiments shown as mean ± SD; ns no significance, ****p < 0.0001 (unpaired Student’s *t* test).

To further gain insight into the mechanism by which alisertib enhances the antitumor activity of BI-2536 in SCLC cells, we first assessed RAD51 foci formation upon treatment with BI-2536 and alisertib, both as monotherapies and in combination. Immunofluorescence staining revealed that the combined treatment markedly reduced the nuclear accumulation of RAD51 14 h post-treatment (Fig. 3A), whereas individual treatments had minimal impact. Notably, while BI-2536 and alisertib as monotherapies promoted RAD51 foci formation, the combination treatment markedly impaired RAD51 foci formation after 24 h (Fig. 3C). Subsequent western blot assays to evaluate DNA double-strand break (DSB) formation and repair showed that treatment with alisertib or BI-2536 alone caused a moderate decrease in phosphorylated -Chk1 (p-Chk1) protein levels. However, the combination resulted in a significant reduction of p-Chk1 protein across all four SCLC cell lines, despite substantial Chk1 protein accumulation regardless of the treatment (Figs. 3B and S5A). BI-2536 treatment alone significantly upregulated *BRCA1* expression, an effect reversed by the addition of alisertib (Figs. 3B and S5A). Both alisertib and BI-2536 reduced RAD51 expression, with alisertib further diminishing RAD51 level (Fig. S5A). Interestingly, extended treatment with both drugs resulted in a similar pattern for p-Chk1, BRCA1, and RAD51 expression (Fig. S5A). Furthermore, SCLC cells treated with the drug combination for 24 h displayed morphological features indicative of chromosome fragmentation, a phenomenon rarely observed with single drug treatment (Fig. 3C, D). Time-course analysis of chromosome fragmentation showed its onset at 8 h, increasing to more than 40% of cells showing chromatin fragmentation after 16 h of combined treatment (Fig. 3E, F). These findings suggest that mitotic cell death via chromatin fragmentation contributes to the synergistic cytotoxic effect of the combination treatment in SCLC.

To determine whether BRCA1, Chk1, and RAD51 are transcriptionally regulated by alisertib and BI-2536, RT-qPCR was performed to assess mRNA levels of these genes. The results indicated a significant increase in CHEK1 mRNA expression, aligning with western blot findings. Surprisingly, RT-qPCR analysis revealed robust increases in BRCA1 and RAD51 transcription regardless of treatment type (Fig. S5B), suggesting that PLK1 might counteract the function of BRCA1 and that regulation of BRCA1 and RAD51 might occur post-translationally.

Considering that proteasome-mediated protein degradation is a primary post-translational regulatory mechanism, we treated SCLC cells with bortezomib (BTZ), a 26S proteasome inhibitor, to assess protein degradation. Inhibition of proteolysis by BTZ effectively nullified the discrepancies between protein and gene expressions induced by the treatments (Fig. S5C). In summary, the downregulation of BRCA1 and Rad51 induced by the BI-2536/alisertib combination appears to be primarily regulated through proteasome-mediated protein degradation.

### Dual inhibition elicits DNA damage and synergistically retards tumor growth

**Fig. 4:**
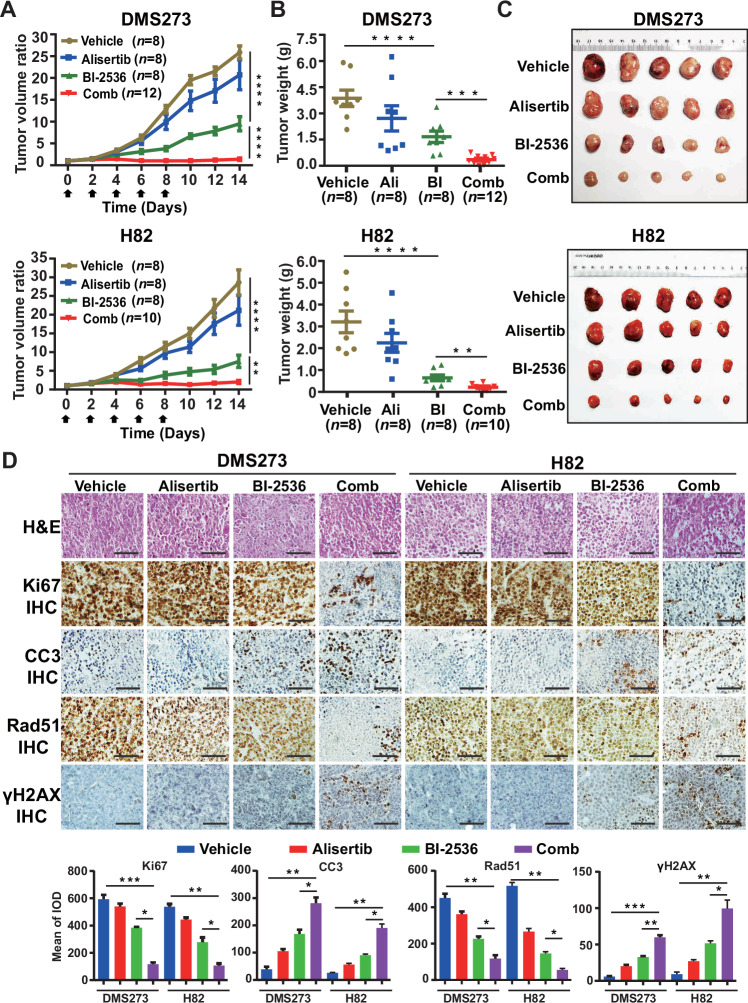
Therapeutic efficacy of BI-2536 and alisertib alone or in combination in vivo. **A** Tumor volume curves of DMS273 and H82 xenograft mice treated with BI-2536, alisertib, or their combination. Data are shown as Mean ± SD. “Comb” denotes the combination of BI-2536 and alisertib. **B** Tumor weights of DMS273 and H82 xenograft mice after the 14 days of drug treatment. “Ali” represents alisertib; “BI” represents BI-2536; “Comb” represents the combination of BI-2536 and alisertib. Statistical analysis as mean ± SEM; **p < 0.01; ***p < 0.001; ****p < 0.0001 (unpaired Student’s *t* test). **C** Images of representative tumors (DMS273, top panel; H82, bottom panel) from each treatment group. Tumor weight was measured after the tumors were harvested at the end of the experiment. **D** Representative immunohistochemistry images of Ki67, cleaved-caspase3, RAD51, and γH2AX in xenografts treated with BI-2536 and alisertib alone or in combination. Scale bar, 50 μm. The histogram takes mean of integrated optical density (IOD) as the ordinate, and each group counts 4 fields of vision, Values were presented as mean ± SD; *p < 0.05; **p < 0.01; ***p < 0.001 (unpaired Student’s *t* test).

We then evaluated the therapeutic efficacy of BI-2536 and alisertib as monotherapies or in combination in SCLC xenograft models. H82 and DMS273 cells were injected subcutaneously into the flanks of immunocompromised mice, and drug treatment was initiated when the tumors reached approximately 100 mm^3^. The treatment regimen was well tolerated, as evidenced by the absence of significant body weight loss (Fig. S6A, B). Alisertib alone had no significant effects on inhibiting the growth of H82 and DMS273 tumors compared with the control group and only showed marginal effects on reducing tumor weight. Conversely, BI-2536 monotherapy significantly inhibited tumor growth. However, the combination of BI-2536 and alisertib led to more substantial and enduring tumor growth inhibition compared to either drug alone. Tumor weights in the combination treatment group were notably lower than those in the monotherapy groups (Fig. 4A–C). Further analysis revealed a significant decrease in Ki67-positive tumor cells and an increase in cleaved Caspase3-positive cells in the combination treatment group compared to the control and monotherapy groups. Immunohistochemical analysis also showed that the drug combination more effectively reduced RAD51 expression and increased γH2AX accumulation than single-agent treatments (Fig. 4D). These findings demonstrate that alisertib enhances the inhibitory effect of BI-2536 on SCLC tumor growth. The observed enhanced DNA damage and cytotoxicity of PLK1i in vivo are likely associated with the alisertib-mediated impairment of DNA repair capacity.

### The MYC/MYCN signaling correlates with BI-2536 sensitivity

**Fig. 5:**
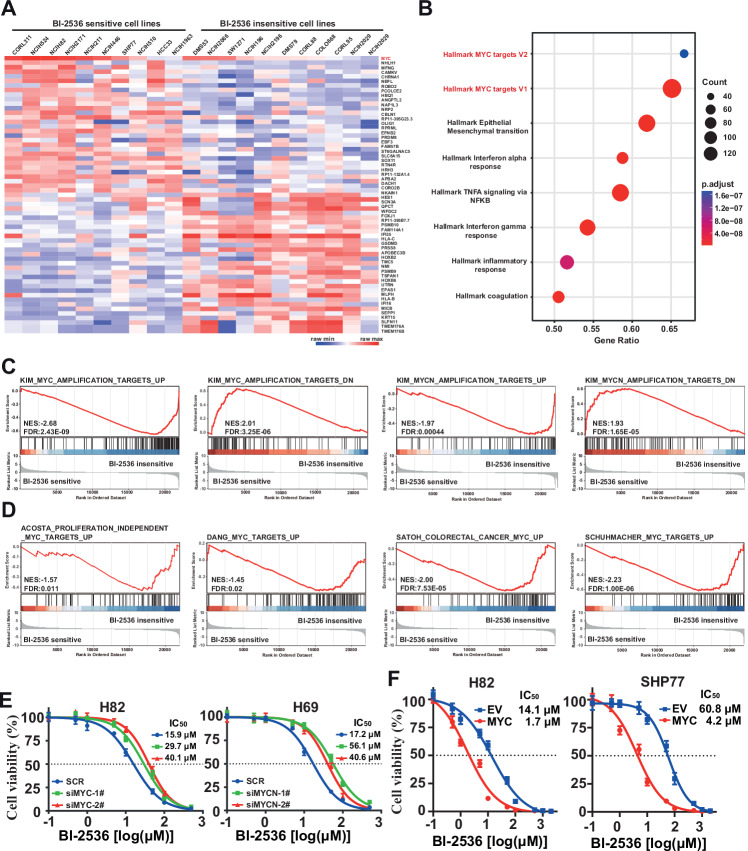
Differential gene expression between BI-2536 sensitive and insensitive cell lines enriched in MYC and MYCN targets. **A** Heatmap showing the top 30 upregulated and downregulated genes between BI-2536 sensitive and insensitive cell lines. The selection criteria for these genes include a fold change greater than 2 and a *p* value less than 0.05. **B** Bubble plot depicting the top 8 gene sets enriched in the Hallmark collection identified by GSEA in BI-2536 sensitive cell lines compared to insensitive ones. **C**, **D** GSEA highlighting gene sets that are downregulated or upregulated and correlated or anticorrelated with MYC or MYCN in BI-2536 insensitive cell lines. Gene sets are derived from C2 gene sets based on studies of SCLC (**C**) and other cancers (**D**). **E**, **F** Dose response curves representing cell viability of BI-2536 in four SCLC cell lines following siRNA-mediated MYC knockdown (**E**) or MYC overexpression (**F**).

We then sought to identify the features that might better predict the sensitivity of SCLC cells to BI-2536. The relative activity of BI-2536 across 56 SCLC cell lines, sourced from the Genomics of Drug Sensitivity in Cancer (GDSC) database, alongside RNA-seq data from the corresponding cell lines in the Cancer Cell Line Encyclopedia (CCLE) database, were gathered and analyzed. The IC_50_ values of these cell lines ranged from 0.0004 to 305 μM. Eleven cell lines yielding markedly higher IC_50_ values were categorized as insensitive, while ten cell lines demonstrating relative lower IC_50_ values were designated as sensitive. After defining the various cell subsets in the dataset, we performed genome-wide transcriptional profiling of the 21 cell lines, to identify potential determinants of BI-2536 activity relevant to SCLC. As illustrated in Fig. 5A, *c-MYC* emerged as a top-ranked gene significantly correlated with BI-2536 sensitivity.

Gene set enrichment analysis (GSEA) was then undertaken to explore the hallmarks associated with BI-2536 response. The enrichment of the Hallmark collection revealed that MYC targets V2 and MYC targets V1 were the top two significant gene sets, particularly for down-regulated genes in BI-2536-insensitive cells. Conversely, the gene expression profile in BI-2536-sensitive cells showed a negative correlation with epithelial-mesenchymal transition (EMT), NF-KB pathway, interferon-gamma response, inflammatory response, and other biological processes (Fig. 5B). To evaluate the effects of BI-2536 more specifically on gene sets regulated by either c-MYC or MYCN, A more targeted enrichment analysis was then conducted on the full set of chemical and genetic perturbations in the C2 collection. This analysis indicated a strong correlation between c-MYC and MYCN targeted gene sets derived in SCLC cells and BI-2536 sensitivity (Fig. 5C). Additionally, BI-2536 sensitive SCLC cells statistically exhibited enrichment in c-MYC and MYCN signatures from other cancer types and normal cells, including colorectal, leukemia, lymphoma, blood B cells, and fibroblasts (Figs. 5D and S7A). These findings indicate that MYC/MYCN signaling play a pivotal role in determining BI-2536 sensitivity.

### Gain and loss of *MYC*/*MYCN* modulates BI-2536 sensitivity in SCLC

In light of our data analysis, we hypothesized that *MYC/MYCN* expression levels could influence BI-2536 sensitivity in SCLC cells. To test a direct role for *MYC*/*MYCN* in BI-2536 sensitivity, we selected SCLC cell lines with high *MYC* expression (H82, H446, and DMS273), high *MYCN* expression (H69), and low *MYC* expression (SHP77) to assess whether *MYC*/*MYCN* depletion confers BI-2536 resistance. As a result, knockdown of MYC/MYCN using two distinct siRNAs led to a 2 to 4-fold increase in BI-2536 IC_50_ values in H82, H446, DMS273, and SHP77 cells (Figs. 5E and S7B). Conversely, ectopic *MYC* expression resulted in a leftward shift in the cell viability curve following BI-2536 treatment (Figs. 5F and S7C), supporting a direct correlation between *MYC/MYCN* expression and BI-2536 sensitivity in SCLC cells.

### Combining BI-2536 and alisertib is highly efficacious and depends on *MYC*/*MYCN* in SCLC

**Fig. 6:**
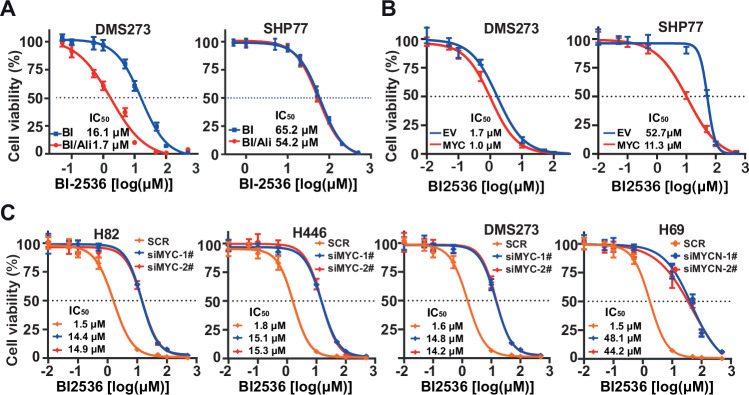
Impact of MYC on the sensitizing effect of targeted PLK1 and AURKA in SCLC. **A** Growth inhibition curves for BI-2536 monotherapy and in combination with alisertib. Growth inhibition curves for BI-2536 monotherapy and in combination with alisertib following MYC overexpression (**B**) or MYC/MYCN siRNA knockdown (**C**). Cells were treated with various concentrations of BI-2536, alone or in combination with 20 nM alisertib, for 24 h. Cell viability was determined by the CellTiter-Glo assay.

Previous studies have highlighted that PLK1 is a potential therapeutic target in tumors with high *MYC* expression [40, 41]. Additionally, the expression levels of *MYC* family genes impact the therapeutic effect of alisertib [42, 43]. To figure out whether *MYC*/*MYCN* expression contributes to the efficacy of the combined use of BI-2536 and alisertib, we assess the effect of changes of *MYC*/*MYCN* expression on the activity of combination using cell viability assays. The cell viability assays showed that ectopic *MYC* expression significantly enhanced the cytotoxic effects of the BI-2536 and alisertib combination, as evidenced by a leftward shift in the viability curve (Figs. 6B and S8B). Correspondingly, in the cell line with high expression of *MYC* (H82, H526, H446, and DMS273) or *MYCN* (H69), the combined treatment showed a significant sensitizing effect compared to treatment with BI-2536 alone, with a more than 4-fold change in the IC_50_ values. However, in the cell line with low MYC expression (SHP77), there was almost no synergistic sensitizing effect observed when the two drugs were combined compared to single-agent treatment (Figs. 6A and S8A). In contrast, concurrent loss of *MYC*/*MYCN* caused a rightward shift in the dose-response curve of the combination treatment (Fig. 6C). These findings collectively underscore the critical role of *MYC*/*MYCN* as a determinant of BI-2536 efficacy, both as a standalone agent and in combination with alisertib in SCLC.

### The MYC/MYCN-RAD51 axis increases the sensitivity of BI-2536 and its combination with alisertib

**Fig. 7:**
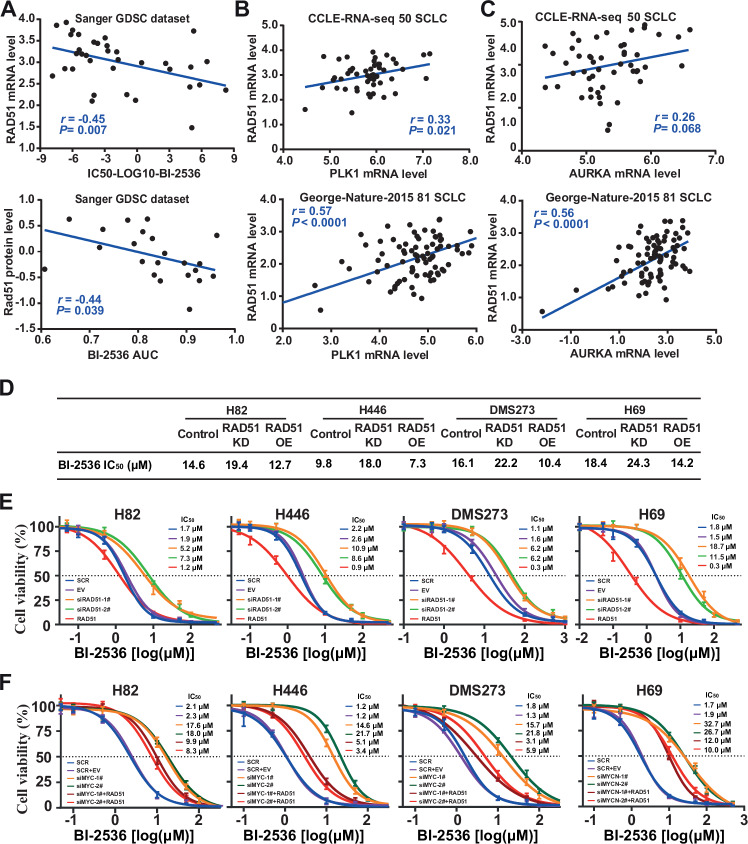
Influence of the MYC-RAD51 axis on BI-2536 and alisertib sensitization in SCLC treatment. **A** Pearson correlation analysis of RAD51 expression levels and BI-2536 drug sensitivity in SCLC cells. **B** Pearson correlation analysis of PLK1 and RAD51 transcription levels in SCLC cells. Analysis was performed using RNA-seq data from 50 SCLC samples in the CCLE dataset and 81 SCLC samples from a 2015 Nature publication. **C** Positive correlation between RAD51 and AURKA transcription levels in SCLC. **D** IC_50_ values of BI-2536 following RAD51 knockdown or overexpression. **E** Growth inhibition curves for BI-2536 monotherapy and combination with alisertib following RAD51 knockdown or overexpression. **F** Growth inhibition curves for BI-2536 combined with alisertib following MYC knockdown or MYC knockdown plus RAD51 overexpression. Cells were treated with various concentrations of BI-2536, alone or in combination with 20 nM alisertib, for 24 h. Cell viability was determined by the CellTiter-Glo assay.

Previous investigations have showed that RAD51 is a direct target of MYC/MYCN [44, 45]. To explore the possibility of RAD51 being responsible for the MYC/MYCN-mediated sensitivity of BI-2536 and its combination with alisertib, we analyzed the correlation of *RAD51* expression and IC_50_ values of BI-2536, the expression of *PLK1* and *AURKA*, using Pearson correlation. As depicted in Fig. 7A, RAD51 expression, at both mRNA and protein levels, was negatively correlated with BI-2536 activity. Additionally, *RAD51* expression was positively associated with the mRNA levels of *PLK1* and *AURKA* (Fig. 7B, C). These results prompted us to hypothesize that *RAD51* expression is critical for BI-2536 sensitivity and that the MYC/MYCN-RAD51 axis affects the activity of combining BI-2536 with alisertib.

To test this hypothesis, we evaluated the survival of SCLC cells treated with BI-2536 alone or in combination with alisertib under conditions of *RAD51* knockdown or overexpression. *RAD51* knockdown resulted in increased cell viability, whereas *RAD51* overexpression heightened drug sensitivity to BI-2536 compared to control cells (Fig. 7D, E). Furthermore, the cytotoxicity of the BI-2536 and alisertib combination was significantly enhanced by *RAD51* overexpression and diminished following RAD51 depletion across the four cell lines tested (Fig. 7E). Notably, loss of RAD51 negated the enhancing effect of MYC/MYCN on BI-2536 sensitivity, both alone and in combination with alisertib (Fig. 7D–F). These findings underscore the potential of targeting the MYC/MYCN-RAD51 axis to augment SCLC sensitivity to BI-2536, both as a monotherapy and in combination with alisertib.

## Discussion

Small cell lung cancer (SCLC), the most malignant form of lung cancer, currently lacks a clinically effective targeted treatment regimen [2]. Although numerous studies have highlighted PLK1 as a promising therapeutic target for SCLC [20, 46], a phase II trial of PLK1 inhibitor BI-2536 as a monotherapy demonstrated limited activity [2, 47]. In our study, several factors controlling BI-2536 activity have been examined using bioinformatics and molecular biology methods. We first illustrated that BRCA1 was evoked in response to BI-2536 treatment and that BRCA1 inhibition could markedly sensitize BI-2536 activity, establishing BRCA1 as a critical factor for BI-2536 efficacy. Targeting AURKA with alisertib at a non-toxic concentration abolished BI-2536-induced BRCA1 and exerted a significant inhibitory effect on SCLC cells compared to BI-2536 alone, both in vitro and in vivo. Furthermore, we established the MYC/MYCN-RAD51 axis as a consistent determinant of response to BI-2636 through loss- and gain-of-function studies. Our research prompts that BRCA1 expression and the MYC/MYCN-RAD51 axis are pivotal determinants in the anti-cancer efficacy of BI-2536 and further proposes a novel combination therapeutic strategy for SCLC care.

*PLK1* and *AURKA* are commonly overexpressed and are recognized as promising therapeutic targets in a wide range of cancers, including for SCLC. In a phase II study, an AURKA inhibitor as monotherapy achieved a 21% objective response rate in patients with relapsed or refractory small-cell lung cancer [48]. Synergy observed between alisertib and paclitaxel led to a phase II study evaluating alisertib plus paclitaxel as a second-line therapy for SCLC [42]. This clinical trial indicated benefits for a subset of patients with high *MYC* expression. In contrast, BI-2536 has shown limited effectiveness in treating sensitive relapsed SCLC [49], with the mechanisms underlying lack of response yet to be fully elucidated. One possibility is that targeting PLK1 alone is insufficient to effectively block G2/M transition and DNA damage checkpoint pathways. Interestingly, BI-2536 has shown efficacy when combined with other chemotherapeutic agents [50]. Due to the deficient G1/S DNA damage checkpoint caused by inactivation of TP53 and RB1, SCLC cells heavily rely on the G2/M checkpoint for proliferation. Both AURKA and PLK1 play pivotal roles in the G2/M transition. Hence, co-targeting AURKA and PLK1 might achieve a robust response in SCLC cells. Indeed, pharmacological inhibition of PLK1 and AURKA demonstrated superior antitumor activity compared with either single drug treatment in nasopharyngeal carcinoma and was minimally harmful to normal epithelial cells. Similarly, in our study, the combination of BI-2536 and alisertib exhibited dramatic tumor-inhibitory effects in SCLC.

Molecular investigations showed that incubating cells with low concentrations of BI-2536 increased BRCA1 at both mRNA and protein levels (Figs. 1A, B and S5B). Previous studies have shown an antagonistic interplay between AURKA and BRCA1 [39]. Unexpectedly, we observed that AURKA inhibition counteracted the BI-2536-induced increase in BRCA1. BRCA1 plays a crucial role in the DNA damage repair pathway, and its accumulation following PLK1 inhibition by BI-2536 potentially facilitated DNA damage repair, thereby mitigating the deleterious impact of BI-2536. This observation could partially explain the limited effectiveness of BI-2536 as a monotherapy in SCLC, as observed in its Phase II clinical trial [2]. The enhancement of BI-2536’s efficacy by alisertib may be attributed to the reduction of BI-2536-induced BRCA1 and the suppression of both p-Chk1 level and Rad51. Notably, while BI-2536 and alisertib individually induced apoptosis, no significant increase in apoptosis was detected despite remarkable cell-killing upon co-targeting PLK1 and AURKA. Instead, a considerable proportion of cells underwent chromosome fragmentation with the combined treatment of BI-2536 and alisertib. It remains to be clarified whether this outcome is due to the significant accumulation of DNA damage or abnormal mitotic progression. The mechanisms by which cells succumb to chromosome fragmentation, as opposed to apoptosis, warrant further investigation.

Previous studies have established alisertib’s specificity and sensitivity towards high expression of *MYC* family genes [42, 43, 51]. PLK1 has also been identified as a potential therapeutic target in cells with elevated *MYC* gene expression [52–55]. Although this correlation is not absolute, it suggests that targeting PLK1 and AURKA could be effective in cancers characterized by high *MYC* family gene expression. Our cytotoxicity assays revealed that the sensitizing effect of alisertib on BI-2536 was specific and pronounced in SCLC cell lines with high *MYC/MYCN* expression. This study is the first to propose a joint therapeutic strategy targeting PLK1 and AURKA, elucidating the role of MYC/MYCN family genes in enhancing the efficacy of this drug combination. Thus, our findings hold significant implications for the treatment of SCLC characterized by elevated *MYC/MYCN* gene expression.

For the first time, our study demonstrates that in small cell lung cancer cells, the sensitivity to BI-2536 and the synergistic effect of its combination with alisertib are modulated by the MYC/MYCN-RAD51 axis. Although prior research has indicated that PLK1 inhibition can suppress RAD51 expression, our discovery elucidates the molecular mechanisms underlying drug sensitivity and synergistic enhancement. This insight lays the groundwork for developing targeted combination therapies for specific tumor cell types.

Currently, AURKA and PLK1 are at the forefront of research on cell cycle progression, showing potential in both preclinical and clinical studies. However, as monotherapies, PLK1 inhibitors have been somewhat disappointing. Therefore, pursuing new combinational treatment strategies for SCLC is of paramount importance [56]. Previous studies have suggested that the Aurora-PLK1 cascade acts in synergy with cyclin B-CDK1 to regulate cell cycle phase transition [26]. Additionally, Aurora A kinase is thought to not only have redundant functions with PLK1 but also to exert PLK1-independent roles during the cell cycle and in DNA damage repair [57, 58]. Based on these insights, our study explored the combination of PLK1 and AURKA inhibitors as a therapeutic strategy for SCLC. We have demonstrated, through in vitro and in vivo experiments, that alisertib significantly enhances the sensitivity of SCLC cells to BI-2536. Our findings provide a compelling rationale for considering the co-inhibition of PLK1 and AURKA in the treatment of SCLC.

## Materials and methods

### Cell culture, chemicals, and antibodies

Human small cell lung cancer cell lines NCI-H526 (H526), NCI-H82 (H82), NCI-H446 (H446), SHP77, and DMS273 were as described previously [59, 60]. All cell lines were propagated in RPMI-1640 supplemented with 10% (v/v) fetal bovine serum and 50 U/ml penicillin-streptomycin at 37 °C in a humidified incubator with 5% CO_2_ and 95% air atmosphere. Routine testing was conducted to ensure that the cells were free from mycoplasma contamination. PLK1 inhibitor BI-2536 and AURKA inhibitor alisertib were acquired from Selleckchem (Houston, TX, USA). These inhibitors were initially dissolved in DMSO (Sigma-Aldrich, Saint Louis, MO, USA) at suitable concentrations, stored at −20 °C, and later diluted in PBS to the desired concentrations for experiments. Antibodies against BRCA1 (#9010S), PARP (#9532S), Chk1 (#2360), p-Chk1 (#12302), γ-H2AX (Ser139) (#7631), MYC (#5605), MYCN (#84406) and Ki67 (#9449) were obtained from Cell Signaling Technology (Danvers, MA, USA). Rad51 (ab133534) was sourced from Abcam (Abcam, Cambridge, UK). Antibody against β-Actin (T0022) was purchased from TransBionovo (Beijing, China).

### Microarray, RNA-seq, and genomic datasets and data analysis

Normalized gene expression from microarray analysis was downloaded from the Gene Expression Omnibus database, specifically from GSE149507 and GSE60052 (http://www.ncbi.nlm.nih.gov/geo). Additionally, microarray and RNA-seq data encompassing 50 SCLC cell lines were sourced from the Cancer Cell Line Encyclopedia (https://portals.broadinstitute.org/ccle/data). Differentially expressed genes between BI-2536 sensitive and insensitive were identified using an unpaired Student’s *t* test with a *p*-value threshold of 0.05. The top 30 up- and downregulated genes identified were visualized in a heatmap. Expression data for *PLK1*, *AURKA*, and *RAD51* genes were retrieved, analyzed, and visually presented in scatter plots. Gene set enrichment (GSEA) analysis was conducted using the clusterProfiler (version 4.4.4) and enrichplot (version 1.16.1) R packages. This analysis utilized RNA-seq data from 50 SCLC cell lines and pre-defined gene sets from the MSigDB Hallmark (h.all.v2022.1.Hs.symbols.gmt) [61]. Specifically, the Hallmarks and chemical and genetic perturbations in the C2 collection were searched to pinpoint signatures associated with BI-2536 sensitivity. The genetic alterations of *PLK1* and *AURKA* in SCLC specimens and cell lines were examined through the cBioPortal database (https://www.cbioportal.org/).

### Cell viability assay

Three thousand cells were seeded into each well of a 96-well plate and cultured overnight. The following day, the cells were exposed to 0.1% dimethyl sulfoxide (DMSO) or nine different concentrations of BI-2536, as well as a combination of BI-2536 and alisertib, for 24 h. Cell viability was measured using the CellTiter-Glo luminescent assay, following the manufacturer’s instructions. The luminescence signal was recorded using an Envision multilabel plate reader (Envision PerkinElmer, USA).

### Flow cytometry

Twenty-four hours after exposure to 0.1% DMSO, BI-2536, or a combination of BI-2536 and alisertib, the cells were collected, fixed with 80% ethanol at −20 °C for 24 h. Following fixation, the cells were then re-suspended and stained with PI/RNase staining buffer (BD Pharmingen, San Diago, CA, USA) for 30 min at room temperature. Cell cycle analysis was conducted using a FACS Calibur (Sony Biotechnology, San Jose, CA, USA). The data obtained from this analysis were subsequently processed and analyzed using FlowJo V10 software (FlowJo LLC, Ashland, Oregon, USA).

### siRNA and plasmid transfection

siRNA sequences against *MYC*/*MYCN* and *RAD51* were designed using the siDirect, a versatile web-based tool (https://sidirect2.rnai.jp/) and synthesized by General Biol (Anhui) Co., Ltd. (Chuzhou, China) with sequences as follows:

siControl, 5’- UUCUCCGAACGUGUCACGUTT-3’,

siMYC-1#, 5’- GCUUGUACCUGCAGGAUCU-3’,

siMYC-2#, 5’-GAGGAUAUCUGGAAGAAAU-3’,

siMYCN-1#, 5’-AUGACGCUGAUACAUAACUAA-3’,

siMYCN-2#, 5’-CGUGCCGGAGUUGGUAAAGAA-3’,

siRAD51-1#, 5’-UGUAGCAUAUGCUCGAGCG-3’,

siRAD51-2#, 5’-CUGGACUUCCAGAAGAACA-3’,

siBRCA1-1#, 5’-GCGUGCAGCUGAGAGGCAU-3’,

siBRCA1-2#, 5’- UUCUCAAUGGCGCAAAUGGAU-3’.

The *c-MYC* and *RAD51* genes were amplified by PCR and inserted into the pWZL vector [45]. pLVX-tight-Puro-BRCA1 was obtained from Addgene (Addgene plasmid #119281). Transfections with siRNA and plasmid transfection were executed using Effectene transfection reagent (Qiagen, Cat. No. 301425 and 301427, Germany), adhering to the manufacturer’s protocols. Following a transfection period of 24 or 48 h, cells were collected for drug treatment or further experimental procedures. The efficiency of gene silencing was assessed using RT-qPCR.

### Comet assay

After SCLC cells were incubated with the specified small molecules for 20 h, the cells were analyzed for DNA strand breaks using established methods [62]. Cells embedded in agarose gel on slides underwent electrophoresis at 25 V and 300 mA for 20 min at 4 °C under alkaline-denaturing conditions. After staining with 1% Gold View at 4 °C for 10 min, the cleaned glass slides were then viewed and photographed under a fluorescence microscope.

### Immunofluorescence staining and microscopy

After drug treatment, cells were fixed in 75% ethanol for 10 min and subsequently in freshly prepared 4% paraformaldehyde for 30 min, followed by three washes with PBS. The fixed cells were then permeabilized with PBS containing 0.3% Triton X-100 for 15 min, then blocked for 1 h in 5% BSA blocking solution. After blocking, the cells were incubated with primary antibodies followed by incubation with Alexa Fluor 488 conjugated secondary antibodies. Images were acquired using an upright fluorescent microscope. Cells exhibiting more than 5 foci per cell were classified as positive.

### Protein extraction and western blot analysis

SCLC cells were lysed in RIPA buffer containing protease and phosphatase inhibitors at 4 °C for 30 min. After centrifuging at 13,000 rpm for 15 min, proteins in the supernatants were quantified using the Green Sky BCA protein quantification kit according to the manufacturer’s instructions. The quantified proteins were separated by 10% SDS-PAGE. Subsequently, the separated proteins were transferred onto a polyvinylidene difluoride (PVDF) membrane using an electroblotting apparatus at 120 V for 90 min. The membrane was then probed by primary antibodies overnight at 4 °C, followed by 1-h incubation with HRP-conjugated anti-rabbit secondary antibodies at room temperature. Finally, chemiluminescent signals were detected using the ECL plus chemiluminescent substrate kit.

### Mouse xenograft model

Mice were housed and cared at the Hefei Institutes of Physical Science Laboratory Animal Center, Chinese Academy of Sciences. All experimental procedures received approval from the Hefei Institute of Physical Science Animal Care Use Committee. Athymic nu/nu mice, frequently used in tumor grafting studies, were chosen to examine tumorigenicity. A total of 5 × 10^6^ SCLC cells, suspended in a 100 μL volume, were mixed with an equal volume of Matrigel (BD Biosciences, Franklin, NJ, USA) and inoculated into the dorsal flank of a 4–6 week-old nu/nu mice. Once the tumors became palpable and measurable, mice were randomized into four groups and treated with either DMSO control, BI-2536 alone, alisertib alone, or a combination of BI-2536 and alisertib. BI-2536 (10 mg/kg) was administered via tail vein injection every other day for a total of five doses. Alisertib (20 mg/kg) was given orally every other day for a total of five doses. Each animal was tracked individually every other day for tumor growth by external caliper measurements of the protruding subcutaneous tumors. The tumor size was calculated using the following formula: Volume = (length × width^2^)/2.

### Reverse transcription and real-time fluorescence quantitative PCR

Total RNA was prepared using the RNeasy Mini RNA Kit (Qiagen, Hilden, Germany). Prior to cDNA synthesis genomic DNA was removed to ensure purity. For cDNA synthesis, 1 µg of RNA was processed with the Transcriptor First Strand cDNA Synthesis Kit (Roche, Mannheim, Germany), following the manufacturer’s guidelines. The expression levels of *BRCA1*, *RAD51*, and *CHEK1* was measured using a real-time quantitative PCR assay performing on a Roche LightCycler 96 Real-Time PCR System (Roche Diagnostics, Switzerland). β-actin was employed as an internal control to normalize the gene expression data. The primer sequences used for this assay are detailed below:

**Table:** 

*RAD51*	Forward primer	5’- CCTCCTCTTTAACGCCTCCTG -3’
	Reverse primer	5’- GGGGACAACTCCCAGACTTTTT-3’
*BRCA1*	Forward primer	5’- ACCTTGGAACTGTGAGAACTCT-3’
	Reverse primer	5’- TCTTGATCTCCCACACTGCAATA-3’
*CHEK1*	Forward primer	5’- CCAGTAAACAGTGCTTCTAG-3’
	Reverse primer	5’- TCTTCAGGAAGTGTCTCTTGC-3’
*MYC*	Forward primer	5’- TCCCTCCACTCGGAAGGAC-3’
	Reverse primer	5’- CTGGTGCATTTTCGGTTGTTG-3’
*MYCN*	Forward primer	5’- CACGTCCGCTCAAGAGTGTC-3’
	Reverse primer	5’- GTTTCTGCGACGCTCACTGT-3’
*ACTB*	Forward primer	5’- CATGTACGTTGCTATCCAGGC-3’
	Reverse primer	5’- CTCCTTAATGTCACGCACGAT-3’

### Histological and immunohistochemical analyses

Xenograft tumors were fixed in 4% paraformaldehyde solution and subsequently embedded in paraffin. For histopathological examination, 4-micrometer sections were prepared and stained with hematoxylin and eosin. Immunohistochemical staining was performed on serial sections using antibodies against Ki67 (1:1000, CST 9449), cleaved Caspase 3 (CC3) (1:300, CST 9661), Rad51 (1:200, Abcam ab133534), and γH2AX (1:500, Ser139, CST 2577). The stained sections were then mounted and photographed with a Leica microscope (Leica Microsystems, Germany).

### Quantification and statistical analysis

All in vitro experiments were conducted at least three times to ensure reproducibility. Data are presented as mean values ± SD. Comparisons between 2 groups were made with a two-tailed Student’s *t* test. Differences were considered statistically significant at *p* < 0.05, with significance levels indicated as follow: *p < 0.05, **p < 0.01, ***p < 0.001, ****p < 0.0001.

### Supplementary information

Supplementary Figure legends

Supplementary Figure S1–9

Original Western Blots

Supplementary Table 1

Supplementary Table 2

Supplementary Table 3

Supplementary Table 4

Supplementary Table 5

Supplementary Table 6

## Data Availability

The data that support the findings of this study are available from the corresponding authors upon reasonable request.
