# Supplementary material for: BRCA1 orchestrates the response to BI-2536 and its combination with alisertib in MYC-driven small cell lung cancer
Source: Cell Death Dis. 2024 Jul 31;15(7):551. doi: 10.1038/s41419-024-06950-w (PMC11291995; doi:10.1038/s41419-024-06950-w)
Supplement: Supplementary file 1 — Supplementary Figure legends [file 41419_2024_6950_MOESM1_ESM.pdf]

**Supplementary Figure legends**

**Figure S1 Effects of BRCA1 manipulation and Bractoppin.**

**(A)** RT-qPCR analysis showing efficient silencing and overexpression of BRCA1 in the cell viability assays. The data were shown as mean ± SD, ** $P < 0.01$; *** $P < 0.001$.

**(B)** Cell viability assays showing the cytotoxic effects of Bractoppin on four SCLC cell lines examined.

**Figure S2 Expression and prognostic value of PLK1 in small cell lung cancer**

**(A)** Scatter plots showing the expression of PLK1 in 50 small cell lung cancer cell lines compared to 75 lung adenocarcinoma cell lines (upper left panel, microarray dataset), 50 small cell lung cancer cell lines compared to 75 lung adenocarcinoma cell lines (upper right panel, RNA-seq dataset), 75 small cell lung cancer tissues compared to 6 normal tissues, and 18 paired small cell lung cancer and adjacent normal tissues. Data are presented as the mean ± SD, * $P < 0.05$, ** $P < 0.01$, **** $P < 0.0001$.

**(B)** Kaplan-Meier analysis showing that the high expression of PLK1 was positively correlated with the overall survival and progression free survival in 77 (left panel) or 33 (right panel) patients with SCLC, respectively. Statistical significance was determined by log-rank tests.

**(C), (D)** Oncoprints of PLK1 alterations in 249 human primary SCLC clinical specimens (C) and 50 SCLC cell lines (D).

**Figure S3 Mutations and expression comparison of AURKA in small cell lung cancer**

**(A), (B)** Oncoprints of AURKA alterations in 249 human primary SCLC clinical specimens (A) and 50 SCLC cell lines (B).

**(C)** Scatter plots showing the expression of AURKA in 75 small cell lung cancer tissues compared to 6 normal tissues (left panel), and 18 paired small cell lung cancer and adjacent normal tissues (right panel). Data are presented as the mean ± SD, * $P < 0.05$, ** $P < 0.01$, **** $P < 0.0001$.

**Figure S4 Analysis of cell cycle, apoptosis and DNA damage after Plk1 and aurka alone or in combination**

**(A)** The effect of PLK1 and AURKA as single agent or in combination on cell cycle. The cells treated with the drugs in each group were made in triplicate, and three experiments were carried out independently. Data are presented as the mean ± SD, ns, no significance, * $P < 0.05$, ** $P < 0.01$.

**(B), (C), (D)** Western blot analysis of cleaved PARP (B&C) and γH2AX (D) following the treatment with PLK1 and AURKA alone or in combination in SCLC cells. SCLC cells treated with drugs as indicated for 14 h (B&D) or 24 h (C). β-Actin was used as a loading control.

**Figure S5 The effect of BI-2536 and alisertib on the mRNA level of DNA damage repair pathway genes and on the protein stability.**

**(A)** Western blot analysis of cleaved PARP, γH2AX and phosphorylated-ChK1, ChK1, BRCA1, and Rad51 following the treatment with PLK1 and AURKA alone or in combination in SCLC cells. SCLC cells treated with drugs as indicated for 24 h. β-Actin was used as a loading control.

**(B)** Effect of BI-2536 and alisertib on the expression of BRCA1 and RAD51. The mRNA level was detected by RT-qPCR, and the mRNA levels in cells with drug treatment were normalized by that in control. Data are presented as the mean ± SD. ns, no significance; * $P < 0.05$; ** $P < 0.01$; *** $P < 0.001$; **** $P < 0.0001$.

**(C)** Western blot analysis of BRCA1 and Rad51 in SCLC cells treated with indicated drugs for 14 h. β-Actin was detected as a loading control. Bortezomab (BTZ, 10 nM), a proteasome inhibitor, was used to inhibit the degradation of intracellular proteins.

**Figure S6 Body weight change of mice bearing DMS273 (A) and H82 (B) tumors**

**Figure S7 Impact of MYC on the sensitizing effect of targeted PLK1 and AURKA**

**in SCLC**

**(A)** GSEA showing enrichment of MYC/MYCN gene set derived from the comparison of other types of cancer and normal cells.

**(B, C)** Dose response curves representing cell viability of BI-2536 in four SCLC cell lines following siRNA-mediated MYC knockdown **(B)** or MYC overexpression **(C)**

**Figure S8 Impact of MYC on the sensitizing effect of targeted PLK1 and AURKA in SCLC**

**(A)** Growth inhibition curves for BI-2536 monotherapy and in combination with alisertib. The cells were treated with different concentrations of BI-2536 alone or combined with 20 nM alisertib for 24 h. The cell viability was determined by the CellTiter-Glo assay.

**(B)** Growth inhibition curves for BI-2536 monotherapy and in combination with alisertib following MYC overexpression. The cells were treated with different concentrations of BI-2536 alone or combined with 20 nM alisertib for 24 h. The cell viability was determined by the CellTiter-Glo assay.

**Figure S9 Expression of MYC/MYCN and RAD51 upon MYC/MYCN or RAD51 knockdown (KD) and MYC or RAD51 ectopic expression**

**(A), (B)** RT-qPCR analysis showing efficient silencing of MYC and MYCN (A) and overexpression of MYC (B) in the cell viability assays. The data were shown as mean ± SD, ** $P < 0.01$; *** $P < 0.001$. Gene expression was normalized to β-actin. The quantitative data represent mean ± S.E.M.

**(C), (D), (E)** Western blot analysis of MYC in SCLC cells upon MYC silencing **(C)** or overexpression **(D)** and MYCN in SCLC cells upon MYCN knockdown **(E)**. β-Actin was used as a loading control.

**(F)** RT-qPCR analysis showing efficient silencing of RAD51 and overexpression of RAD51 in the cell viability assays. The data were shown as mean ± SD, ** $P < 0.01$; *** $P < 0.001$.
