# Supplementary figures and images for: BRCA1 orchestrates the response to BI-2536 and its combination with alisertib in MYC-driven small cell lung cancer
Source: Cell Death Dis. 2024 Jul 31;15(7):551. doi: 10.1038/s41419-024-06950-w (PMC11291995; doi:10.1038/s41419-024-06950-w)

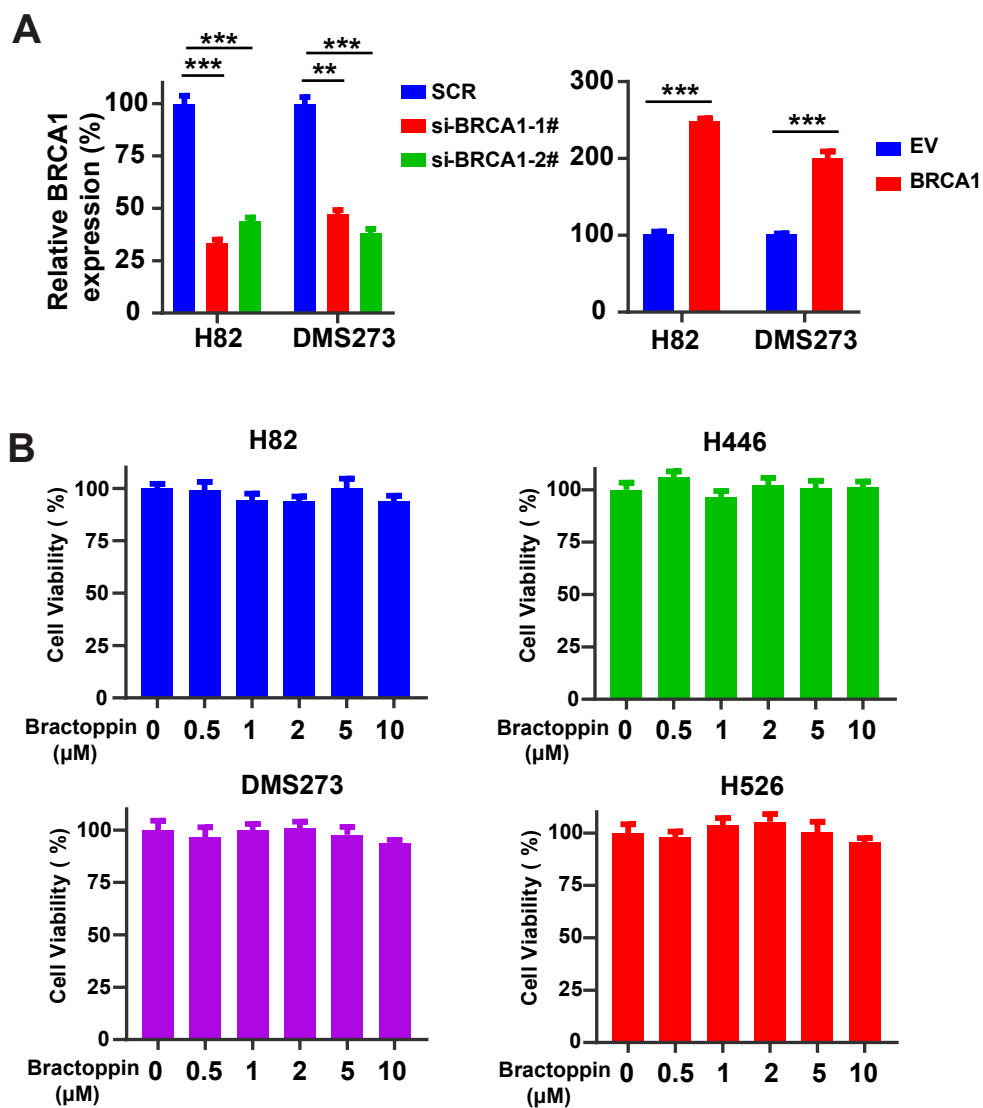

**Figure S1.**

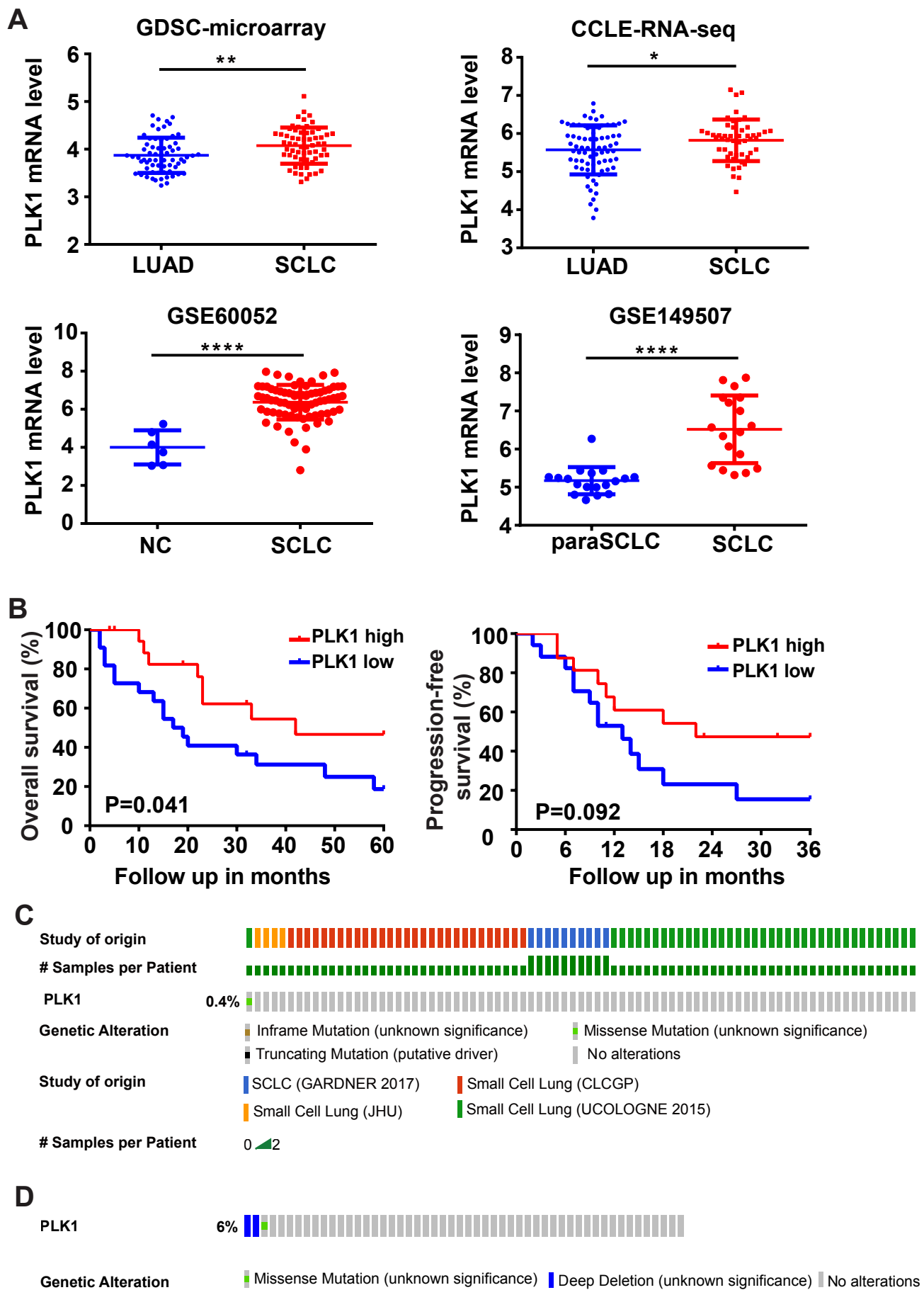

**Figure S2.**

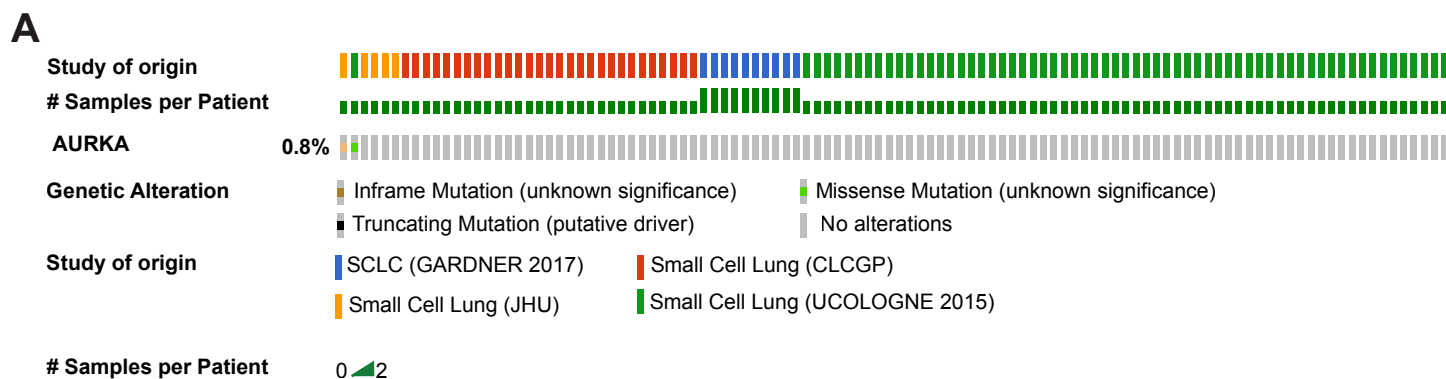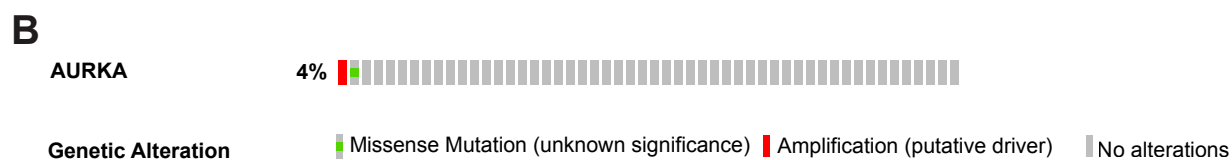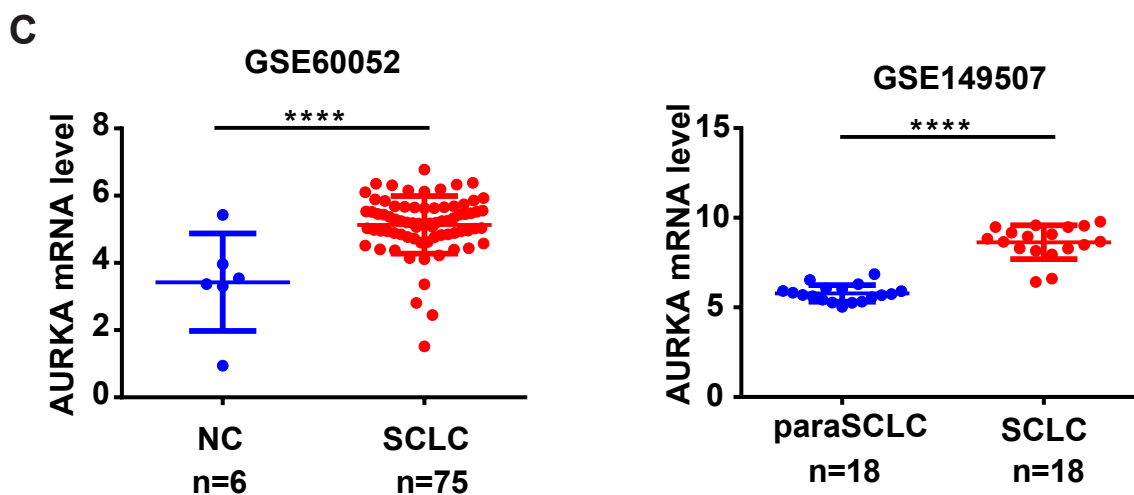

Figure S3.

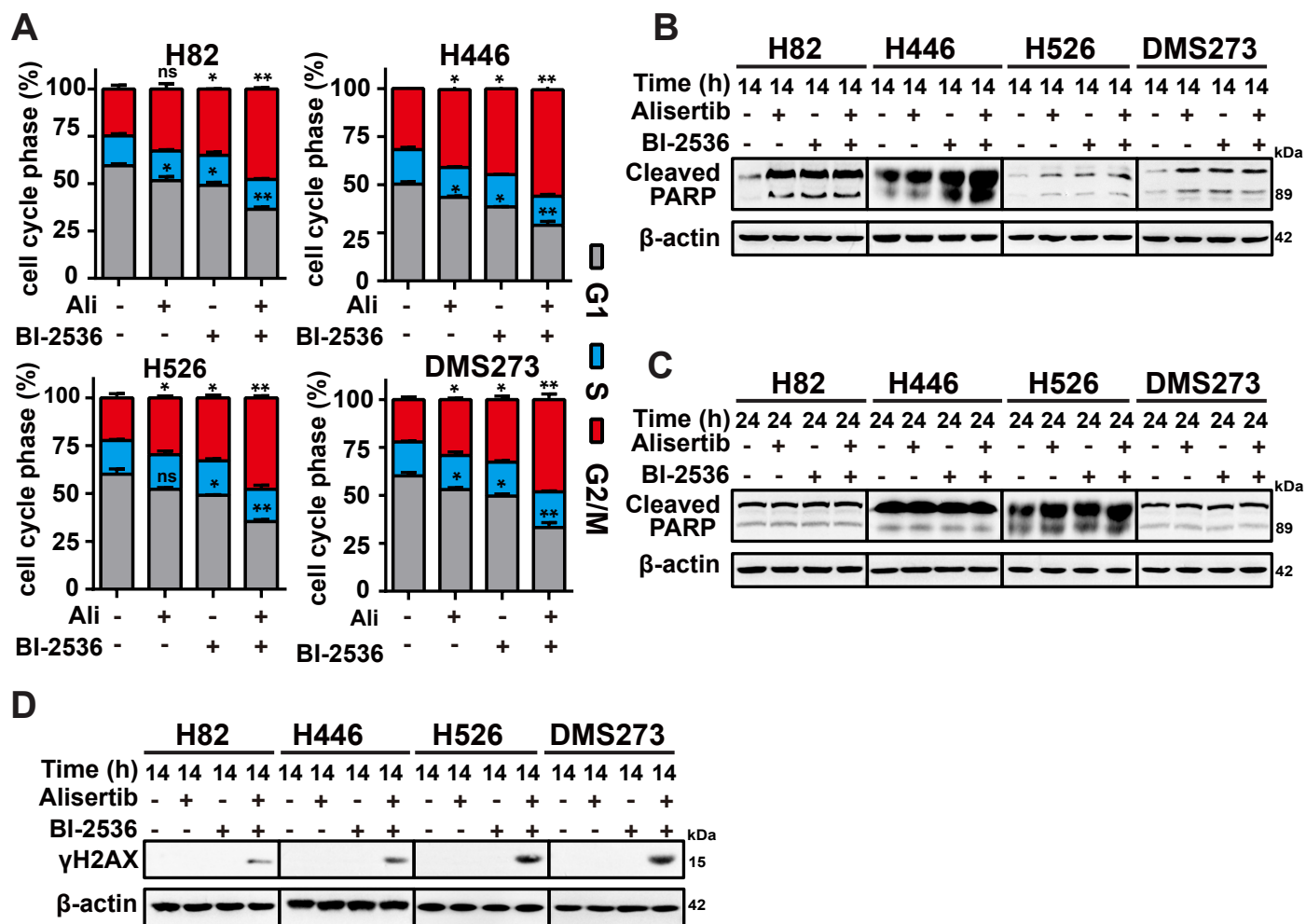

**Figure S4.**

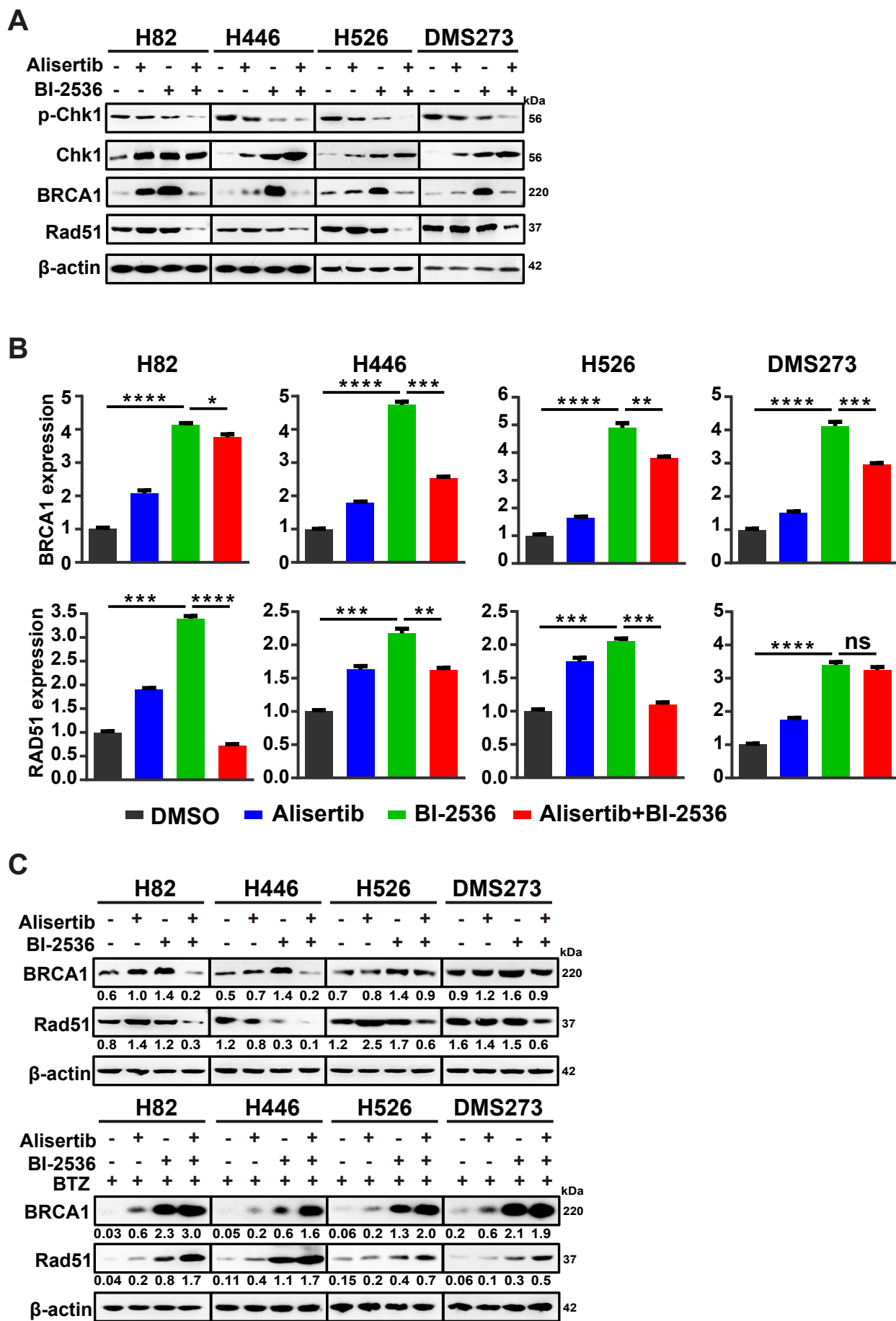

Figure S5.

**A**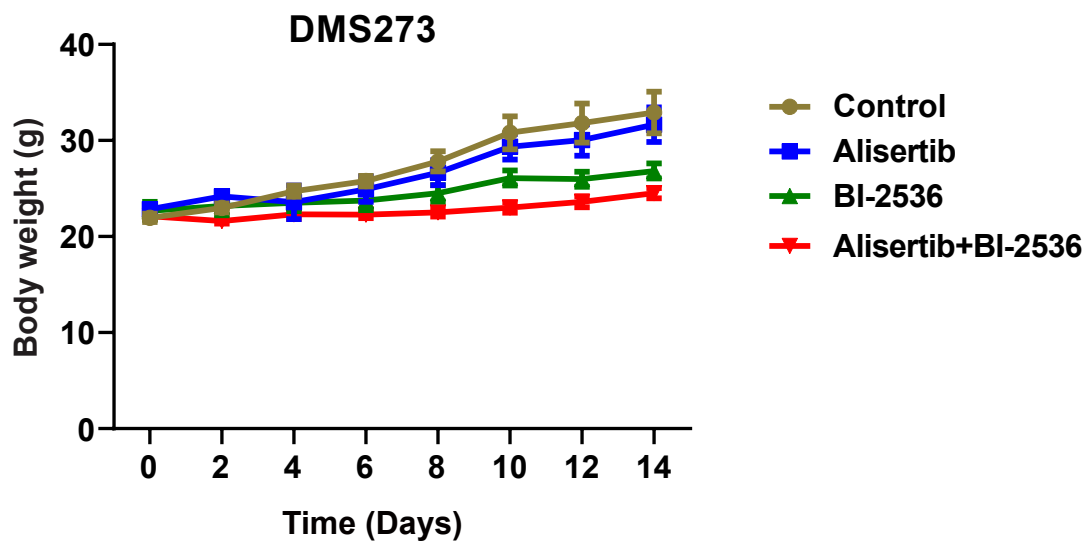**B**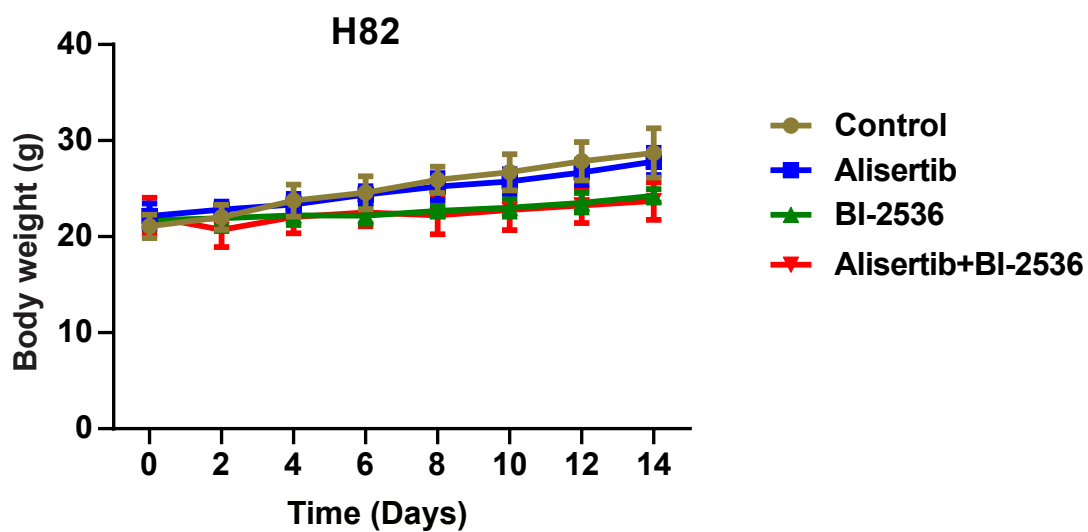**Figure S6.**

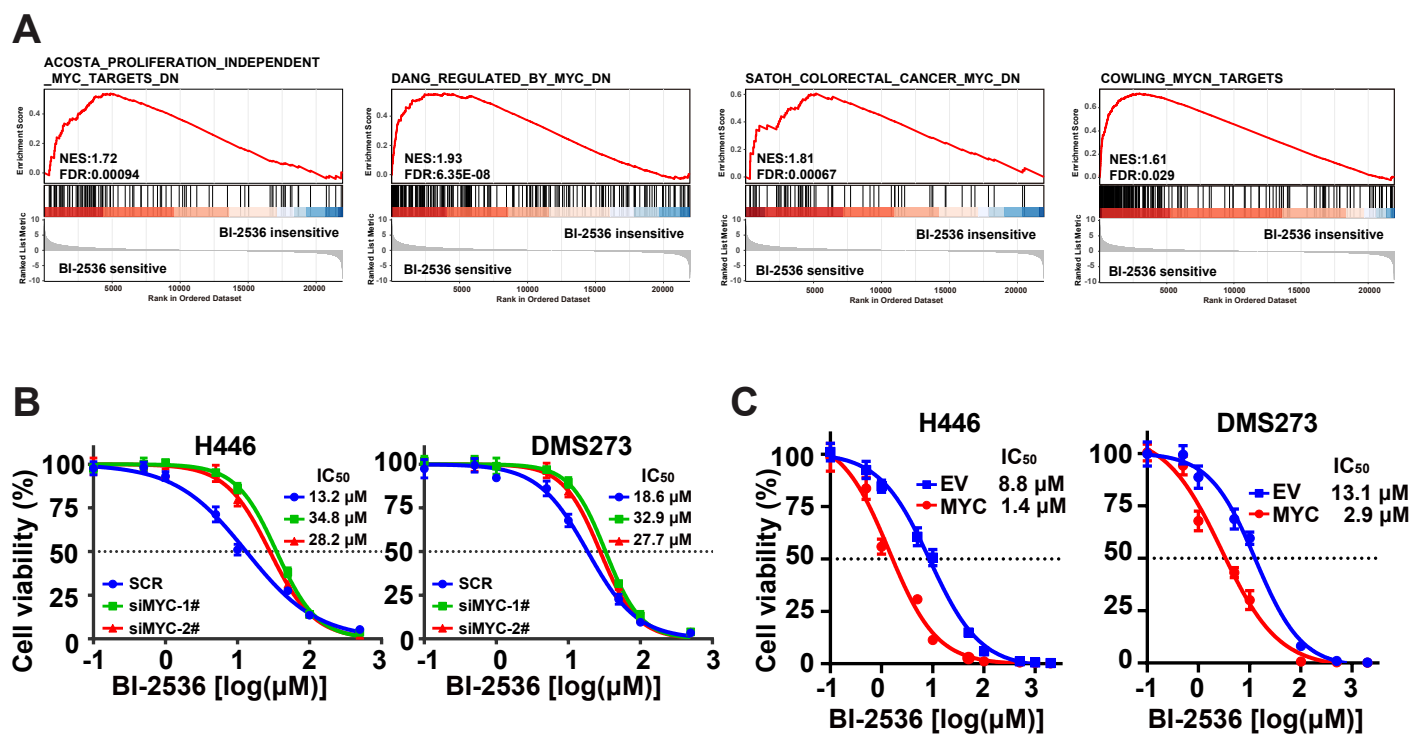

Figure S7.

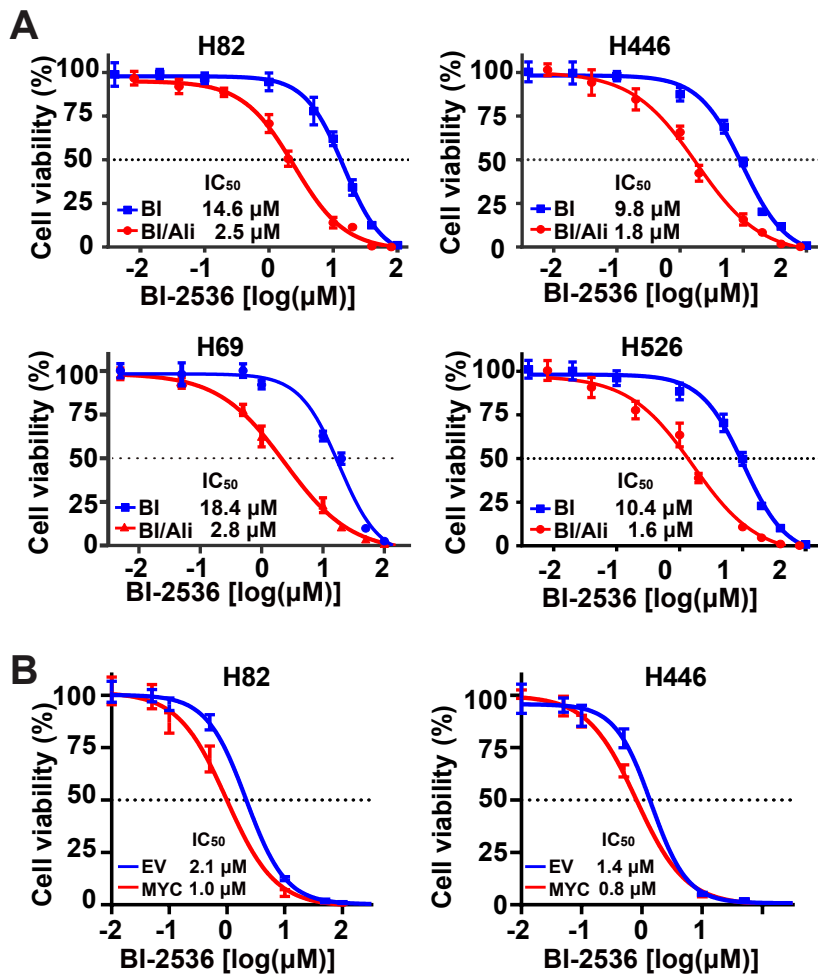

**Figure S8.**

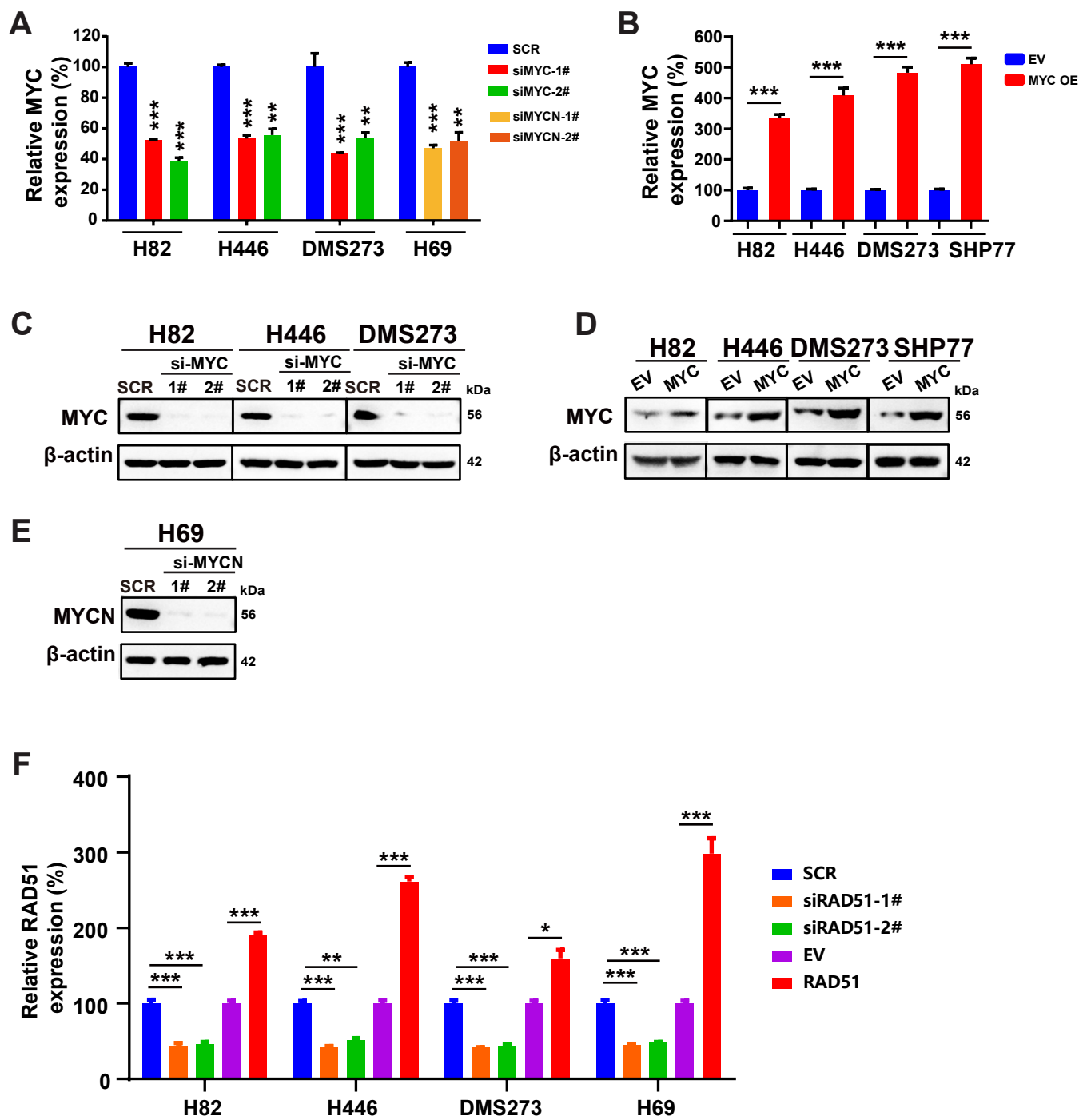

**Figure S9.**

Supplement: Supplementary file 2 — Supplementary Figure S1–9 [file 41419_2024_6950_MOESM2_ESM.pdf]
