# Supplementary material for: BRCA1 orchestrates the response to BI-2536 and its combination with alisertib in MYC-driven small cell lung cancer
Source: Cell Death Dis. 2024 Jul 31;15(7):551. doi: 10.1038/s41419-024-06950-w (PMC11291995; doi:10.1038/s41419-024-06950-w)
Supplement: Supplementary file 3 — Original Western Blots [file 41419_2024_6950_MOESM3_ESM.pdf]

Original Western Blots

■ Original Western Blots of Figure 1-A

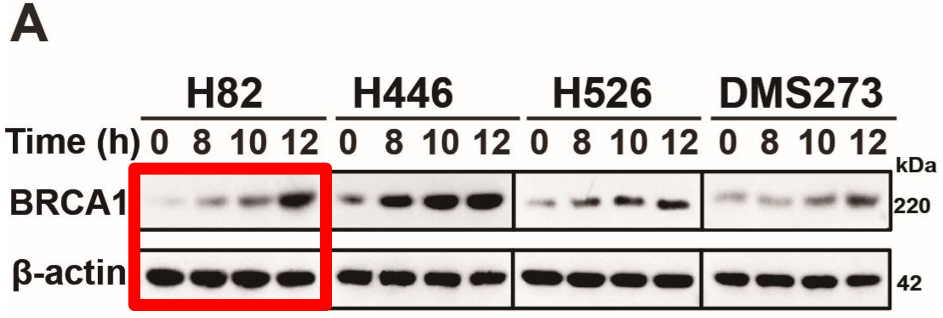

H82-βActin

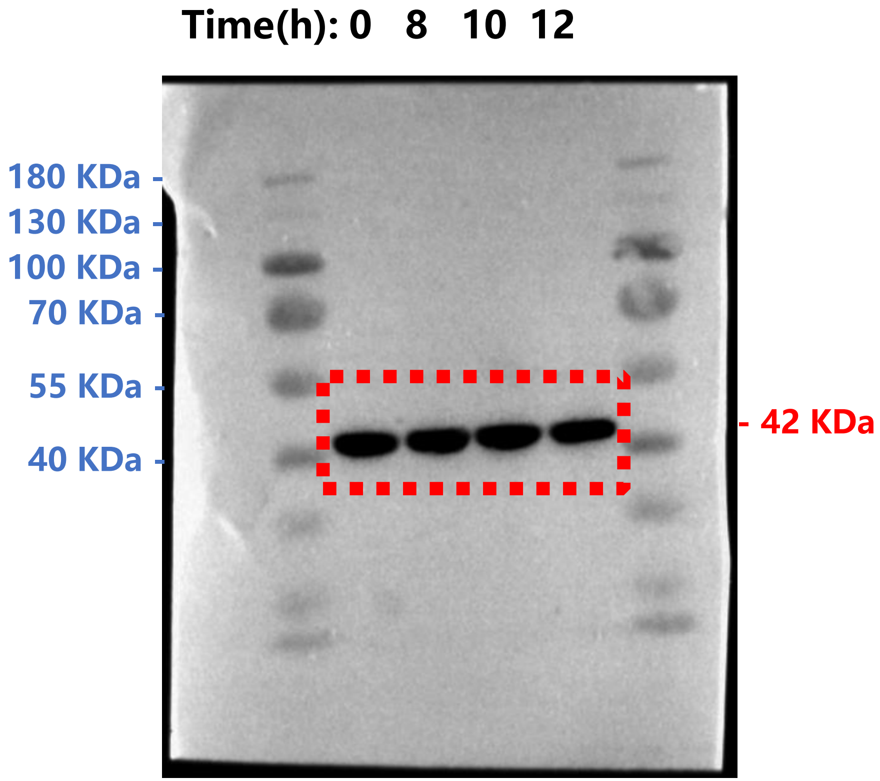

H82-BRCA1

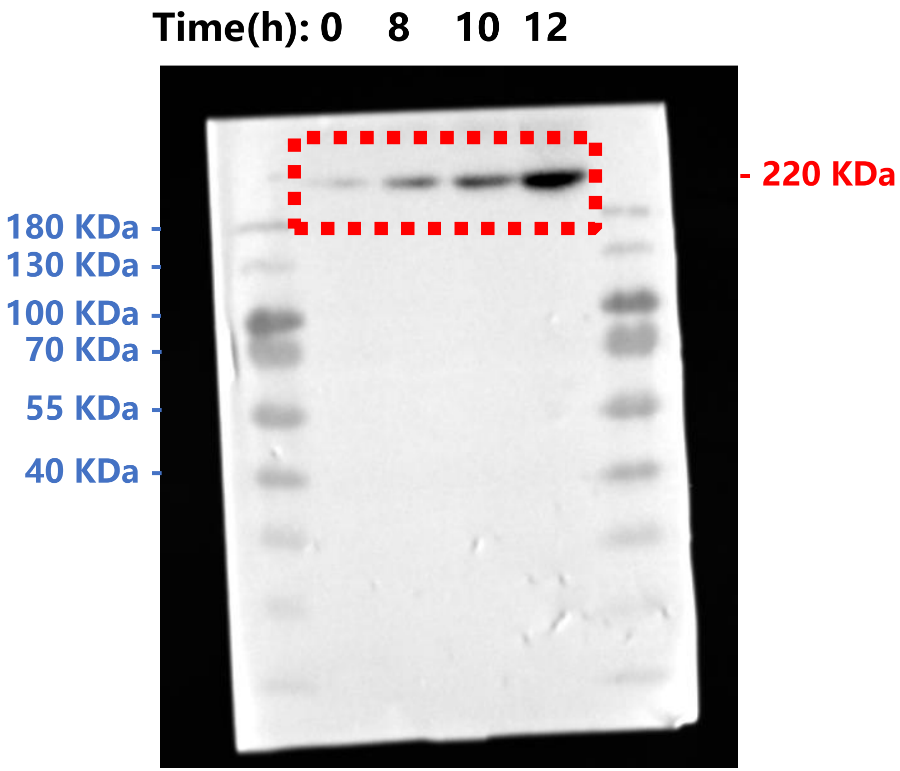

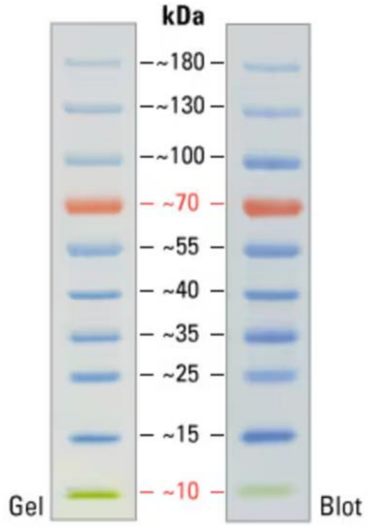

Thermo Scientific™ Marker-26617

■ Original Western Blots of Figure 1-A

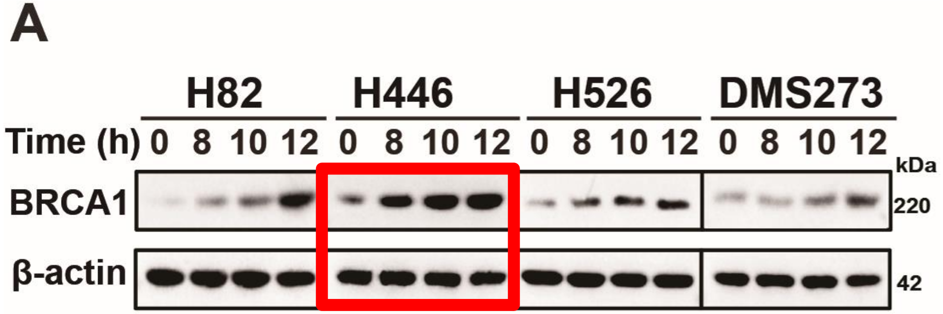

H446-BRCA1

Time(h): 0 8 10 12

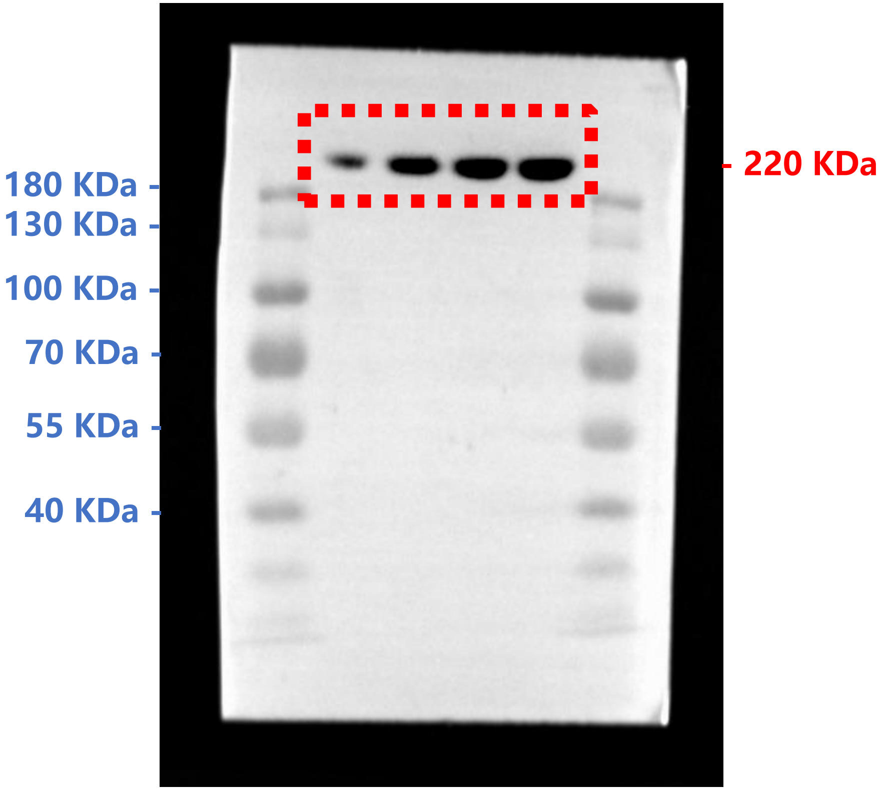

H446-βActin

Time(h): 0 8 10 12

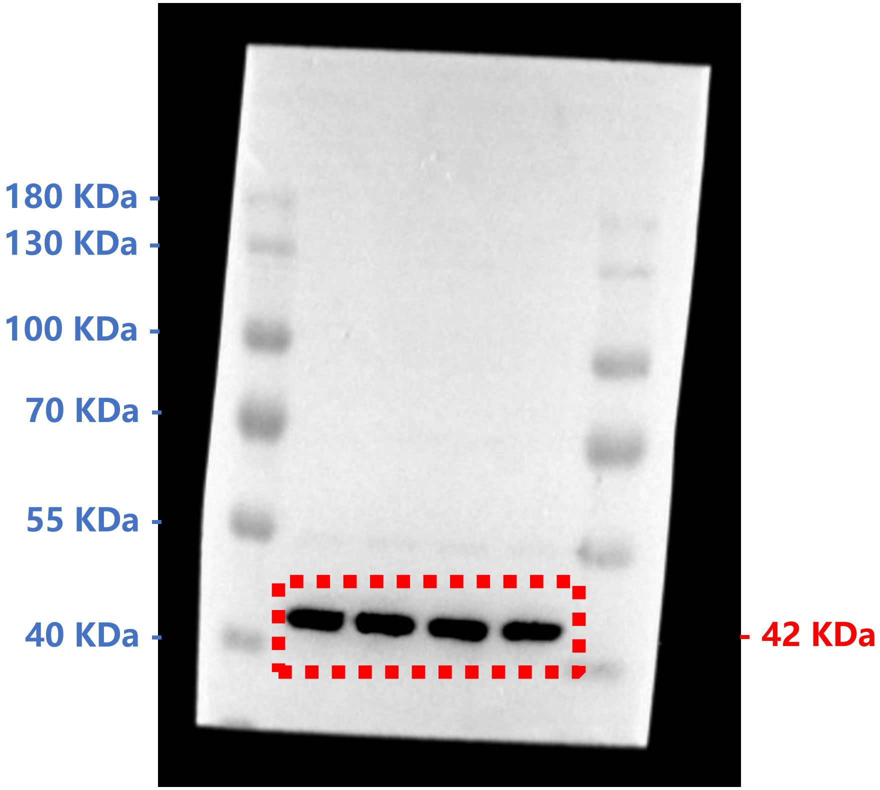

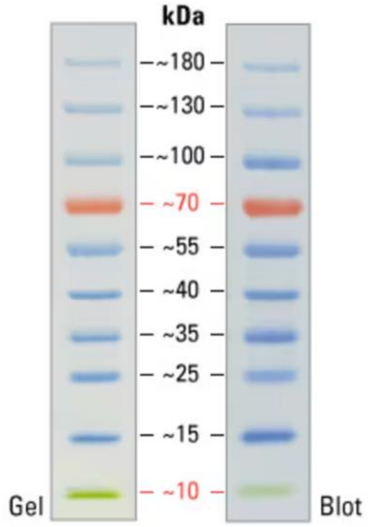

Thermo Scientific™ Marker-26617

■ Original Western Blots of Figure 1-A

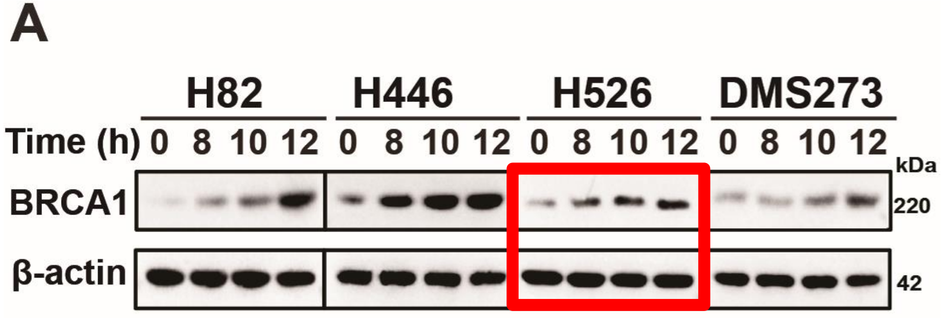

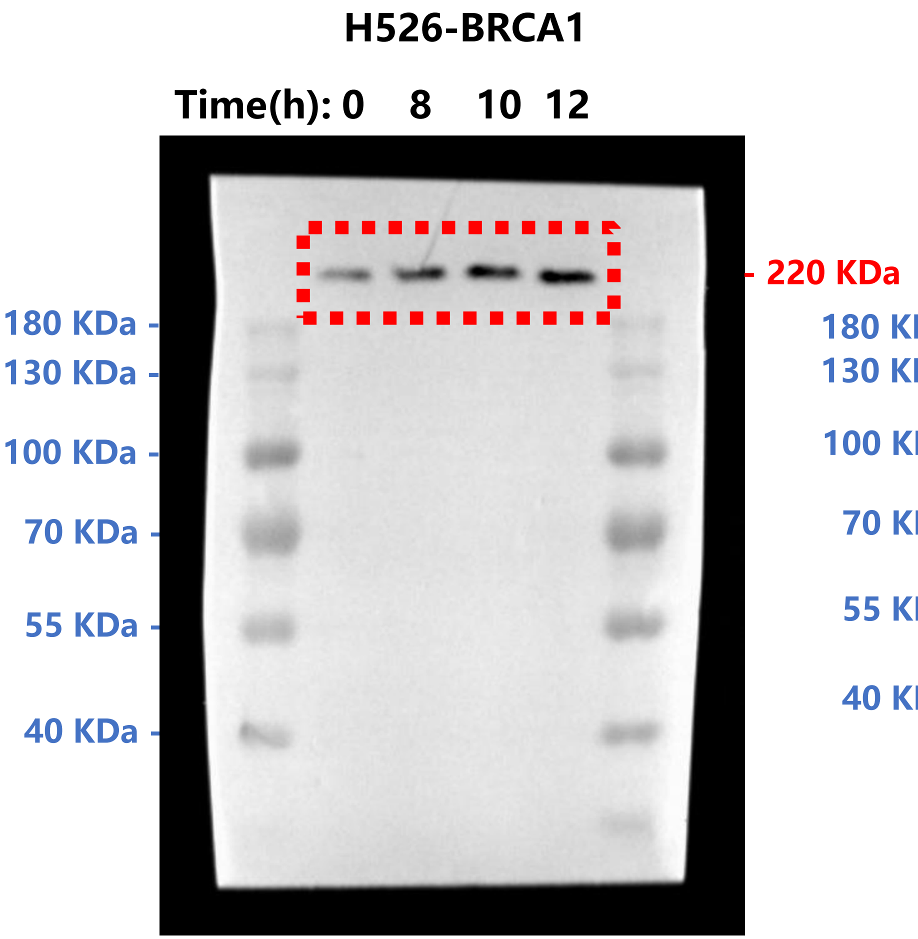

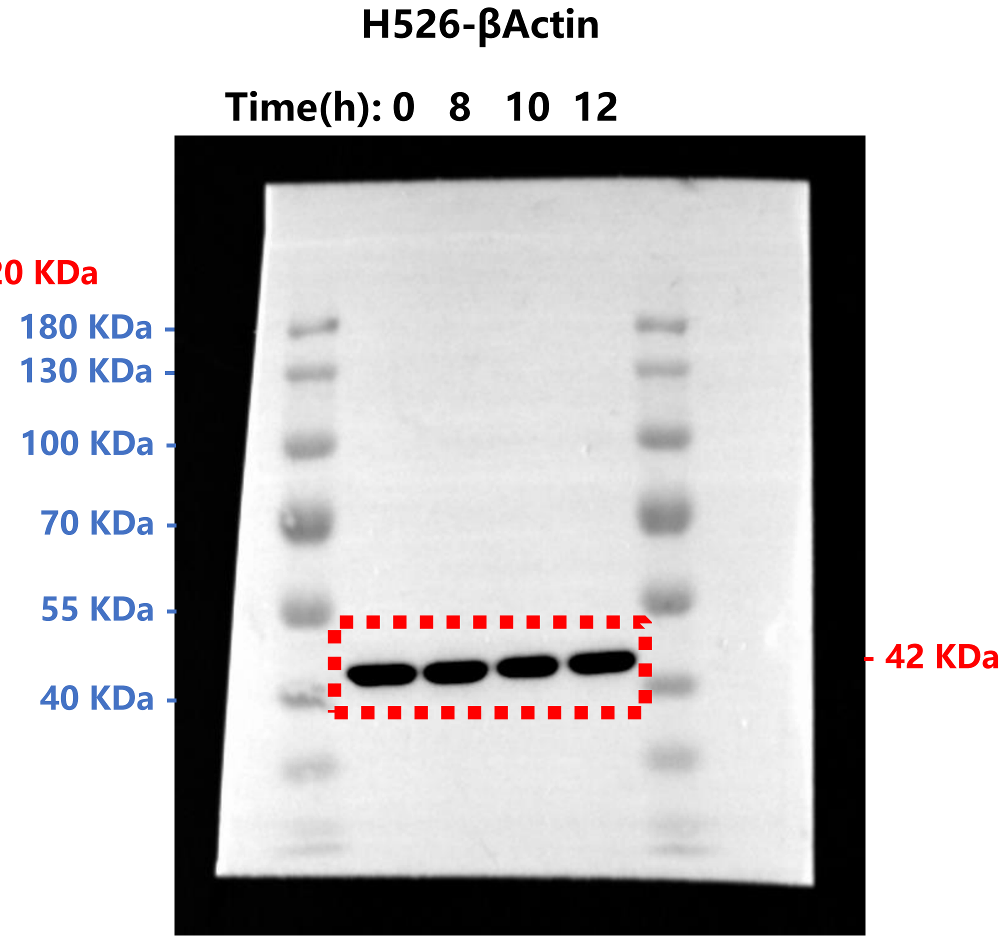

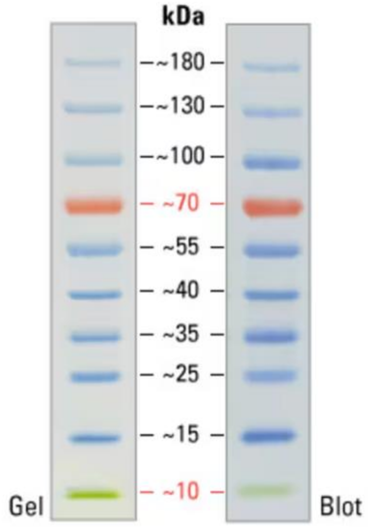

Thermo Scientific™ Marker-26617

■ Original Western Blots of Figure 1-A

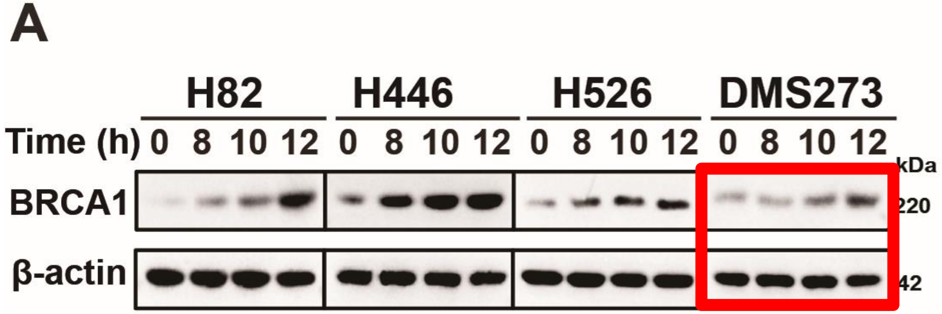

DMS273-BRCA1

Time(h): 0 8 10 12

DMS273-βActin

Time(h): 0 8 10 12

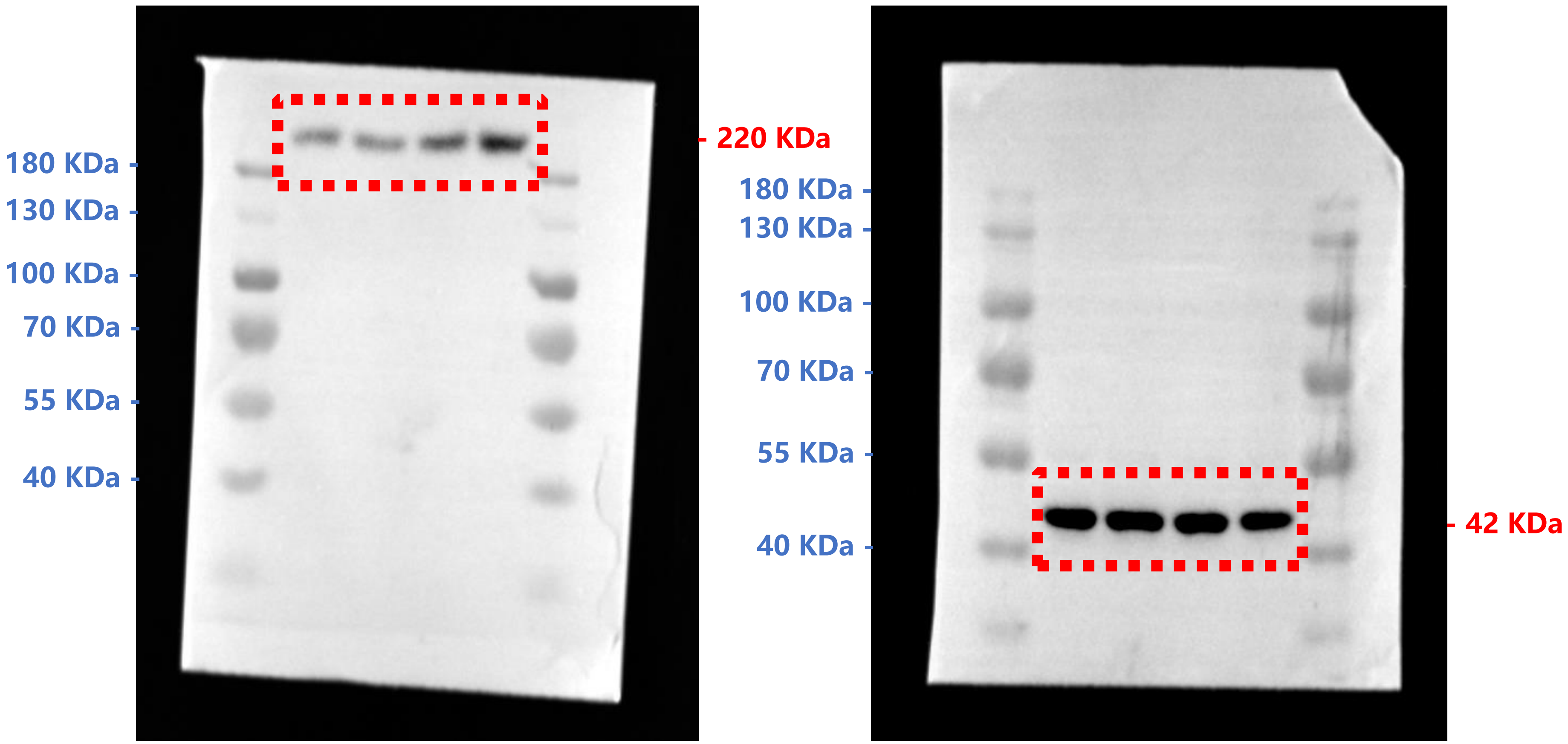

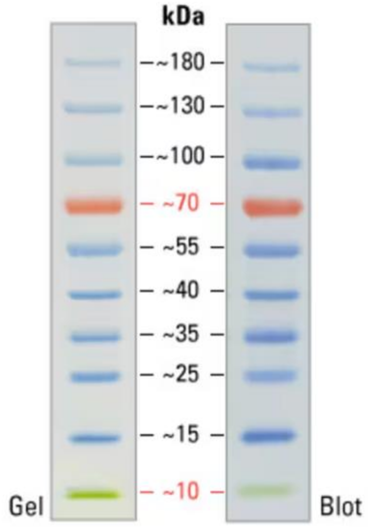

Thermo Scientific™ Marker-26617

■ Original Western Blots of Figure 1-B

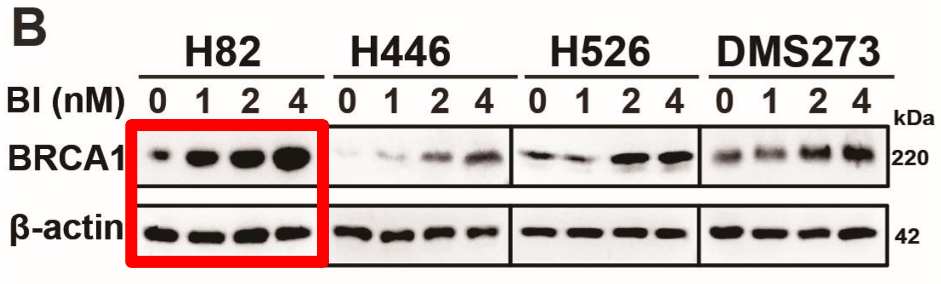

H82-BRCA1

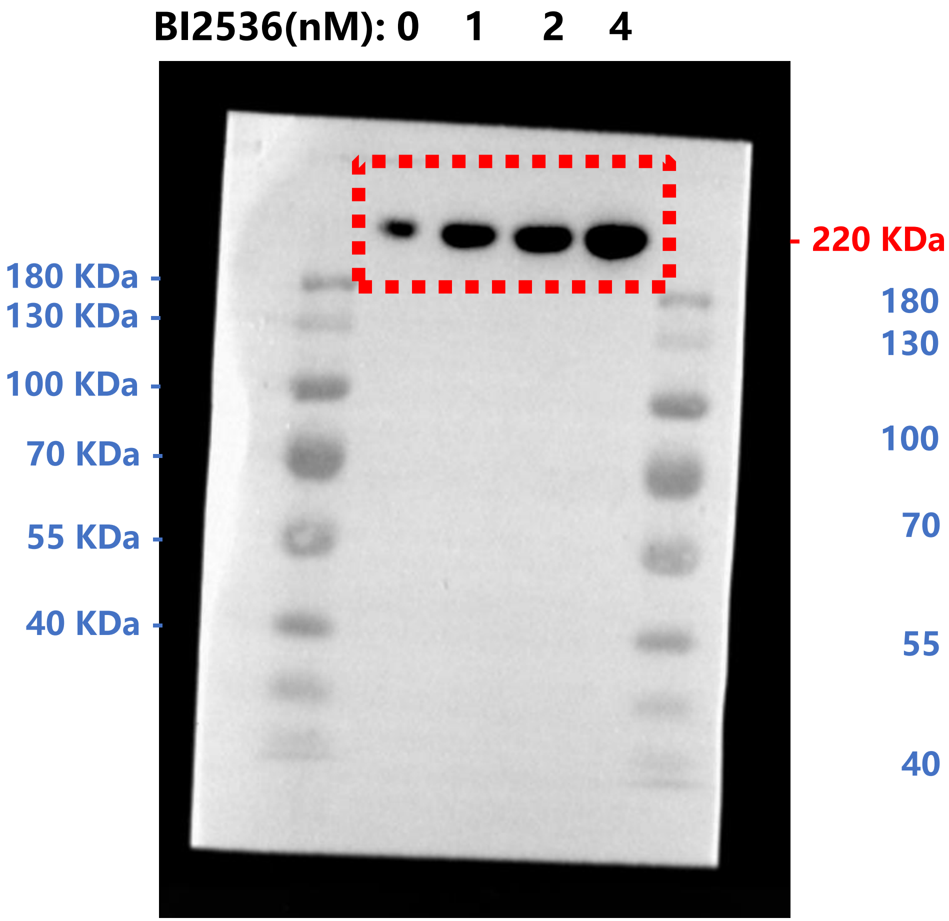

H82-βActin

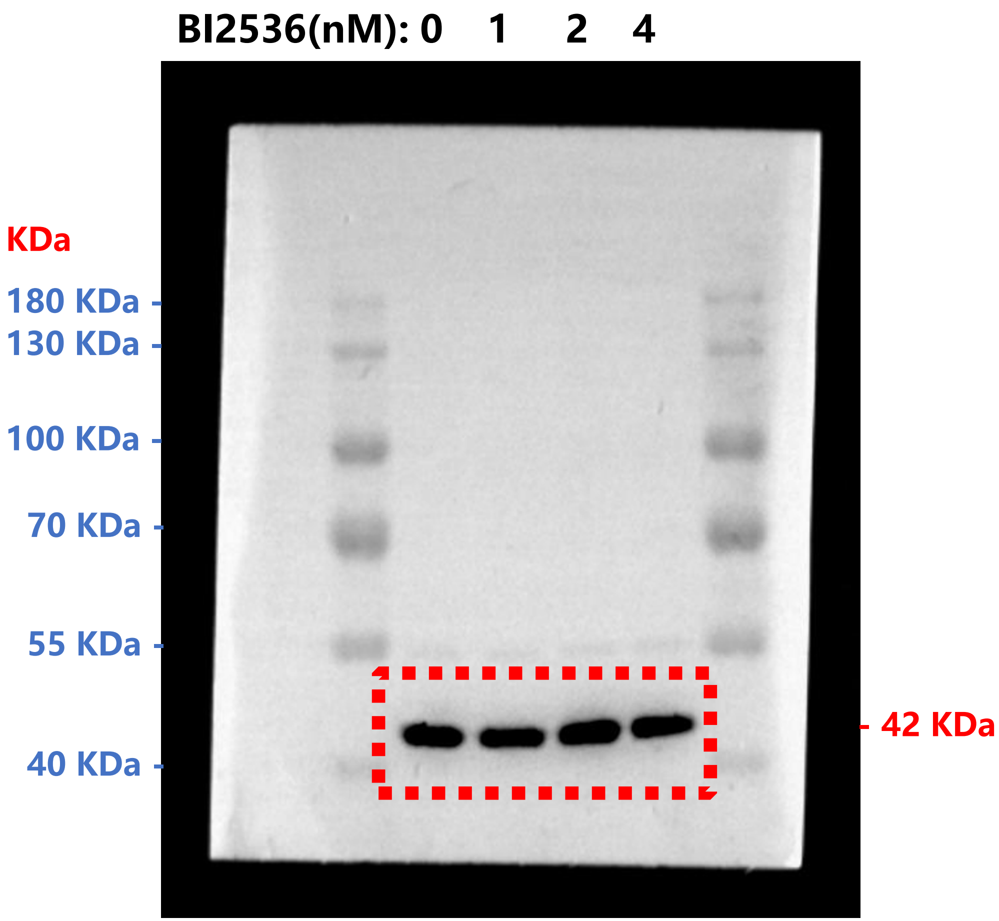

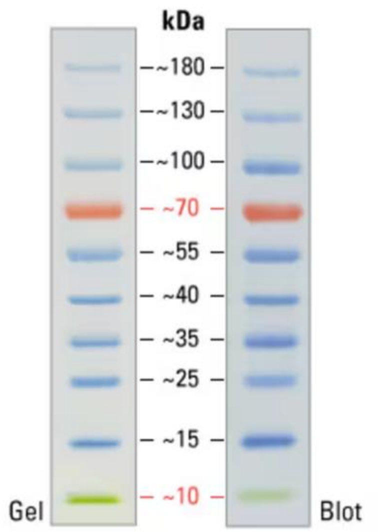

Thermo Scientific™ Marker-26617

■ Original Western Blots of Figure 1-B

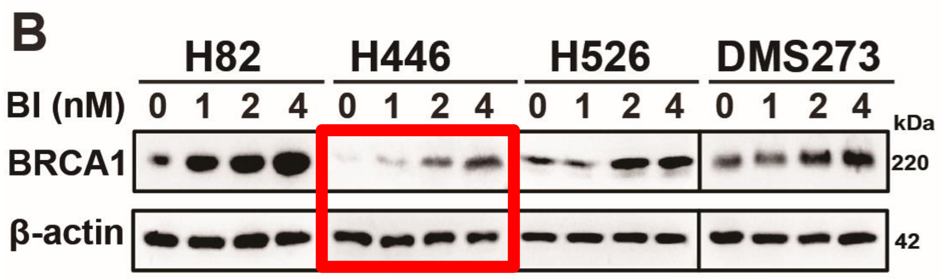

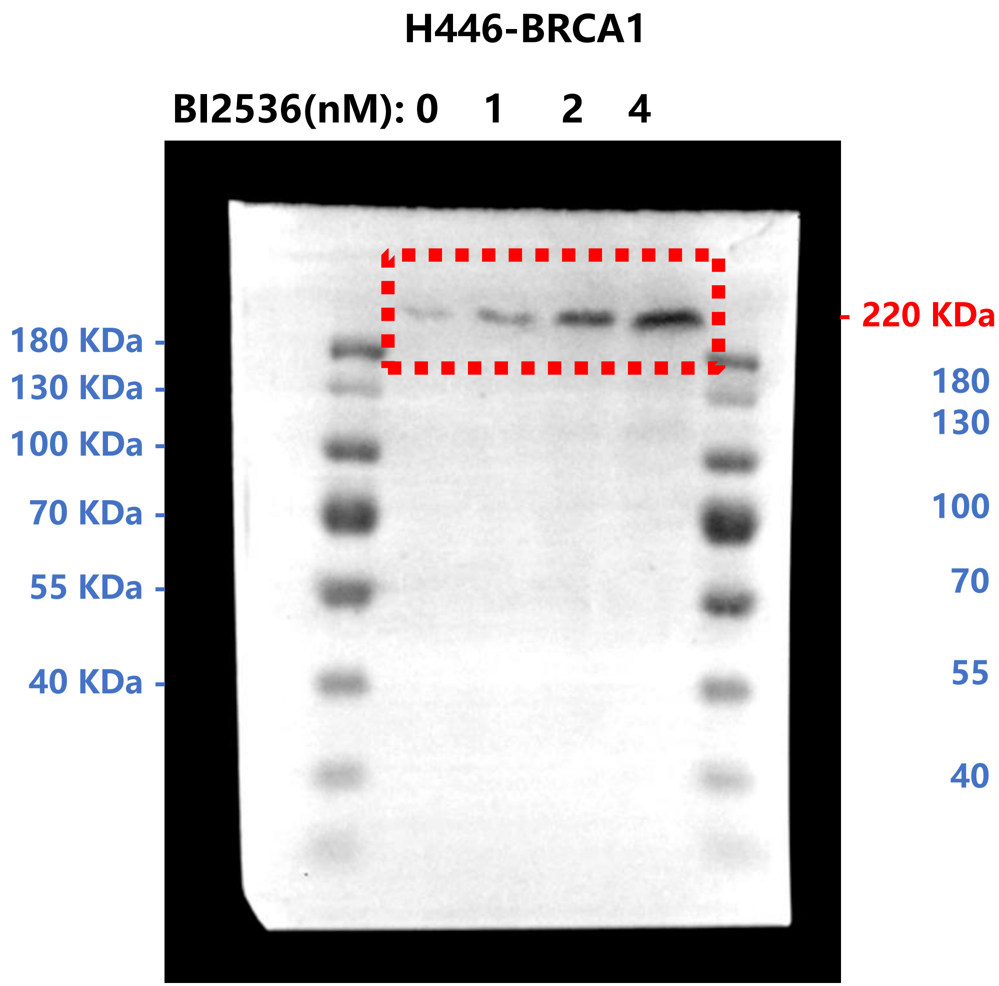

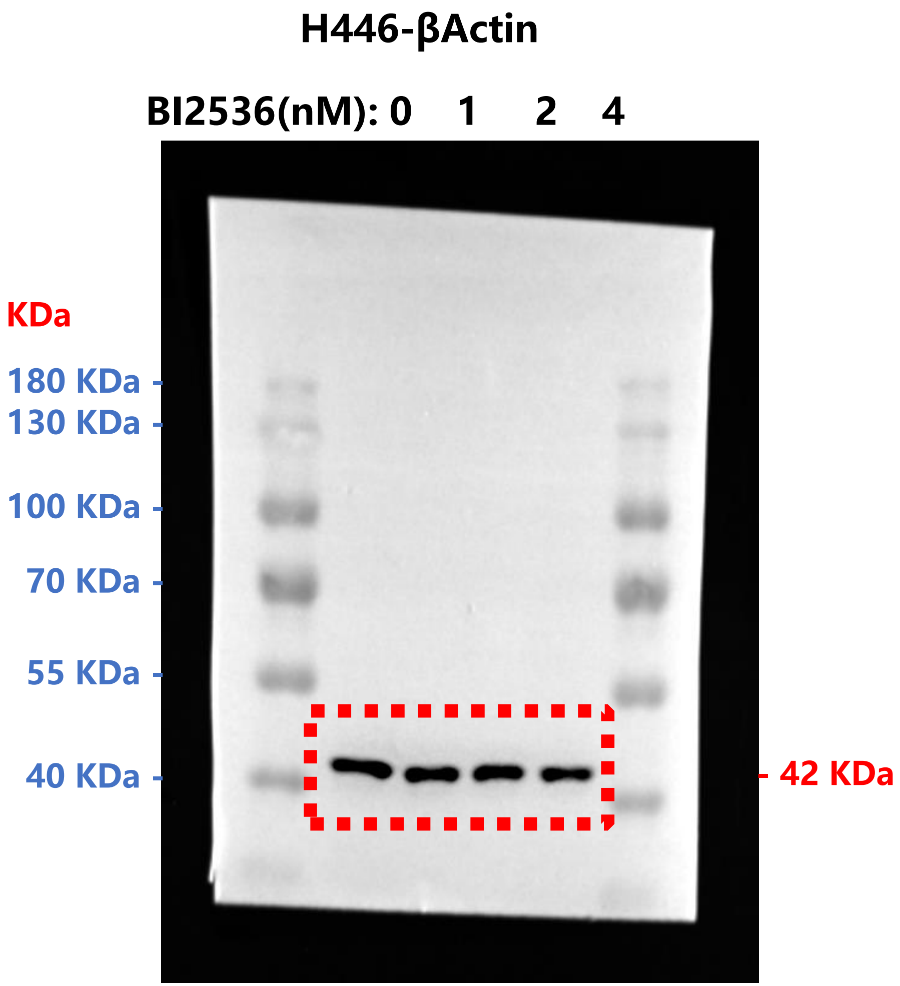

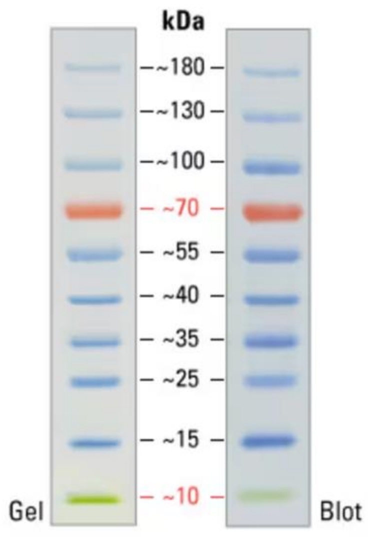

Thermo Scientific™ Marker-26617

■ Original Western Blots of Figure 1-B

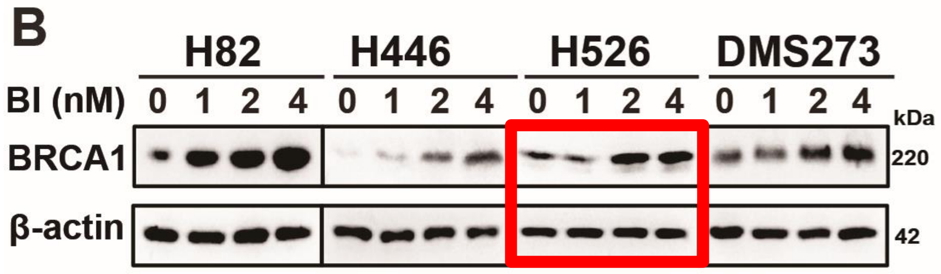

H526-BRCA1

BI2536(nM): 0 1 2 4

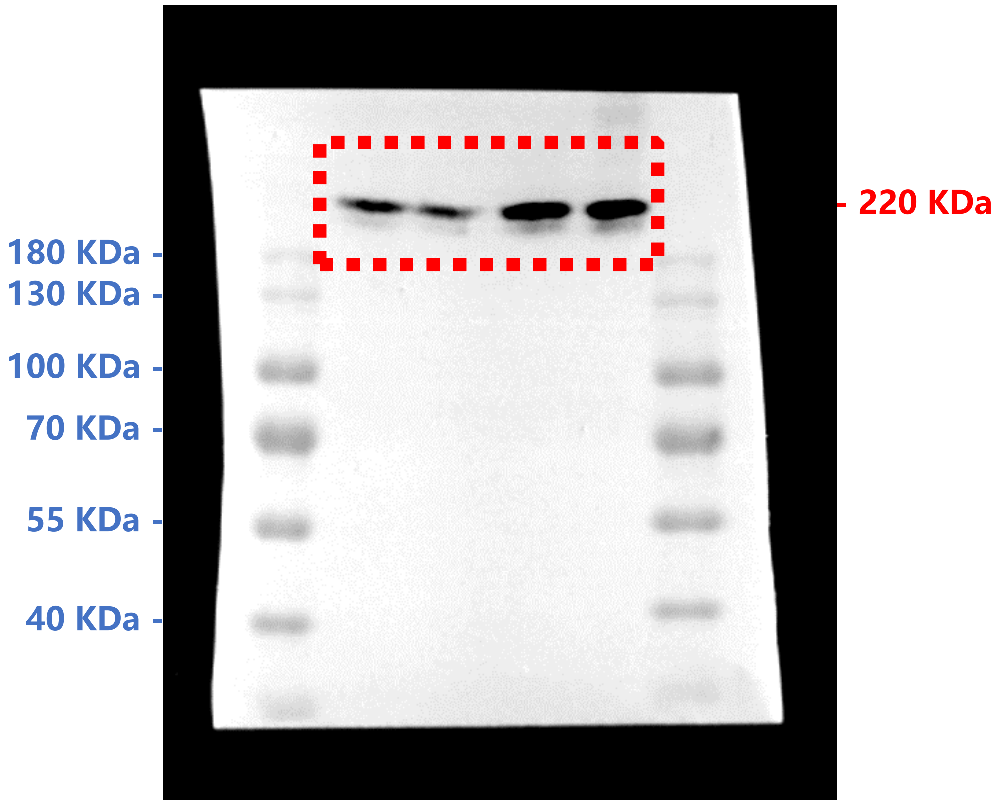

H526-βActin

BI2536(nM): 0 1 2 4

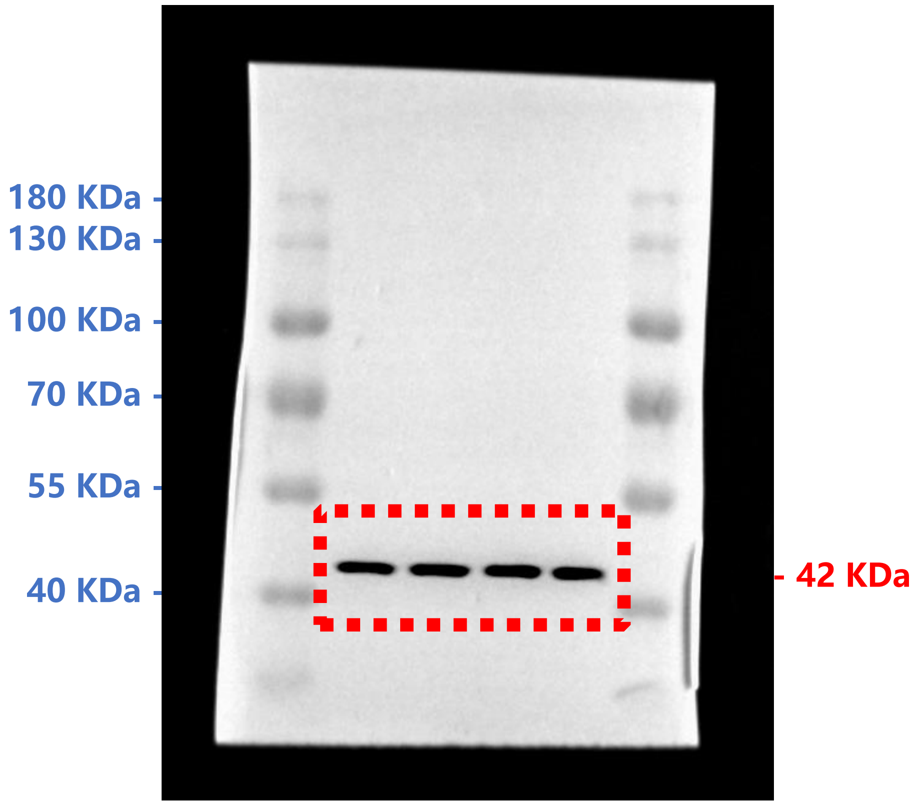

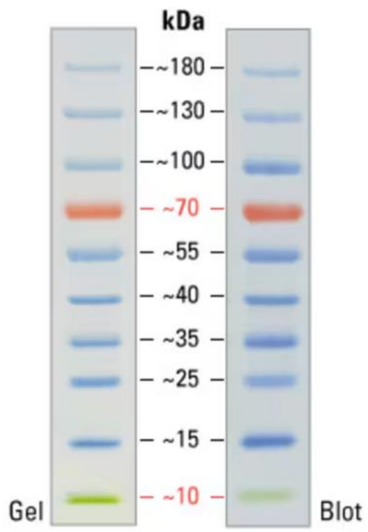

Thermo Scientific™ Marker-26617

■ **Original Western Blots of Figure 1-B**

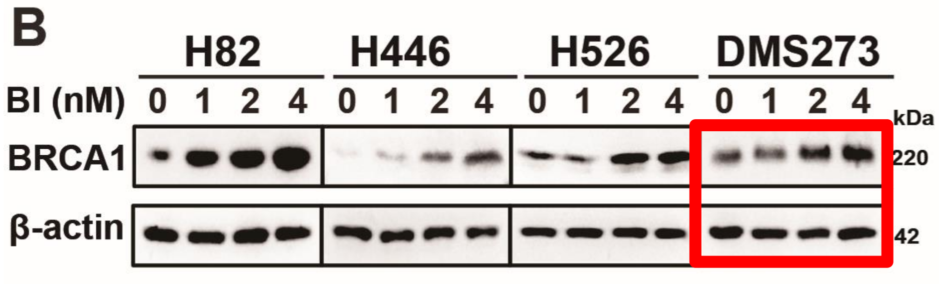

DMS273-BRCA1

BI2536(nM): 0 1 2 4

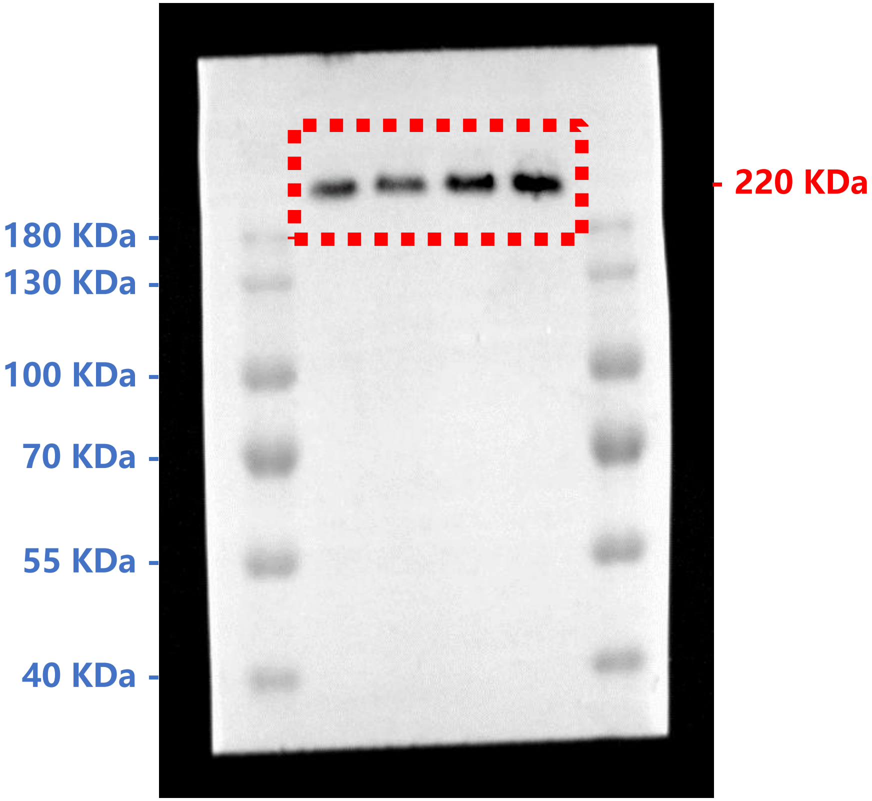

DMS273-βActin

BI2536(nM): 0 1 2 4

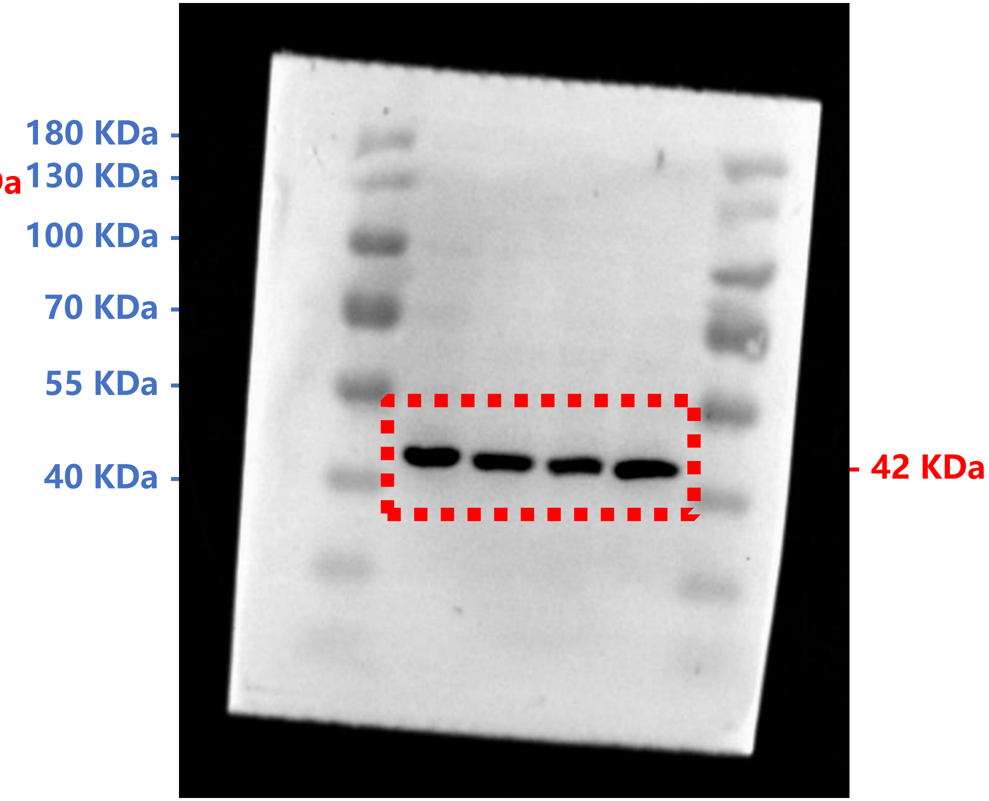

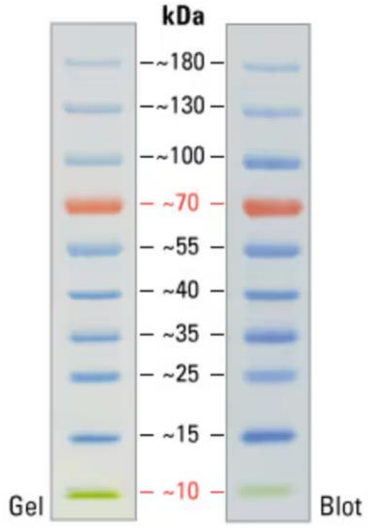

Thermo Scientific™ Marker-26617

■ Original Western Blots of Figure 1-C

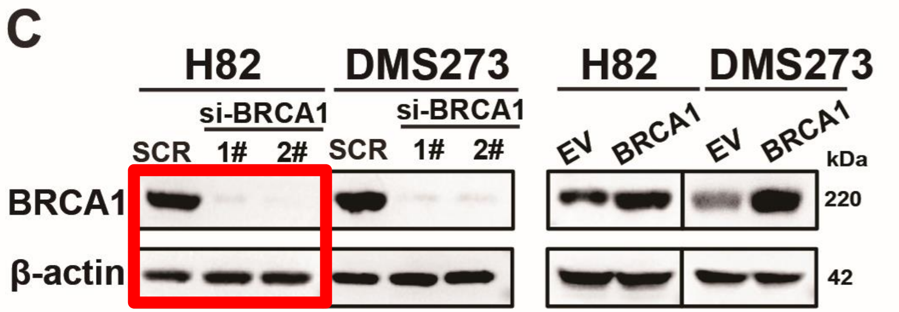

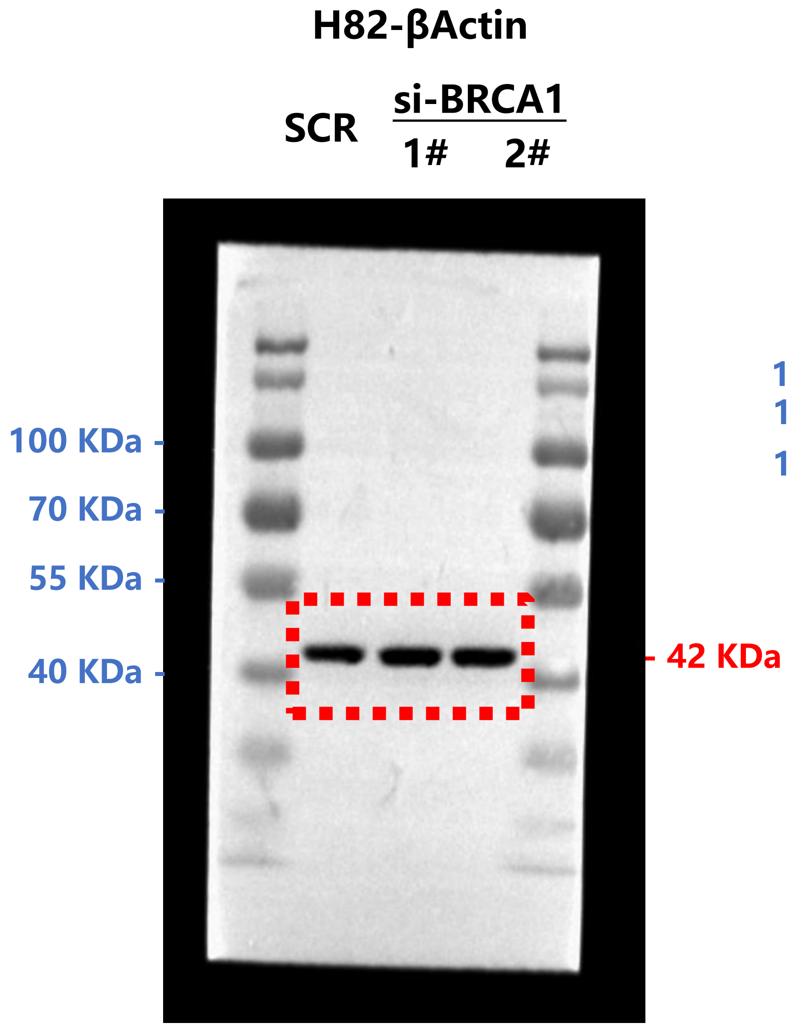

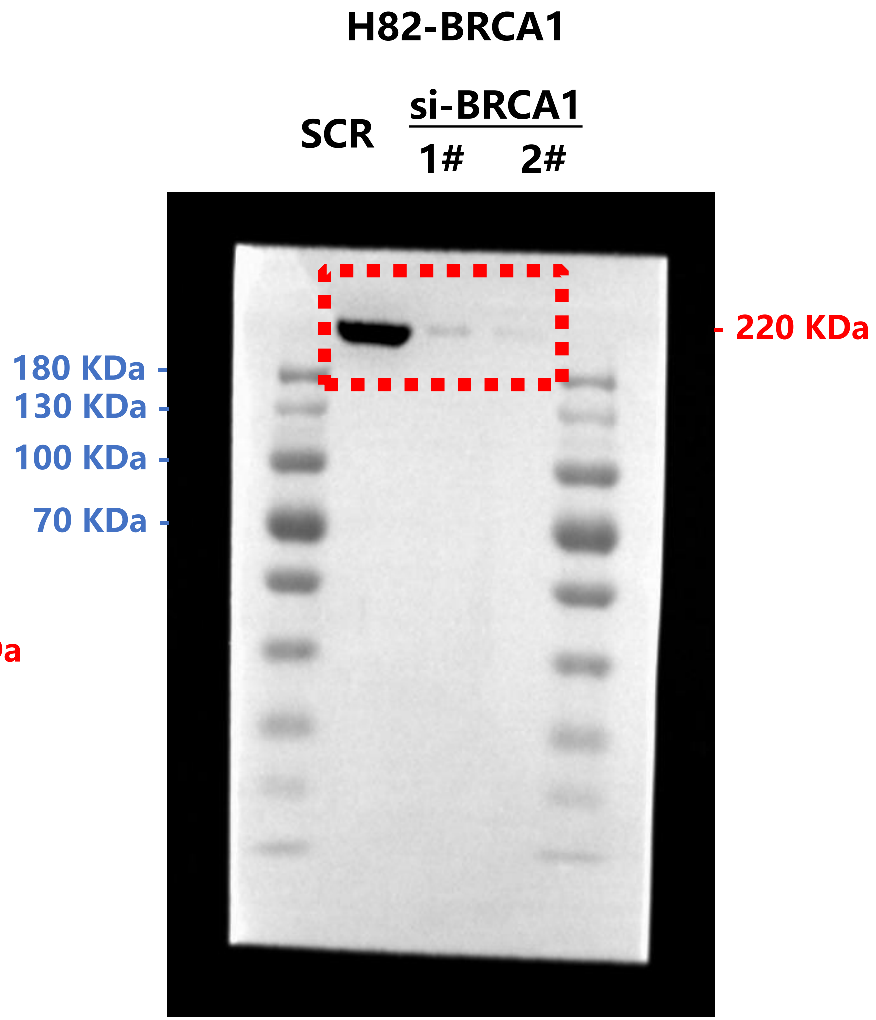

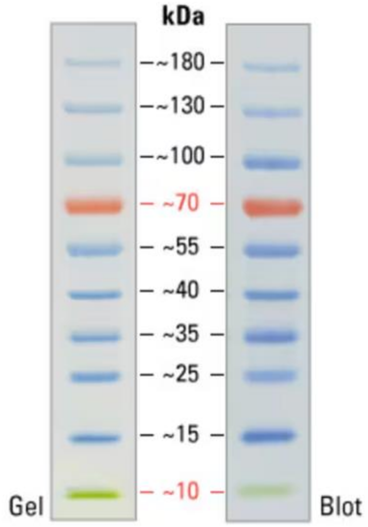

Thermo Scientific™ Marker-26617

■ Original Western Blots of Figure 1-C

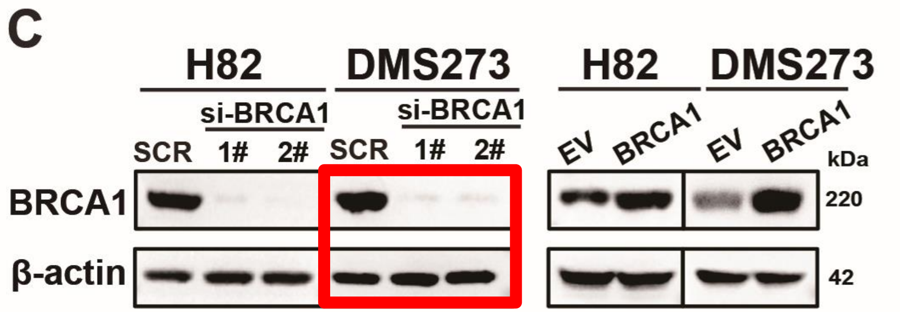

DMS273-βActin

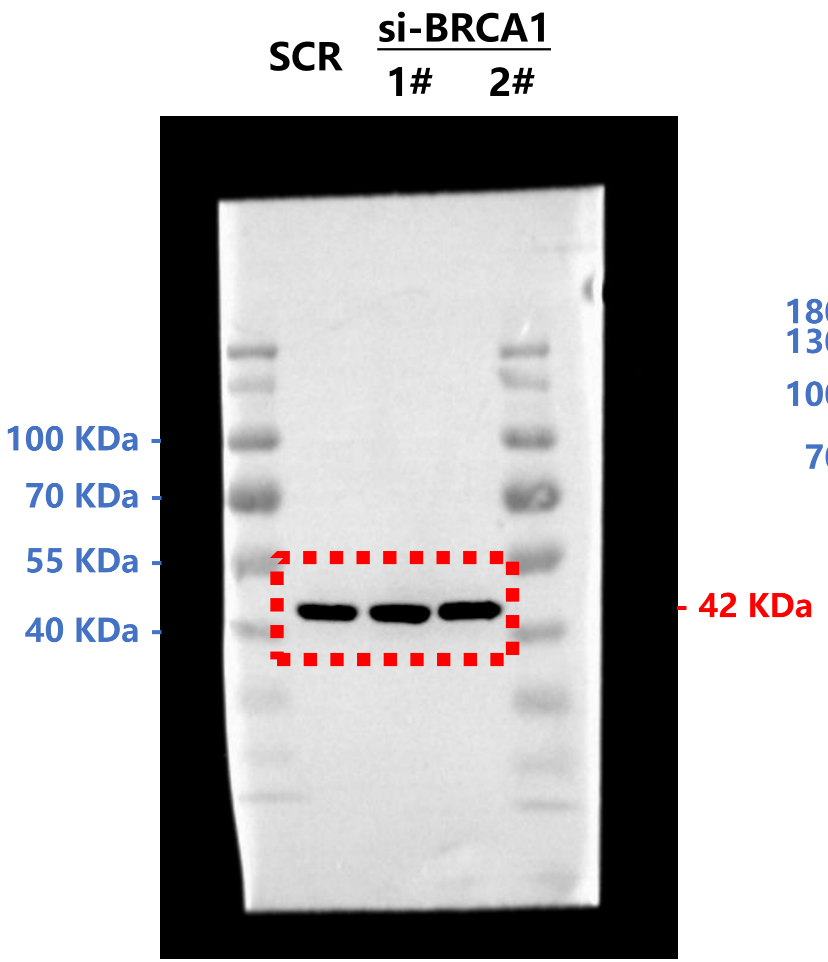

DMS273-BRCA1

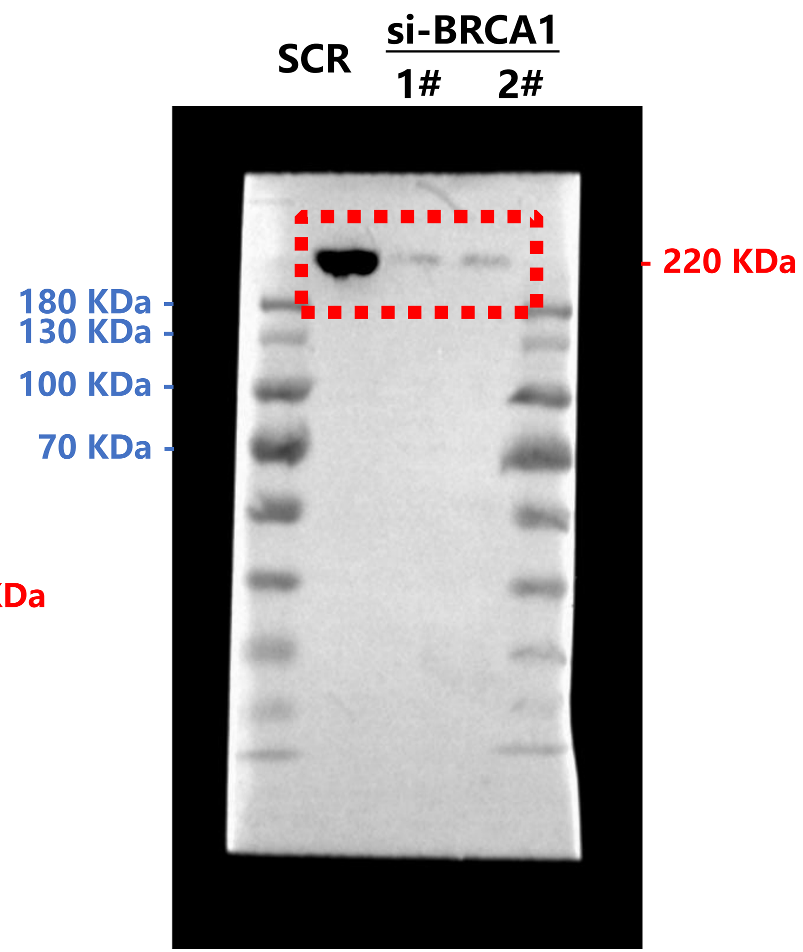

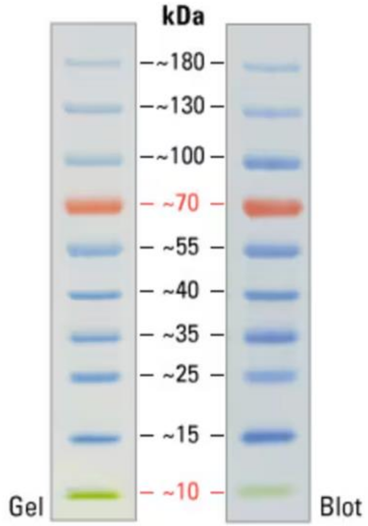

Thermo Scientific™ Marker-26617

■ Original Western Blots of Figure 1-C

C

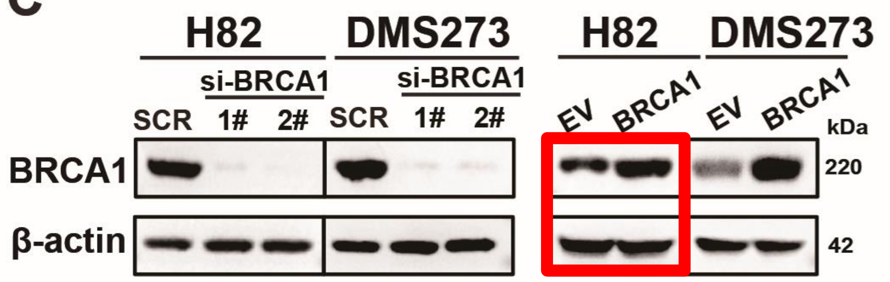

H82-βActin

EV BRCA1

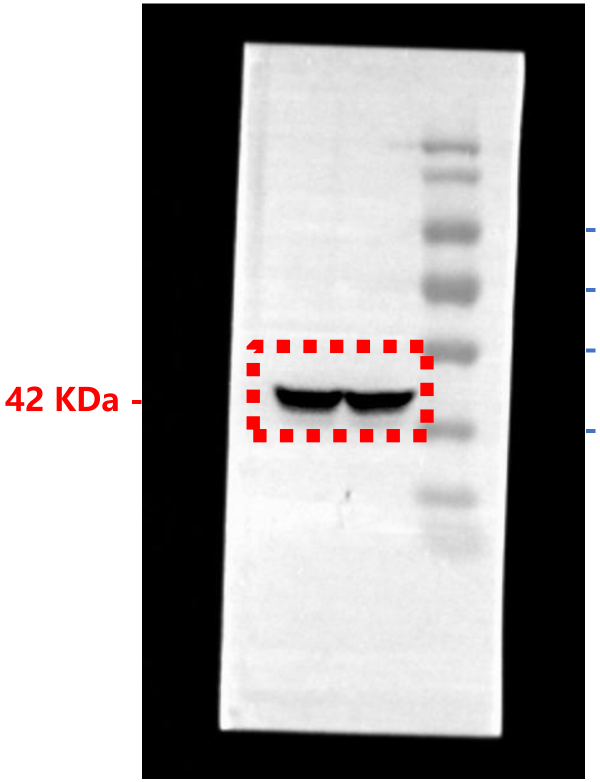

42 KDa -

- 100 KDa
- 70 KDa
- 55 KDa
- 40 KDa

H82-BRCA1

EV BRCA1

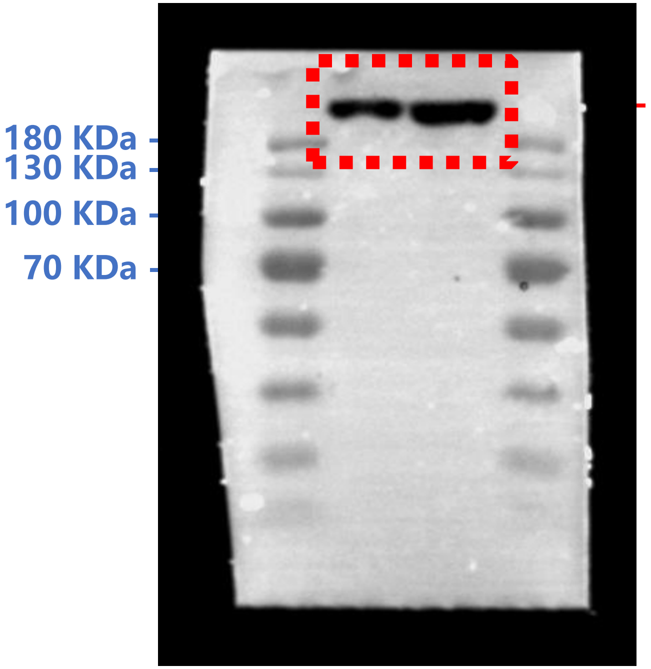

180 KDa -
130 KDa -
100 KDa -
70 KDa -

- 220 KDa

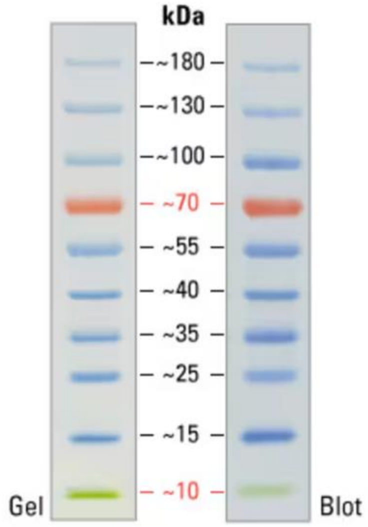

Thermo Scientific™ Marker-26617

■ Original Western Blots of Figure 1-C

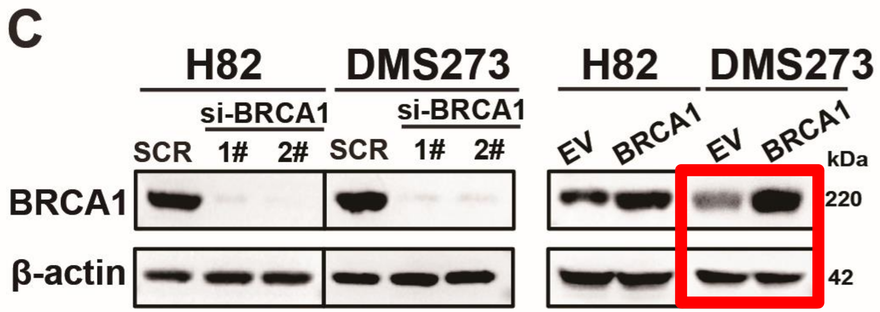

DMS273-βActin

EV BRCA1

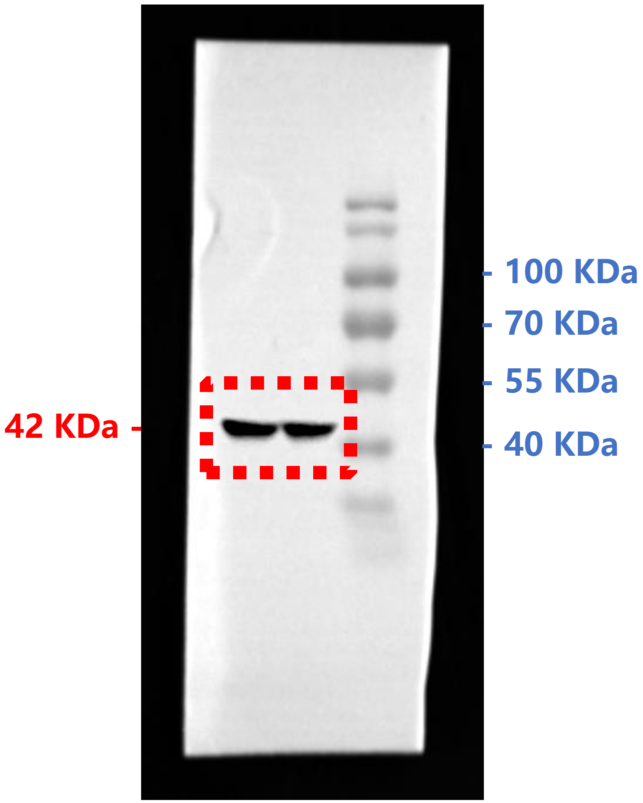

DMS273-BRCA1

EV BRCA1

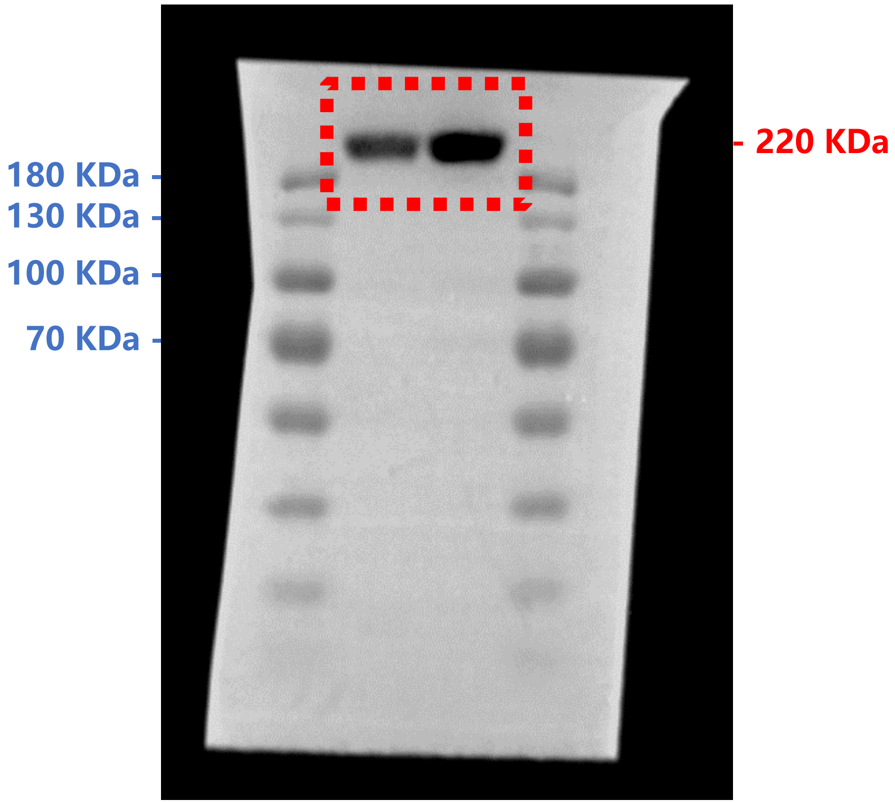

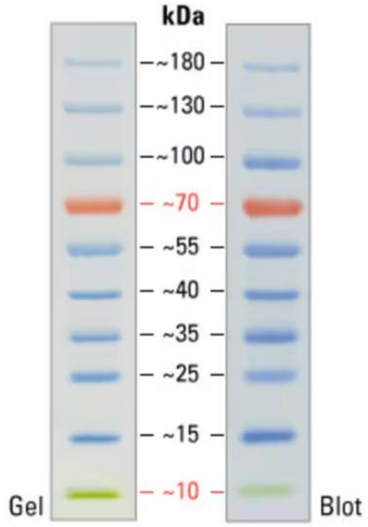

Thermo Scientific™ Marker-26617

■ Original Western Blots of Figure 2-C

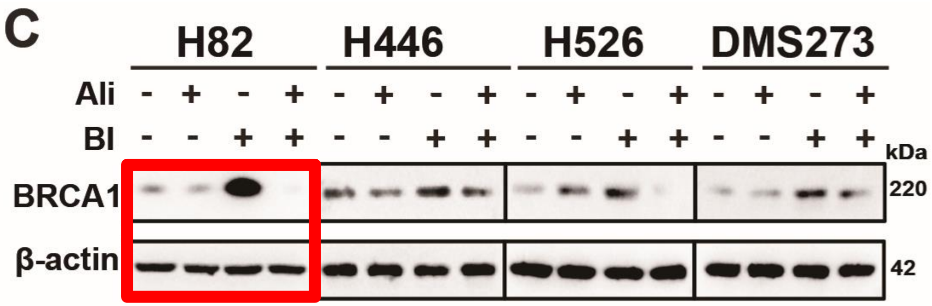

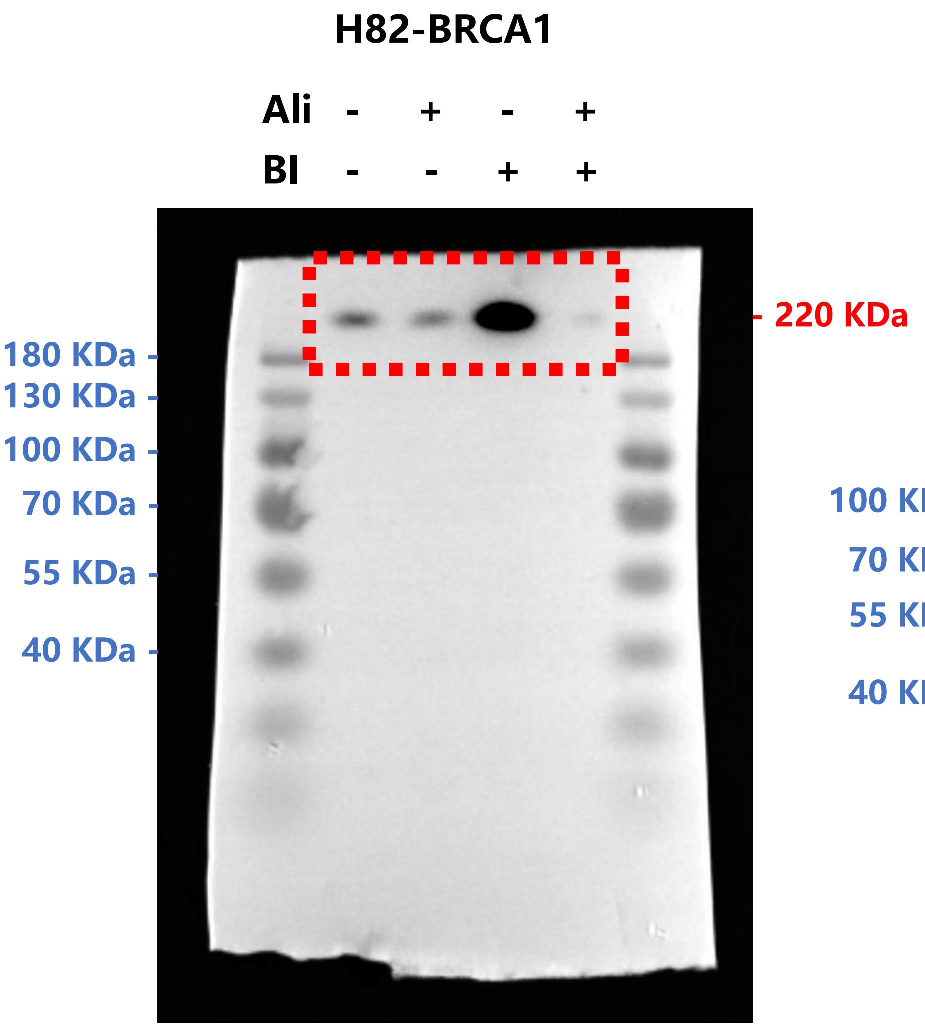

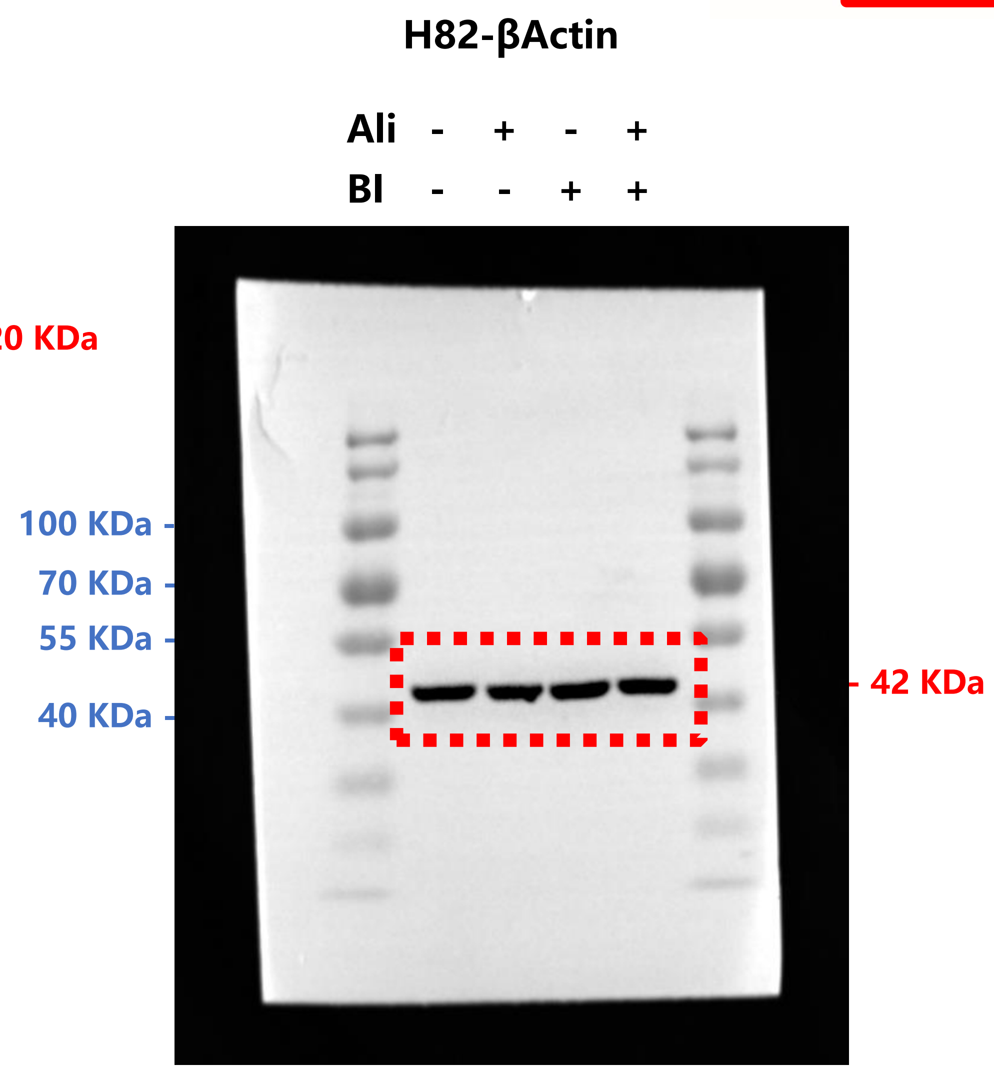

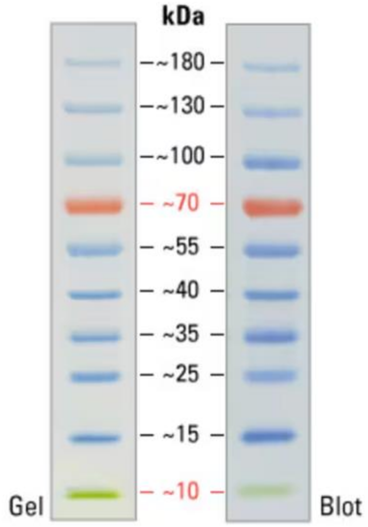

Thermo Scientific™ Marker-26617

■ Original Western Blots of Figure 2-C

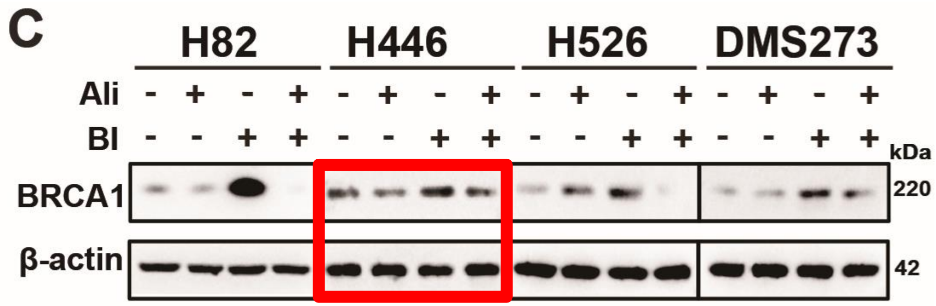

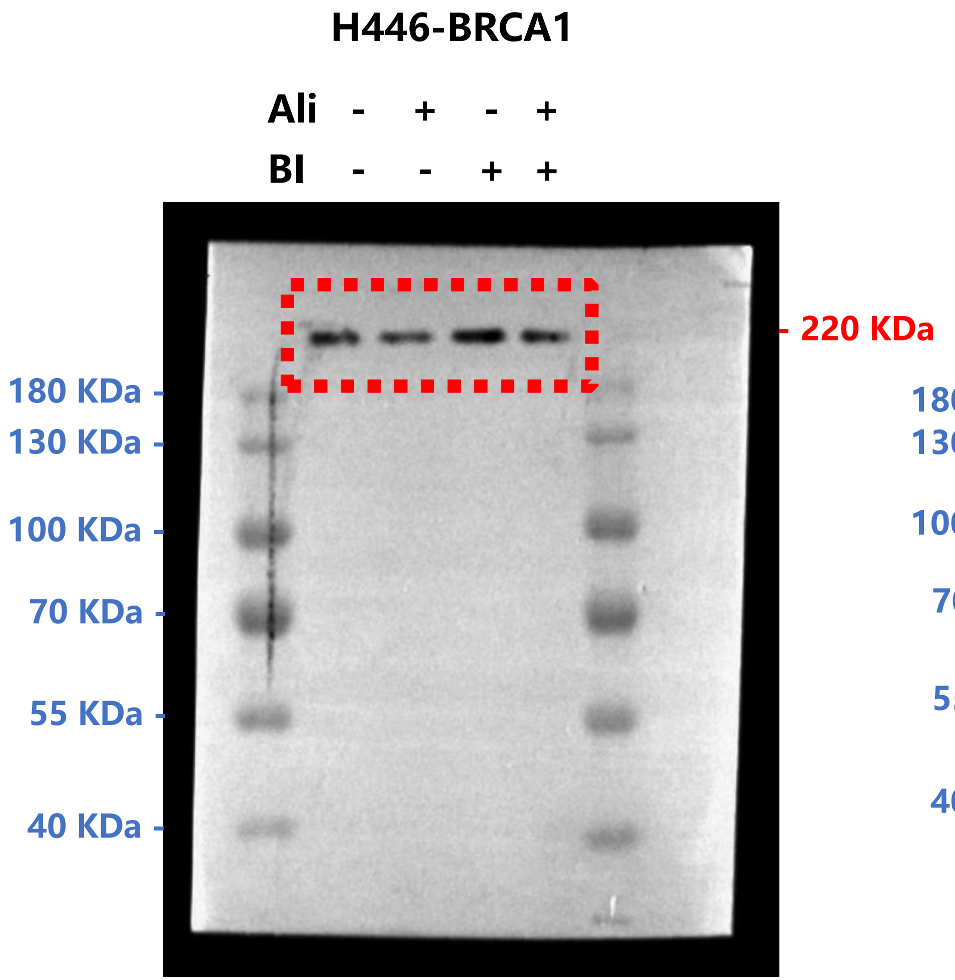

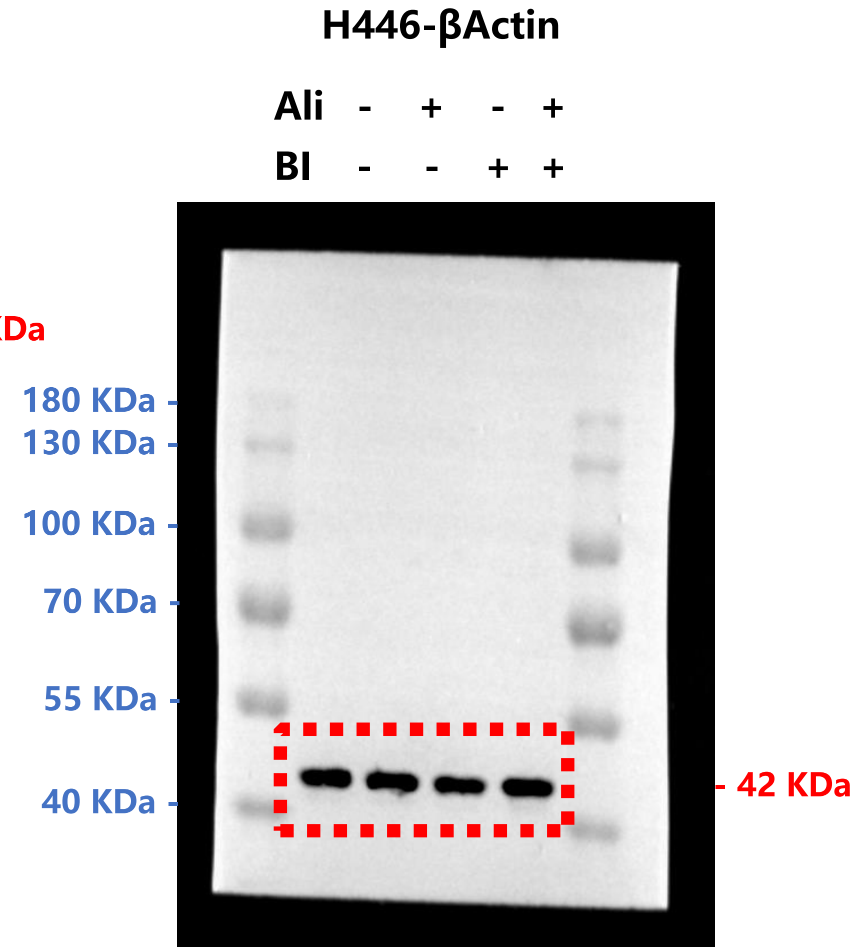

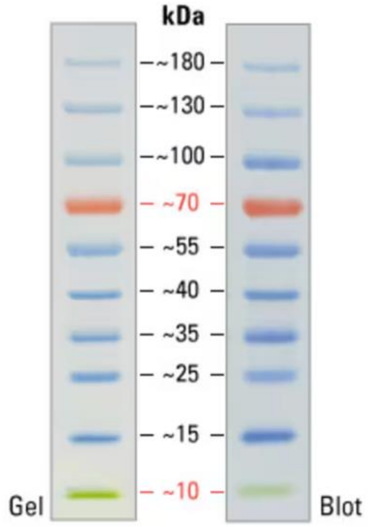

Thermo Scientific™ Marker-26617

■ Original Western Blots of Figure 2-C

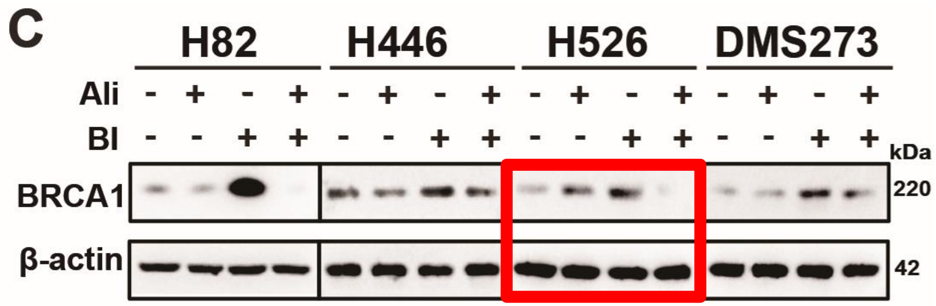

H526-BRCA1

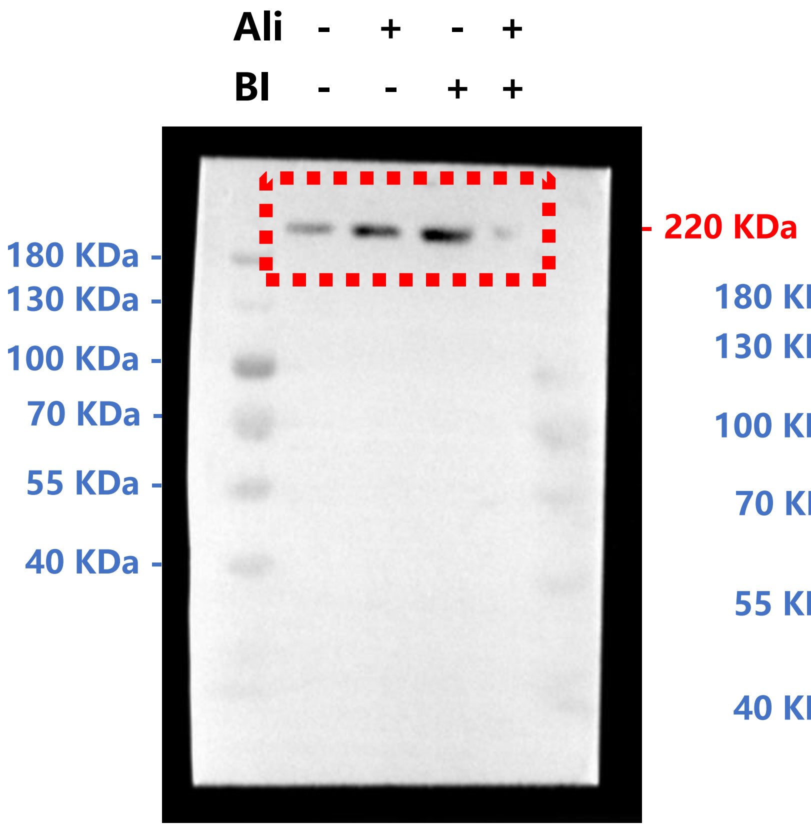

H526-βActin

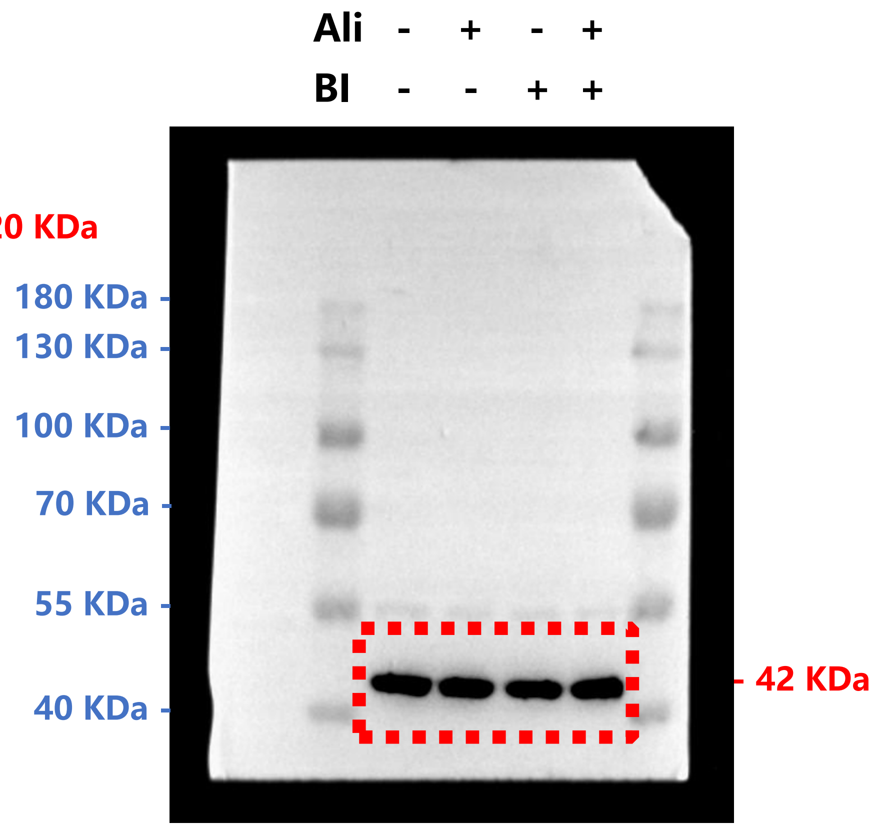

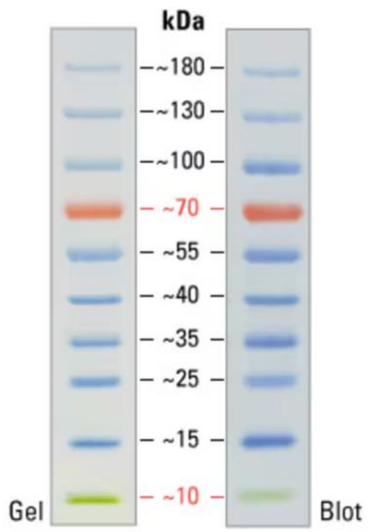

Thermo Scientific™ Marker-26617

■ Original Western Blots of Figure 2-C

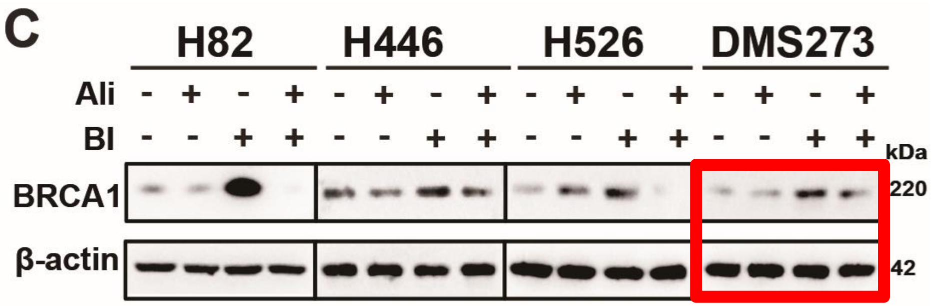

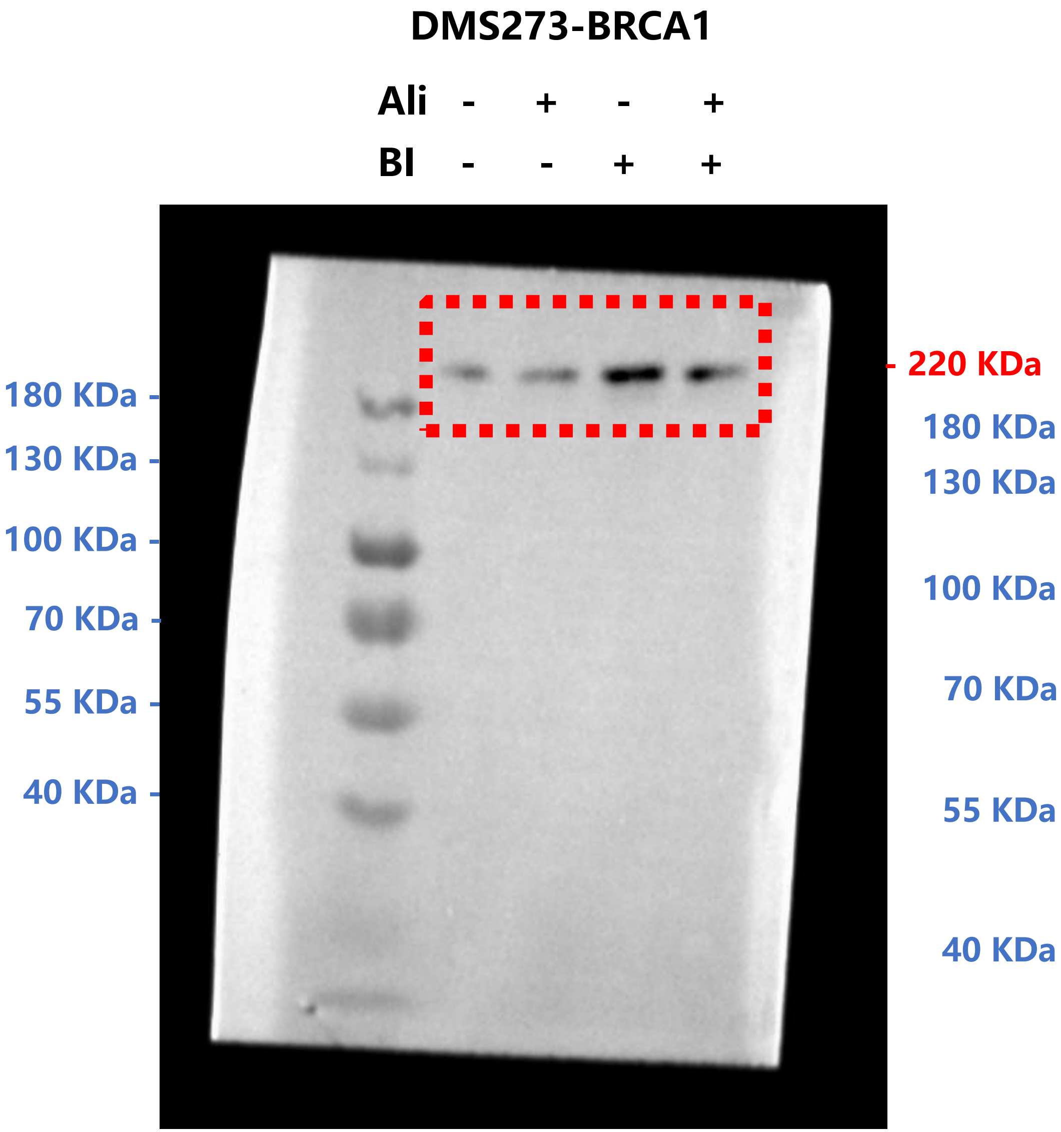

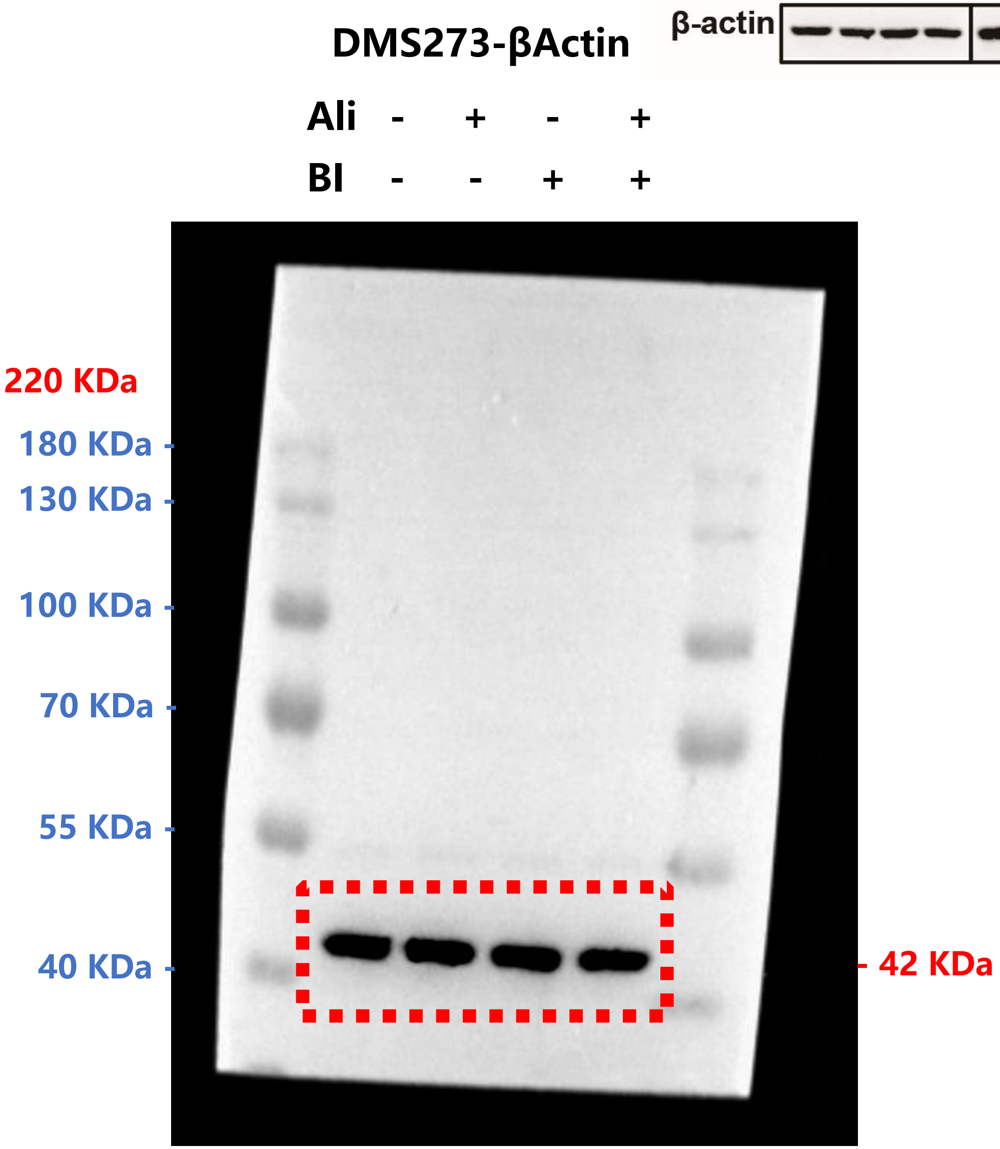

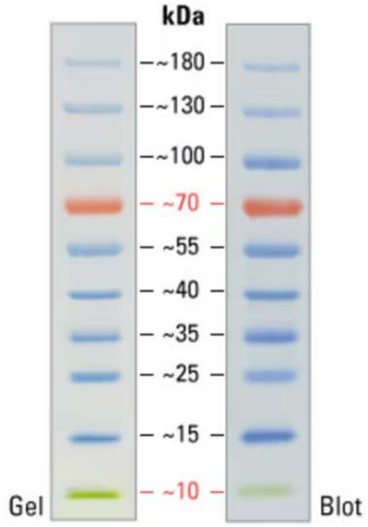

Thermo Scientific™ Marker-26617

■ Original Western Blots of Figure 2-D

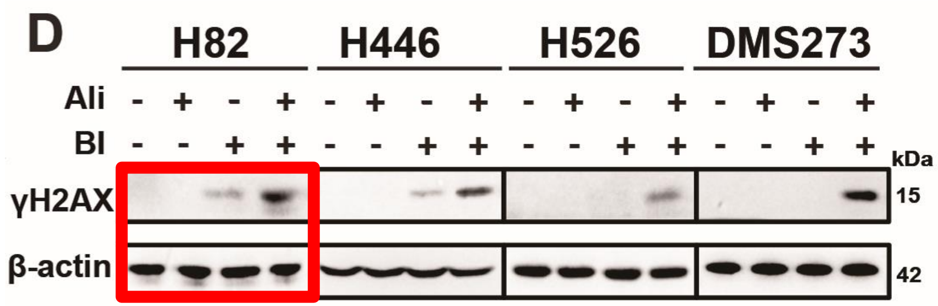

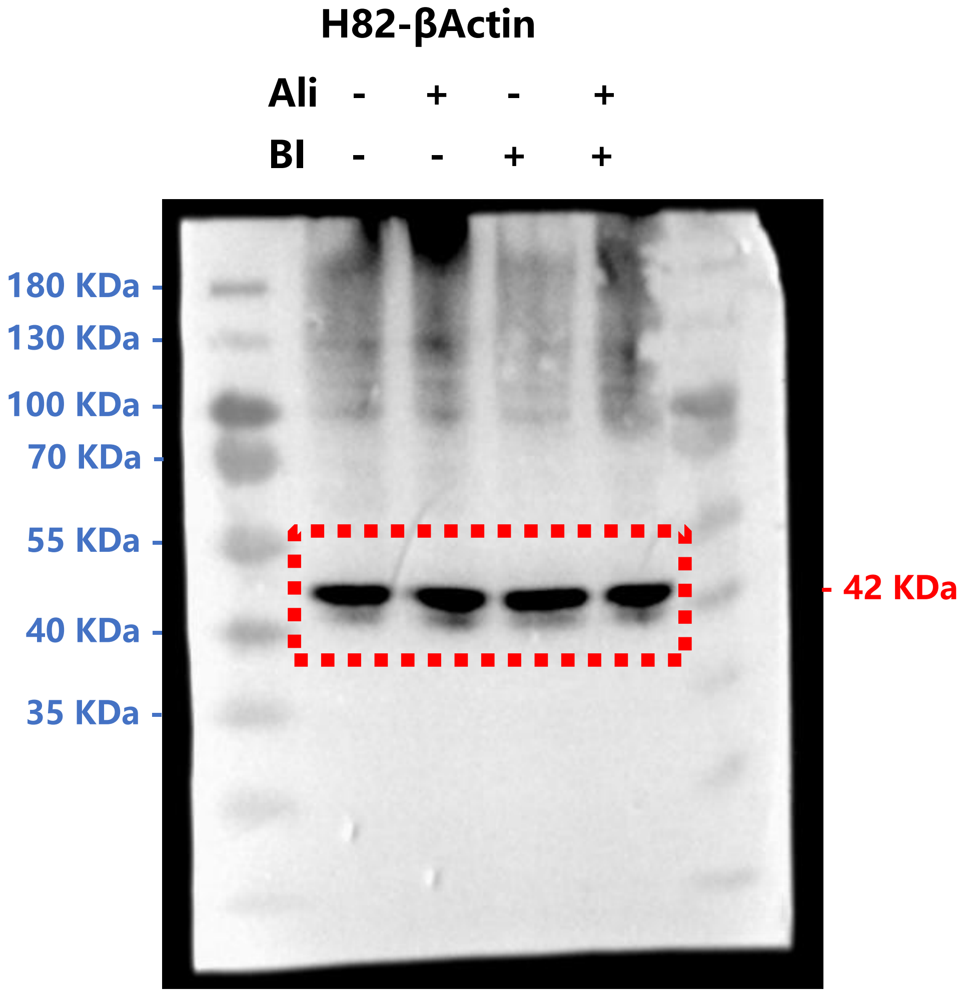

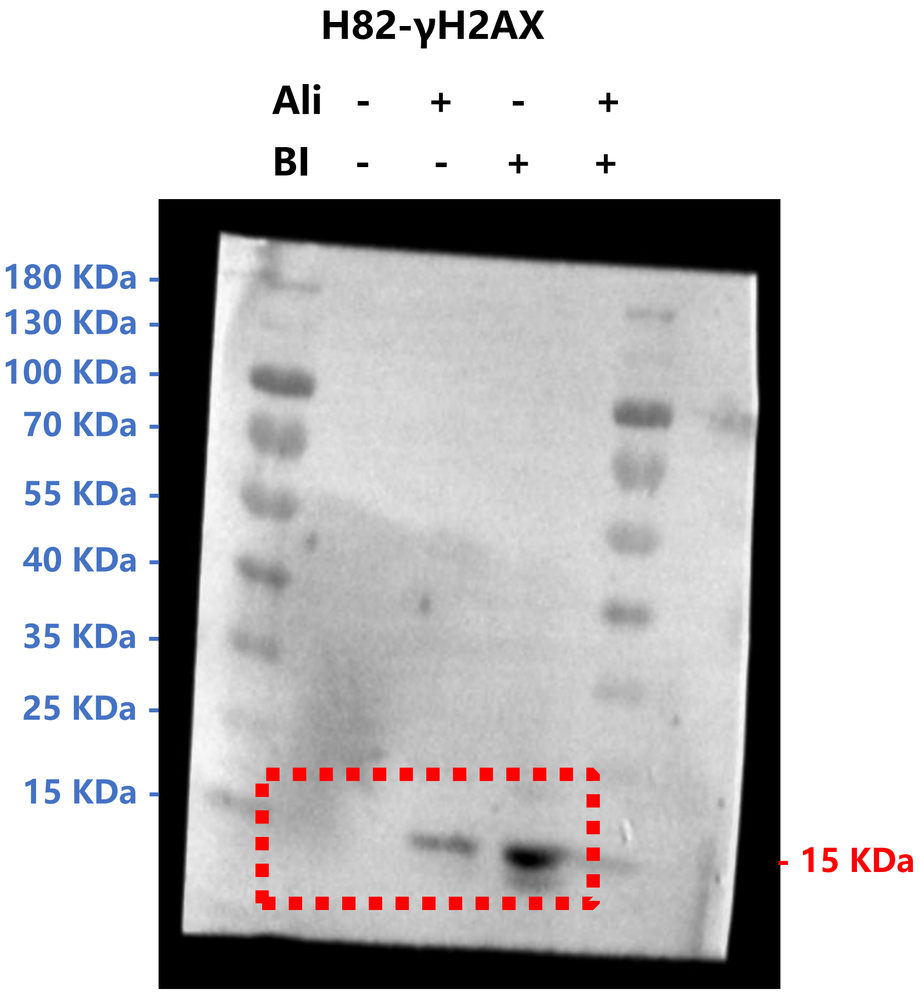

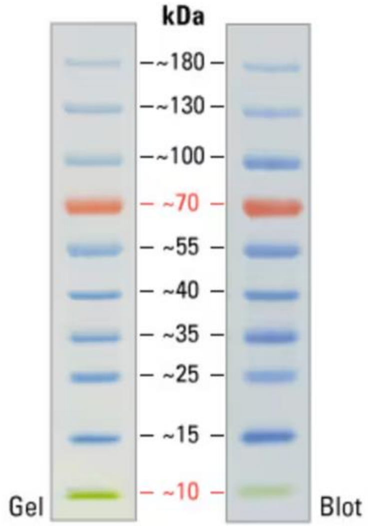

Thermo Scientific™ Marker-26617

■ Original Western Blots of Figure 2-D

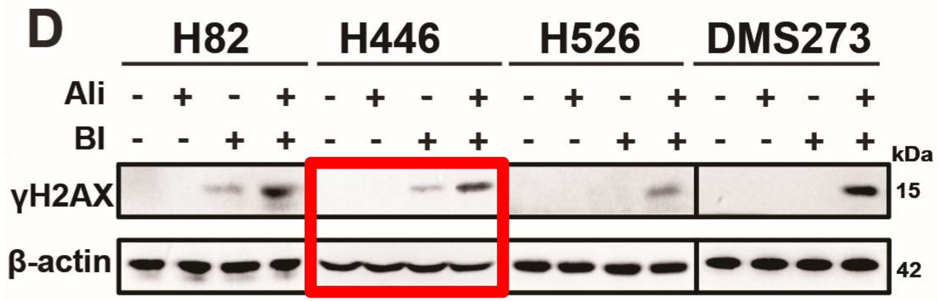

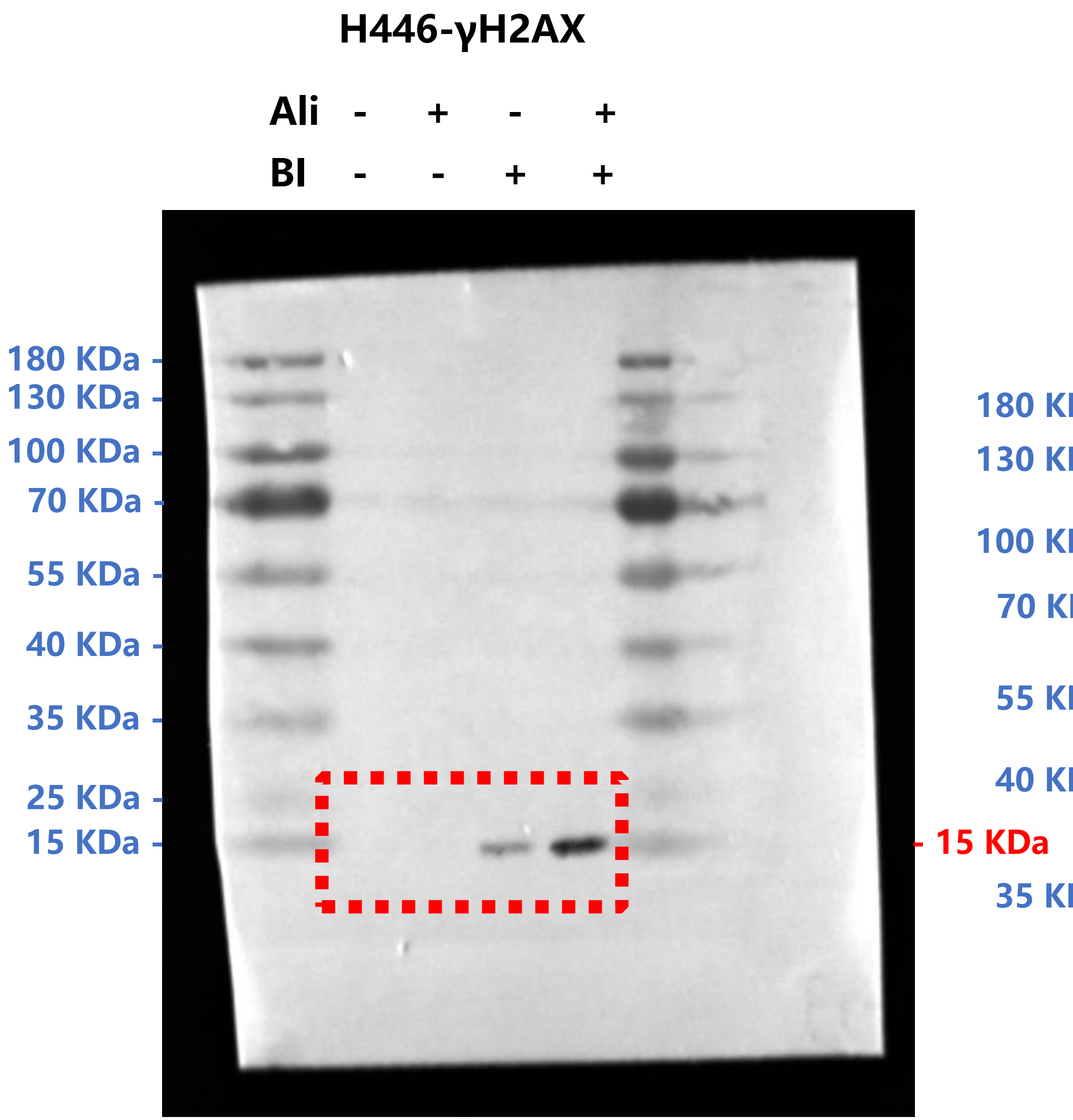

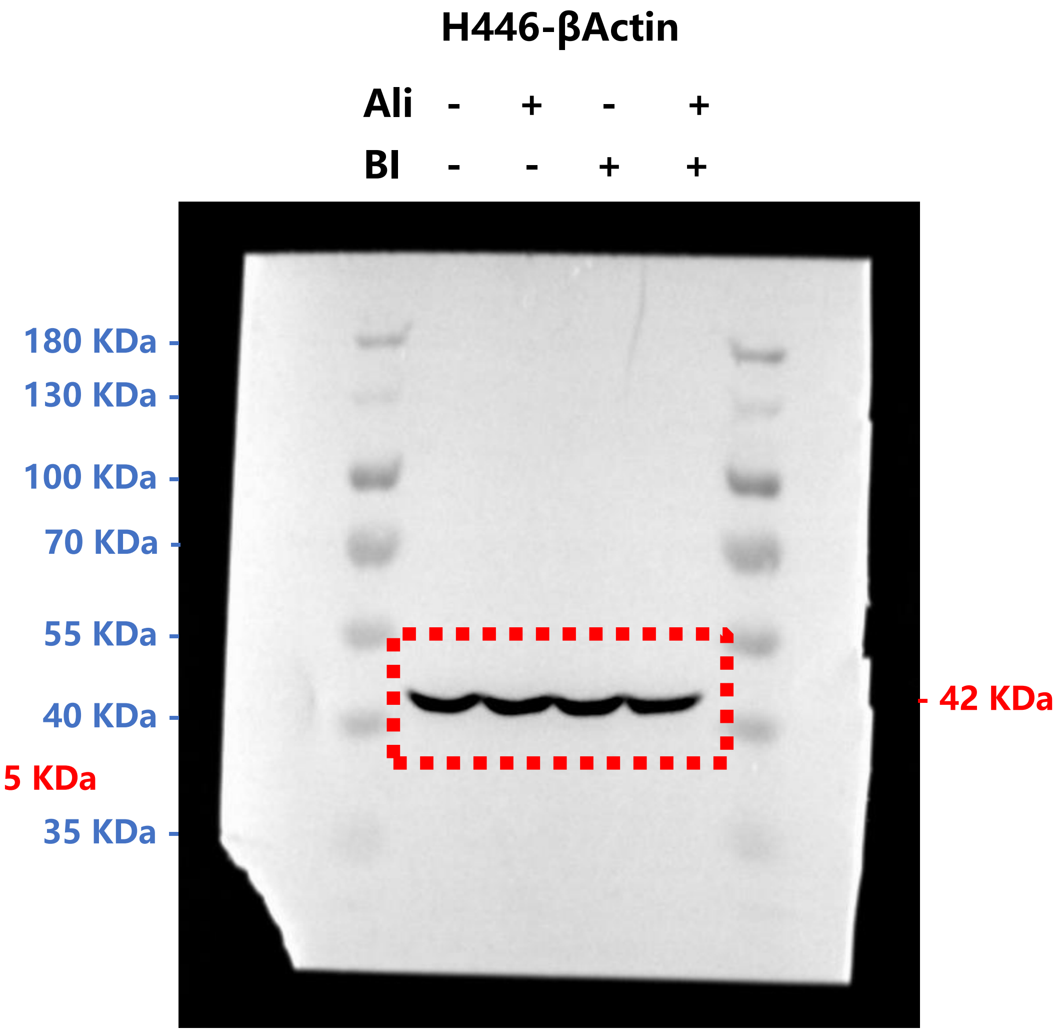

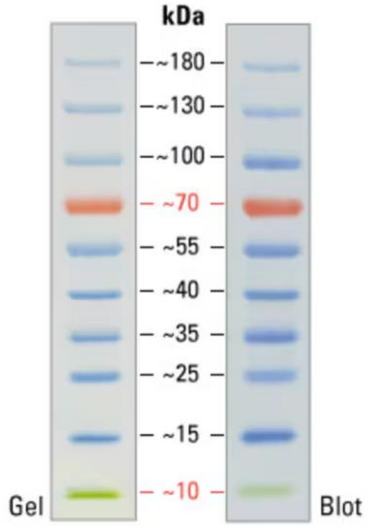

Thermo Scientific™ Marker-26617

■ **Original Western Blots of Figure 2-D**

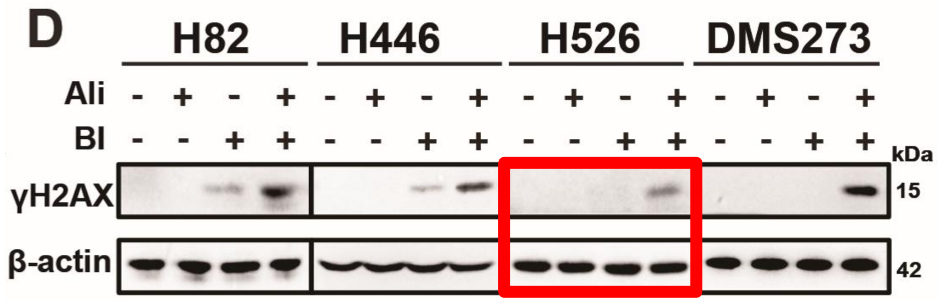

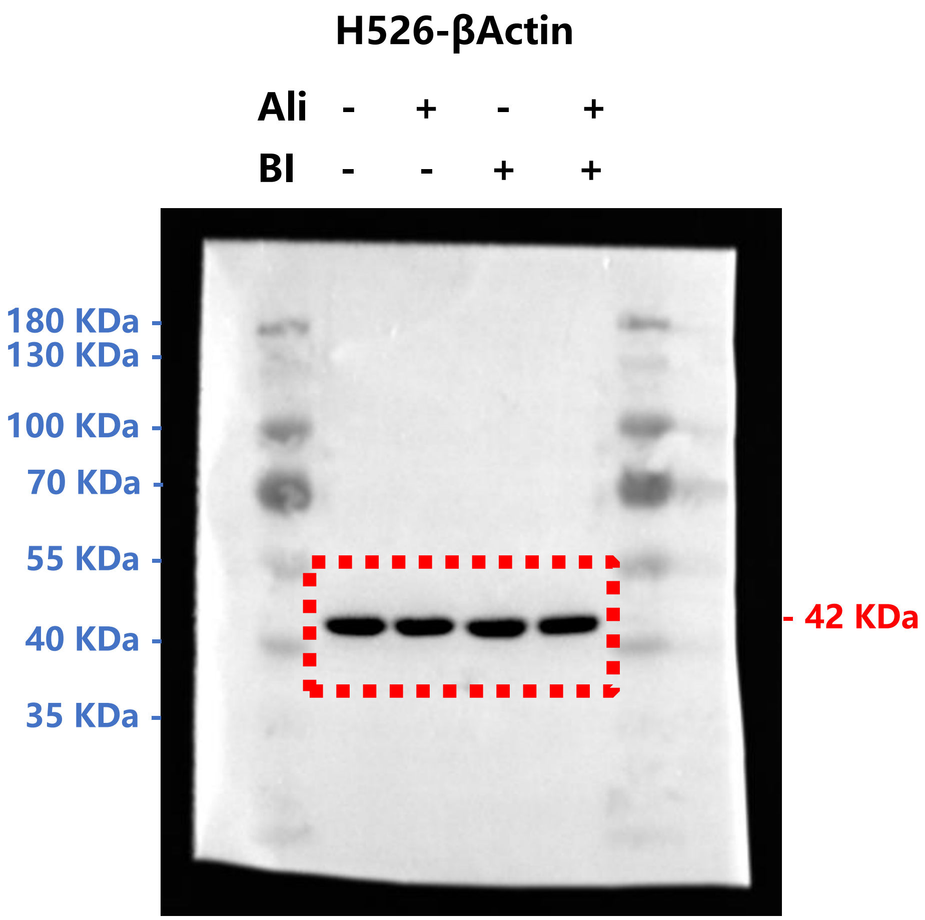

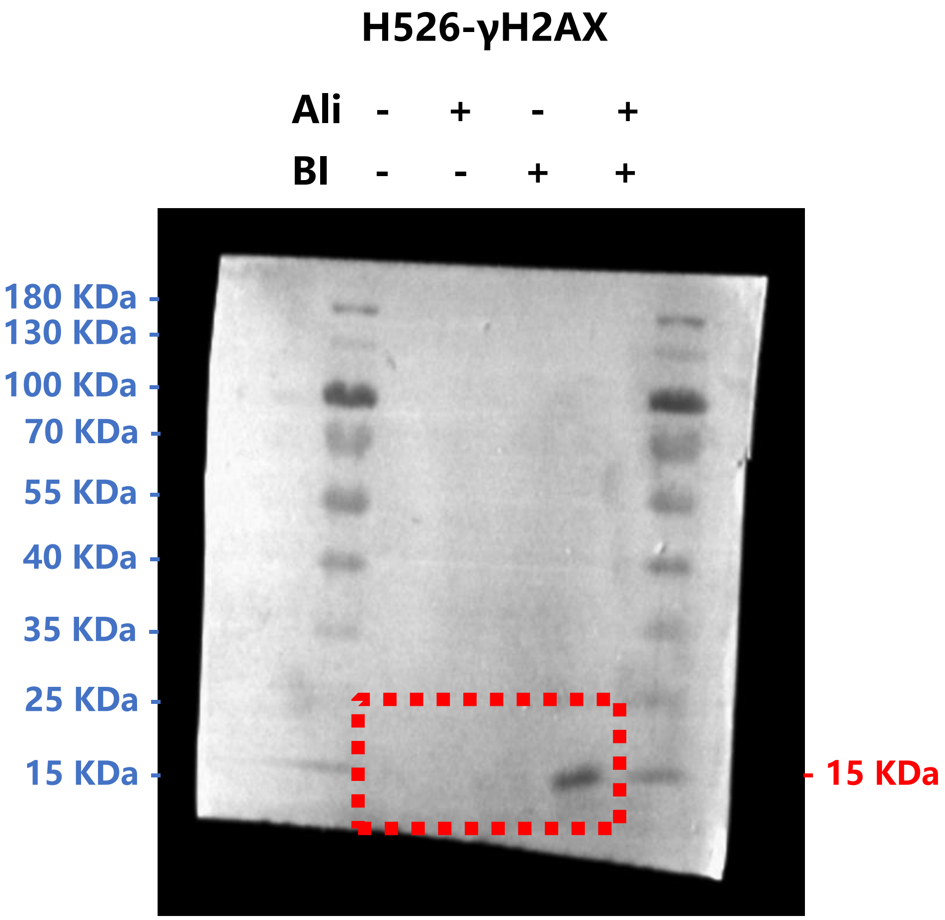

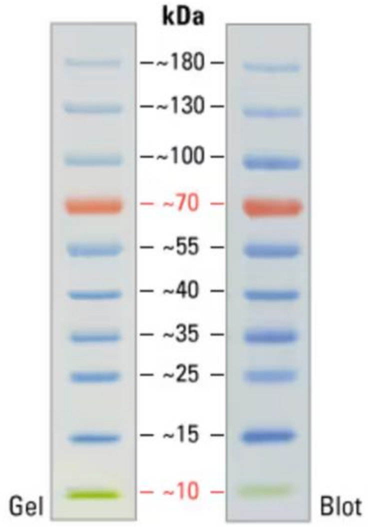

Thermo Scientific™ Marker-26617

■ **Original Western Blots of Figure 2-D**

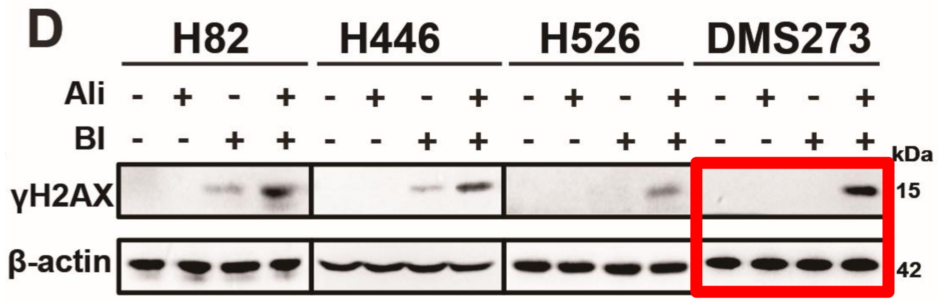

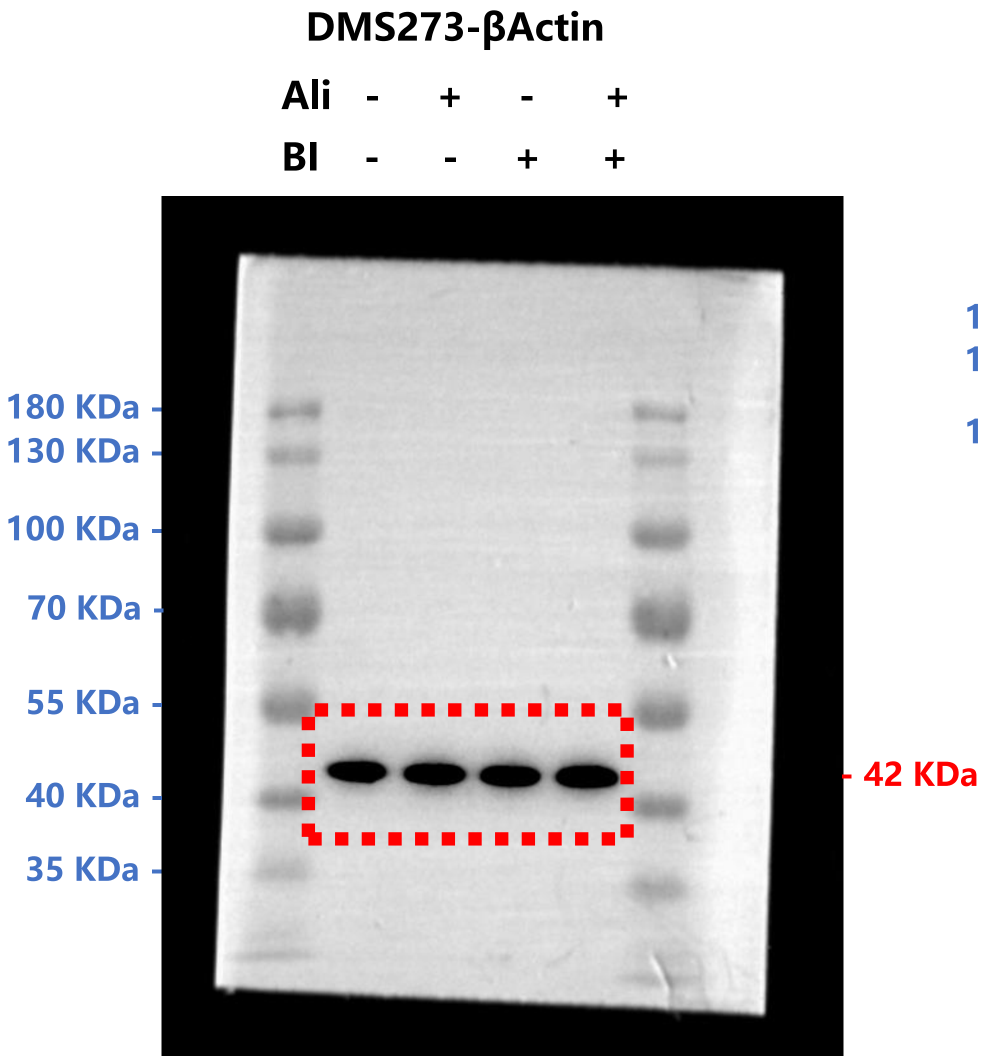

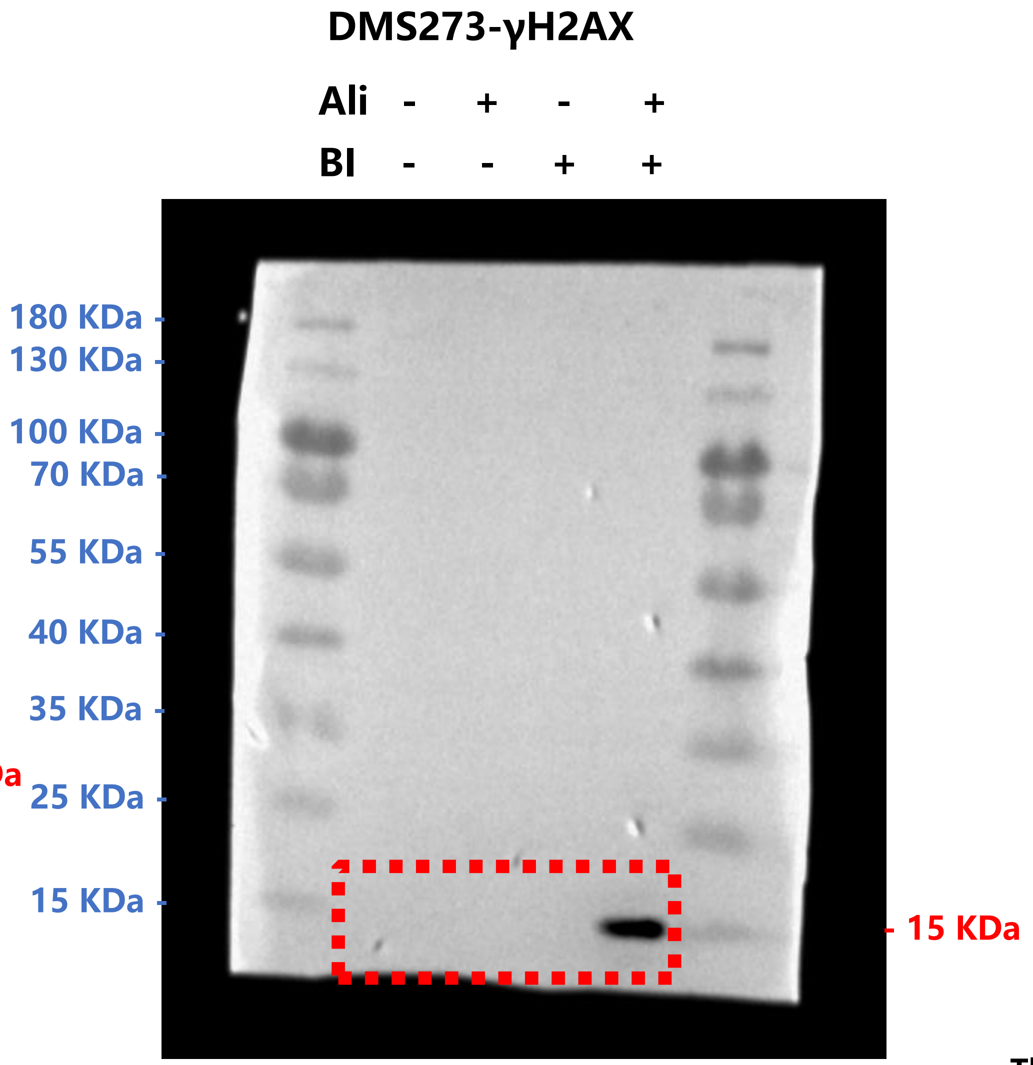

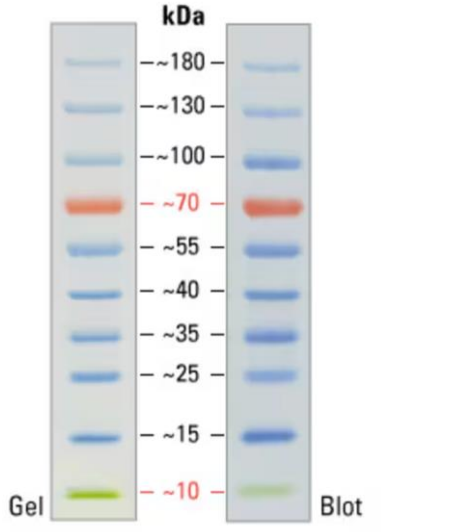

Thermo Scientific™ Marker-26617

■ Original Western Blots of Figure 3-B-14h

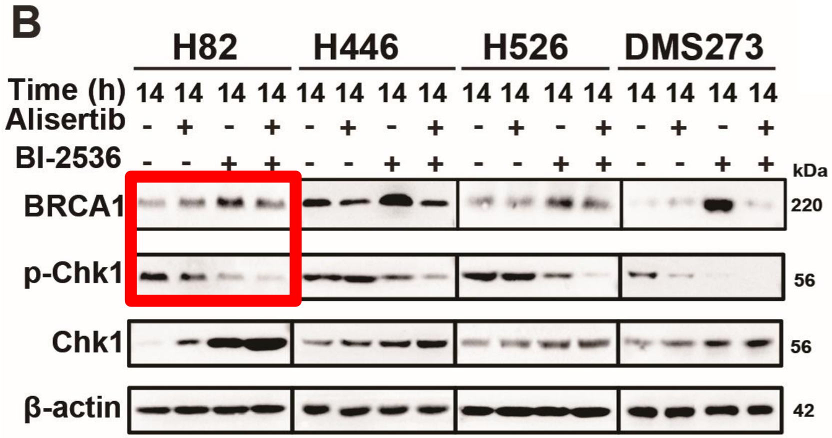

H82-BRCA1-14h

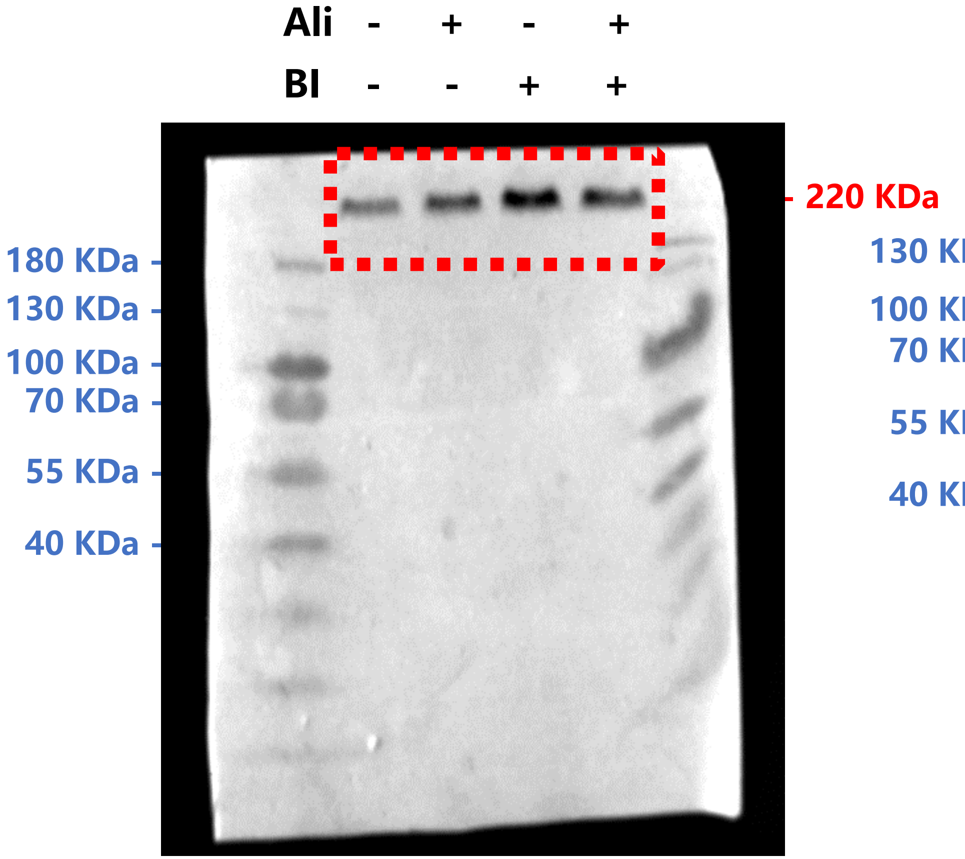

H82-pCHK1-14h

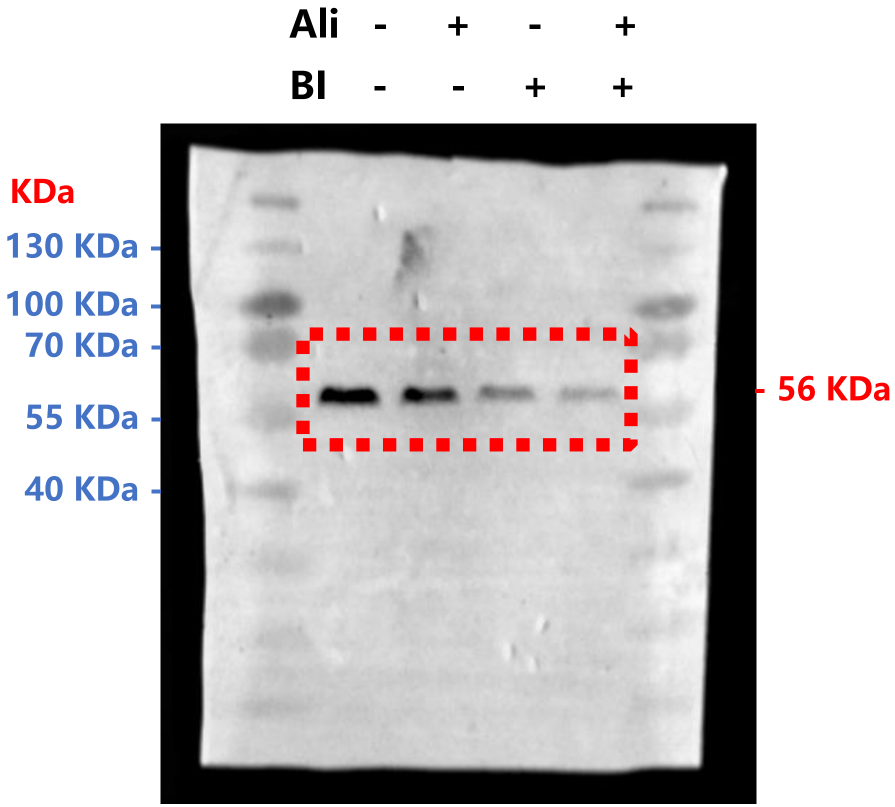

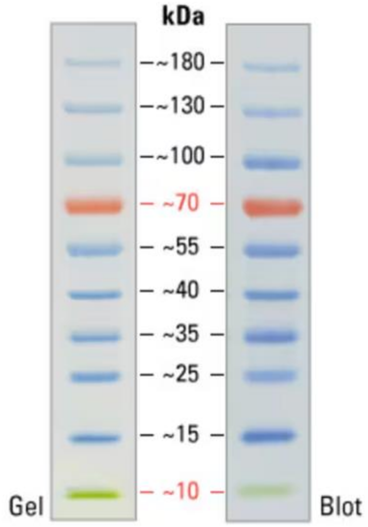

Thermo Scientific™ Marker-26617

■ Original Western Blots of Figure 3-B-14h

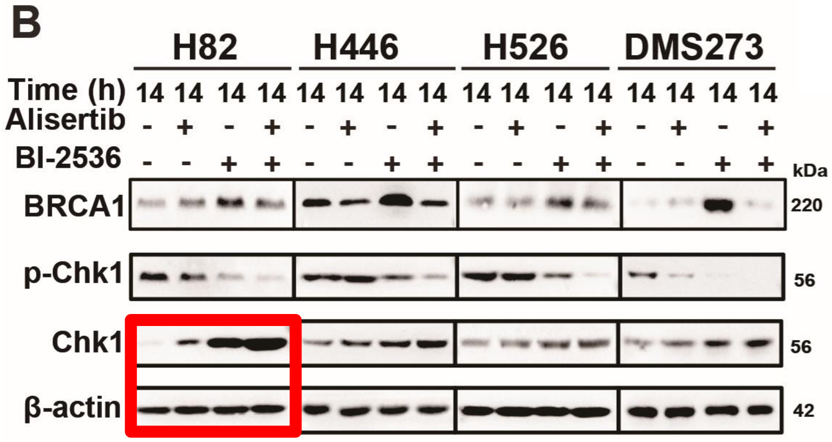

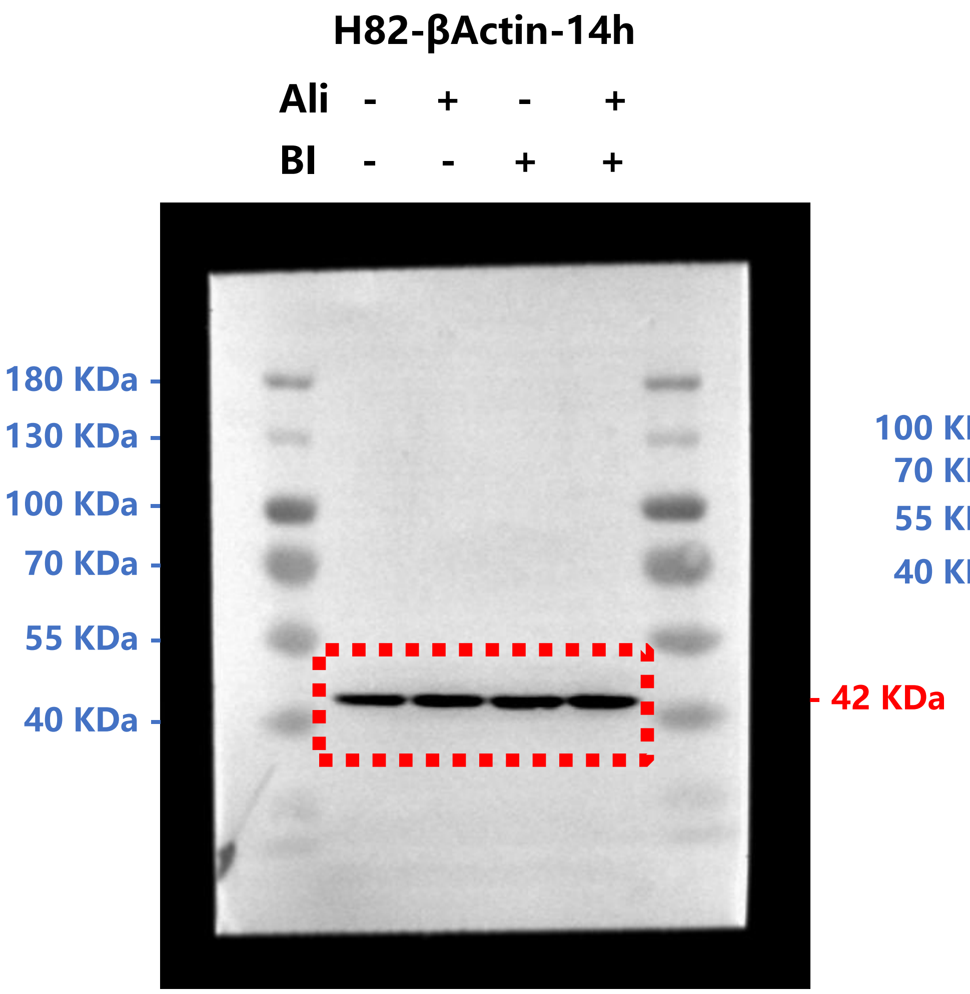

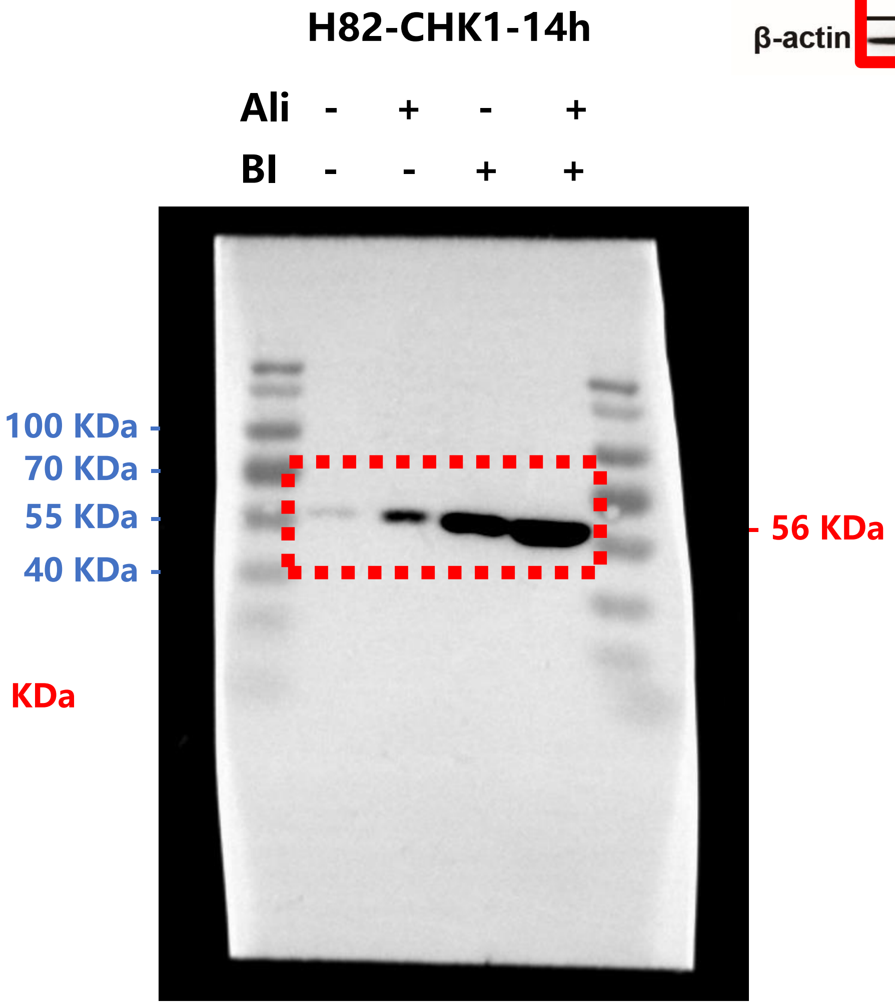

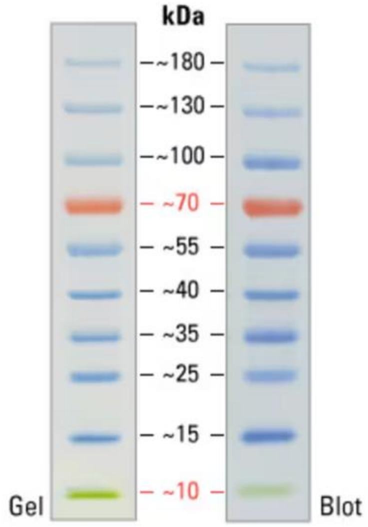

Thermo Scientific™ Marker-26617

■ Original Western Blots of Figure 3-B-14h

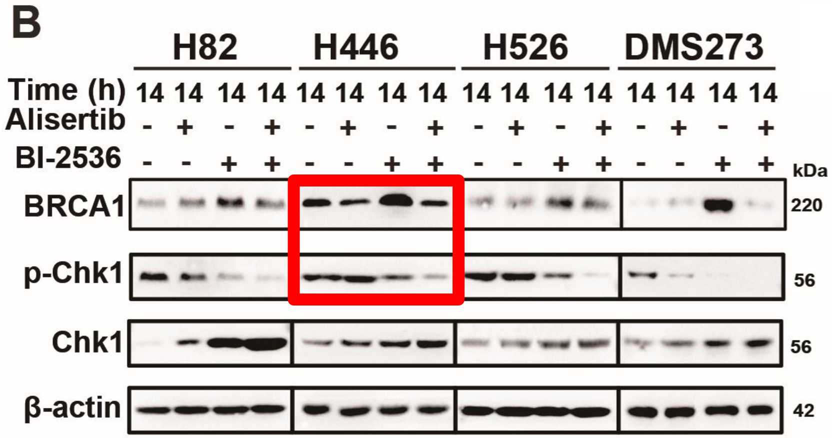

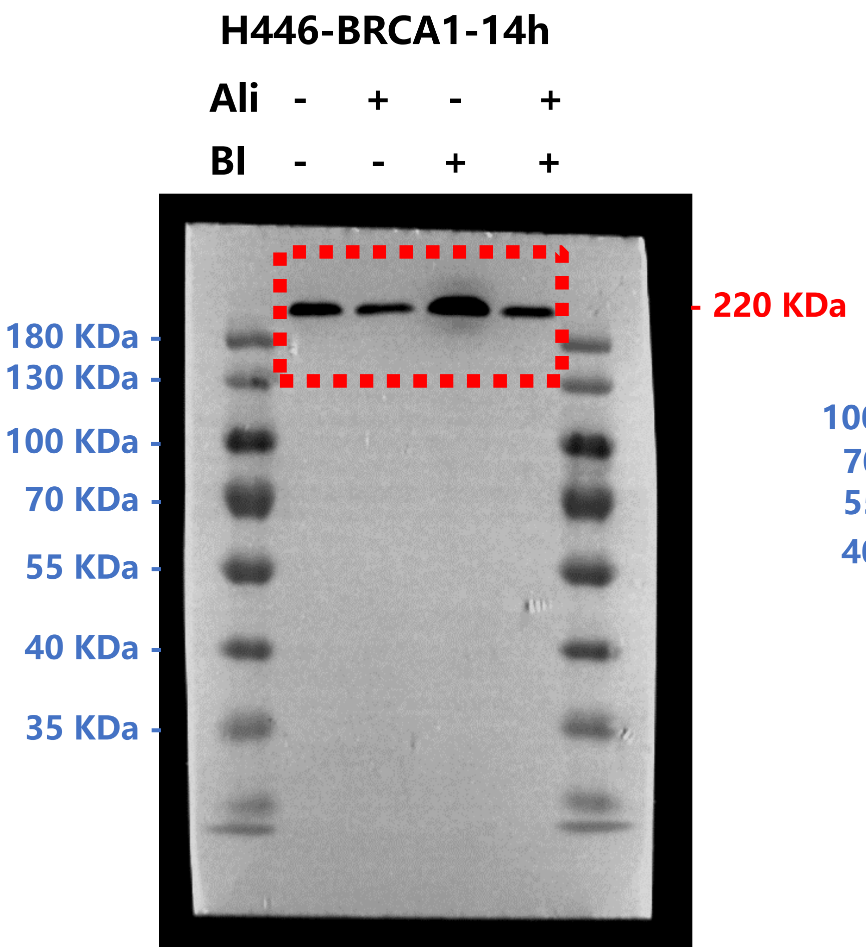

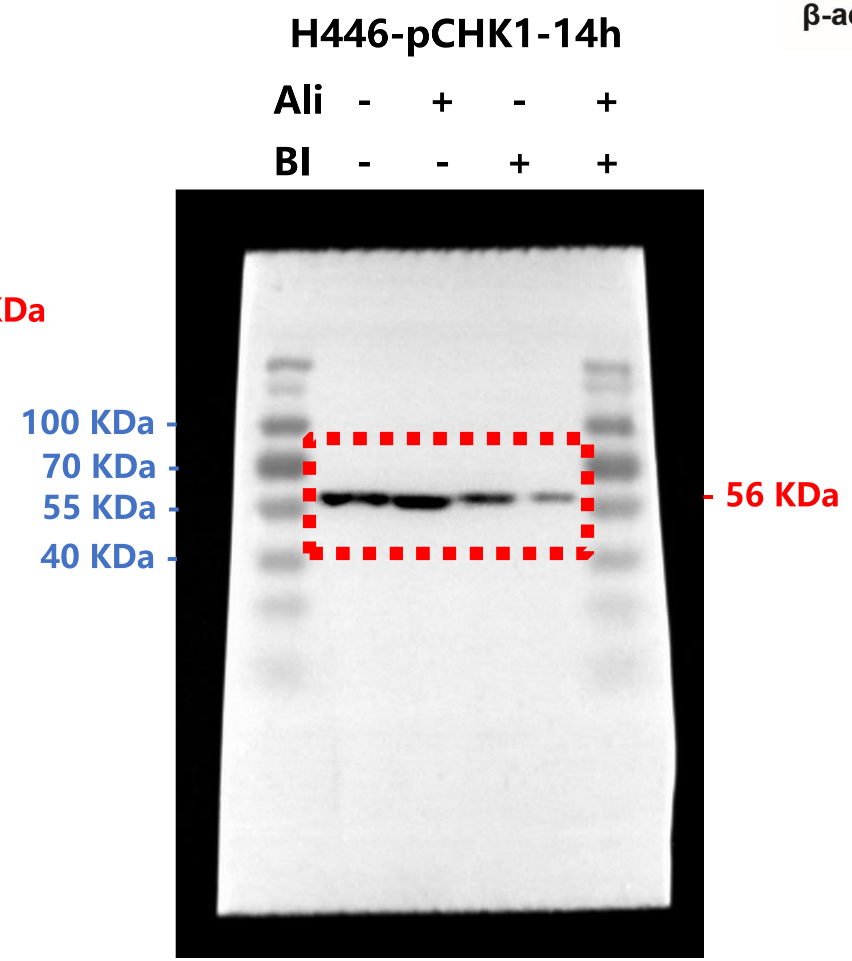

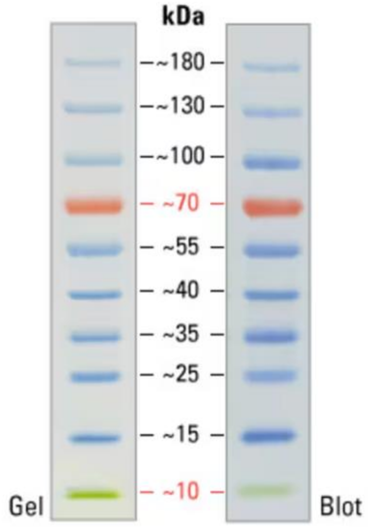

Thermo Scientific™ Marker-26617

■ Original Western Blots of Figure 3-B-14h

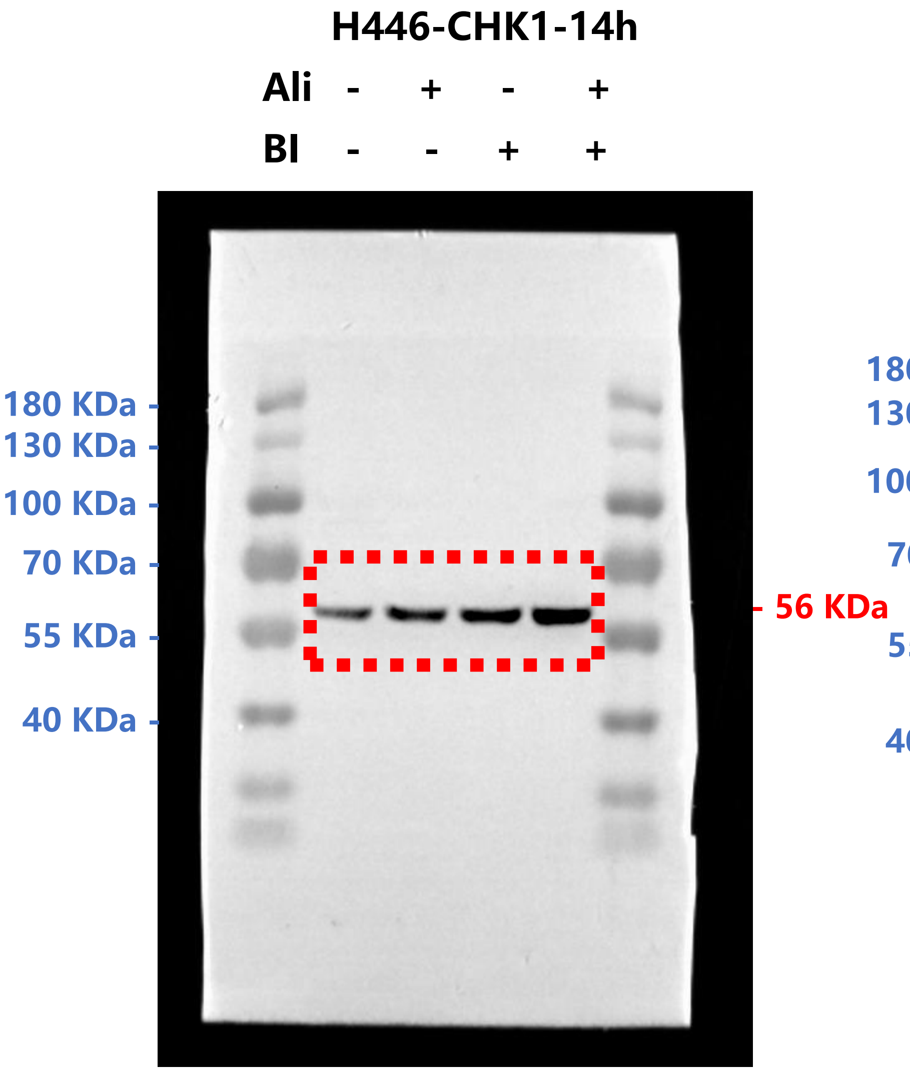

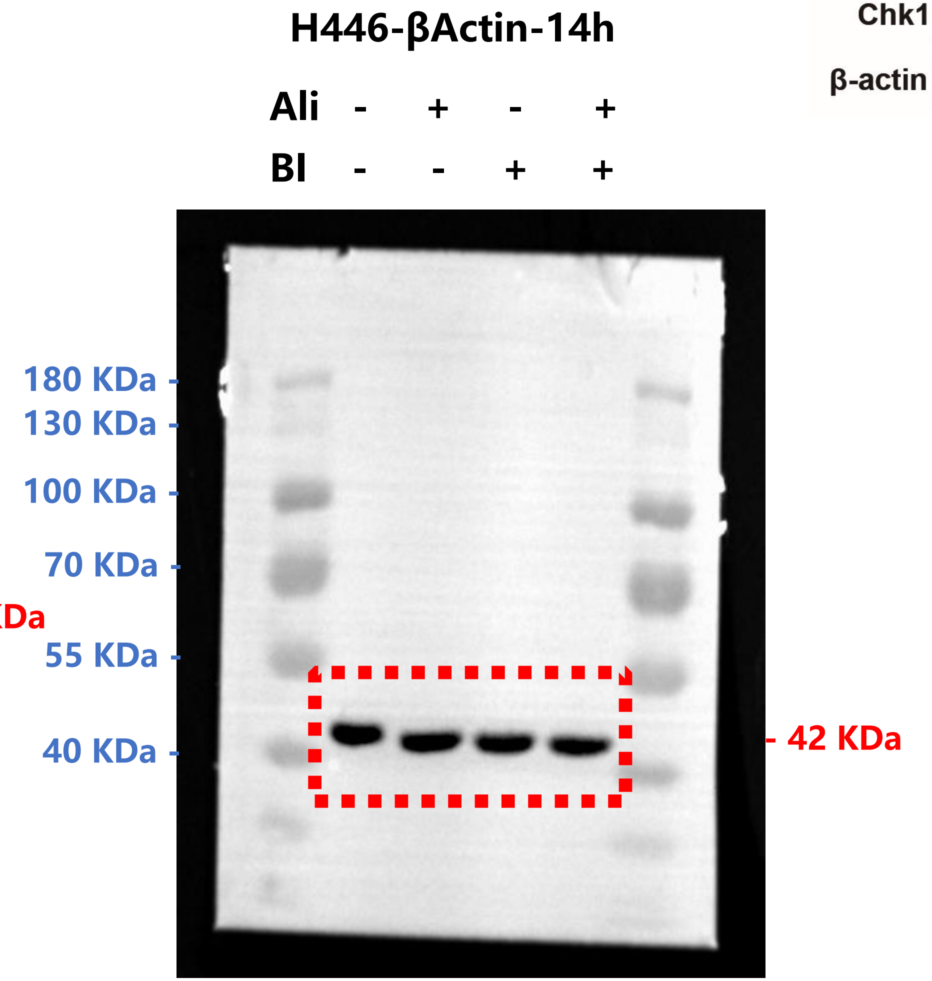

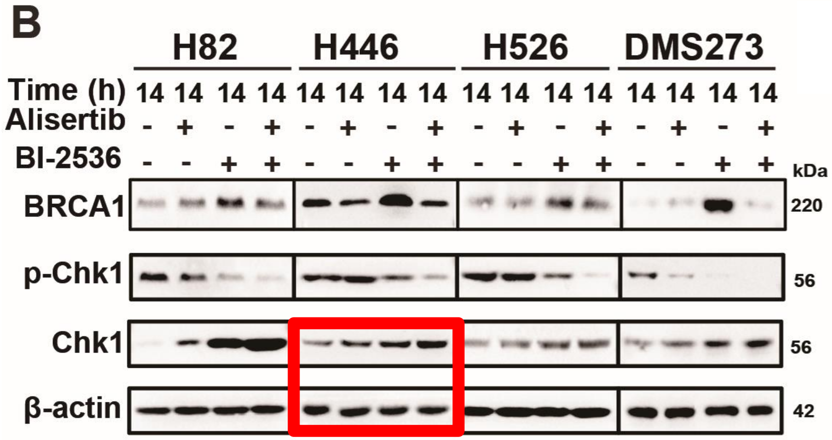

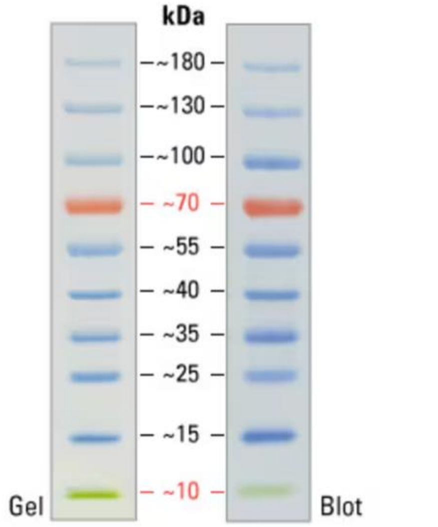

Thermo Scientific™ Marker-26617

■ Original Western Blots of Figure 3-B-14h

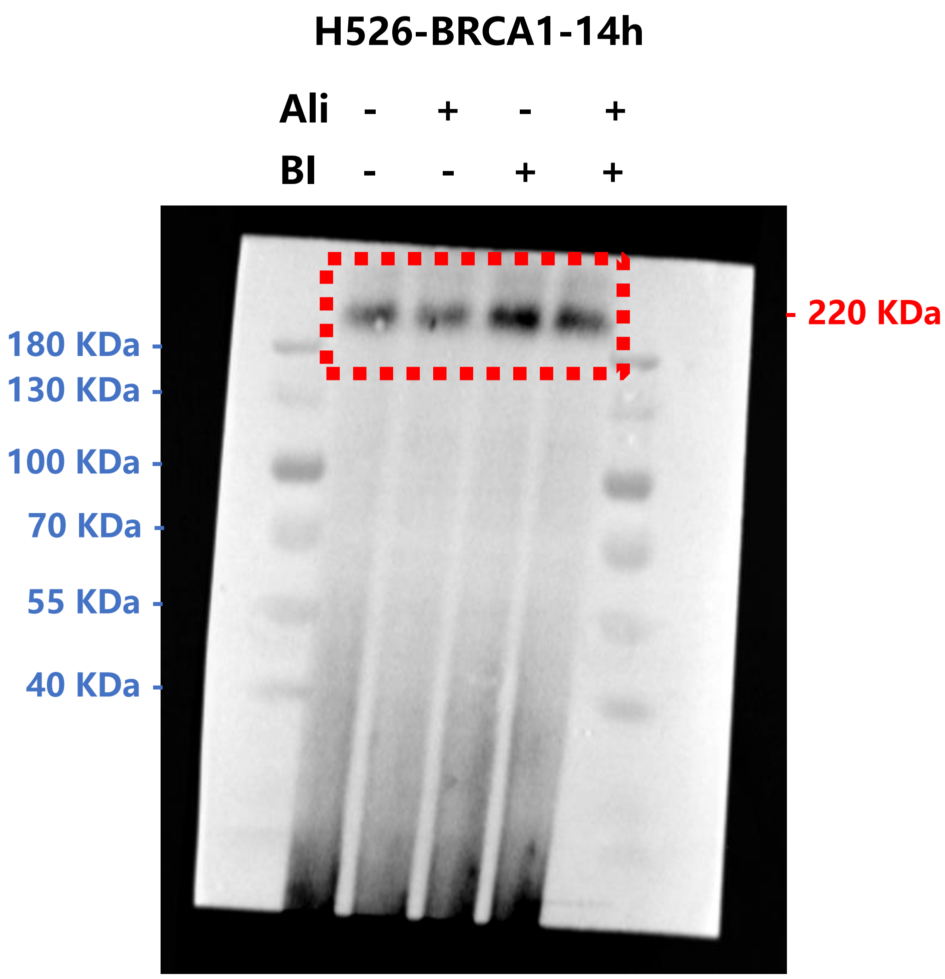

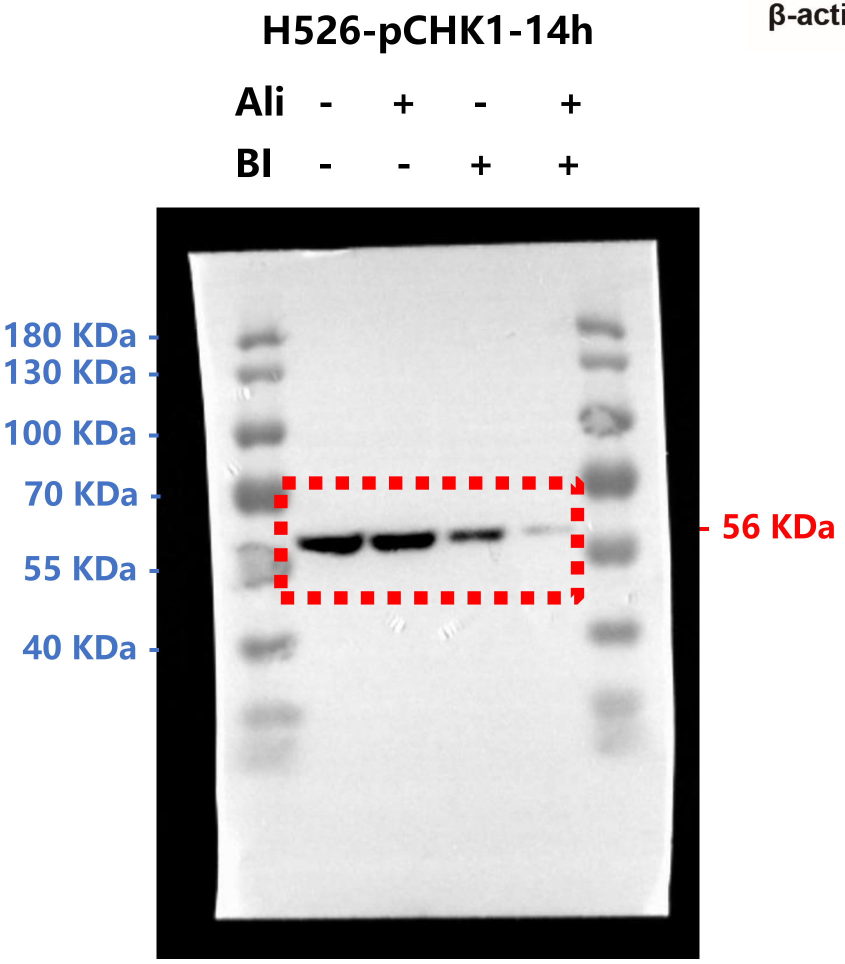

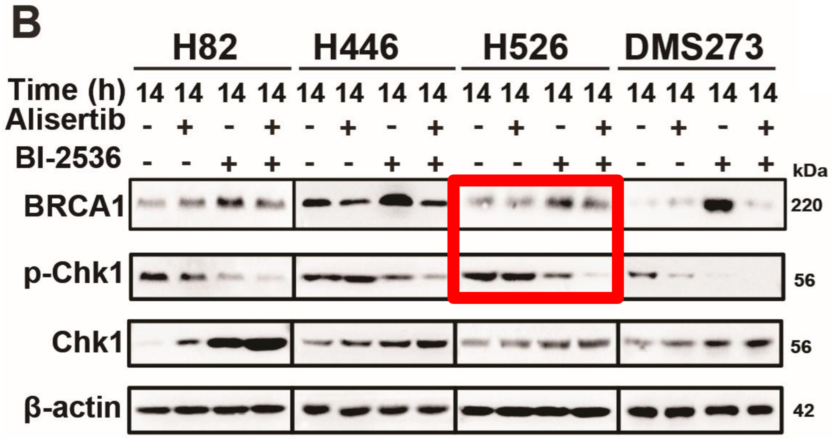

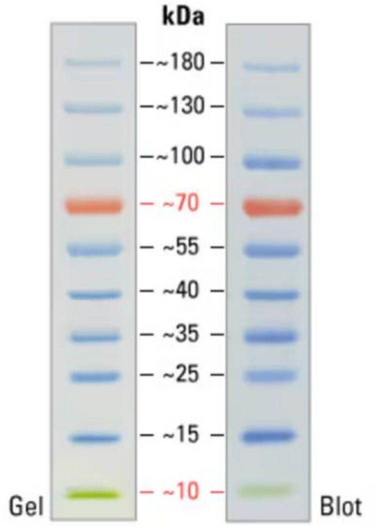

Thermo Scientific™ Marker-26617

■ Original Western Blots of Figure 3-B-14h

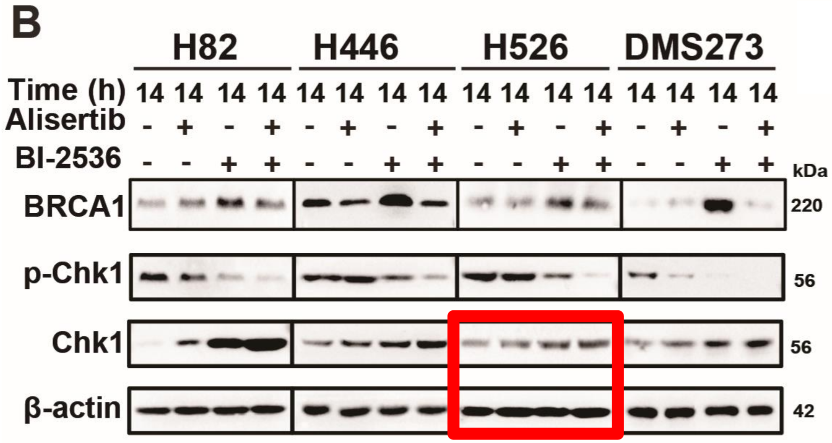

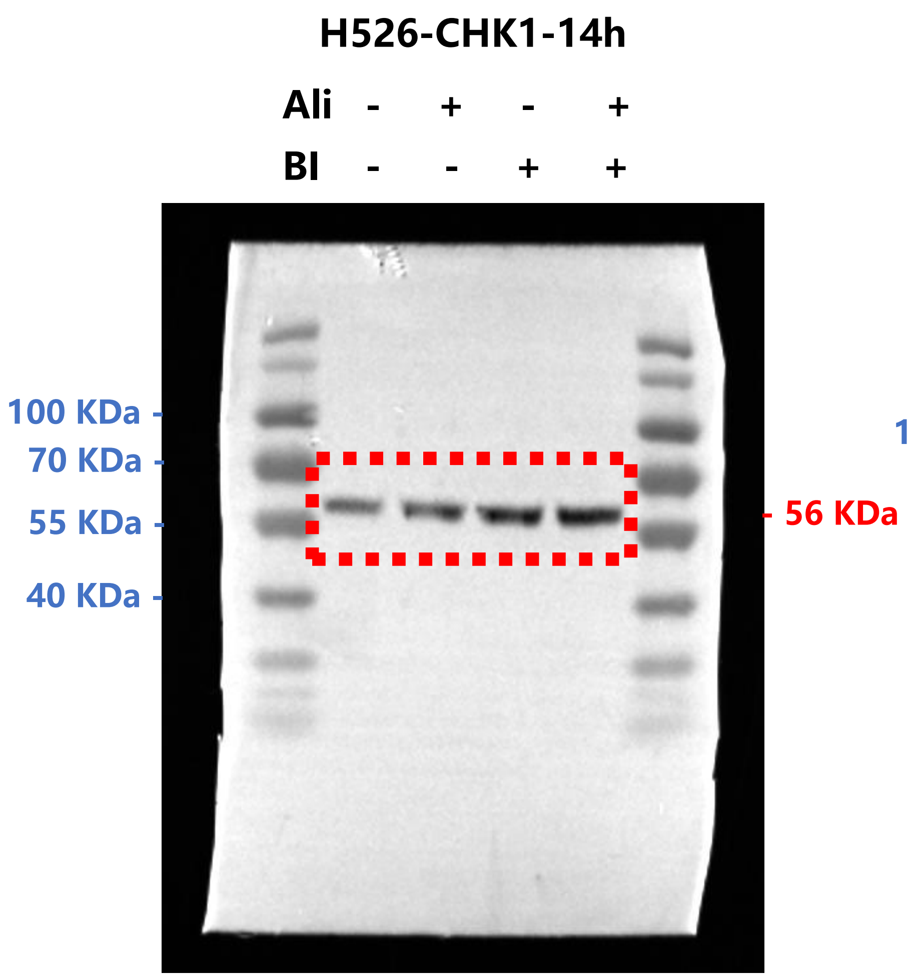

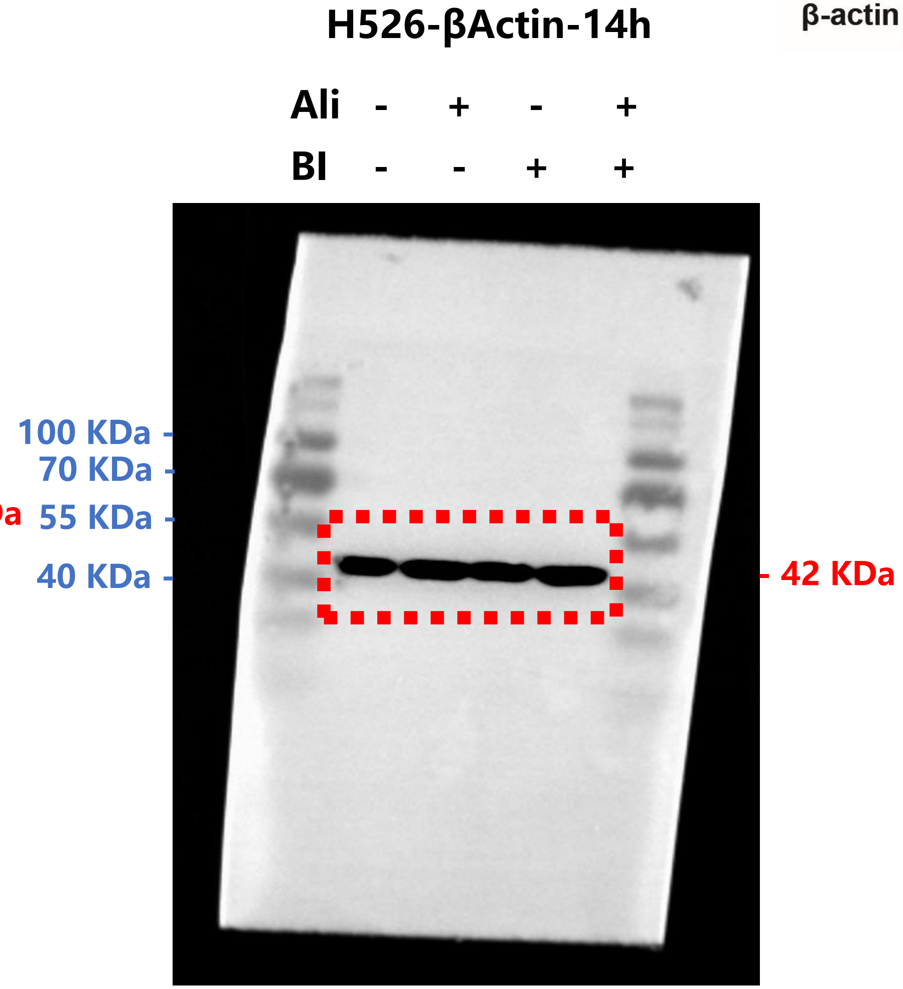

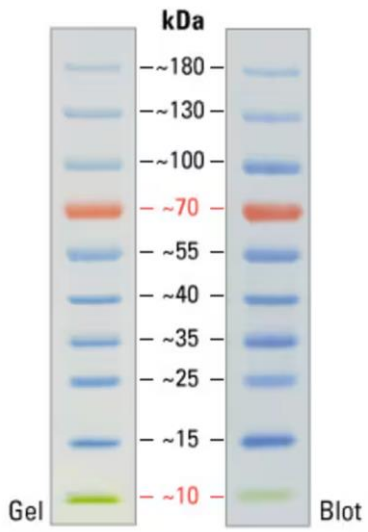

Thermo Scientific™ Marker-26617

■ Original Western Blots of Figure 3-B-14h

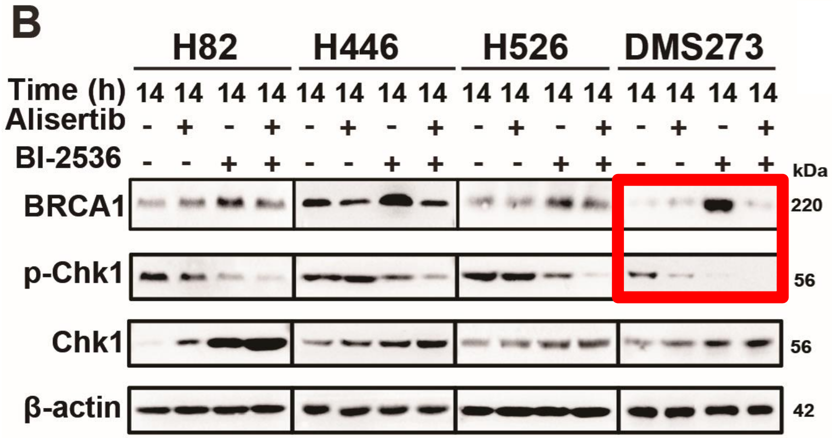

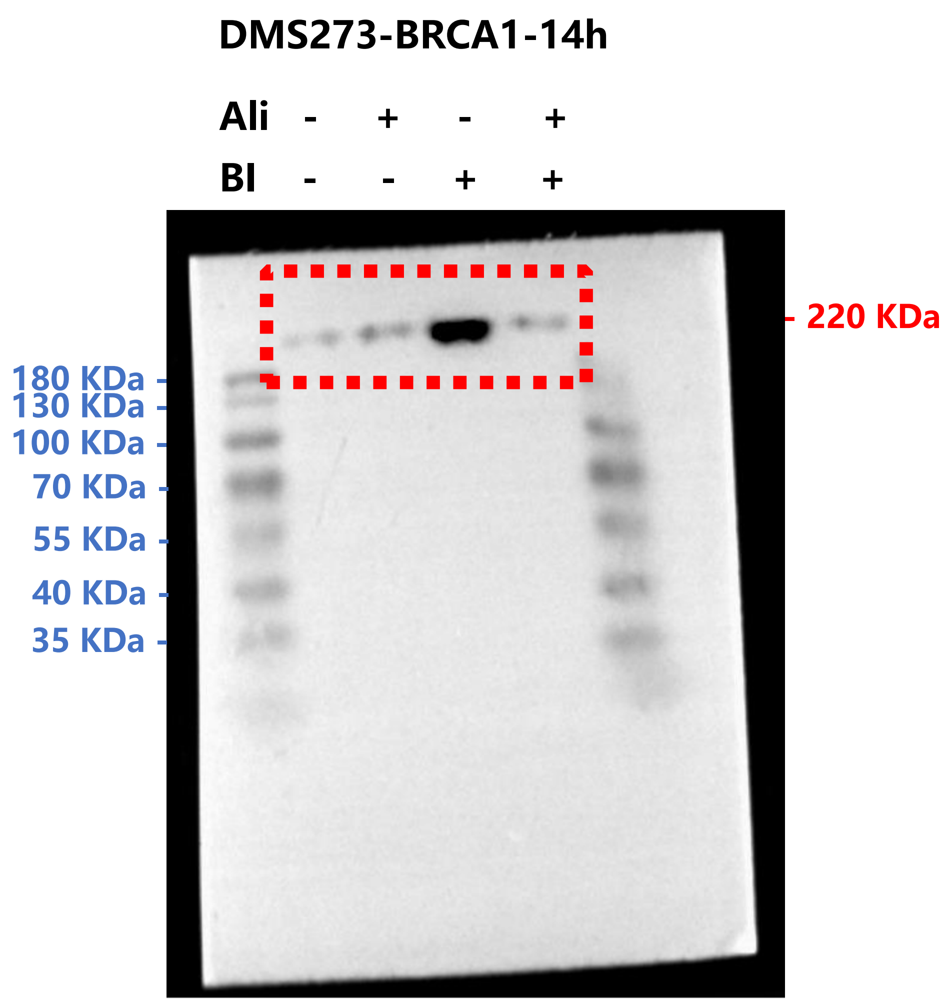

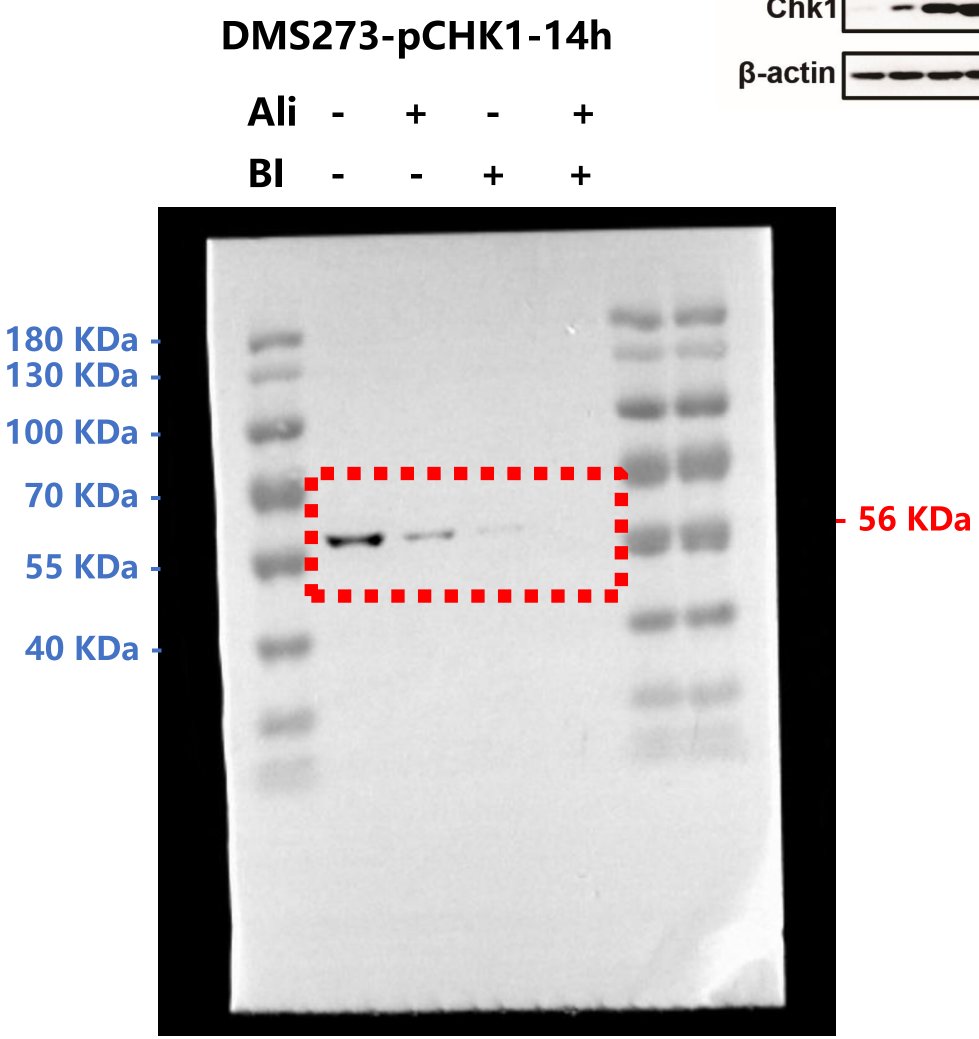

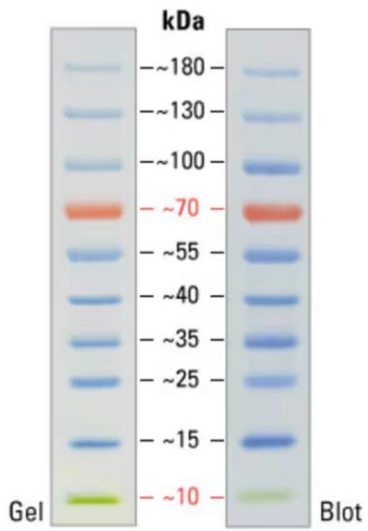

Thermo Scientific™ Marker-26617

■ Original Western Blots of Figure 3-B-14h

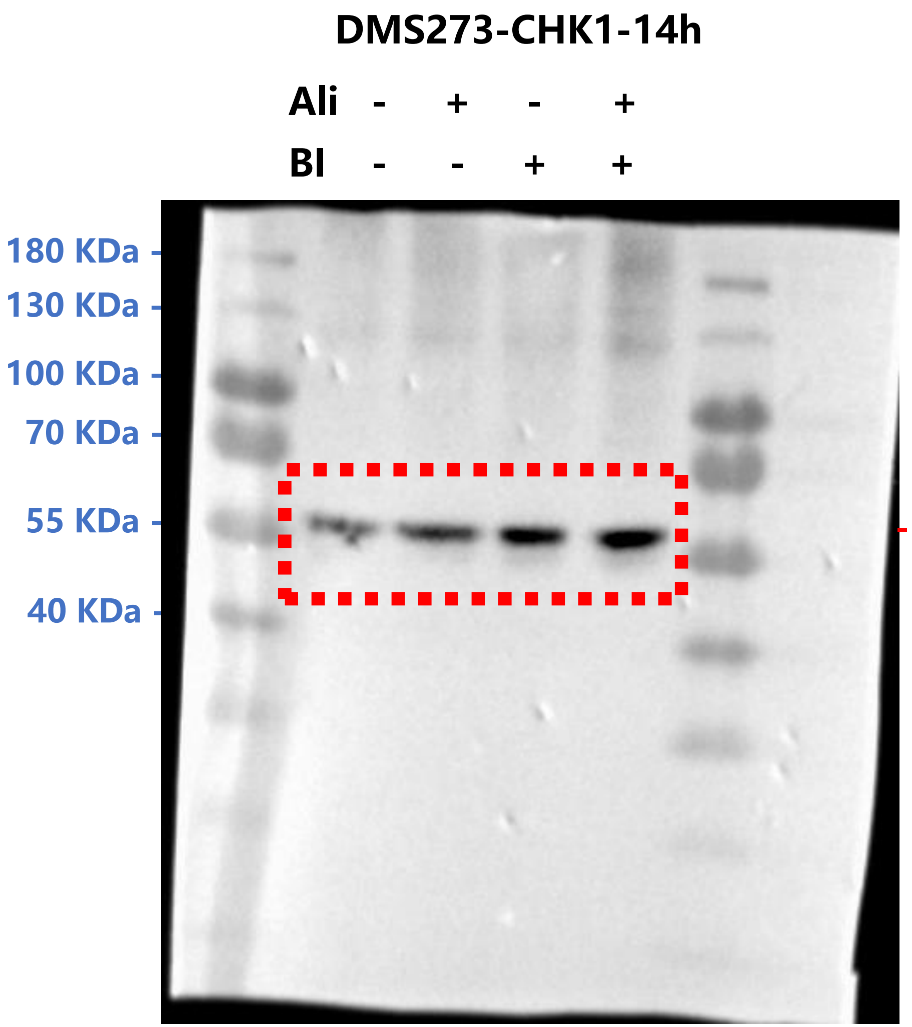

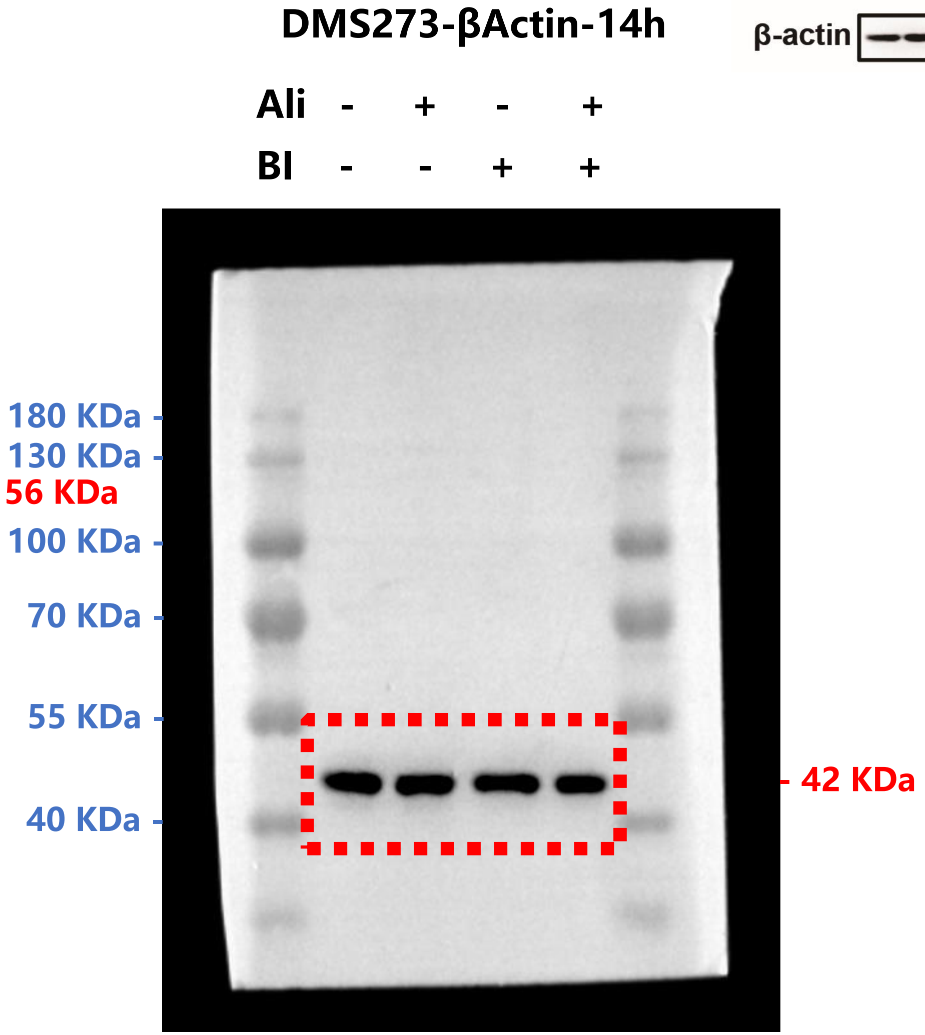

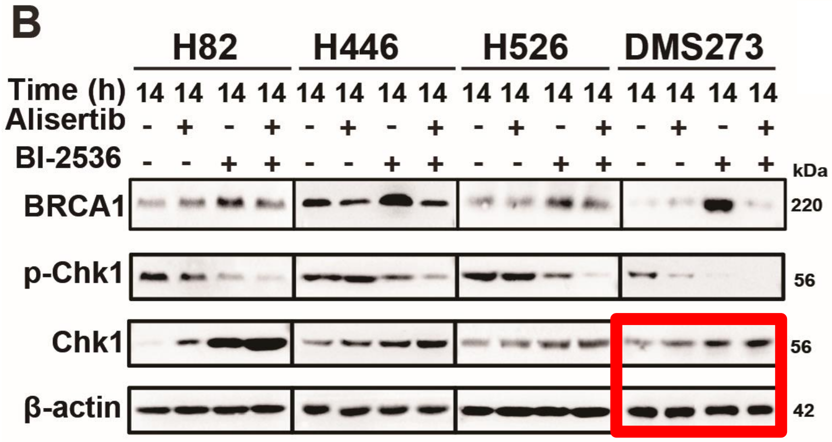

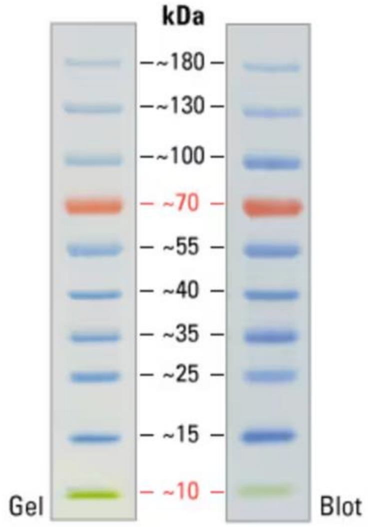

Thermo Scientific™ Marker-26617

■ **Original Western Blots of Figure S4-B**

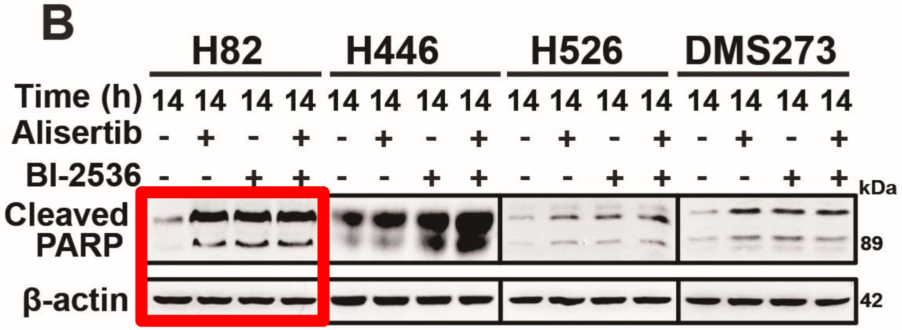

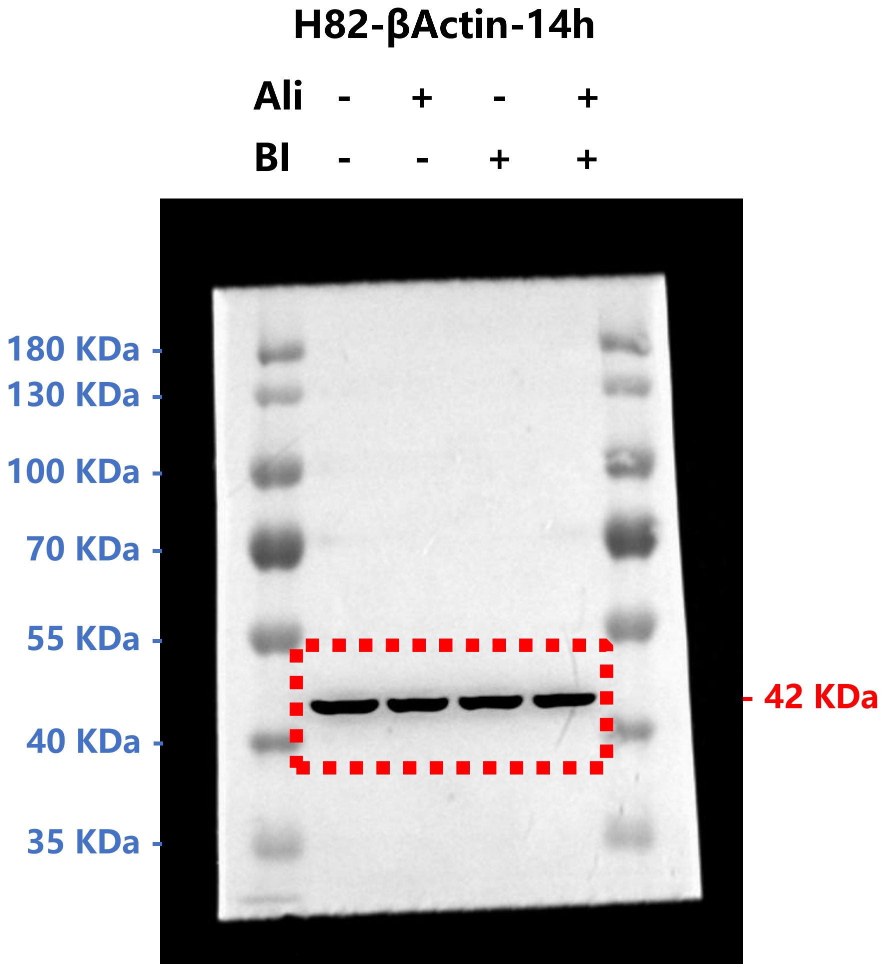

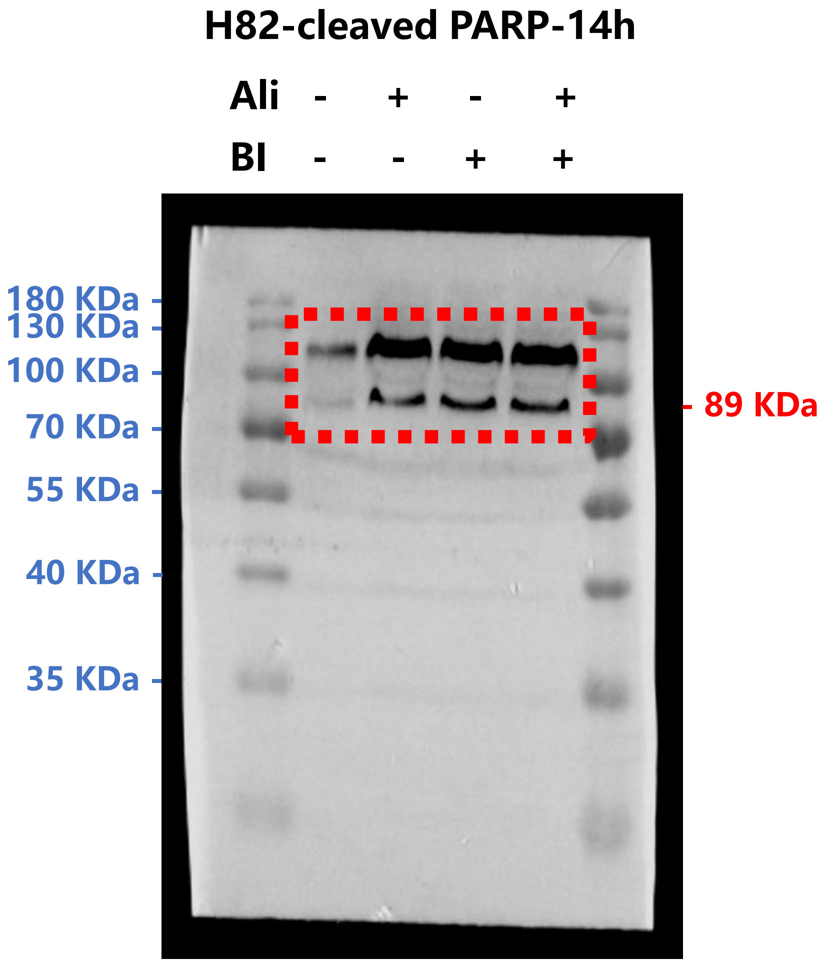

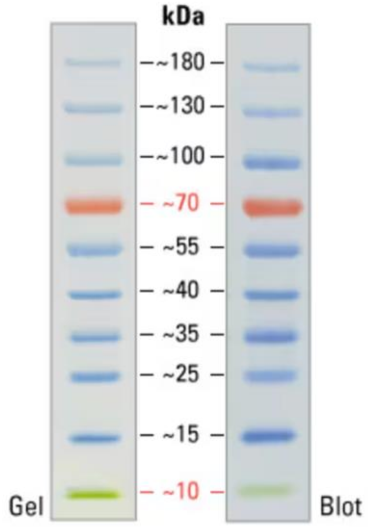

Thermo Scientific™ Marker-26617

■ Original Western Blots of Figure S4-B

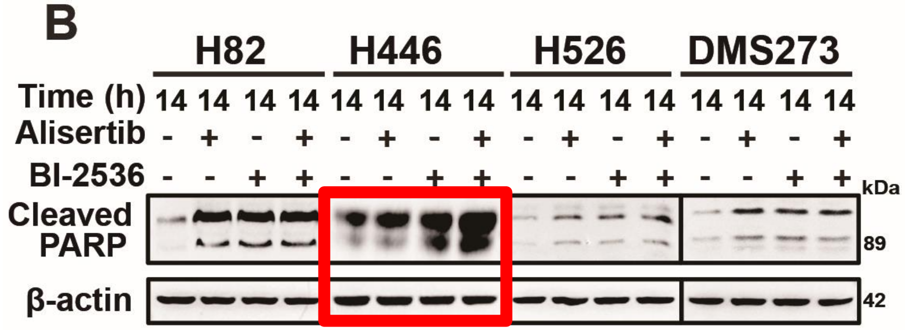

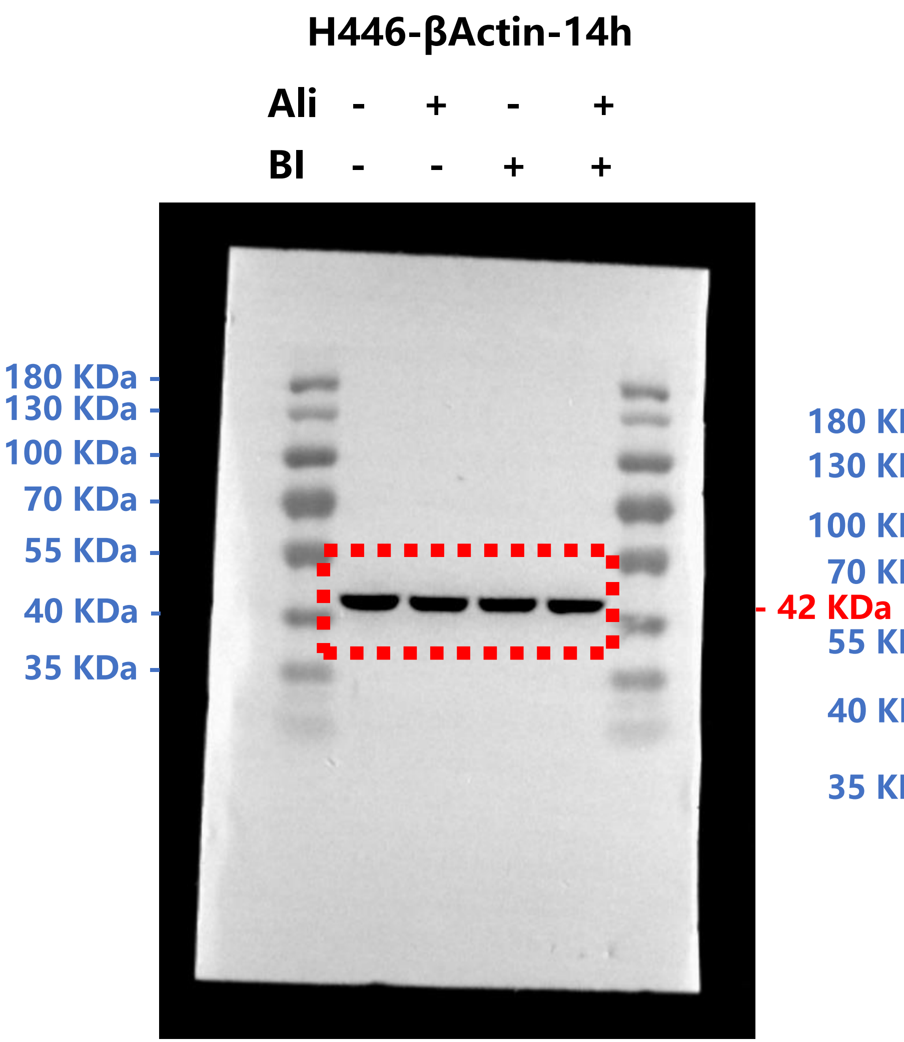

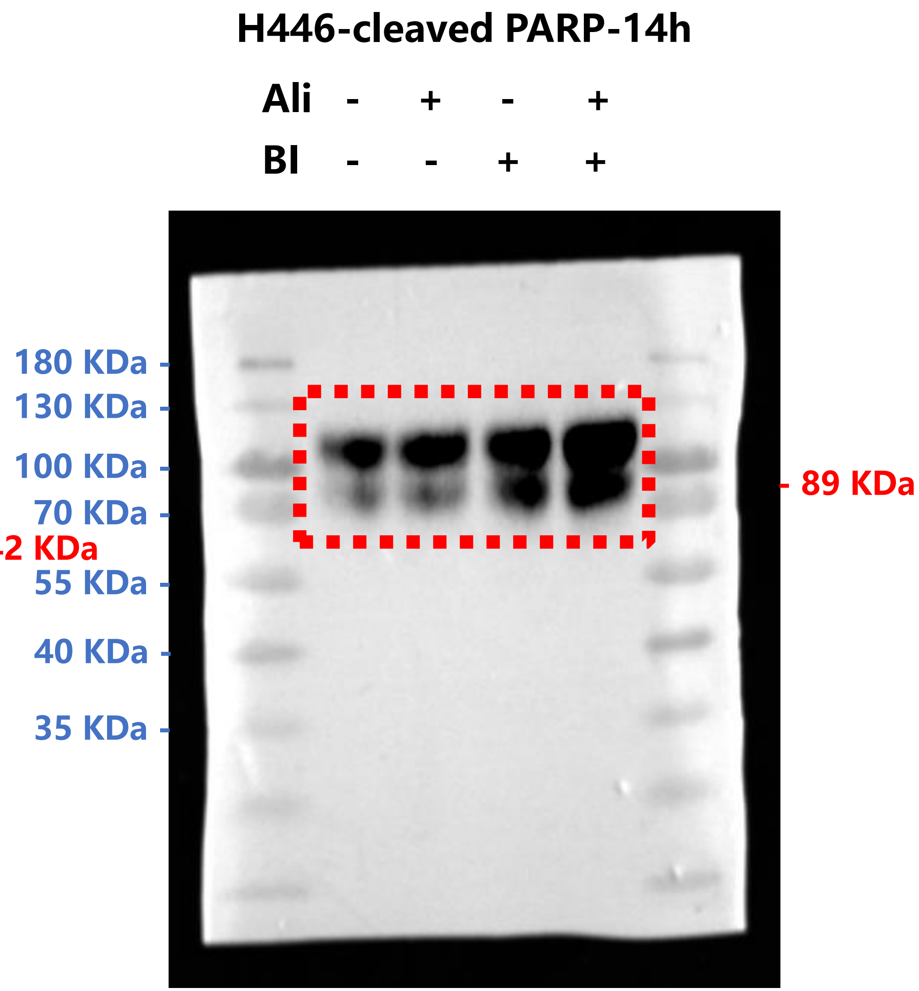

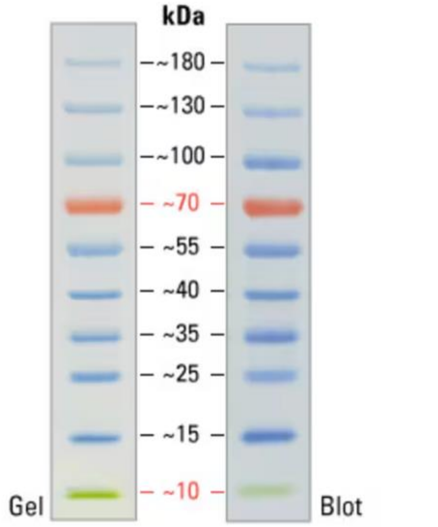

Thermo Scientific™ Marker-26617

■ Original Western Blots of Figure S4-B

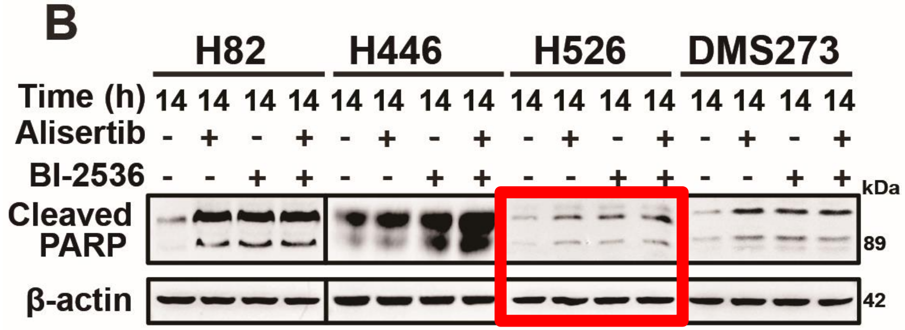

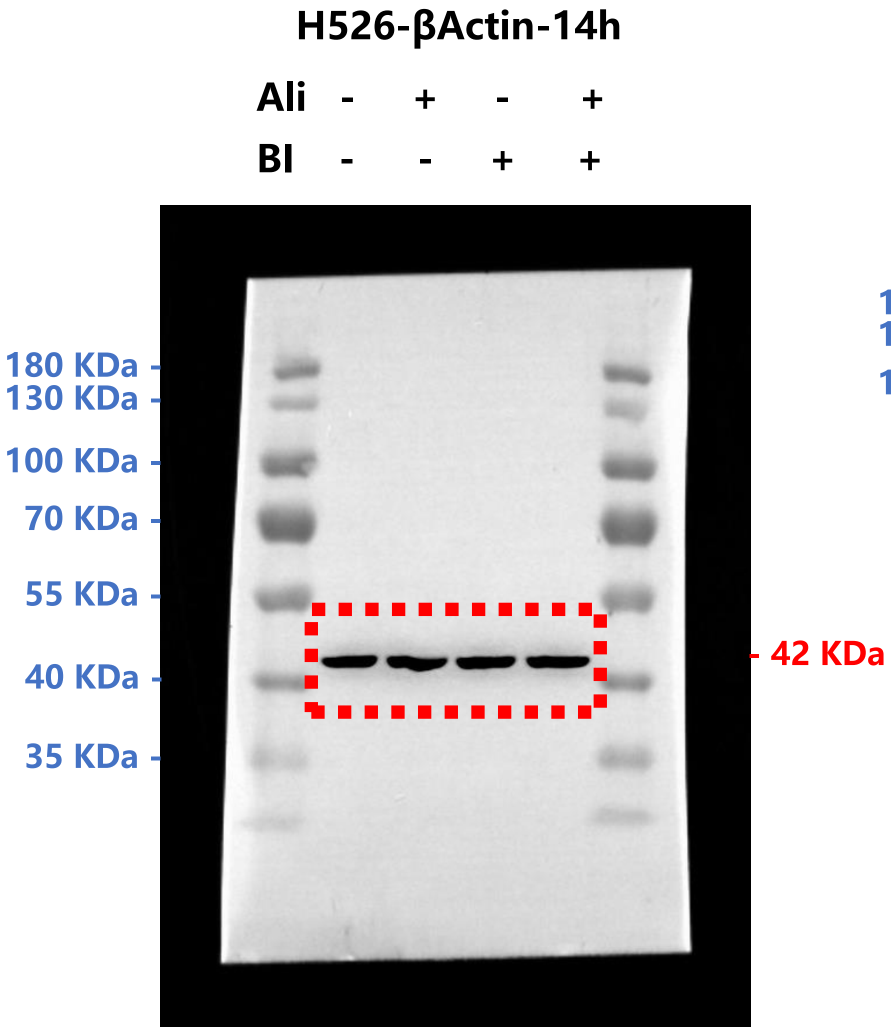

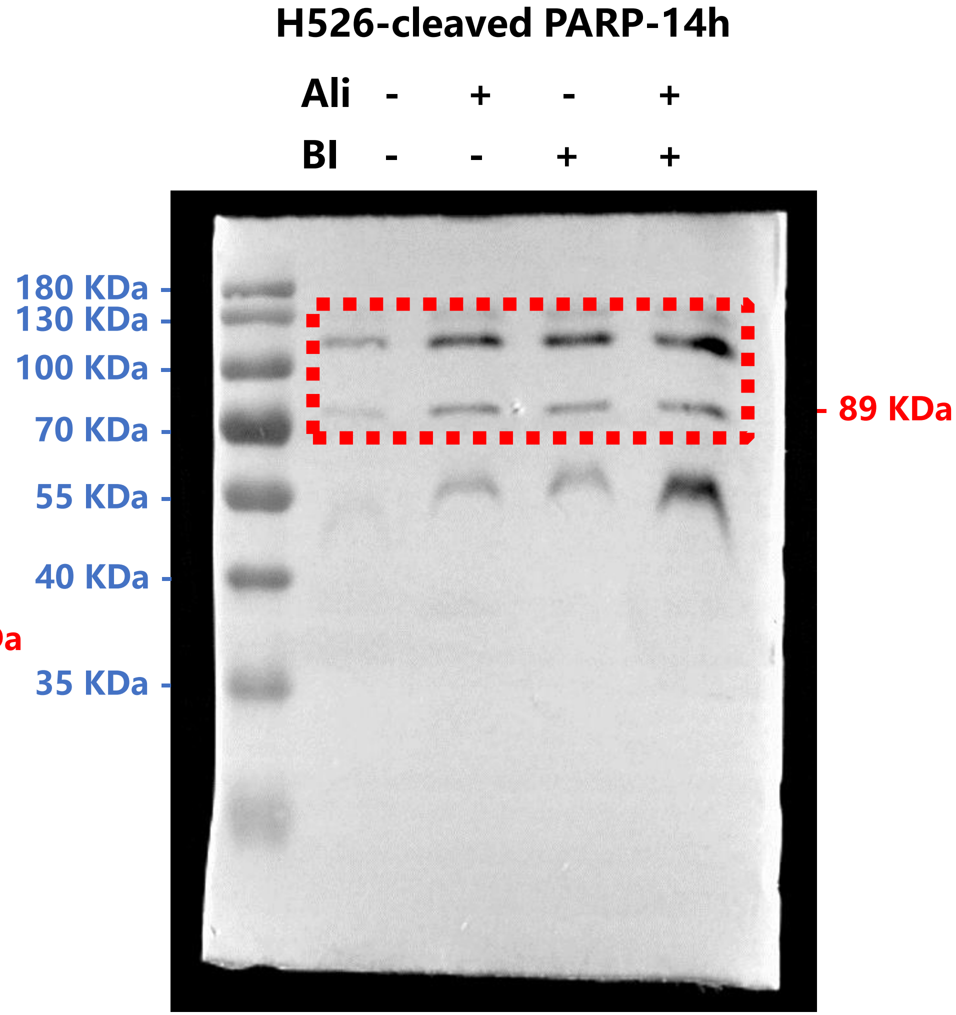

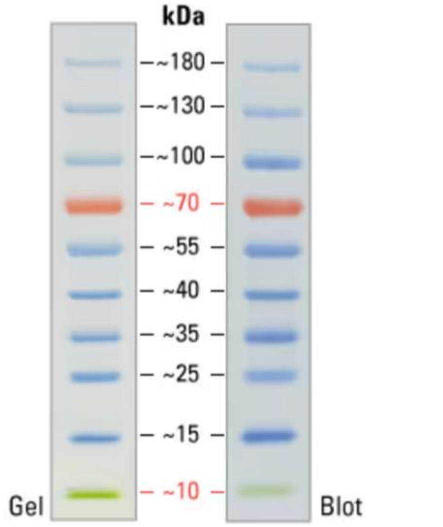

Thermo Scientific™ Marker-26617

■ Original Western Blots of Figure S4-B

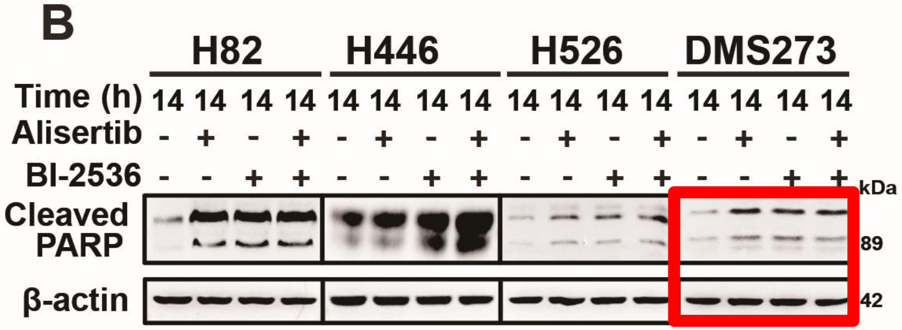

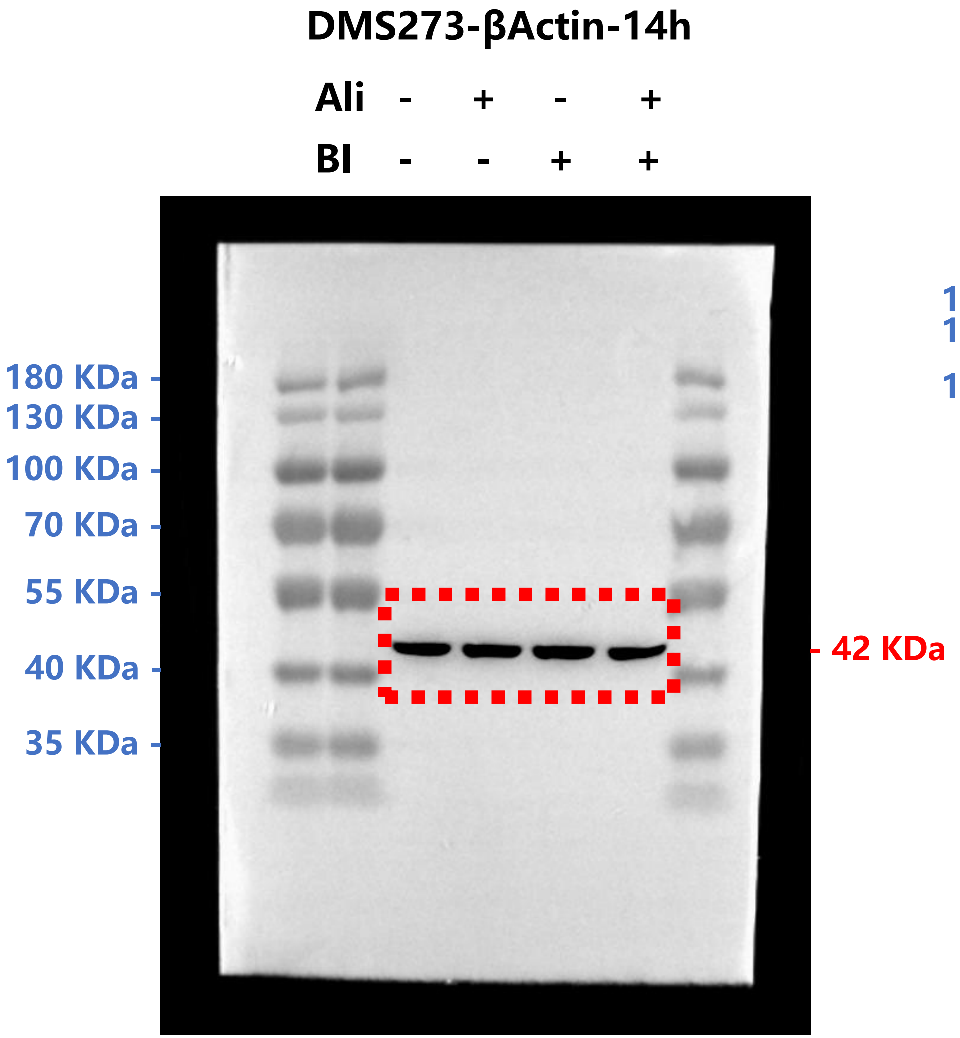

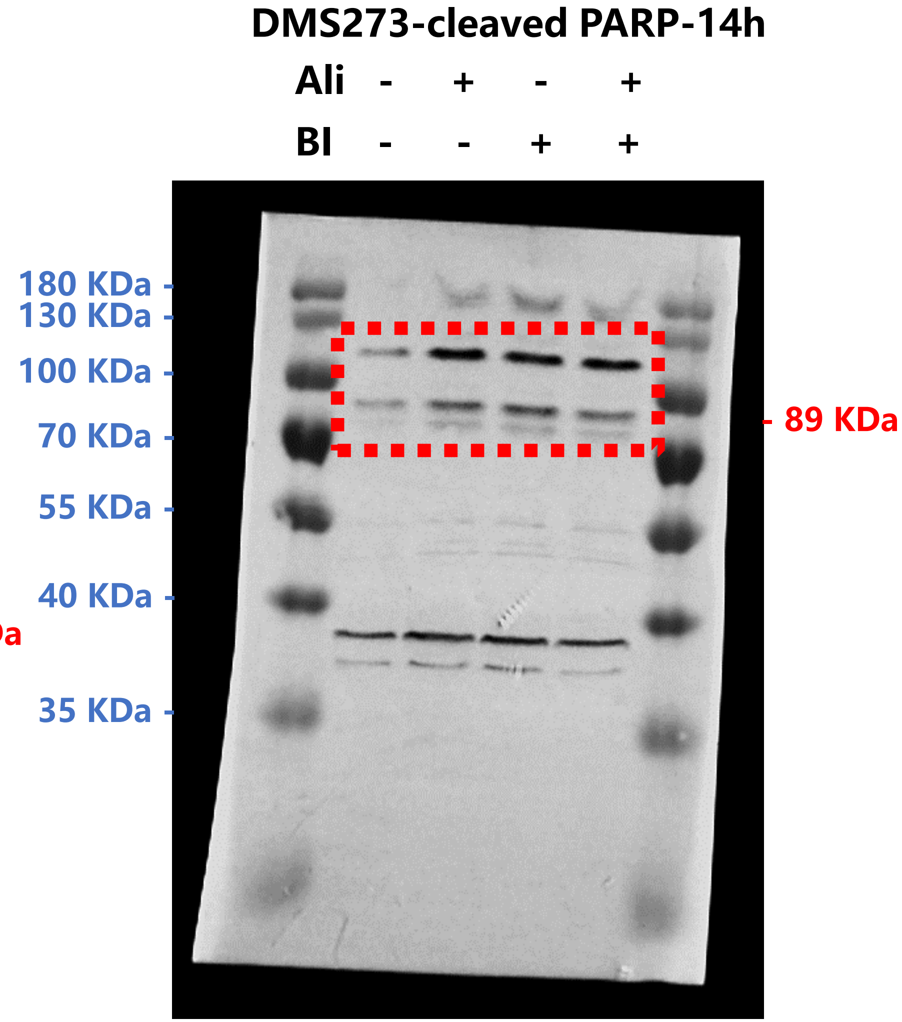

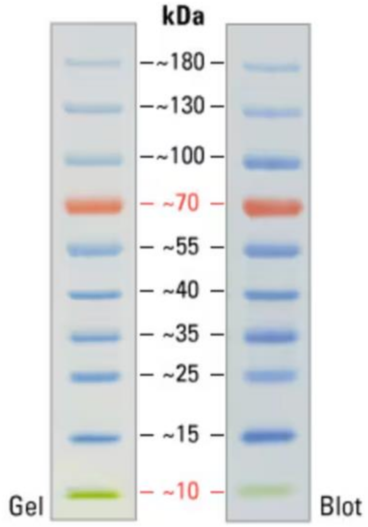

Thermo Scientific™ Marker-26617

■ **Original Western Blots of Figure S4-C**

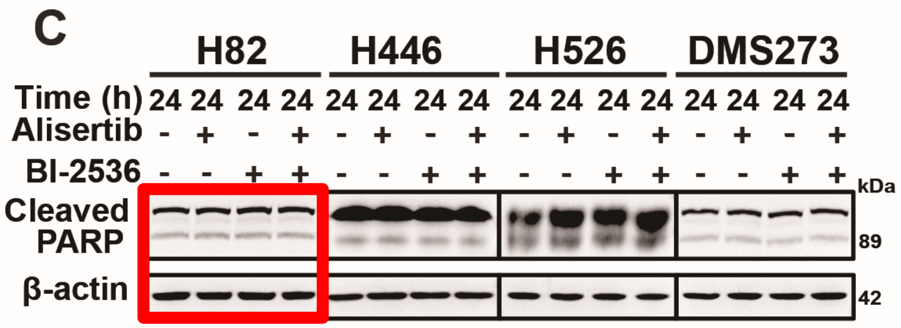

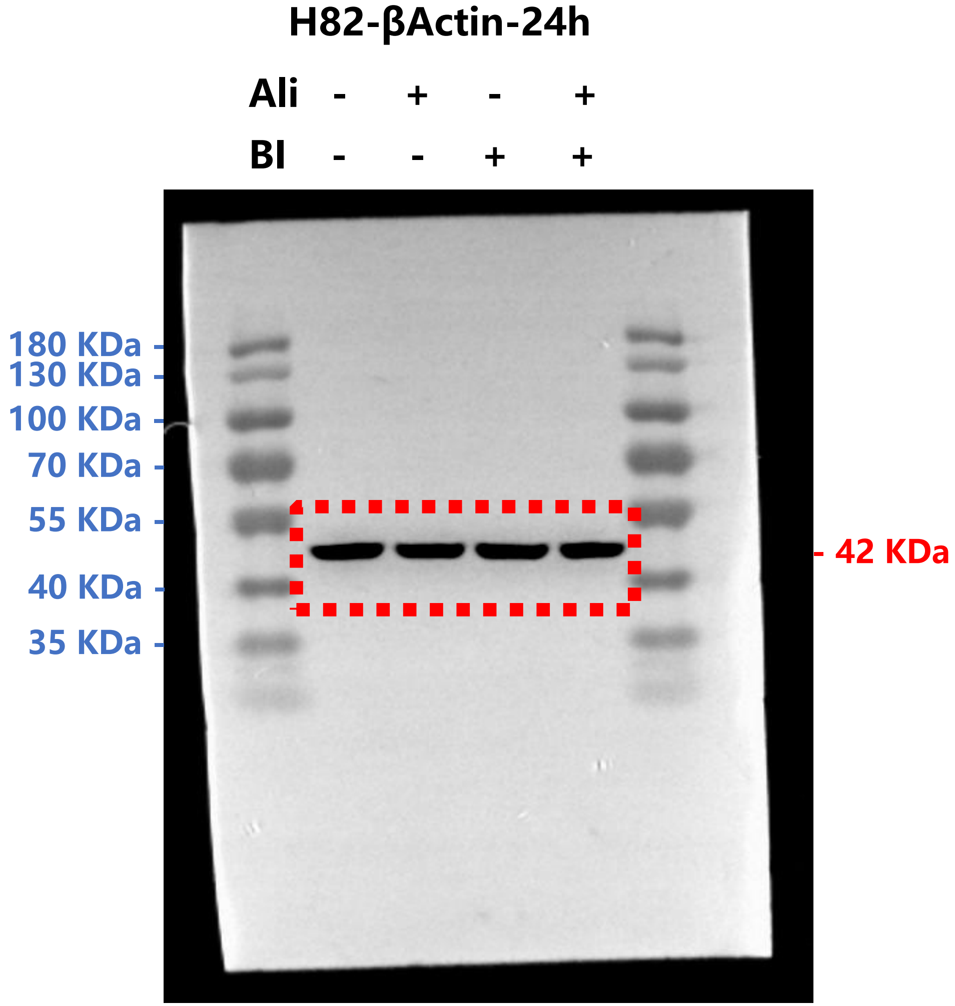

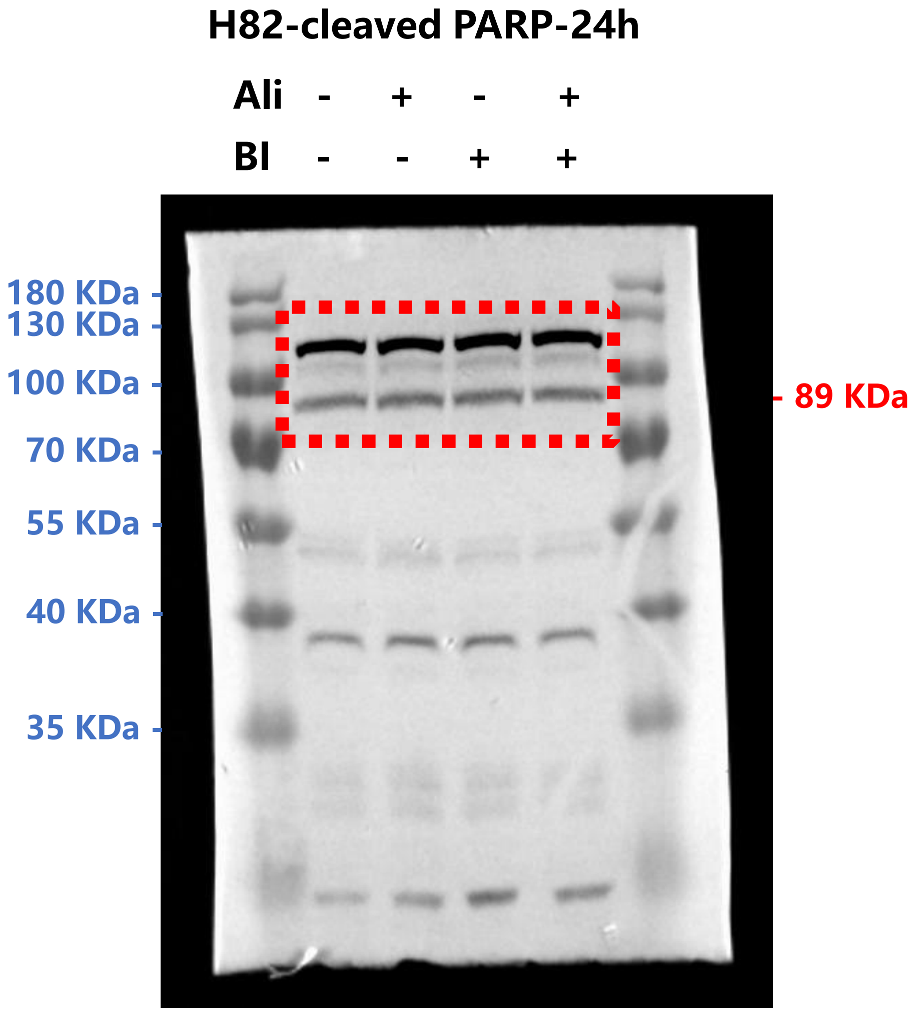

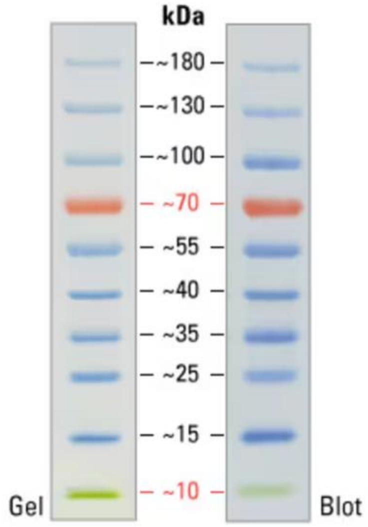

Thermo Scientific™ Marker-26617

■ Original Western Blots of Figure S4-C

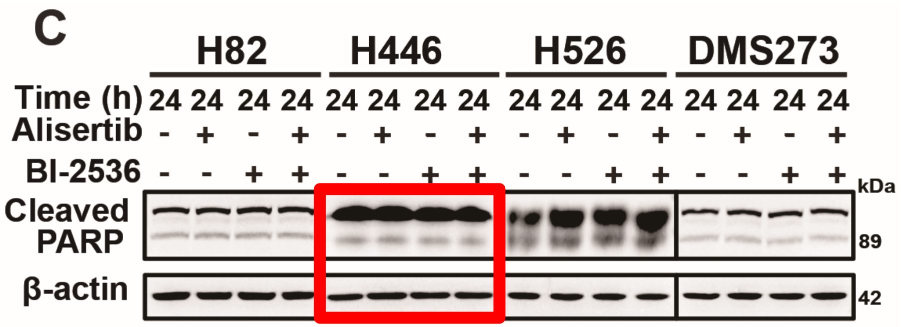

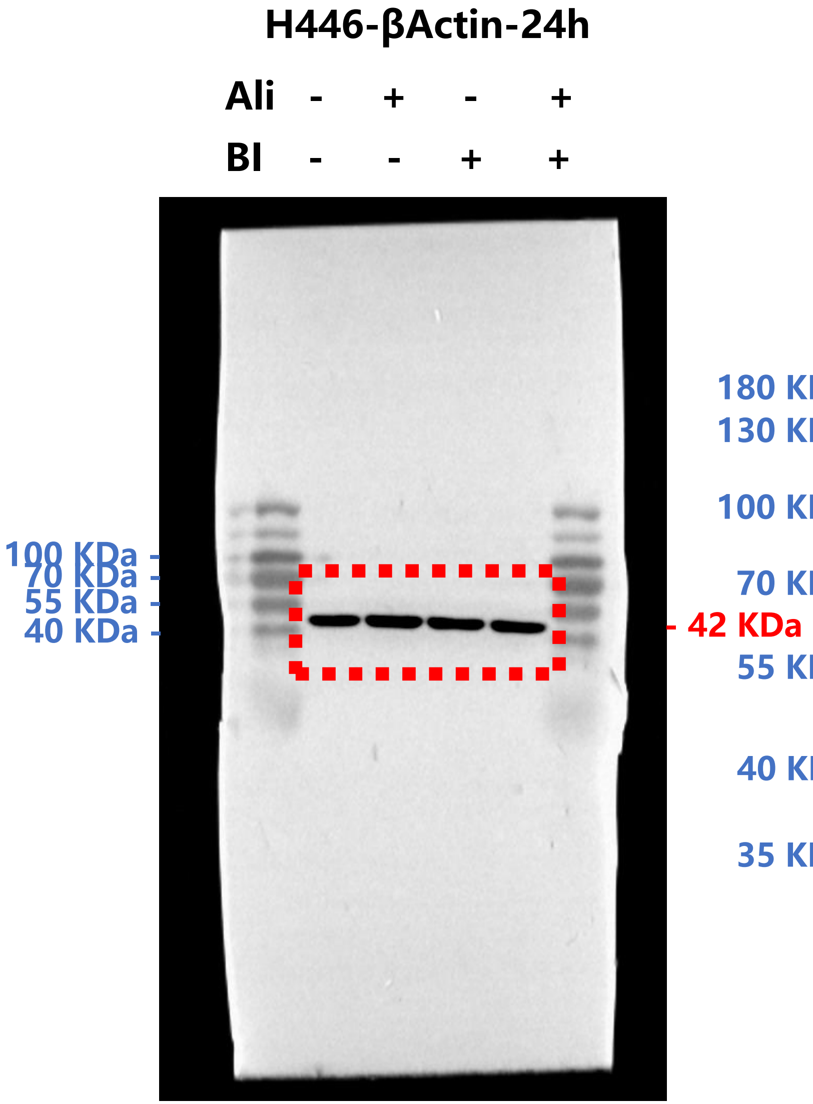

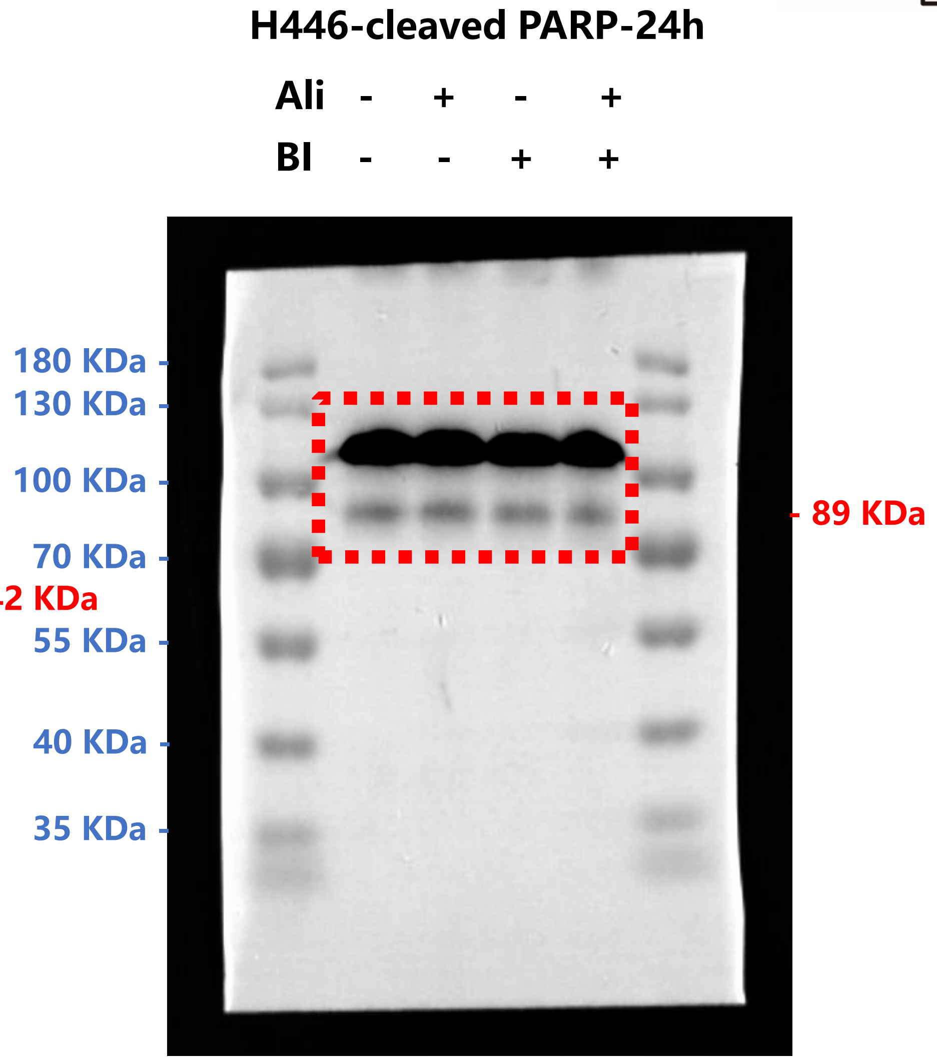

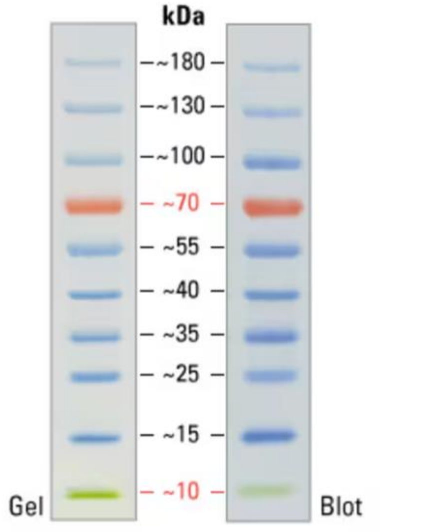

Thermo Scientific™ Marker-26617

■ Original Western Blots of Figure S4-C

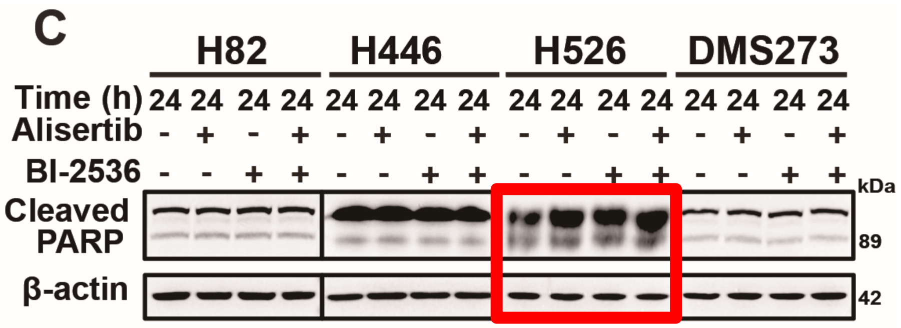

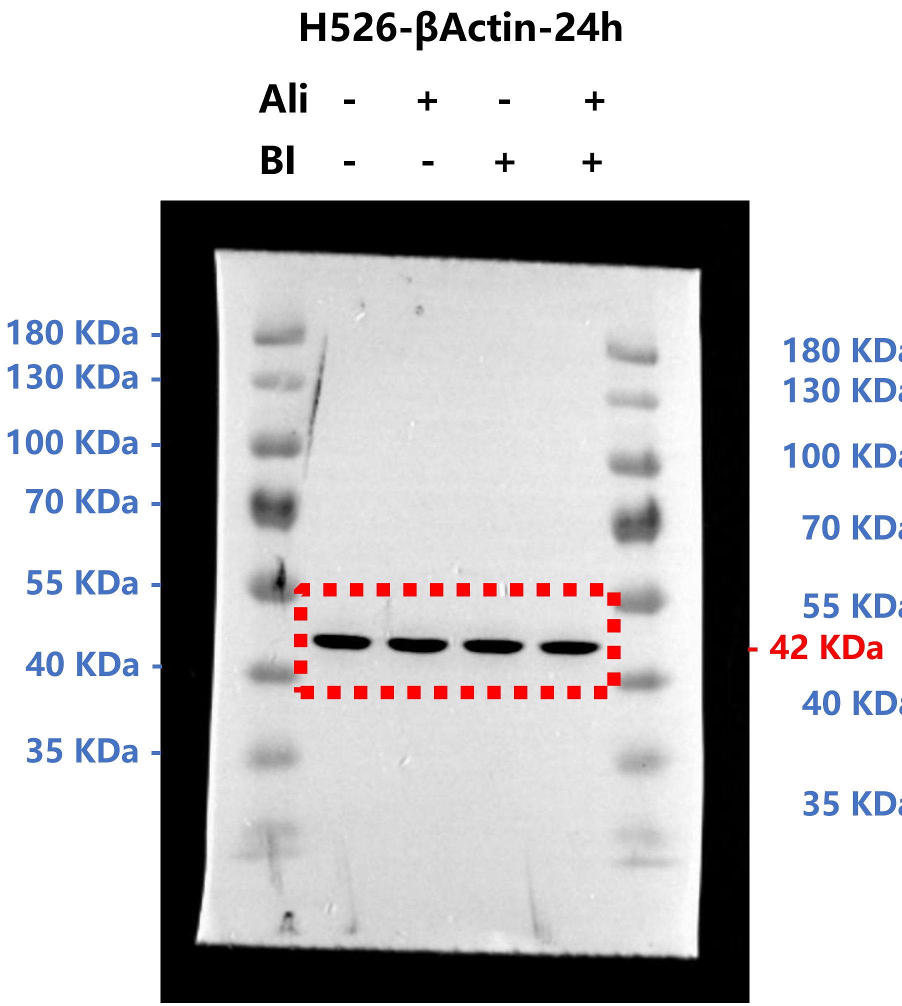

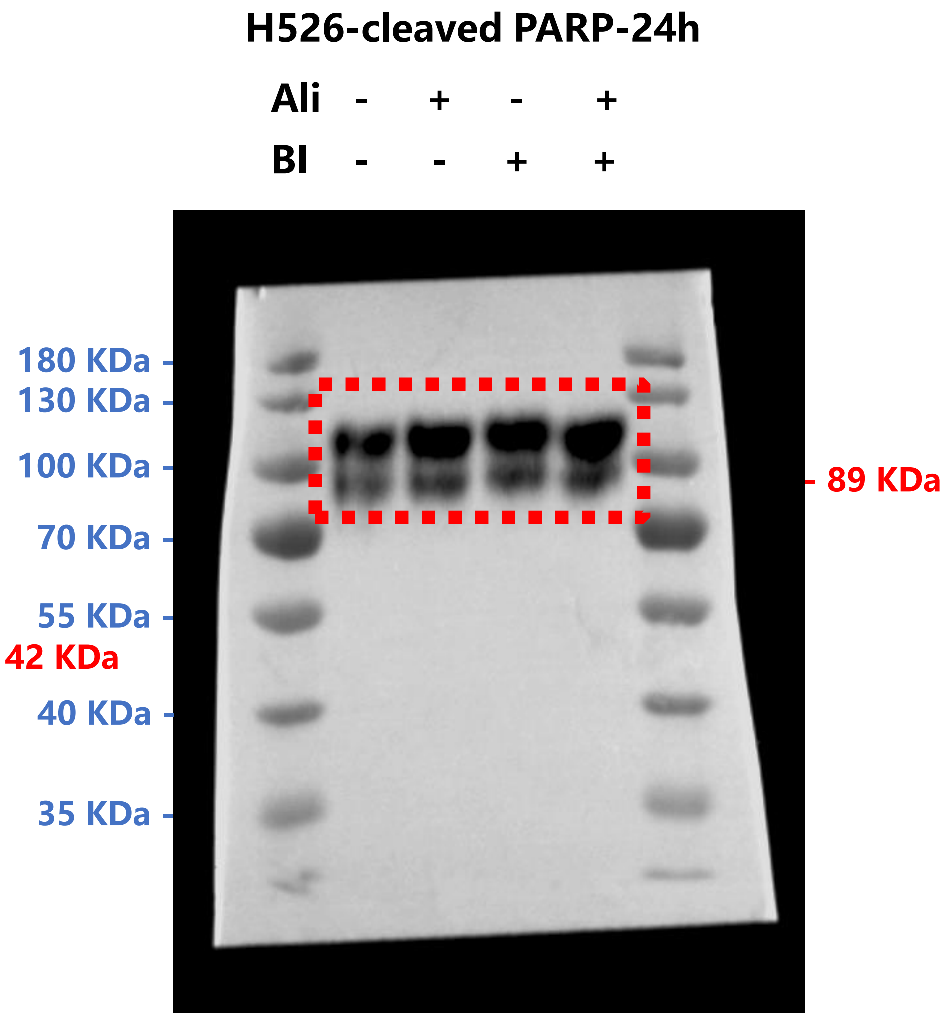

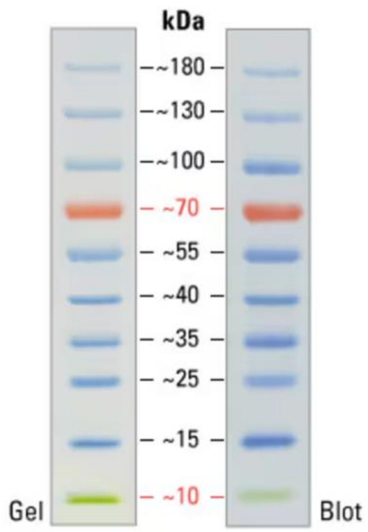

Thermo Scientific™ Marker-26617

■ Original Western Blots of Figure S4-C

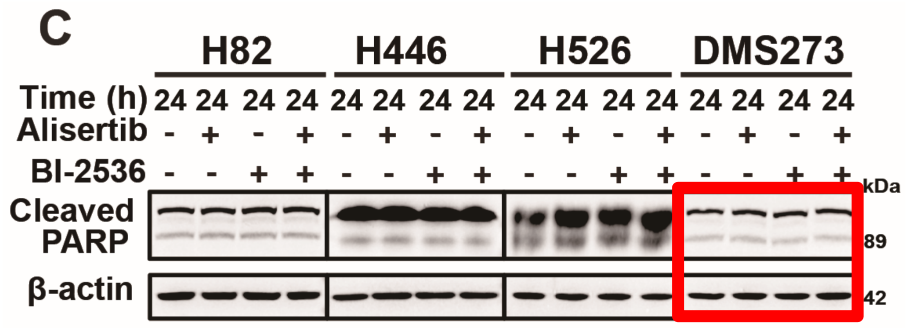

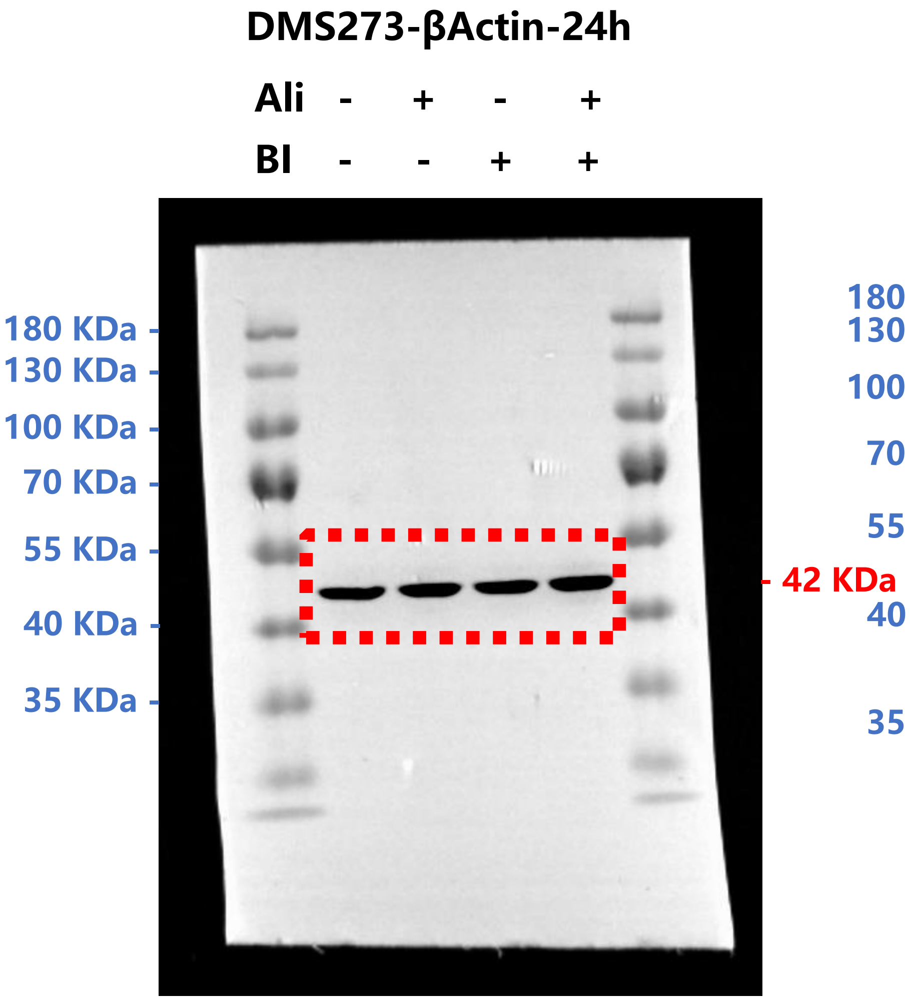

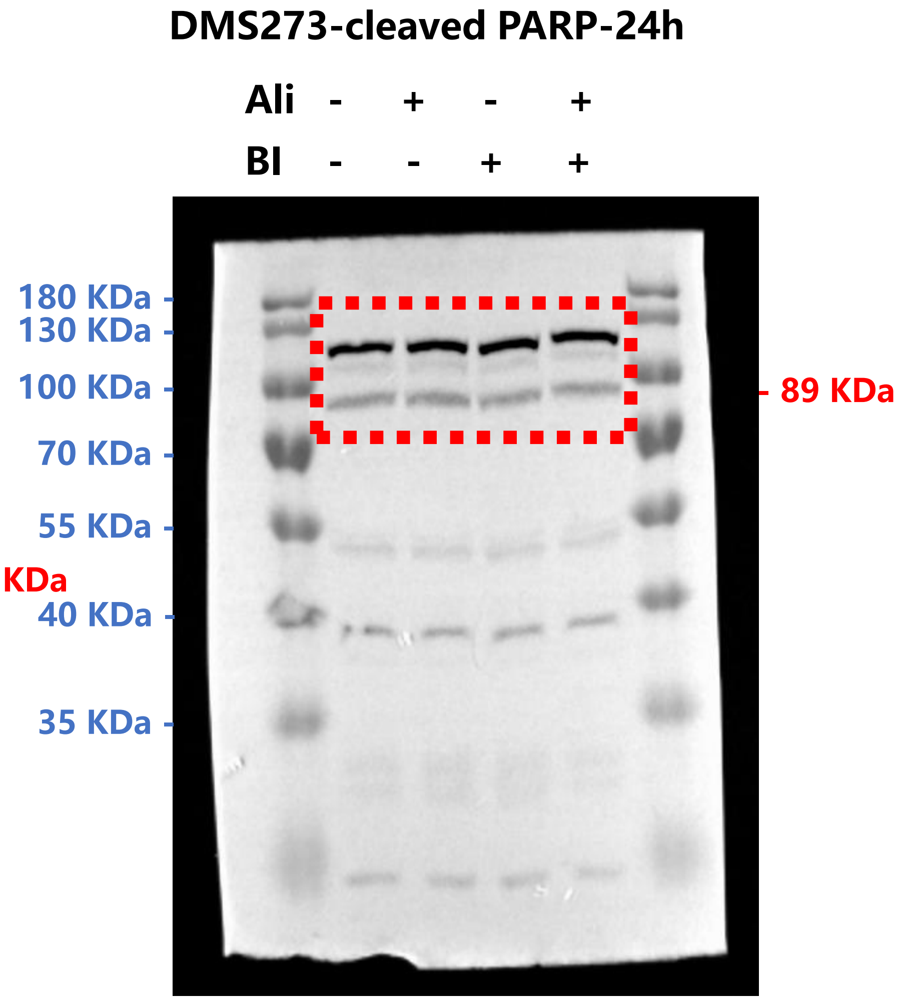

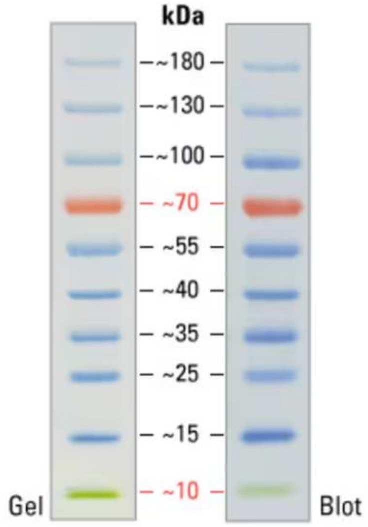

Thermo Scientific™ Marker-26617

■ Original Western Blots of Figure S4-D

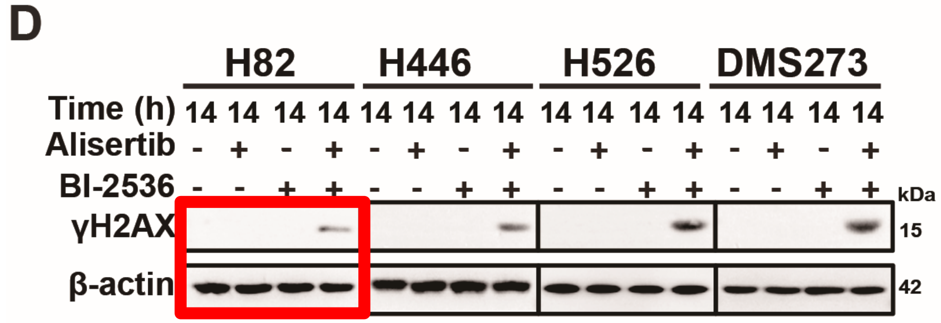

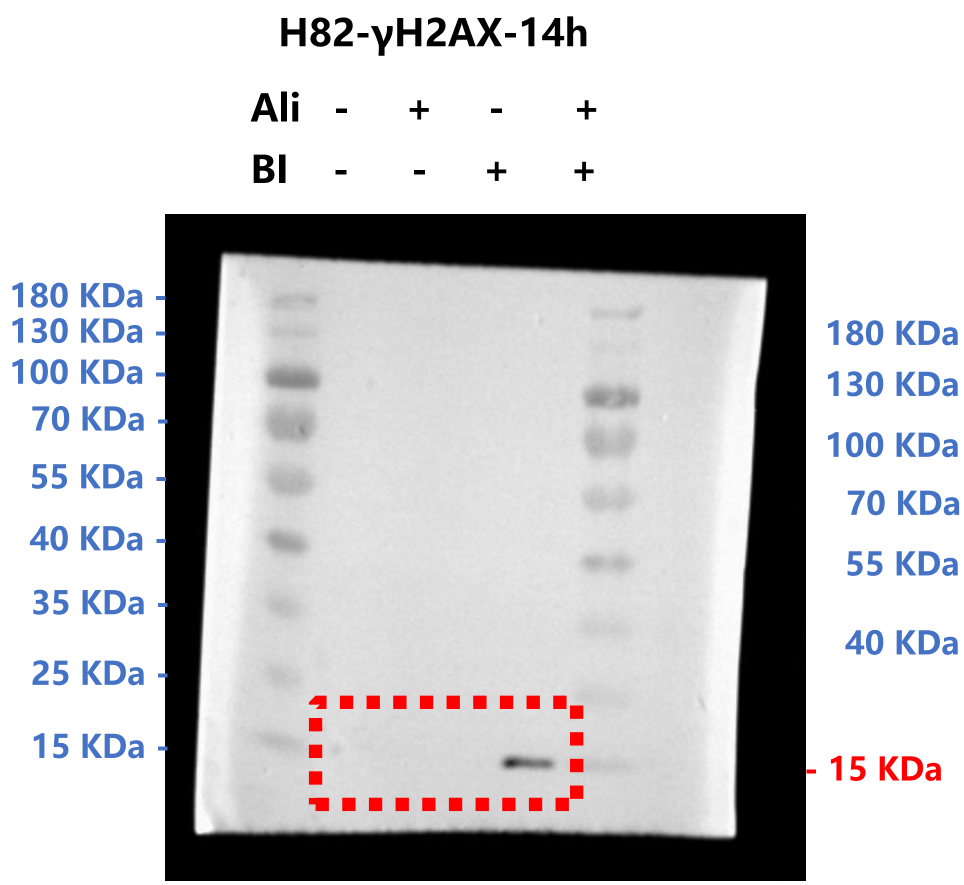

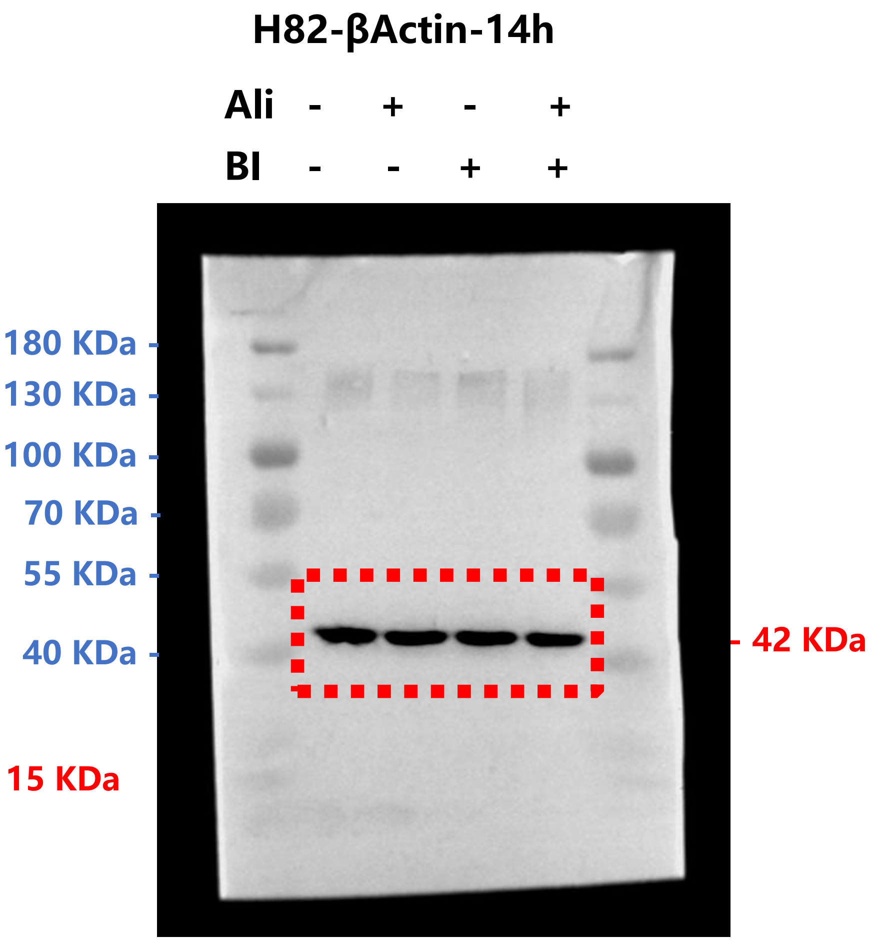

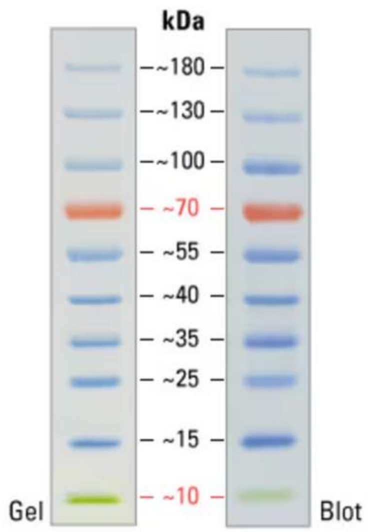

Thermo Scientific™ Marker-26617

■ Original Western Blots of Figure S4-D

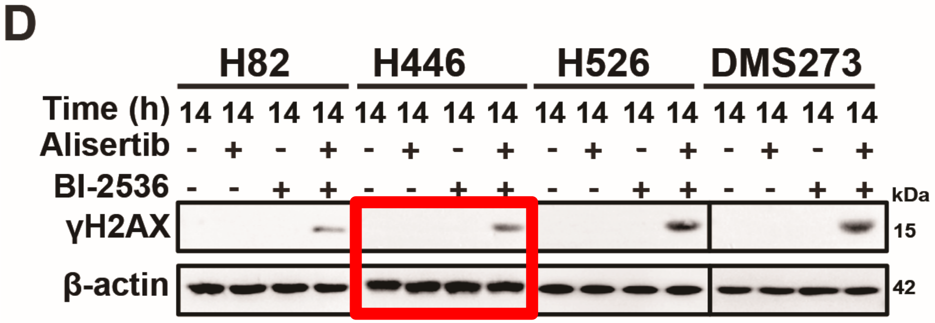

H446-βActin-14h

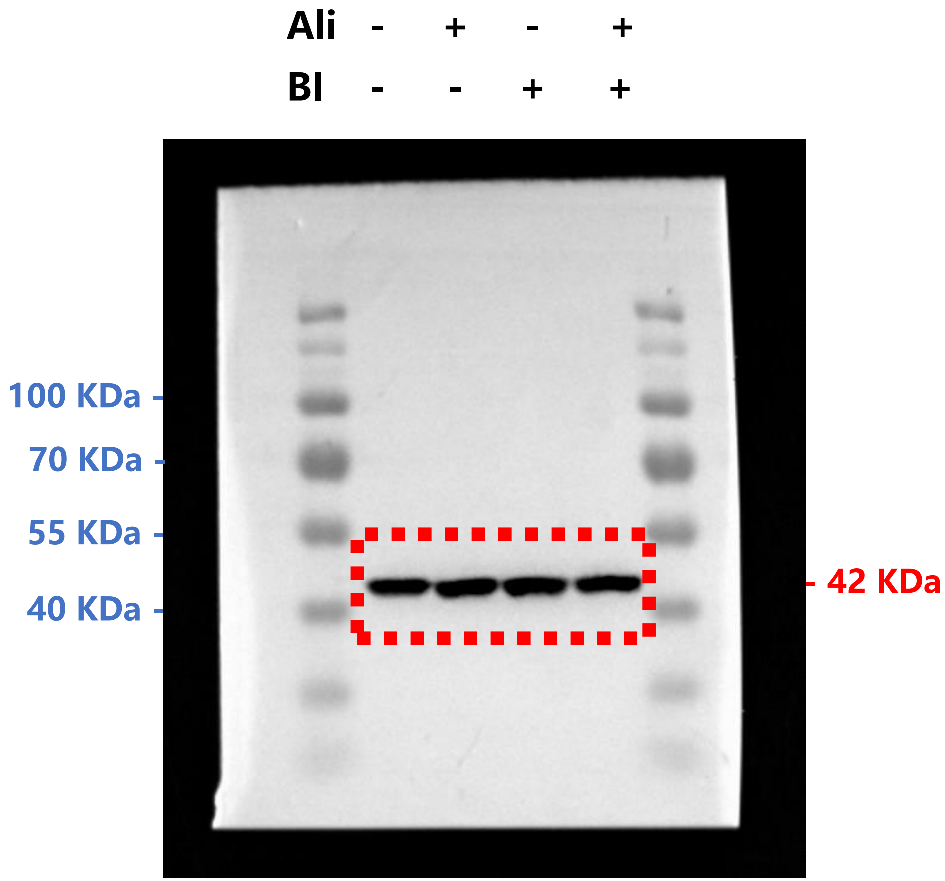

H446-γH2AX-14h

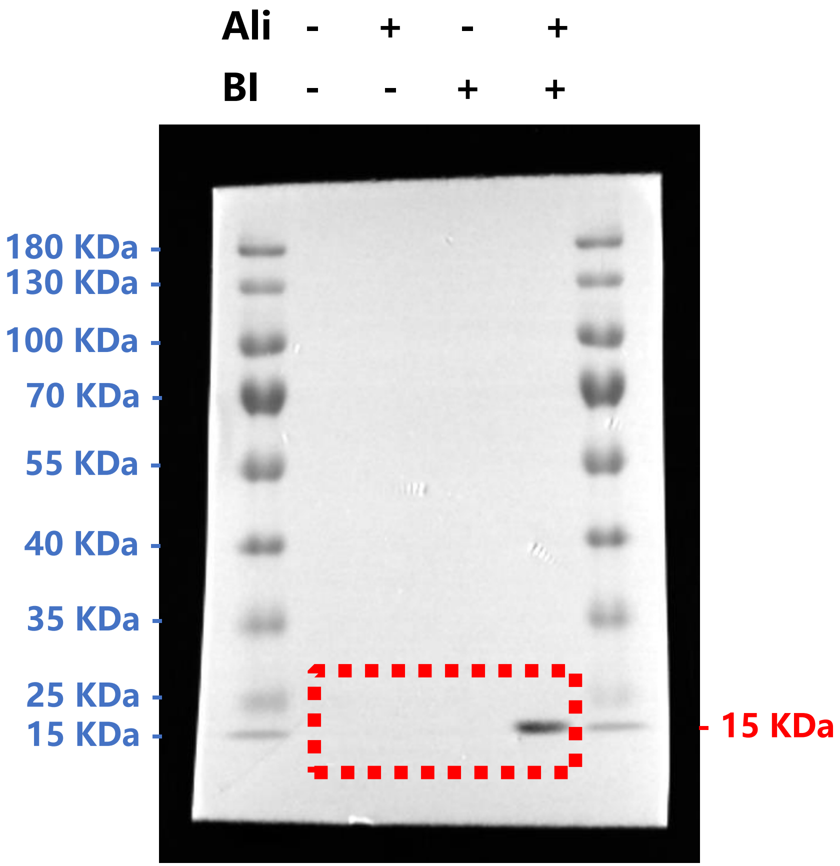

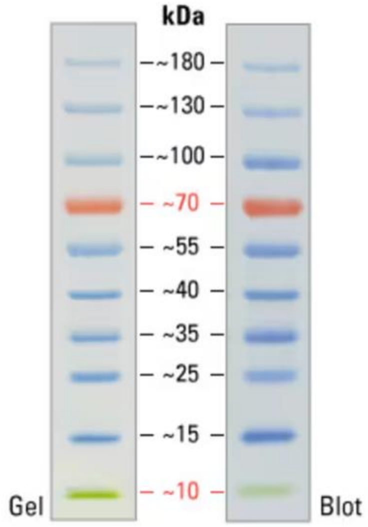

Thermo Scientific™ Marker-26617

■ **Original Western Blots of Figure S4-D**

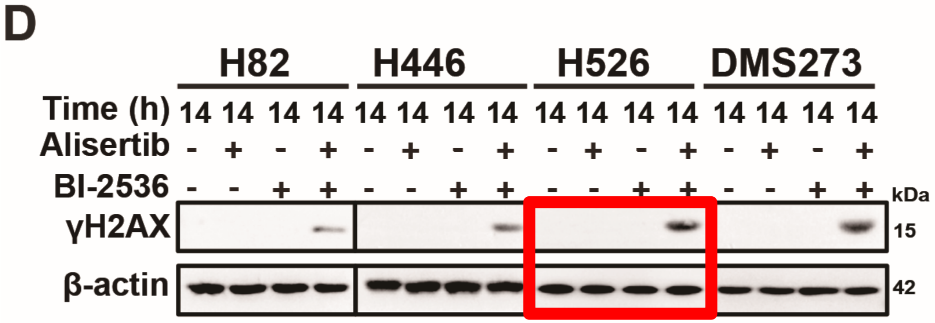

H526-βActin-14h

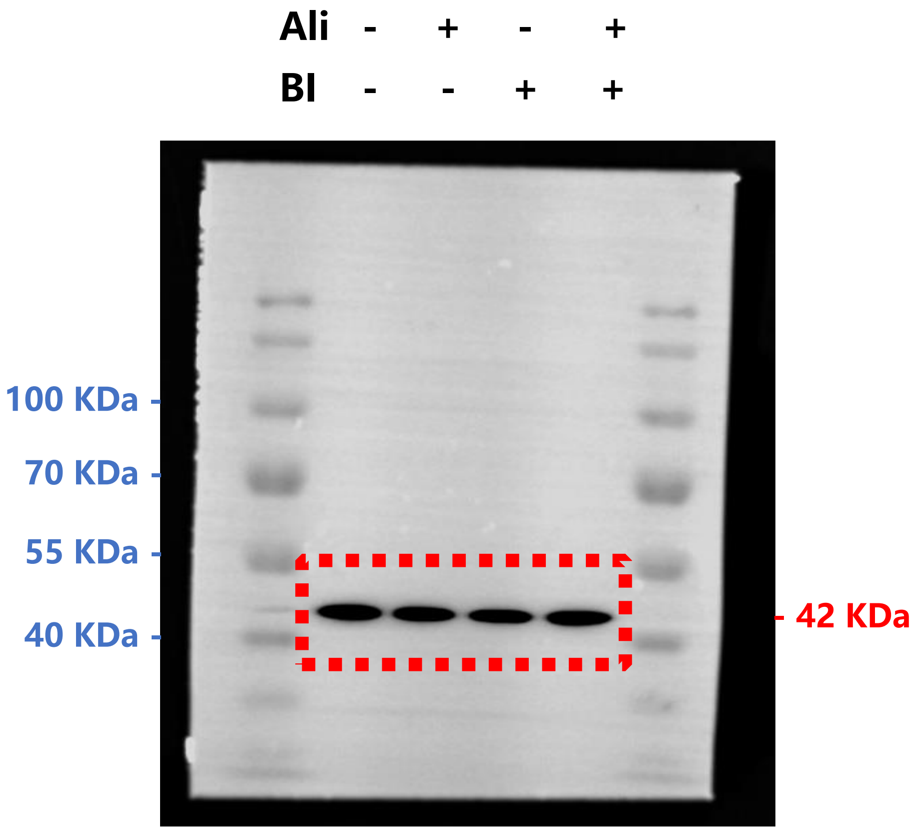

H526-γH2AX-14h

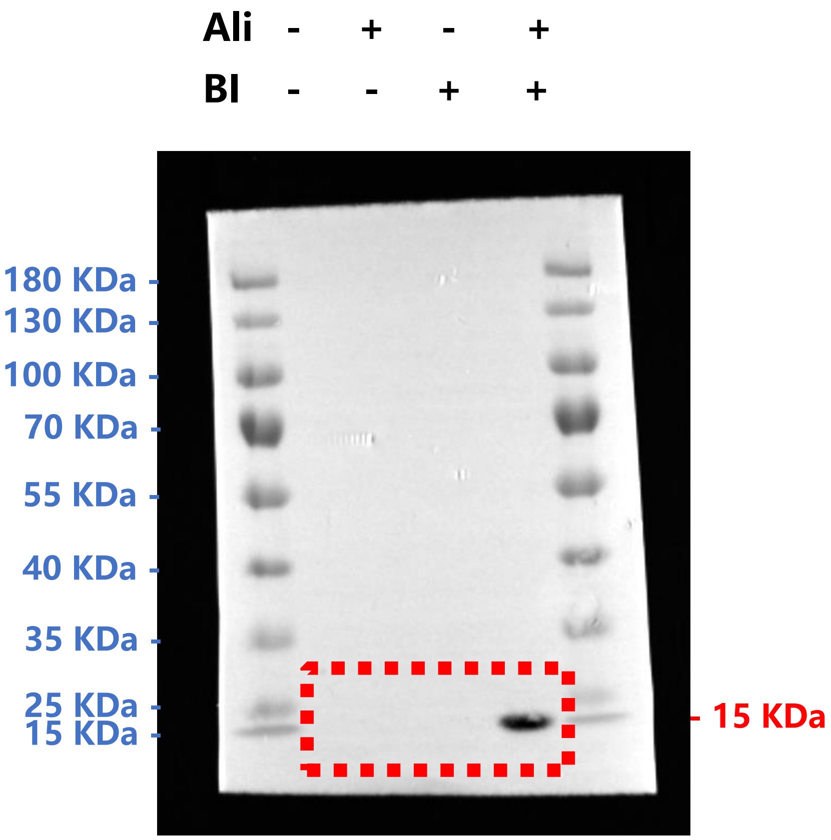

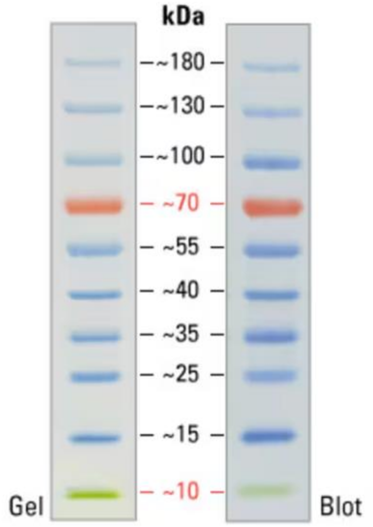

Thermo Scientific™ Marker-26617

■ Original Western Blots of Figure S4-D

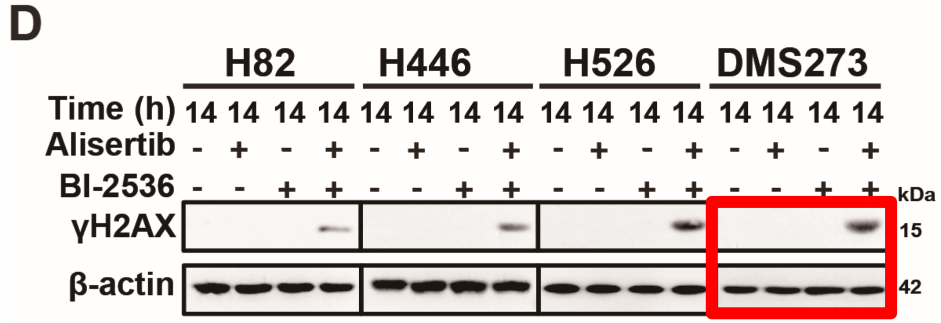

DMS273-βActin-14h

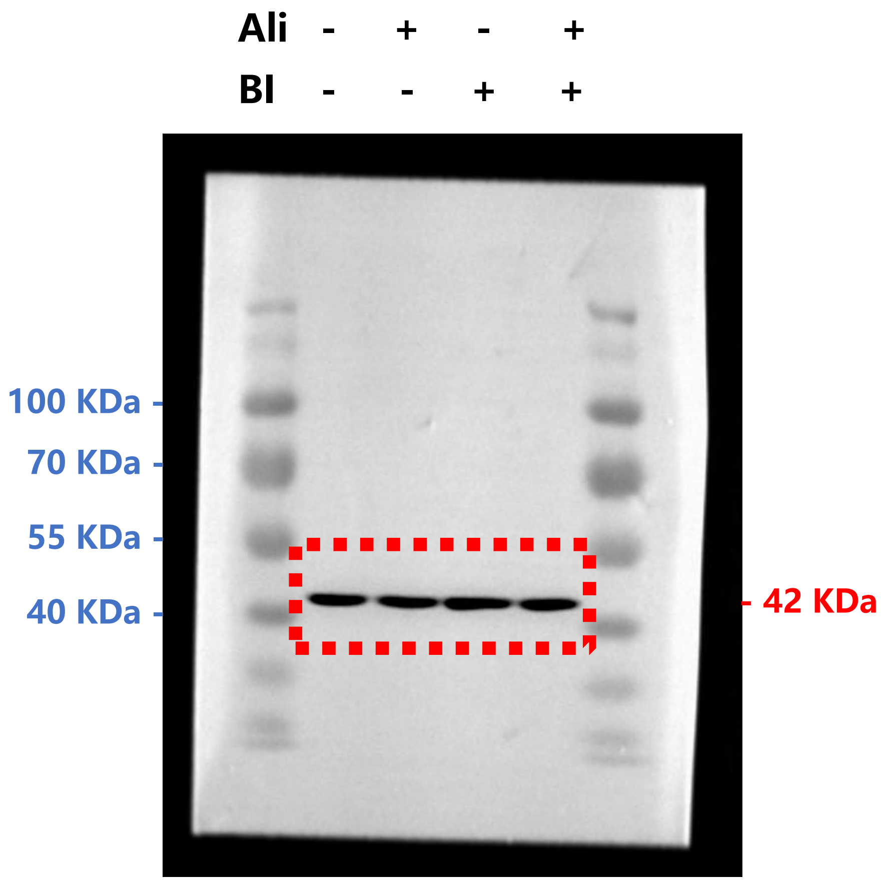

DMS273-γH2AX-14h

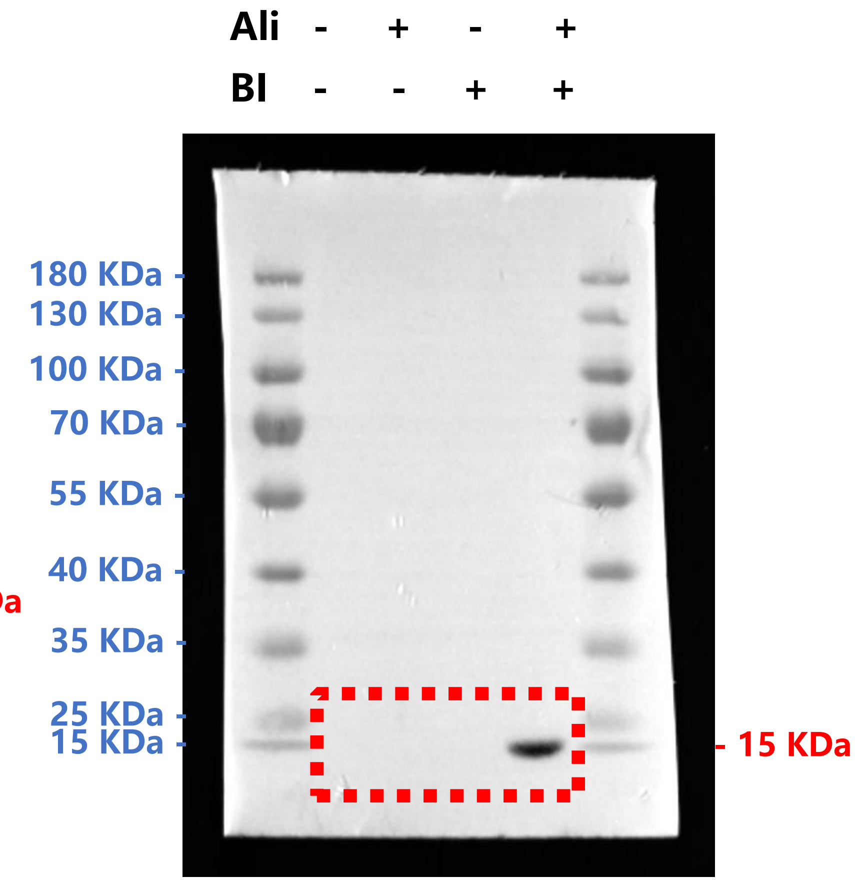

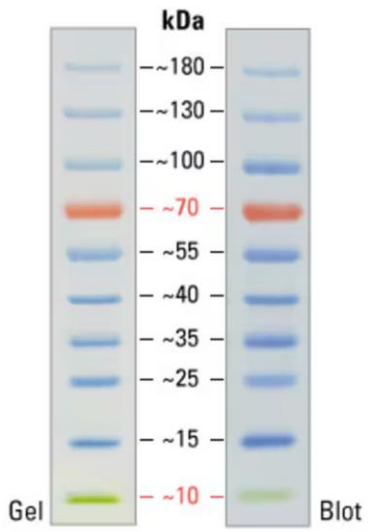

Thermo Scientific™ Marker-26617

■ Original Western Blots of Figure S5-A-24h

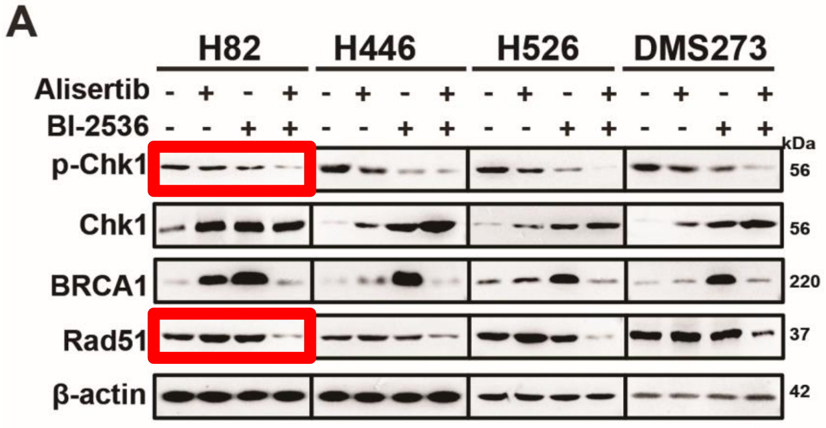

H82-pChk1

Ali - + - +

BI - - + +

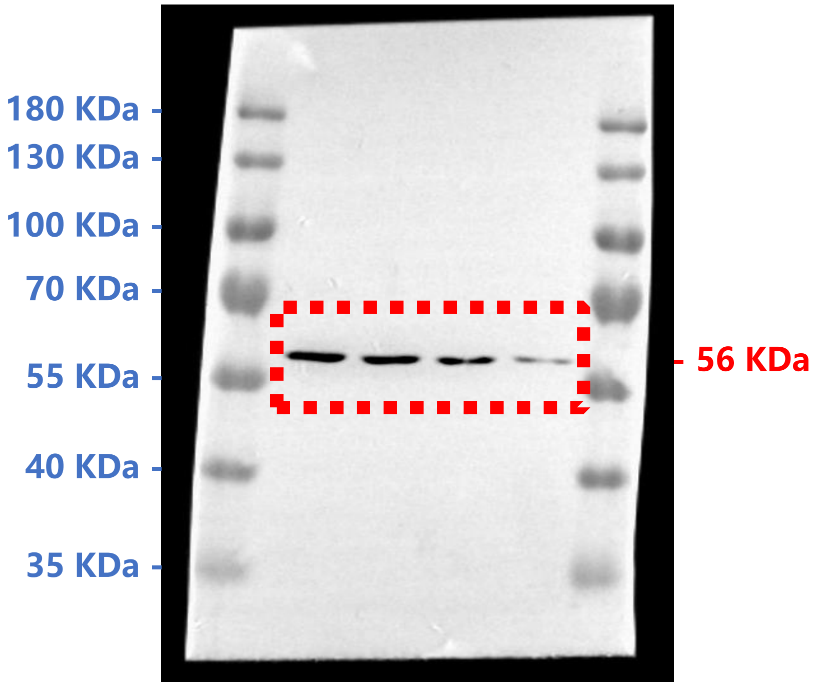

H82-Rad51

Ali - + - +

BI - - + +

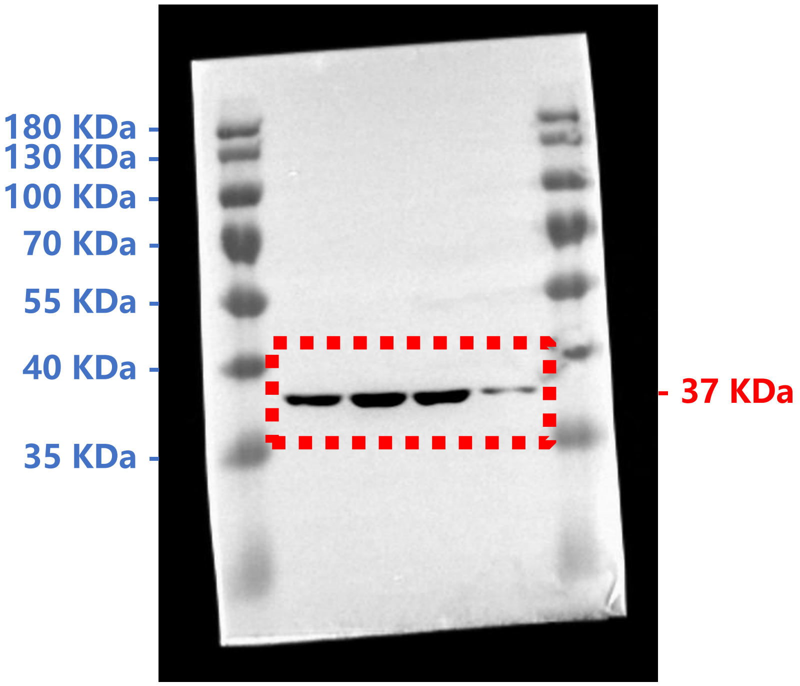

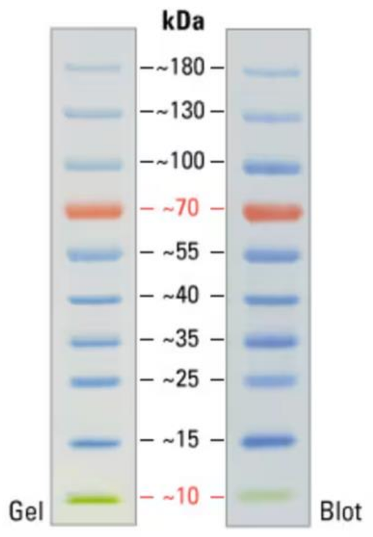

Thermo Scientific™ Marker-26617

■ **Original Western Blots of Figure S5-A-24h**

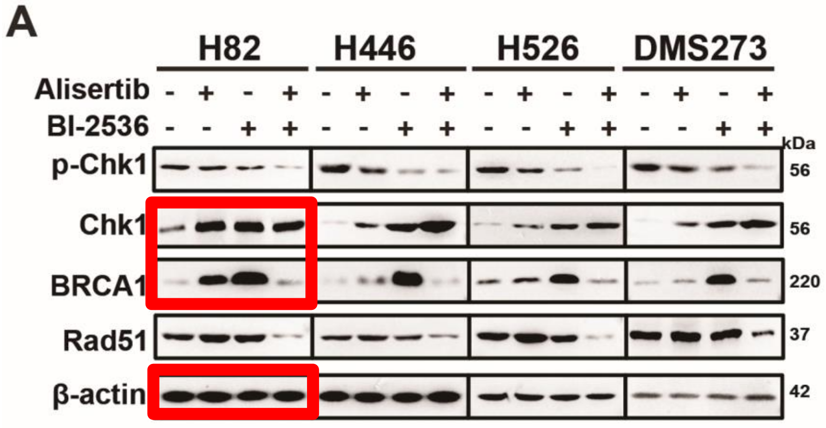

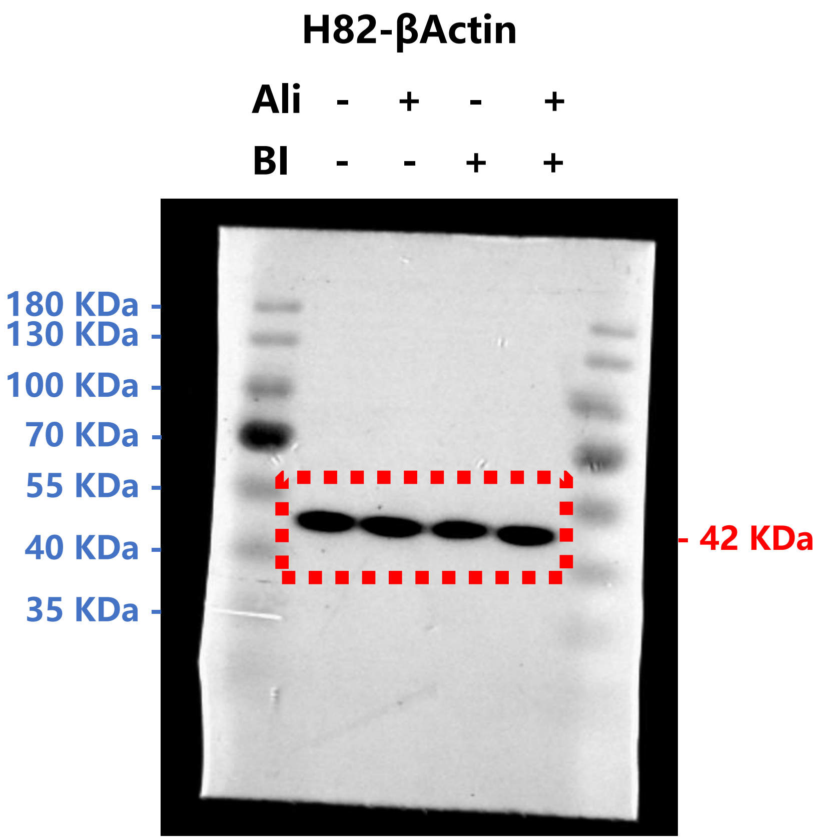

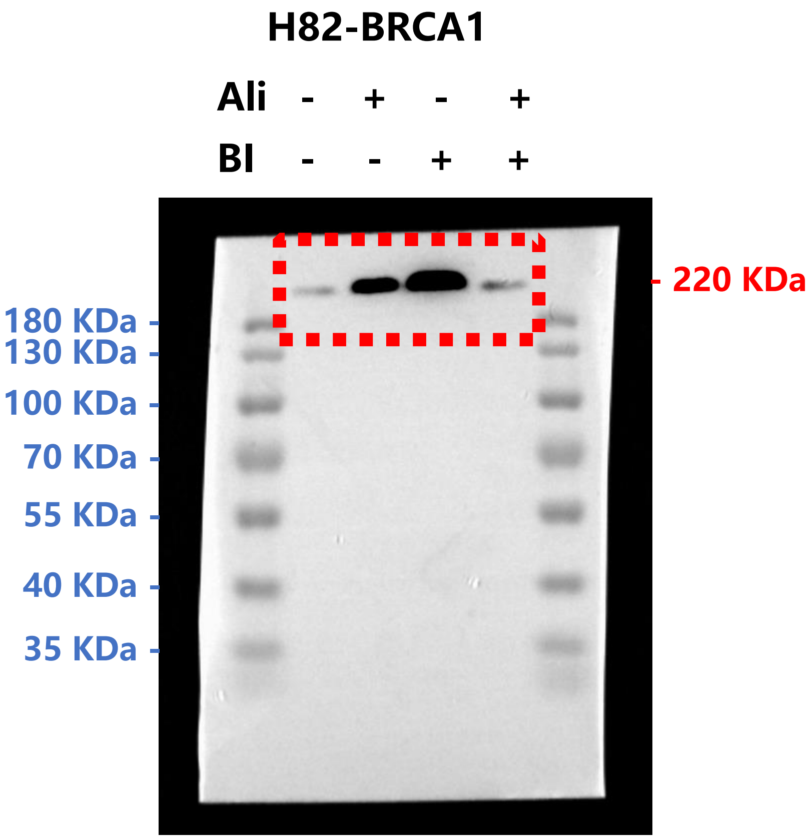

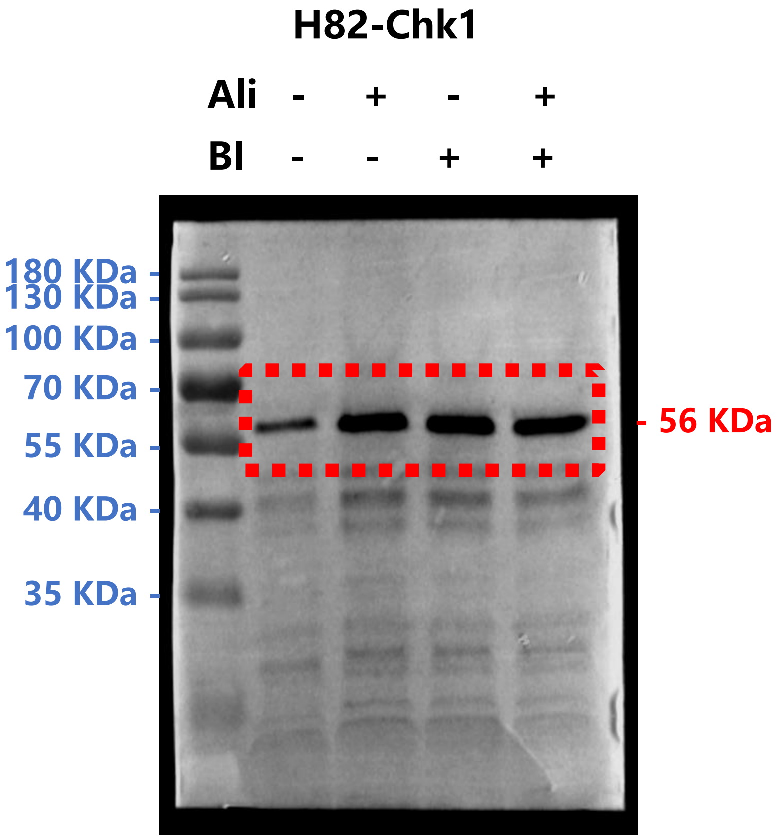

■ Original Western Blots of Figure S5-A-24h

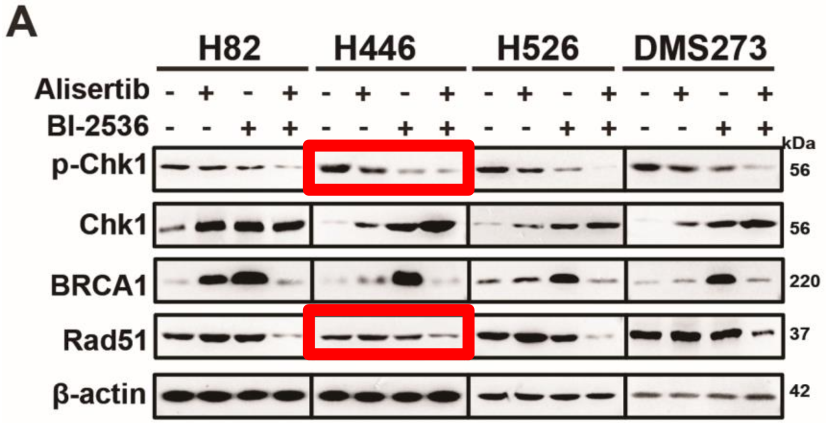

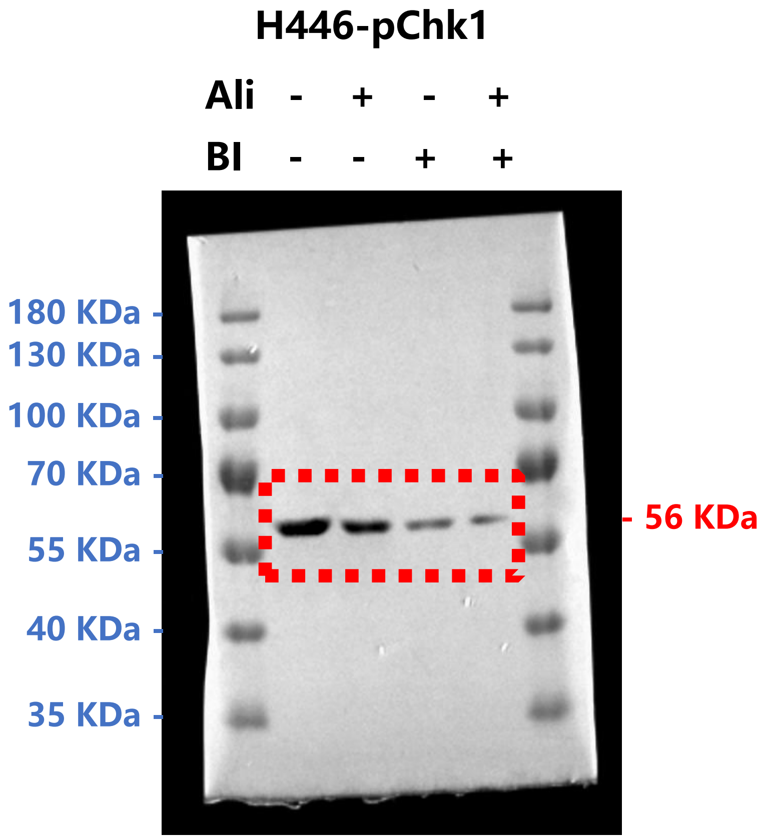

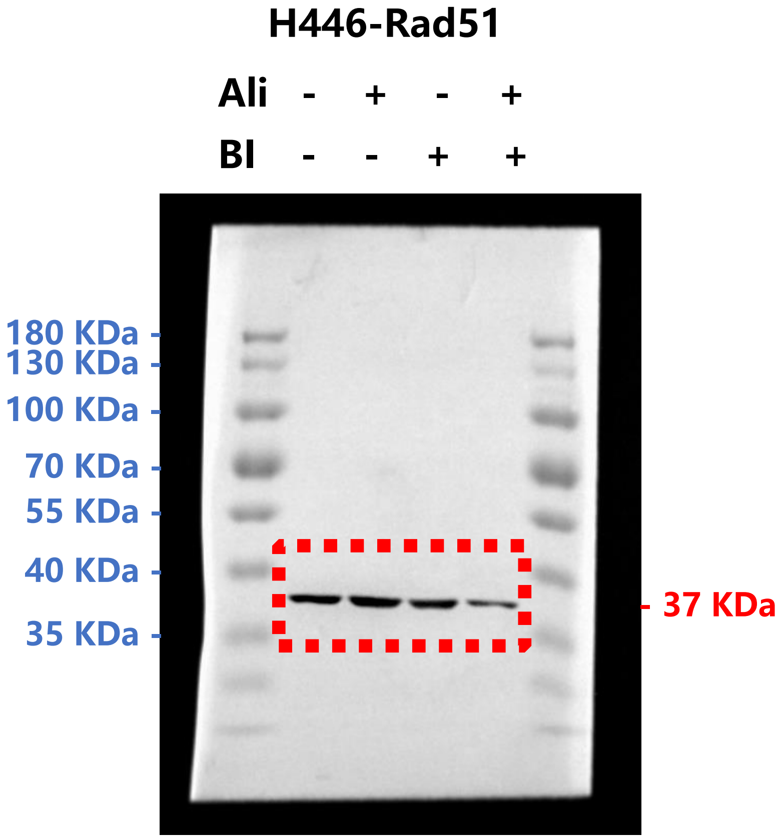

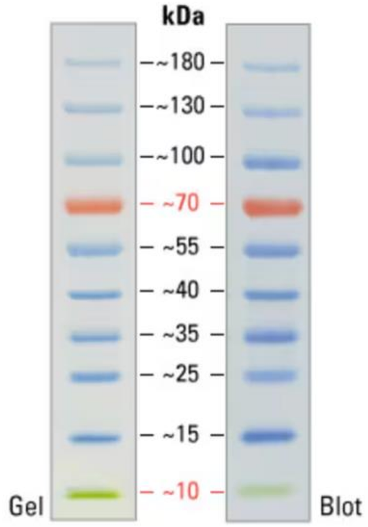

Thermo Scientific™ Marker-26617

■ **Original Western Blots of Figure S5-A-24h**

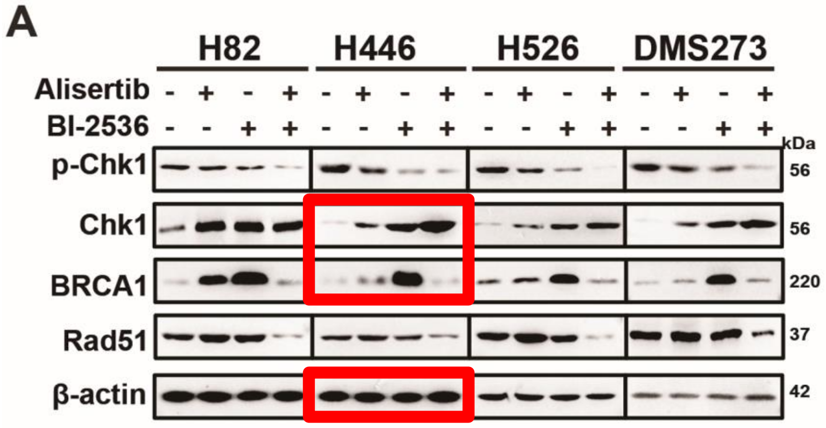

H446-βActin

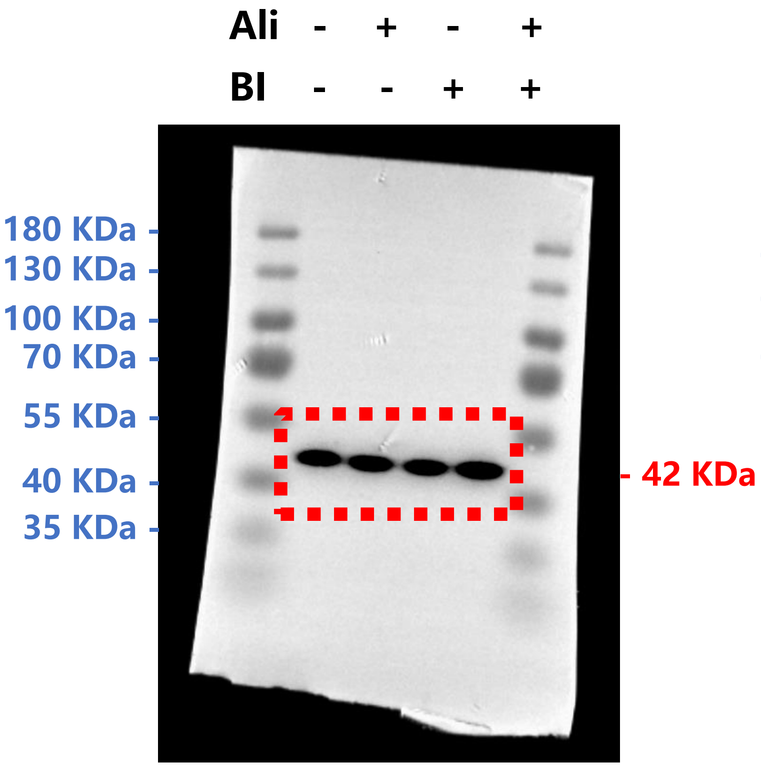

H446-Chk1

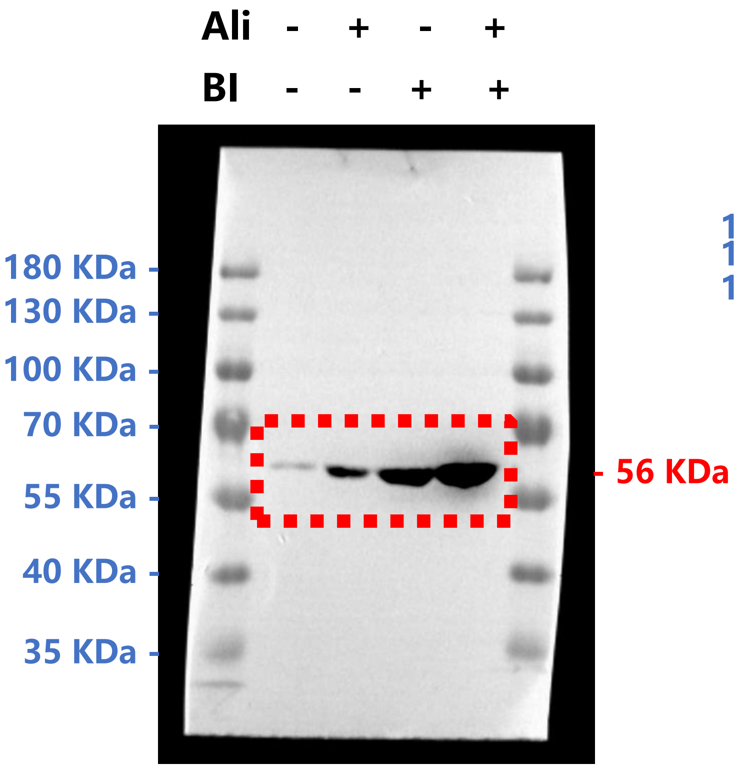

H446-BRCA1

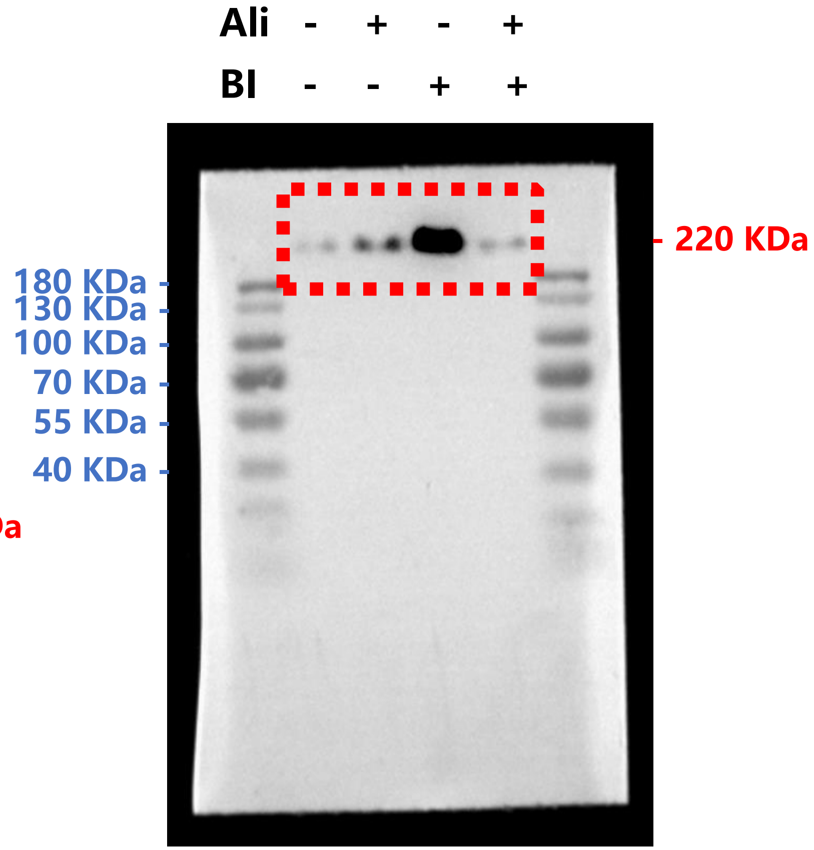

■ Original Western Blots of Figure S5-A-24h

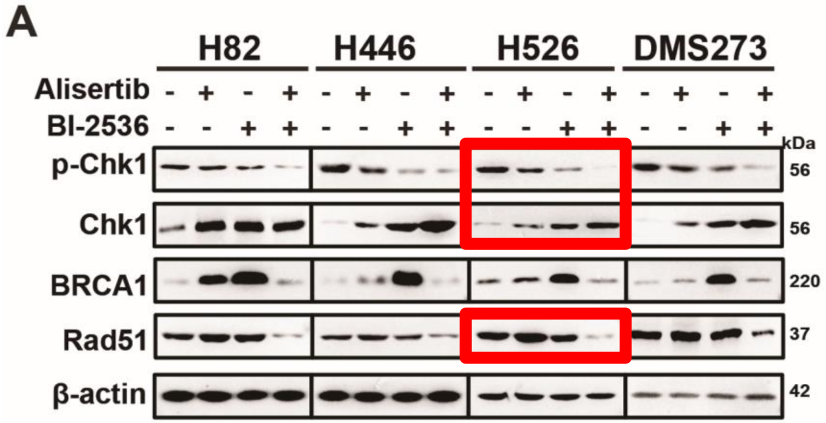

H526-pChk1

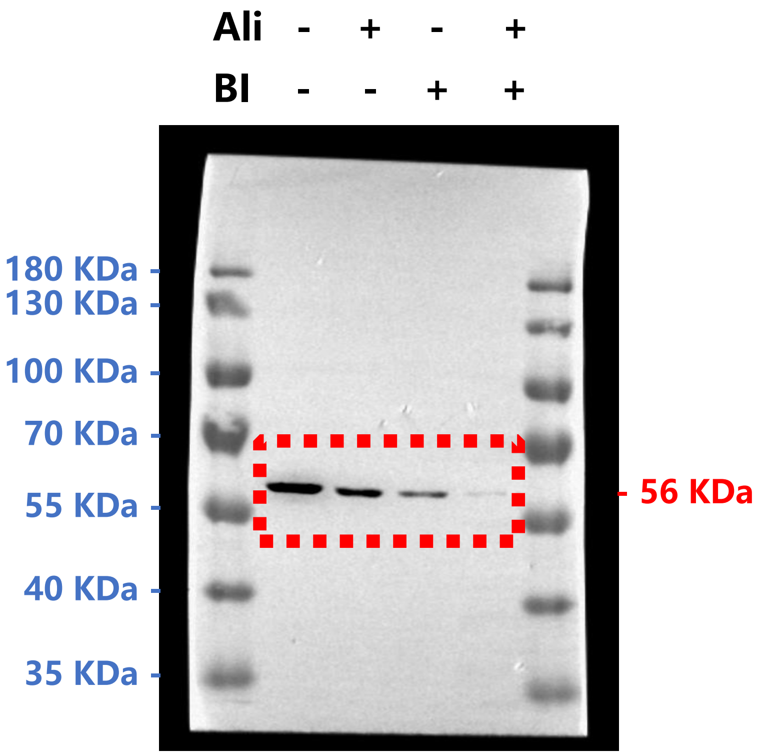

H526-Chk1

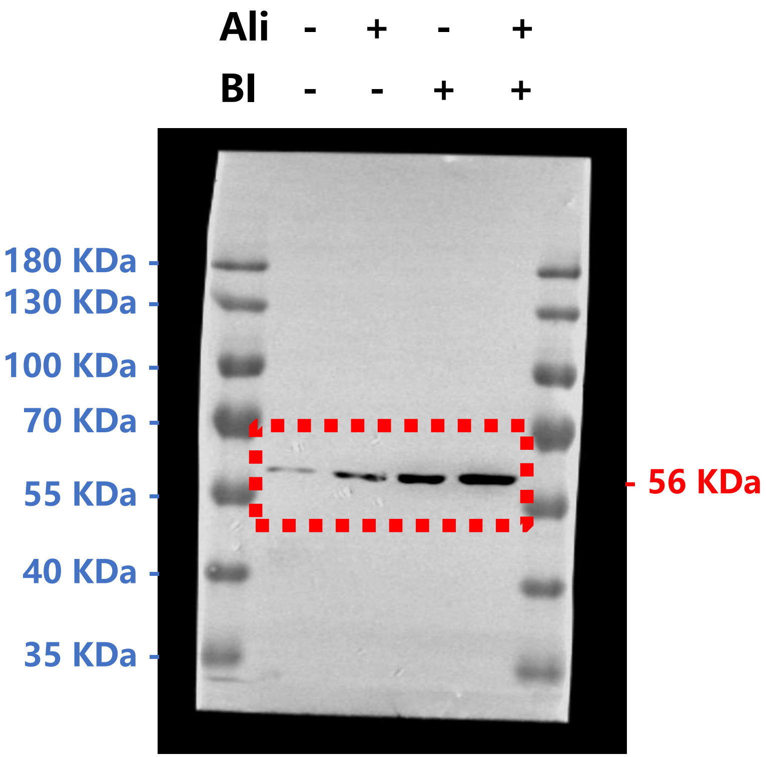

H526-Rad51

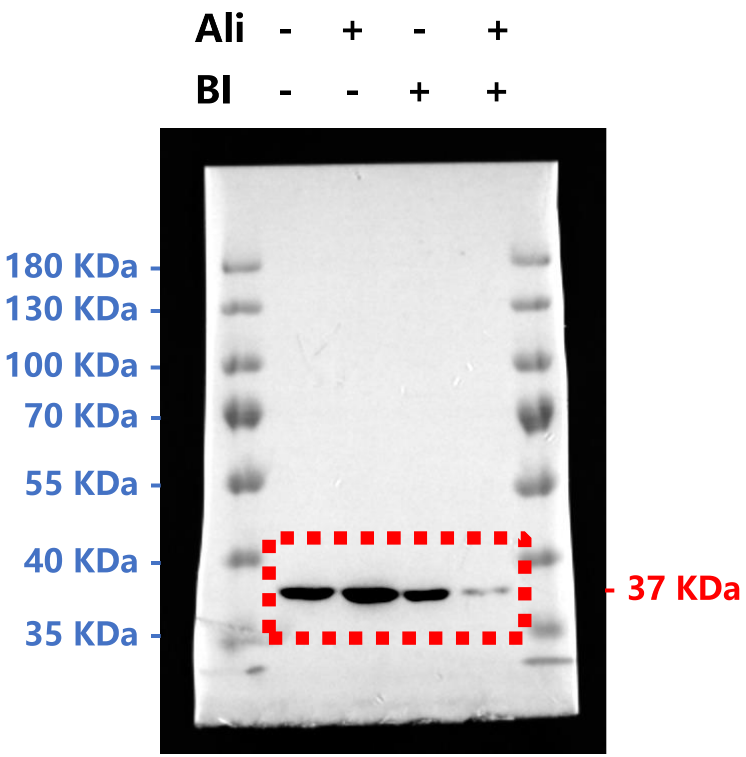

■ **Original Western Blots of Figure S5-A-24h**

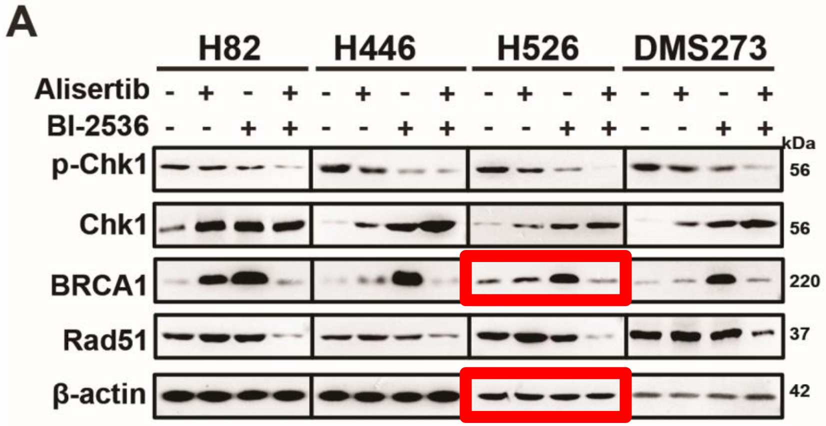

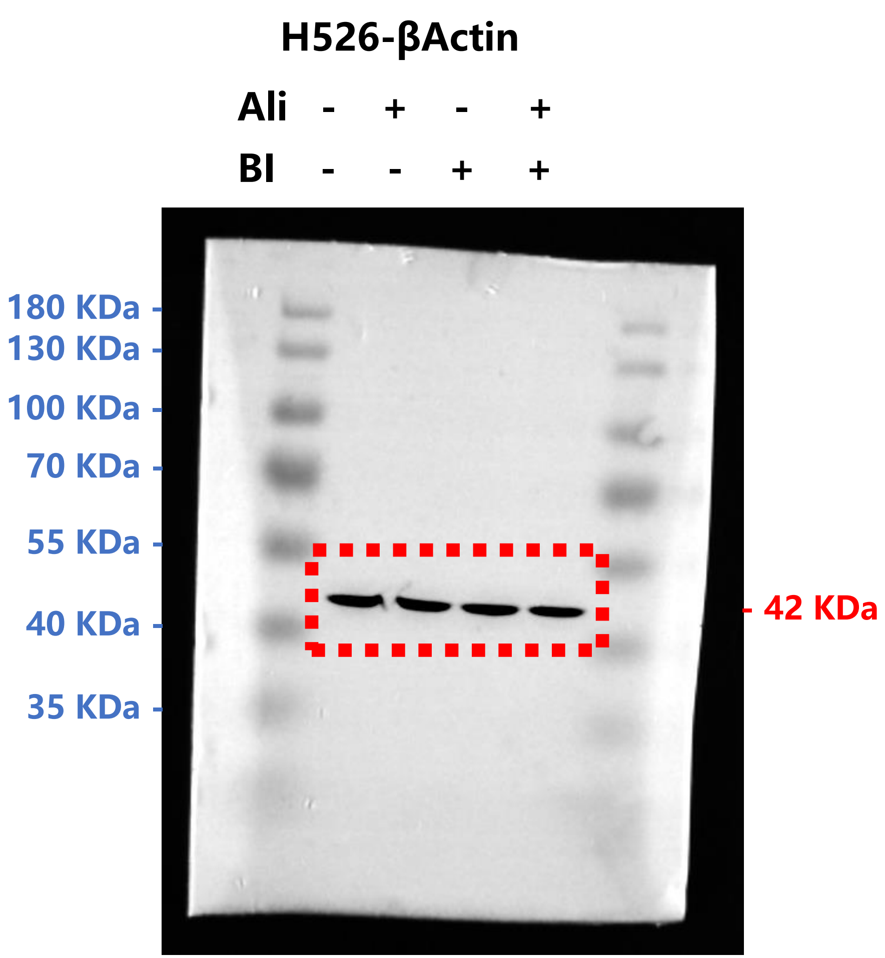

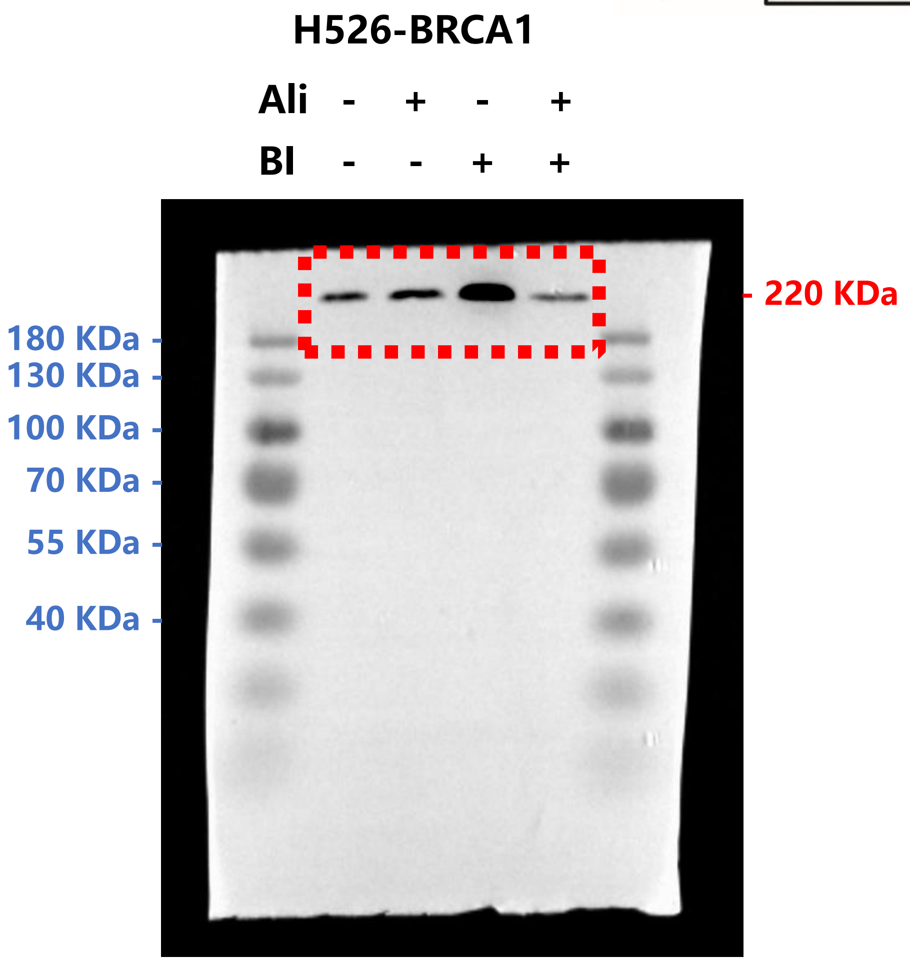

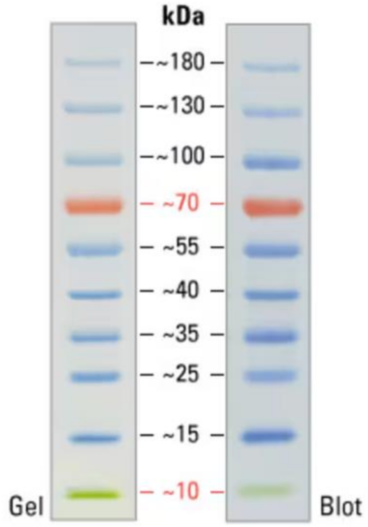

Thermo Scientific™ Marker-26617

■ Original Western Blots of Figure S5-A-24h

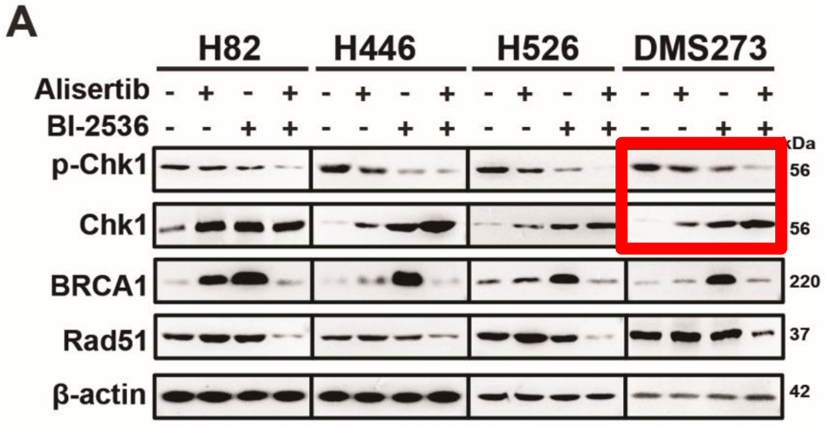

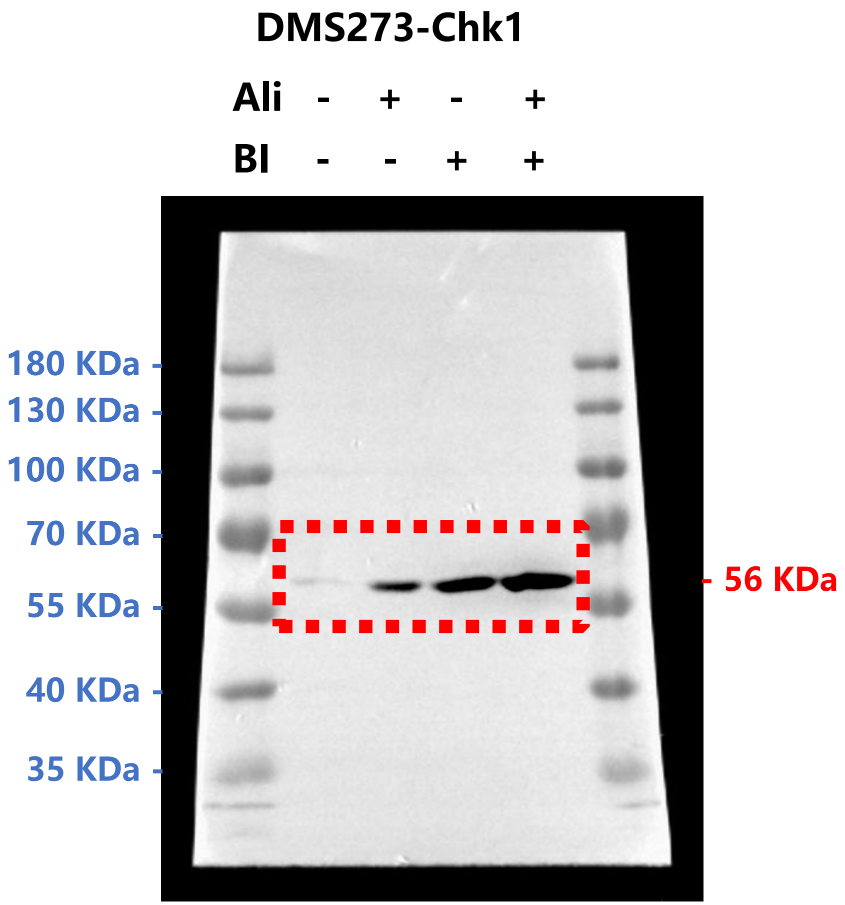

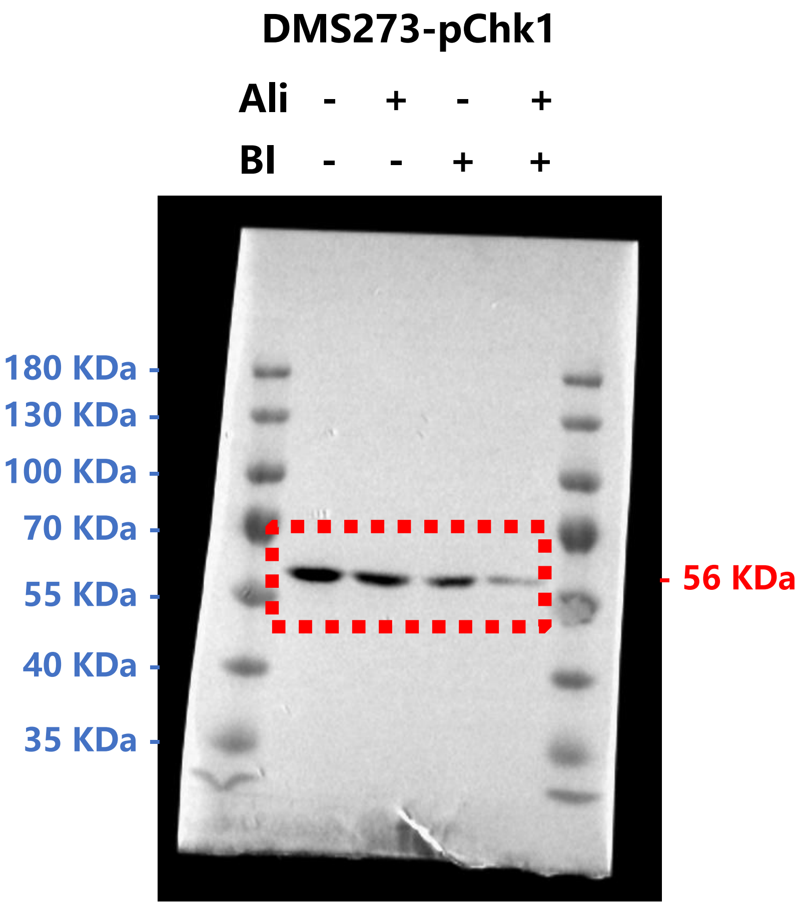

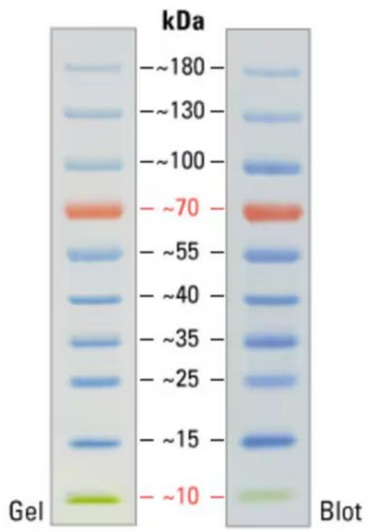

Thermo Scientific™ Marker-26617

■ **Original Western Blots of Figure S5-A-24h**

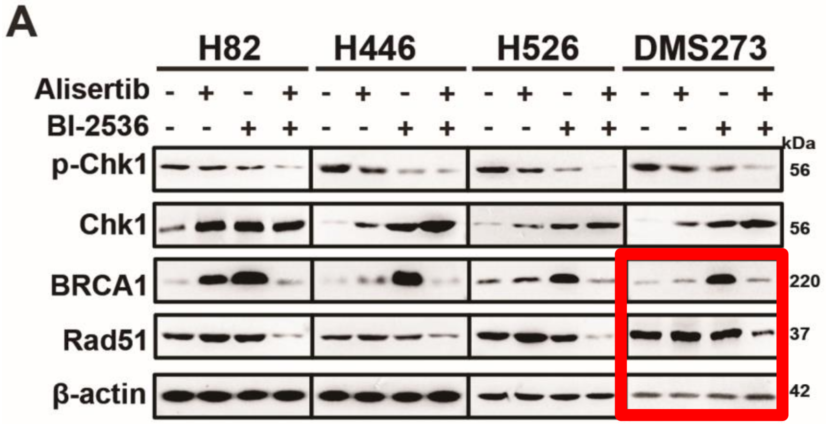

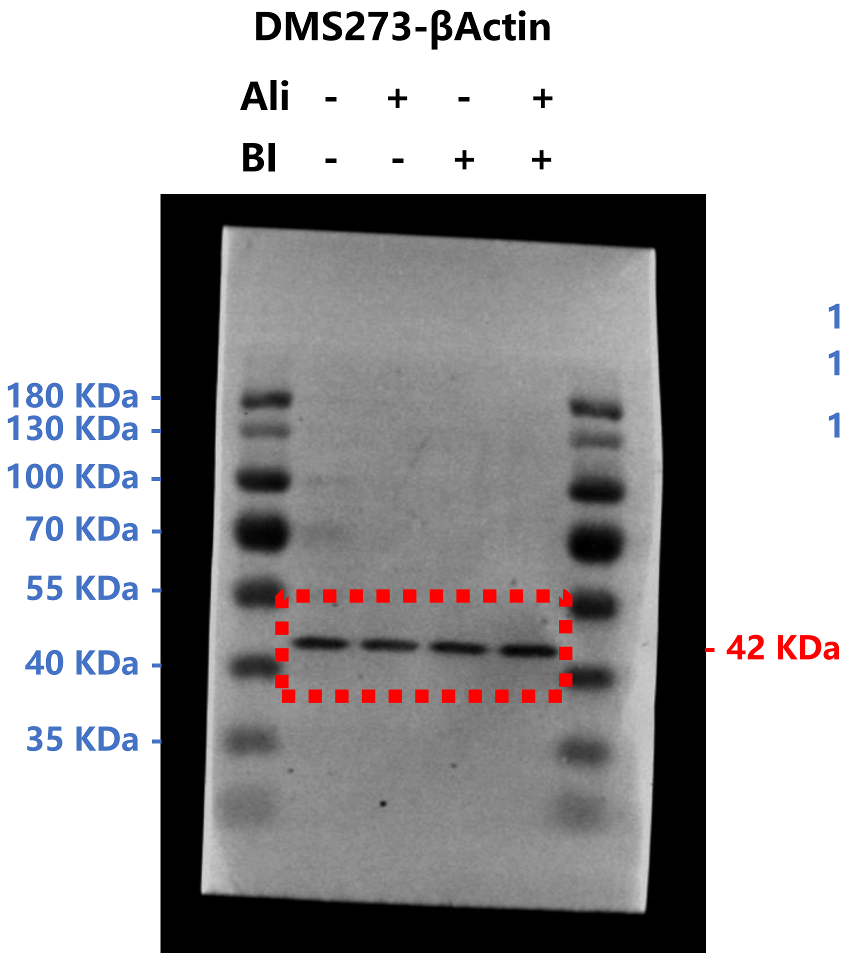

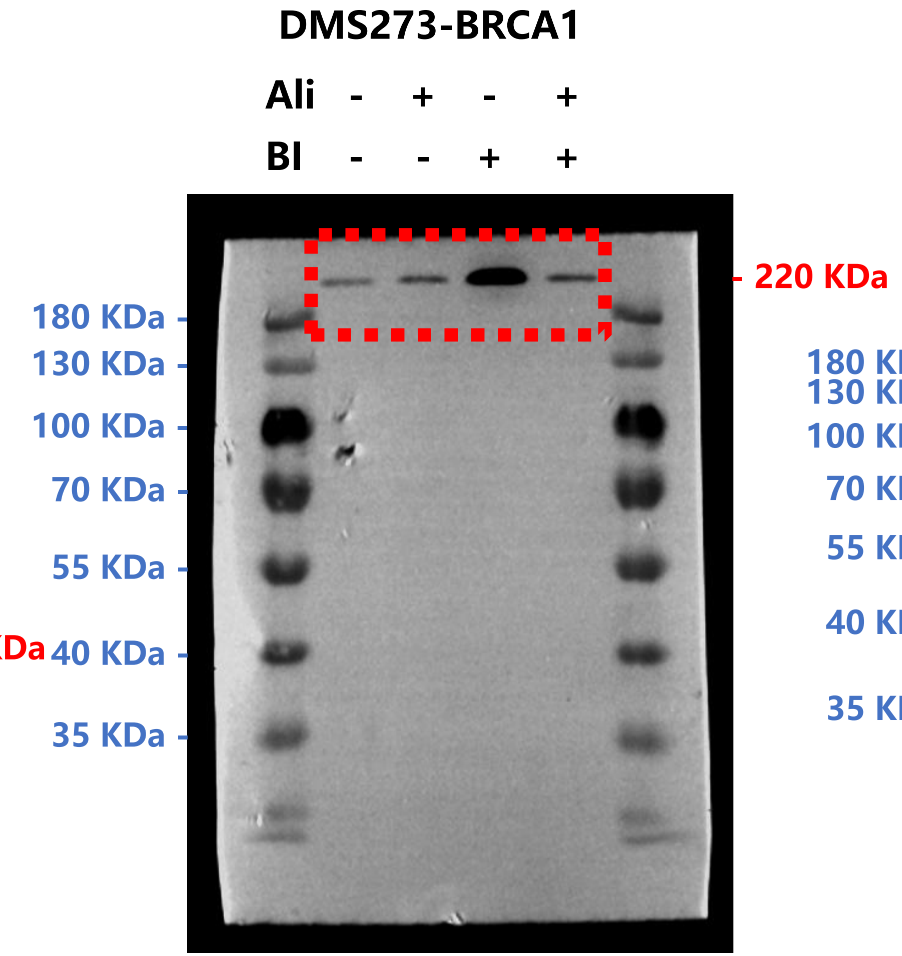

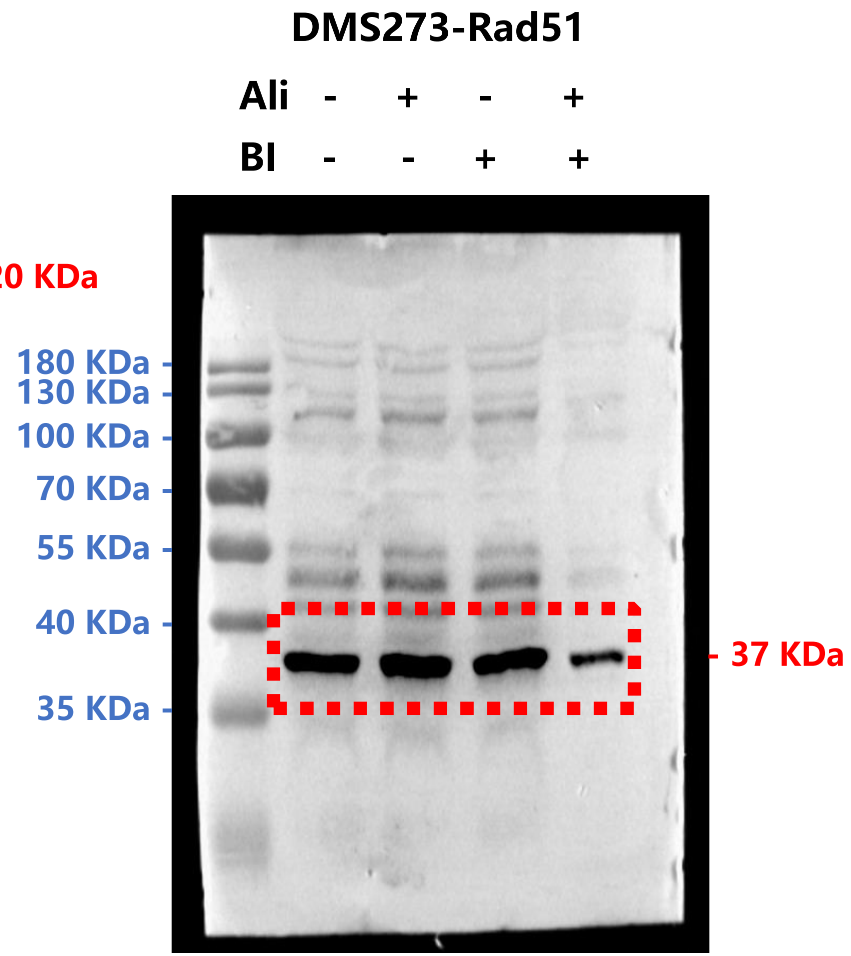

■ Original Western Blots of Figure S5-C

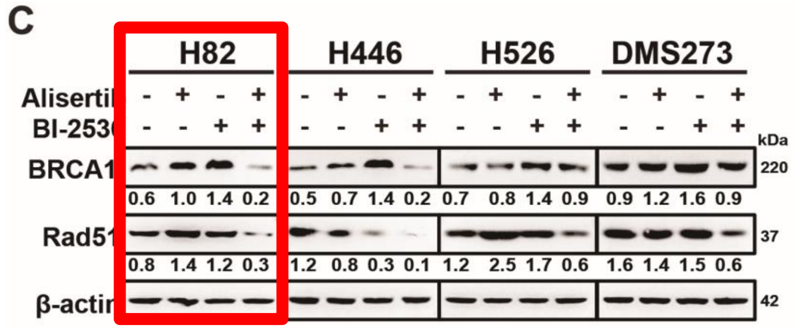

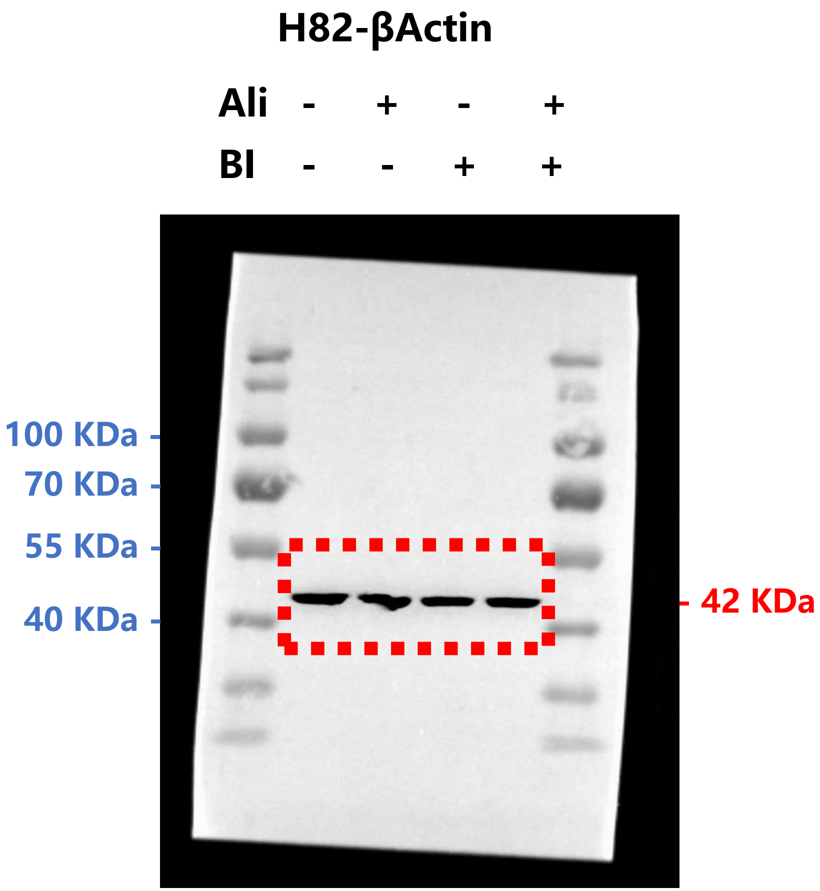

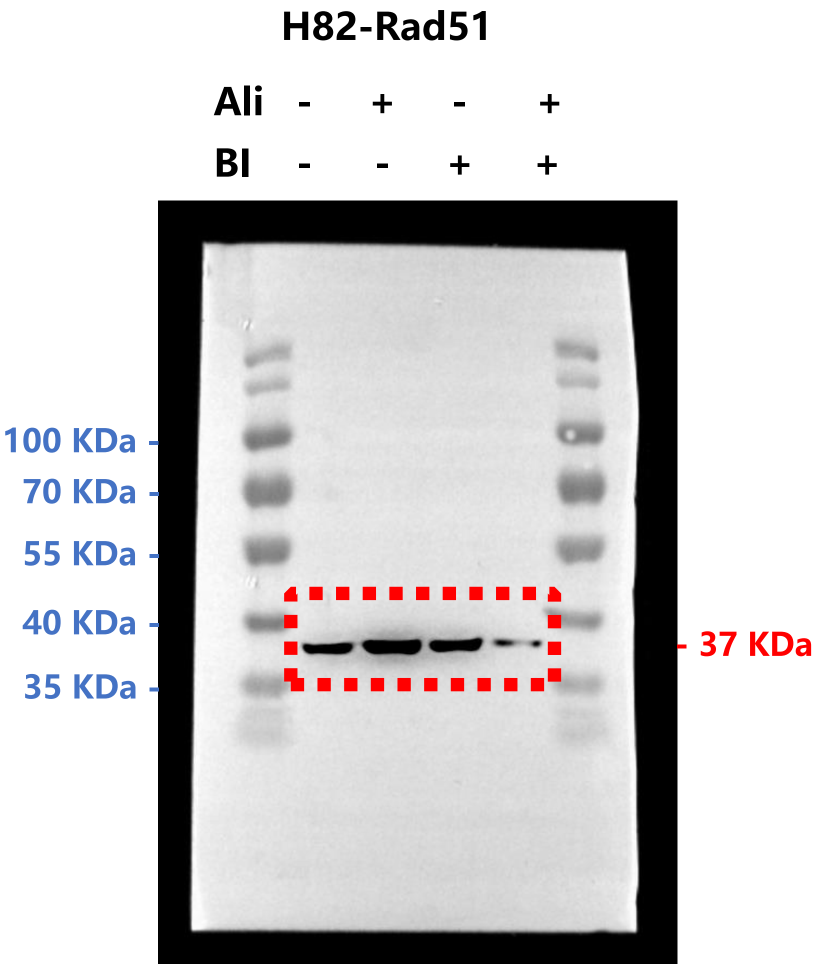

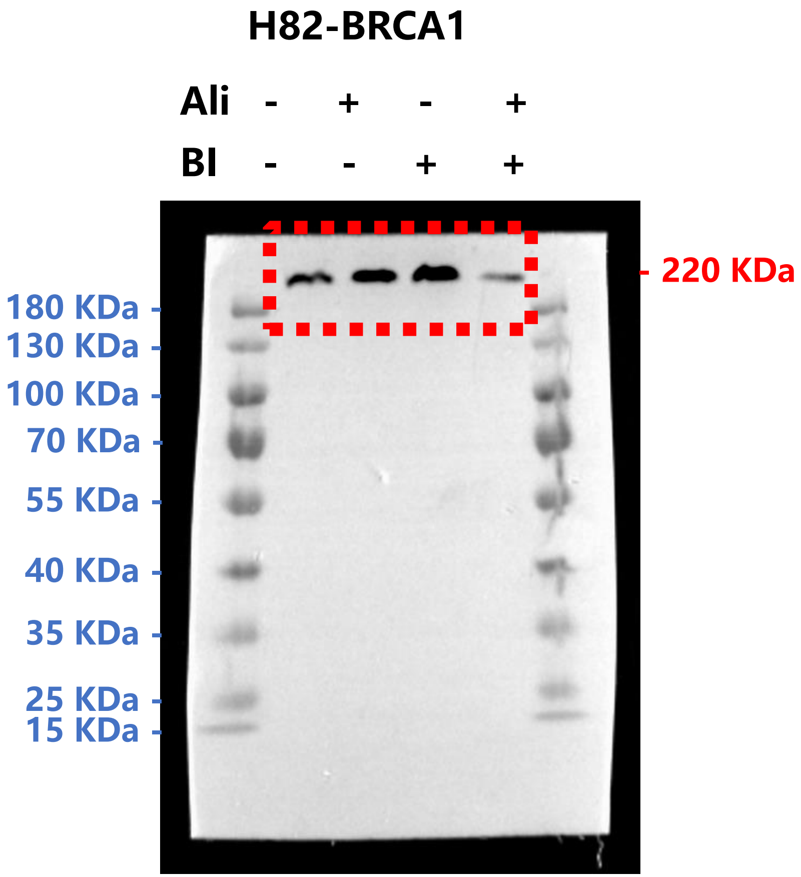

■ Original Western Blots of Figure S5-C

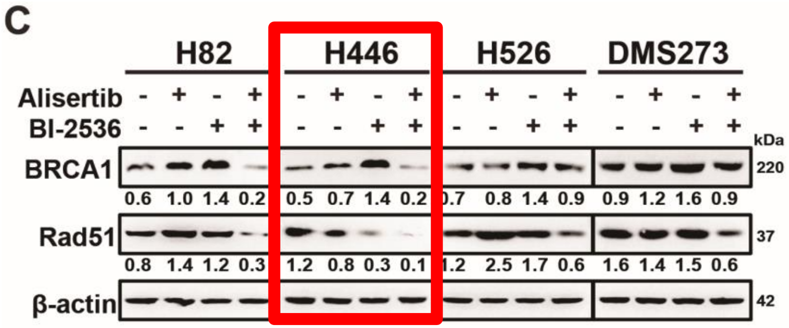

H446-βActin

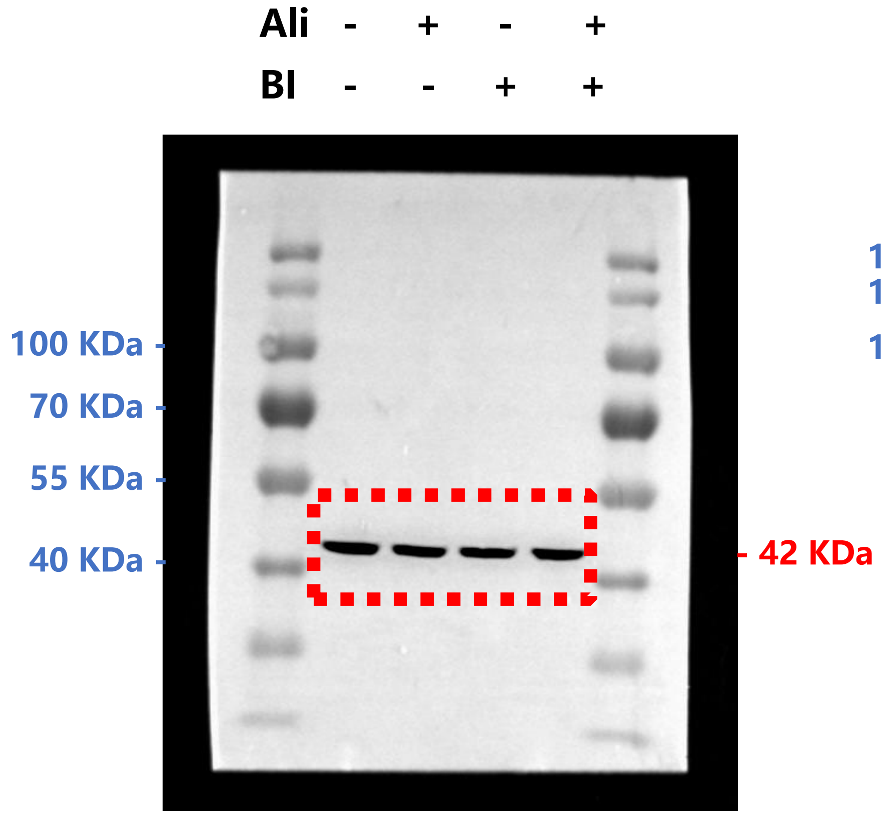

H446-BRCA1

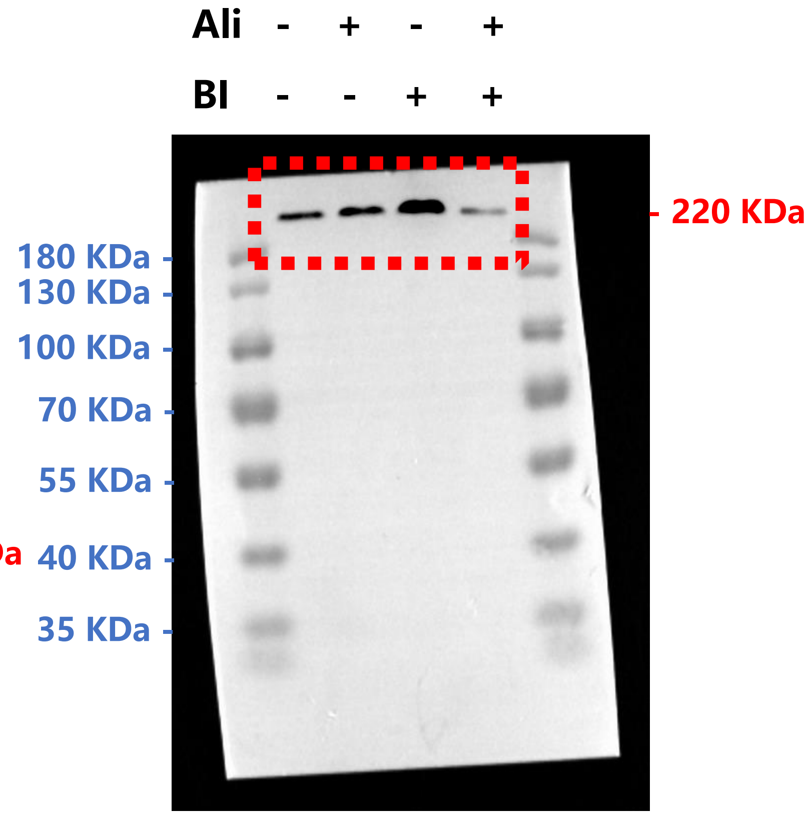

H446-Rad51

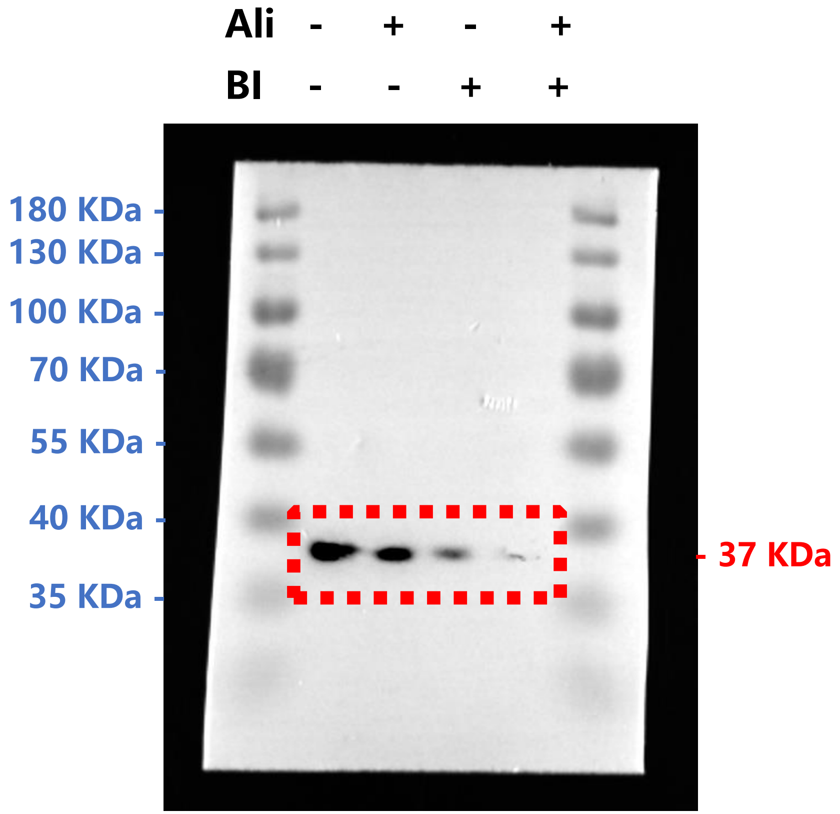

■ Original Western Blots of Figure S5-C

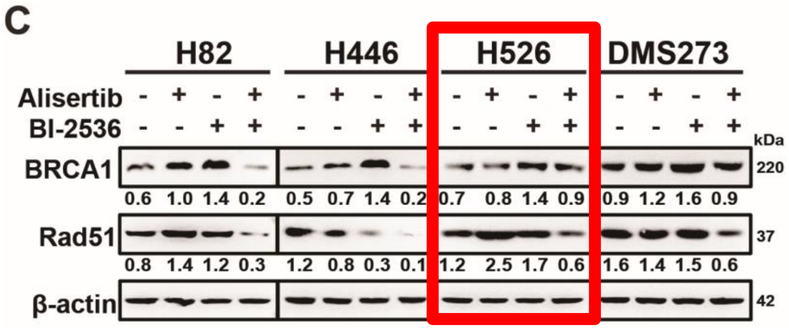

H526-βActin

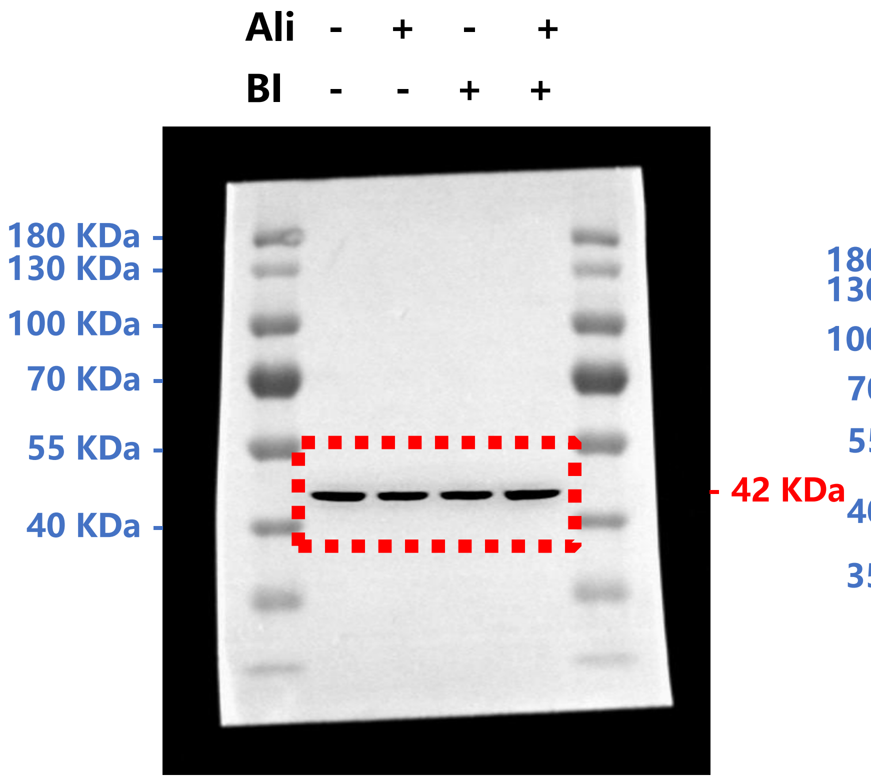

H526-BRCA1

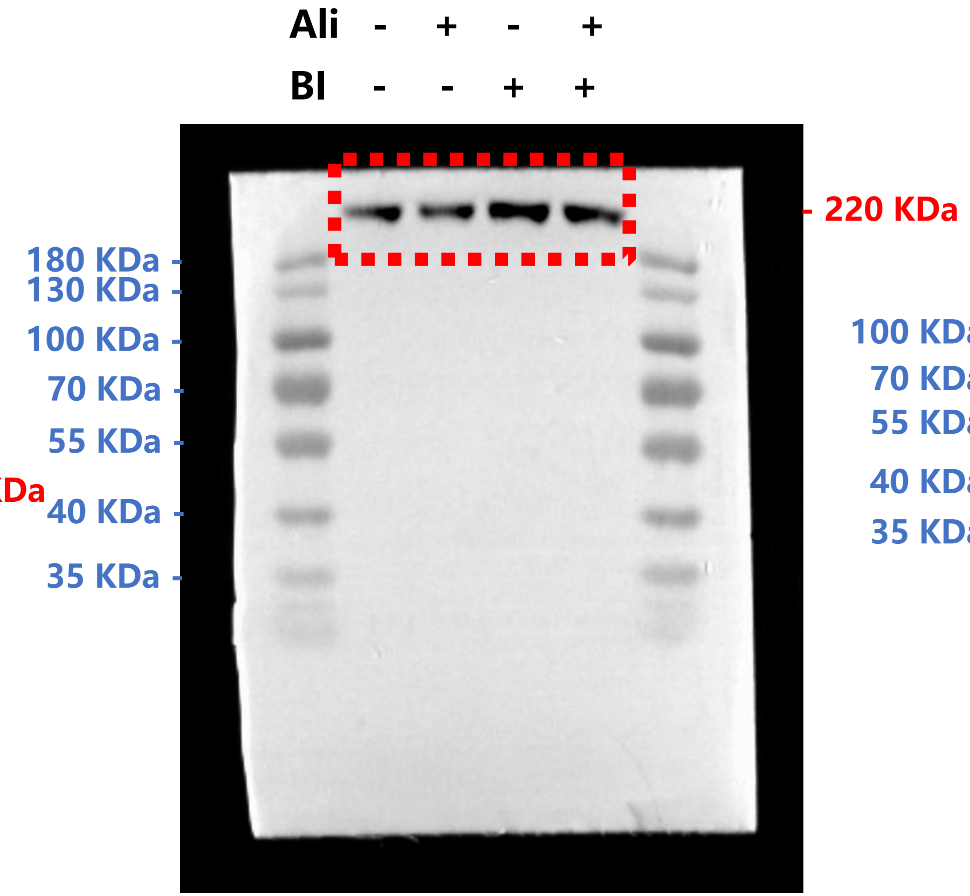

H526-Rad51

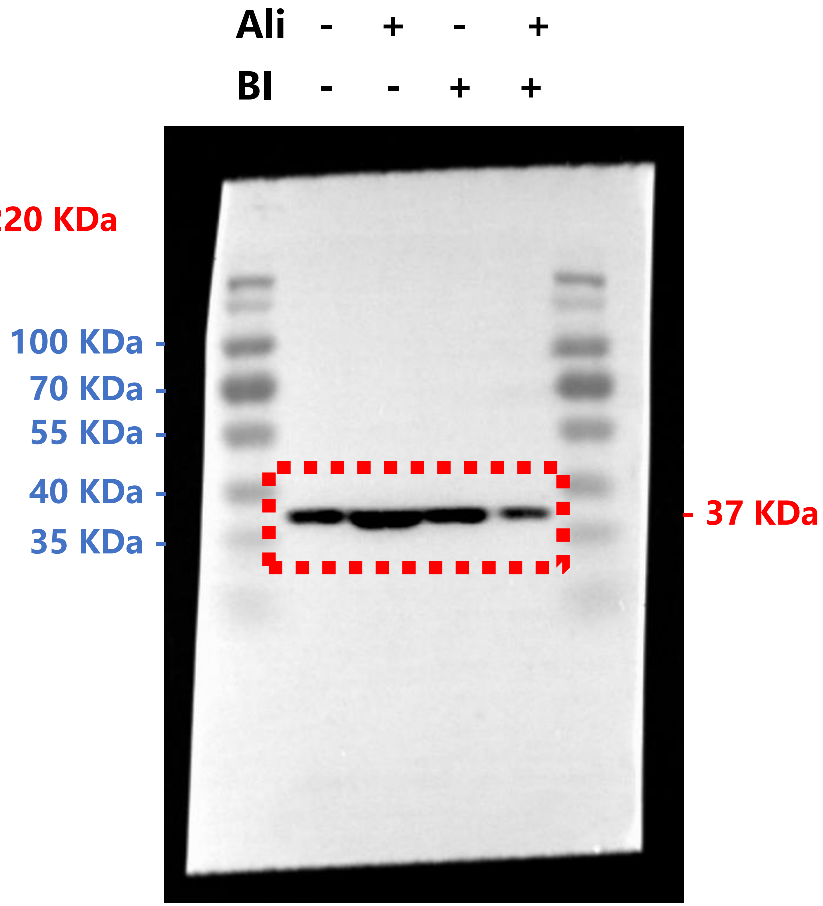

■ Original Western Blots of Figure S5-C

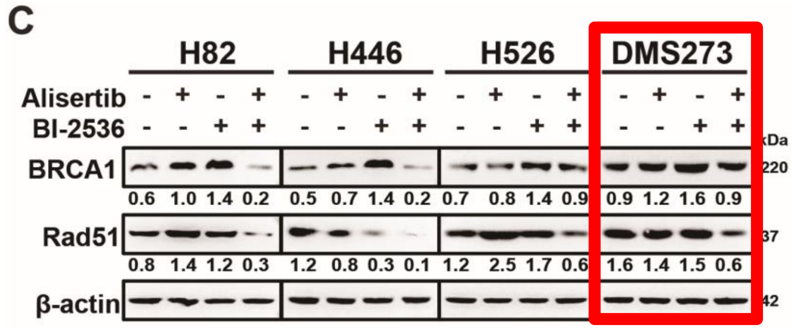

DMS273-βActin

Ali - + - +

BI - - + +

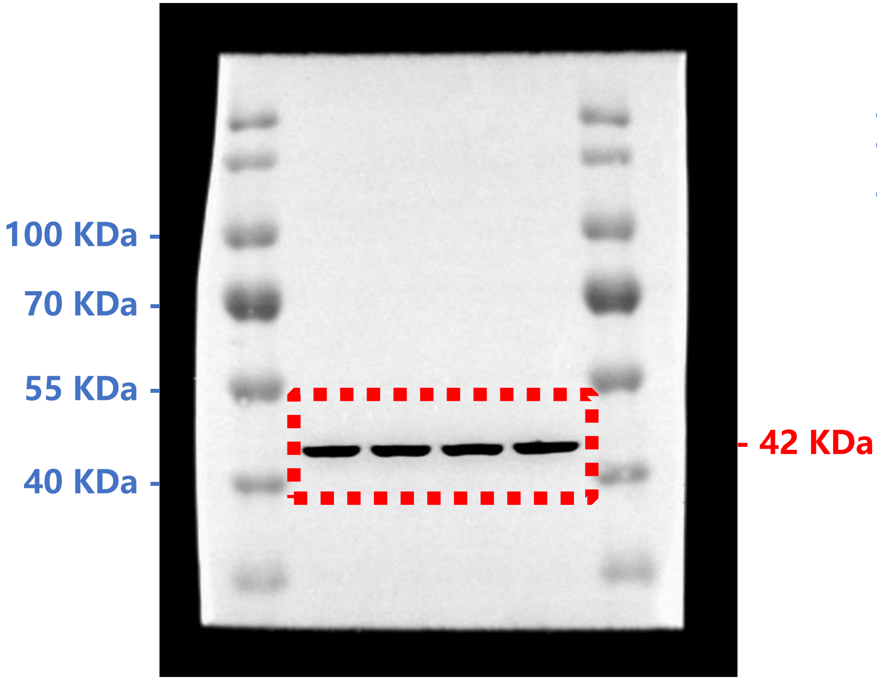

DMS273-BRCA1

Ali - + - +

BI - - + +

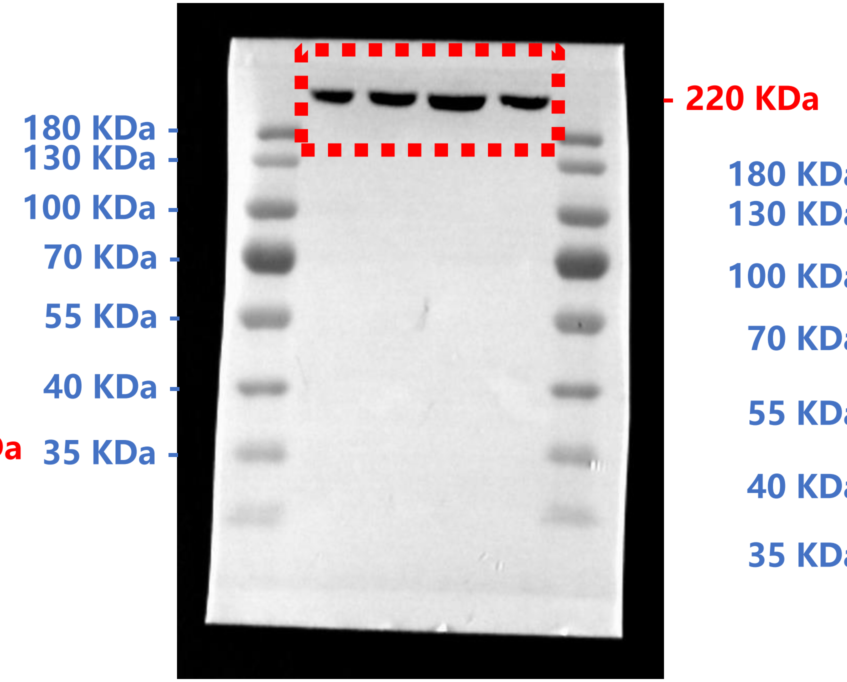

DMS273-Rad51

Ali - + - +

BI - - + +

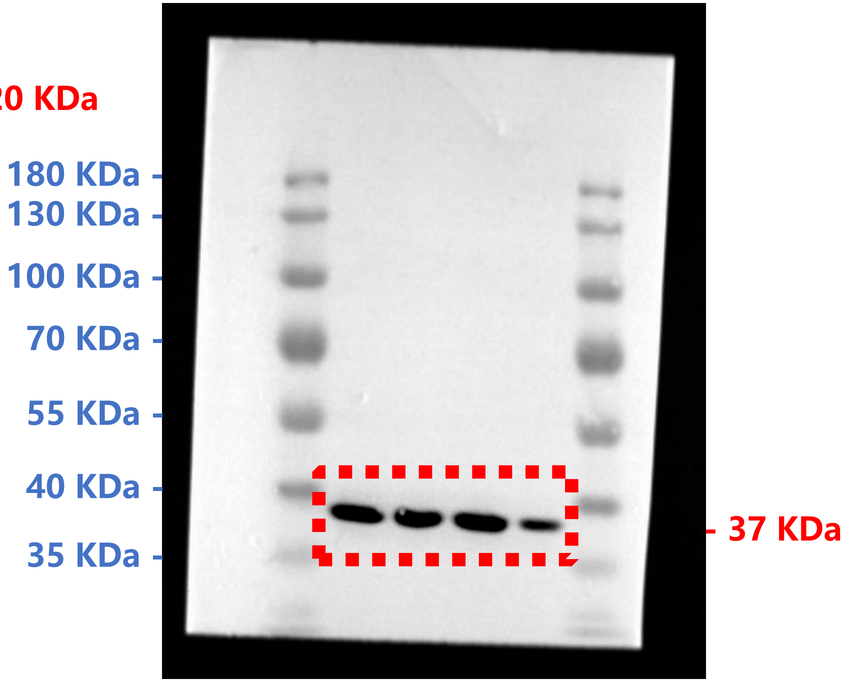

■ Original Western Blots of Figure S5-C

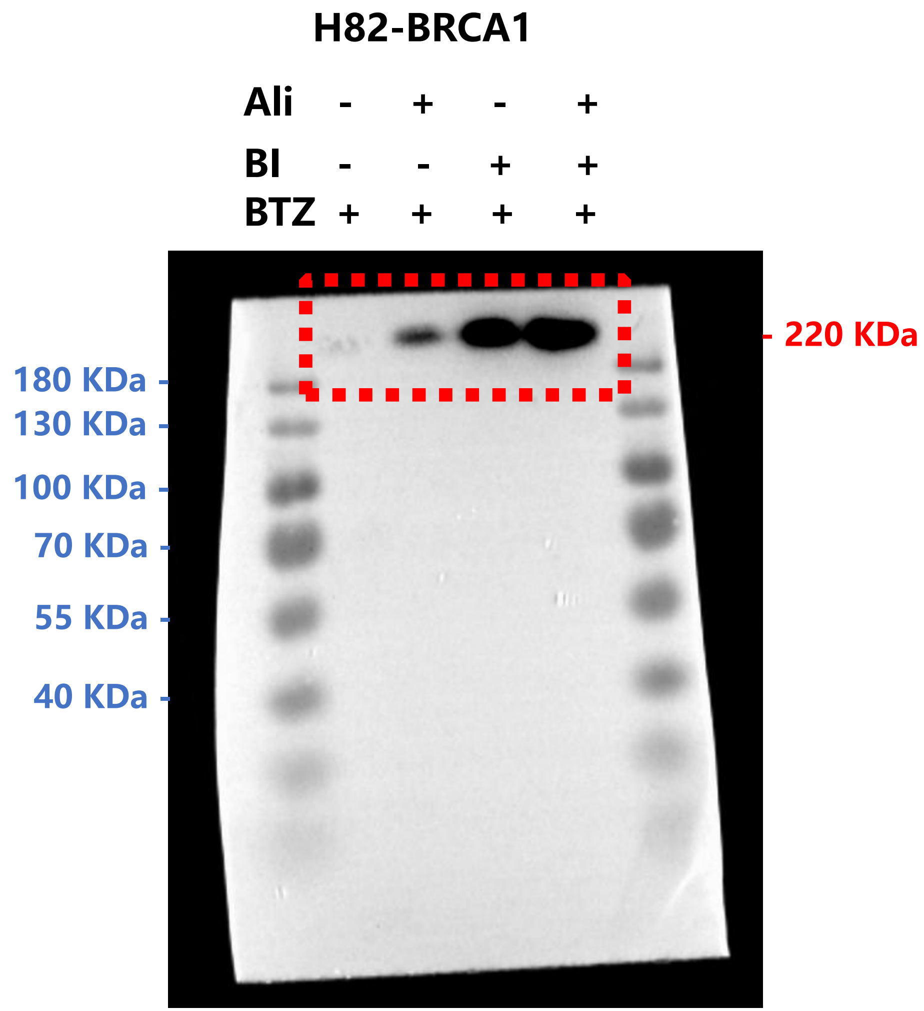

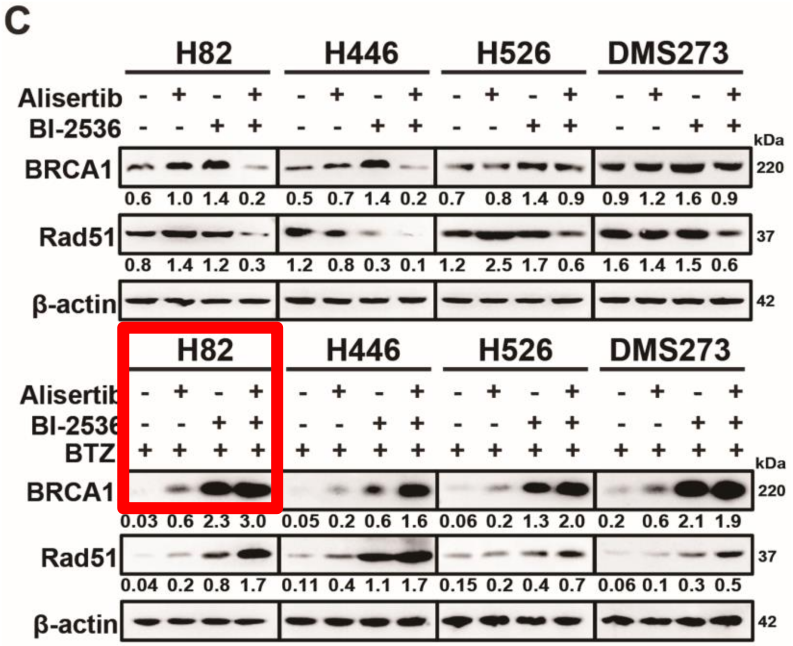

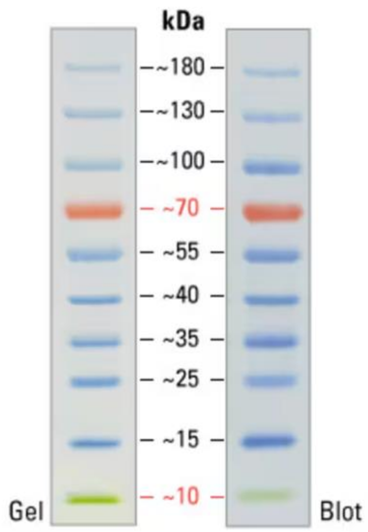

Thermo Scientific™ Marker-26617

■ **Original Western Blots of Figure S5-C**

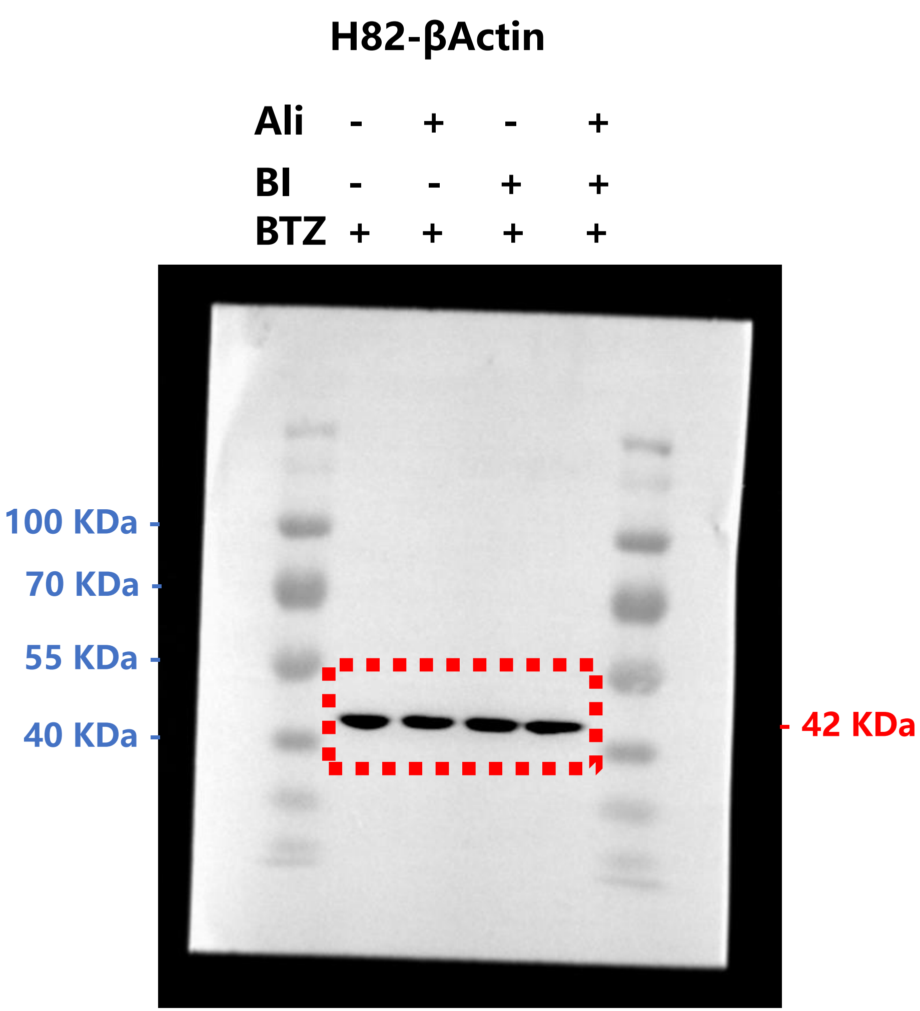

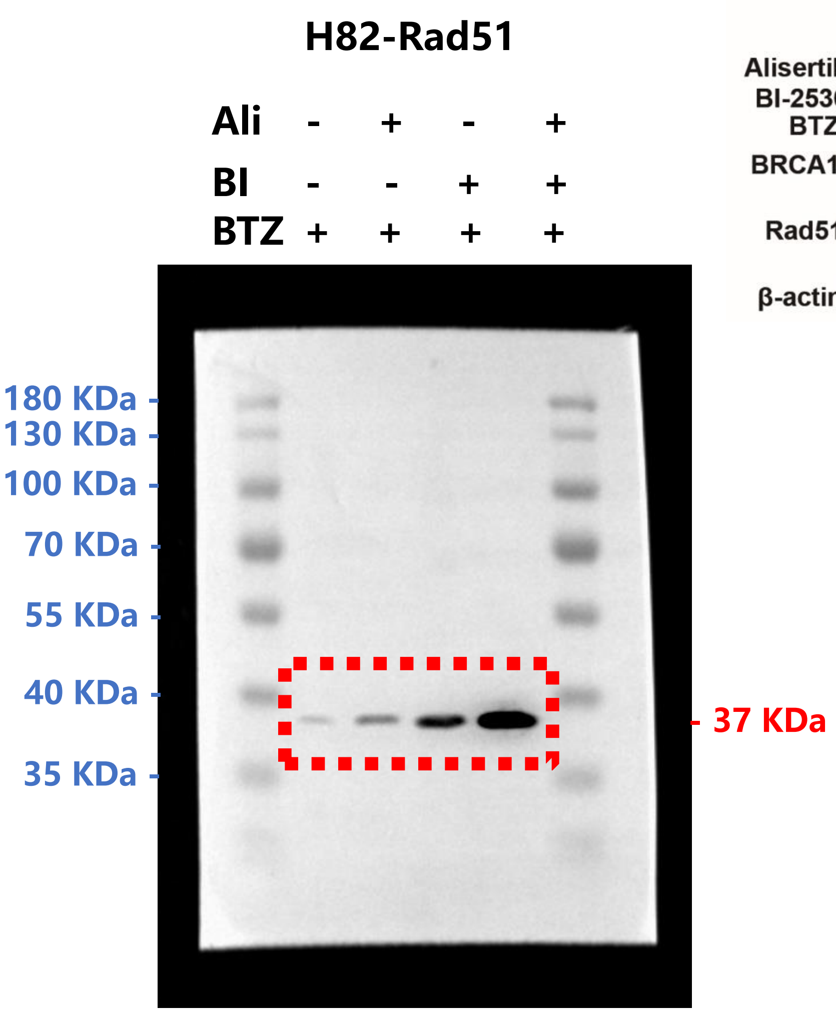

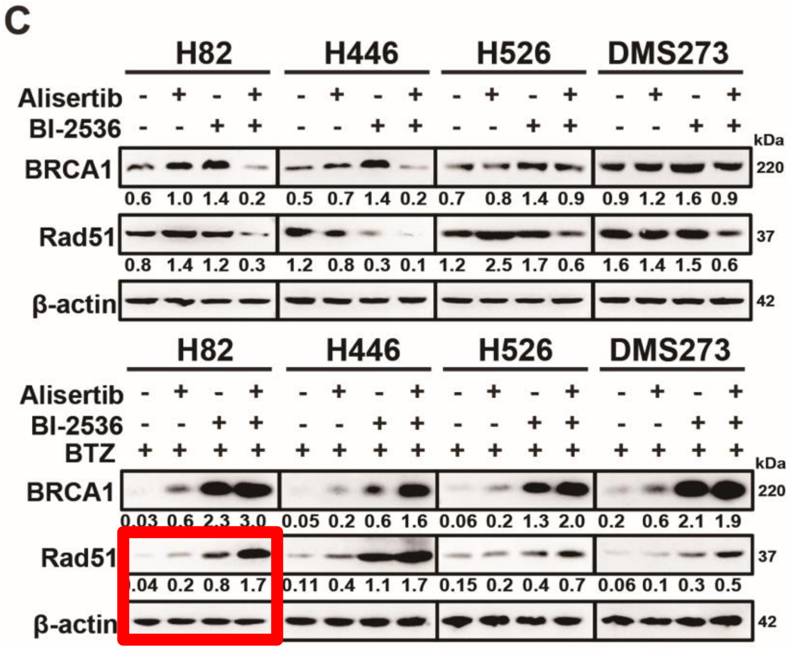

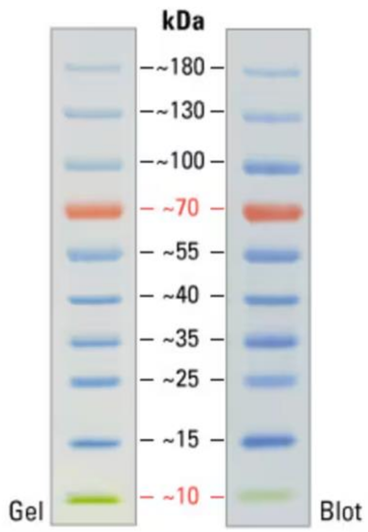

Thermo Scientific™ Marker-26617

■ Original Western Blots of Figure S5-C

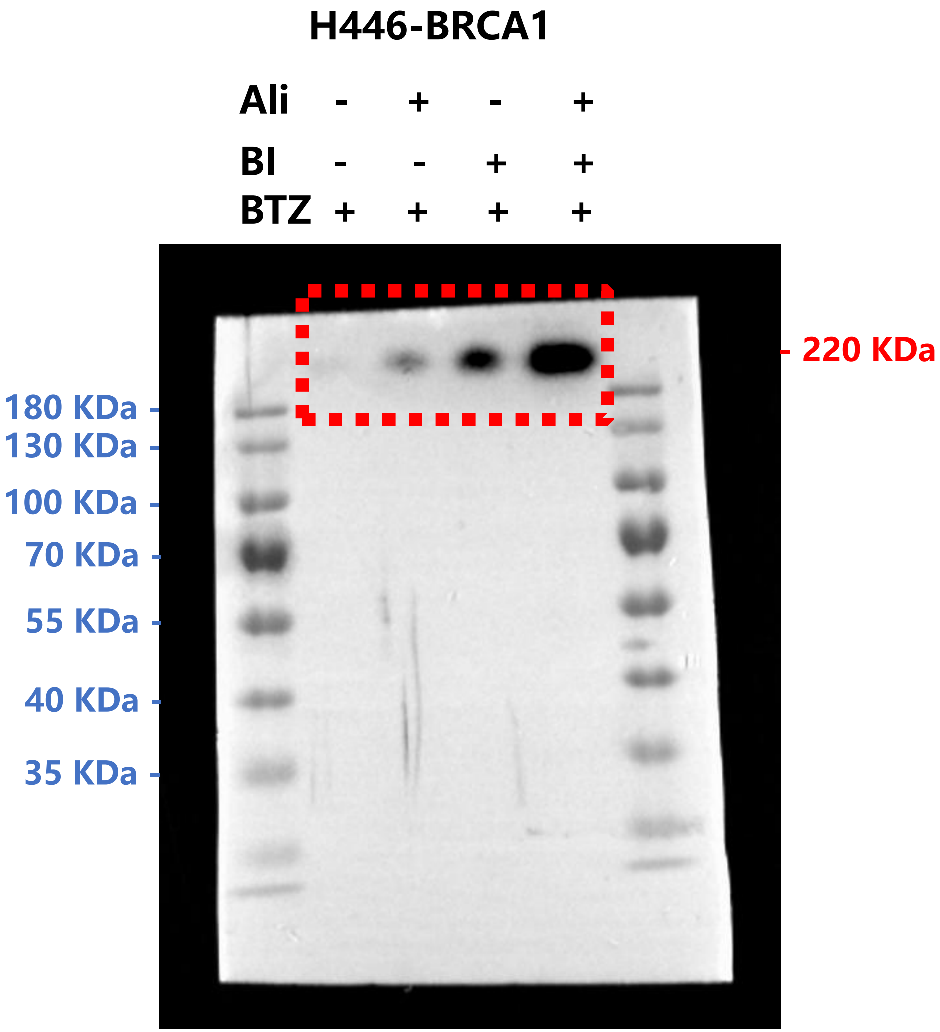

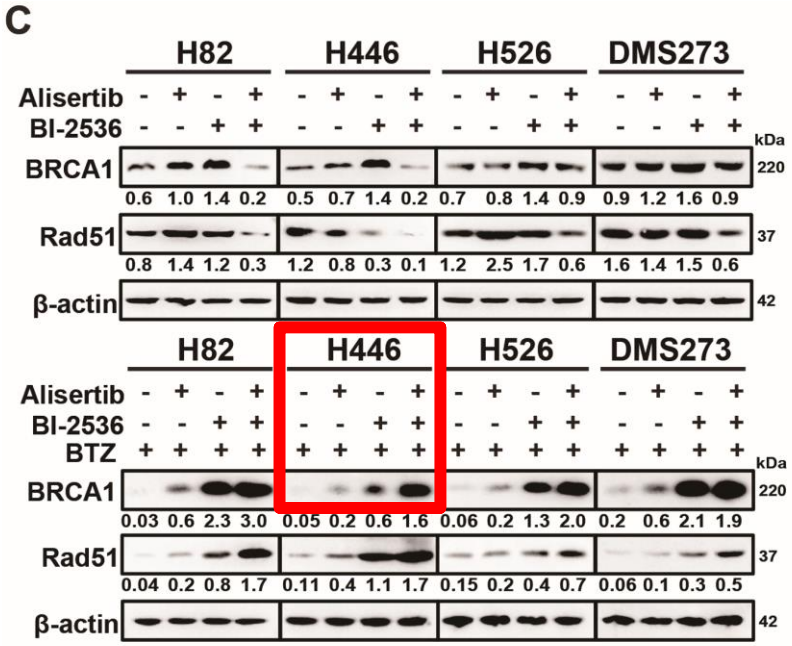

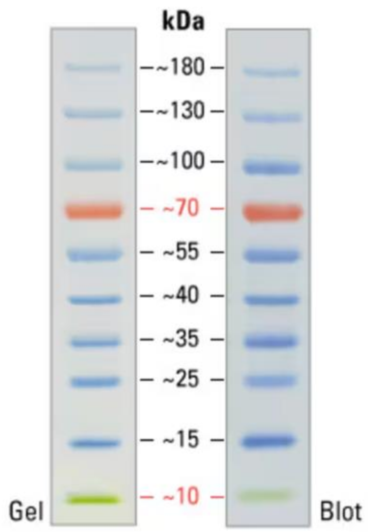

Thermo Scientific™ Marker-26617

■ Original Western Blots of Figure S5-C

H446-βActin

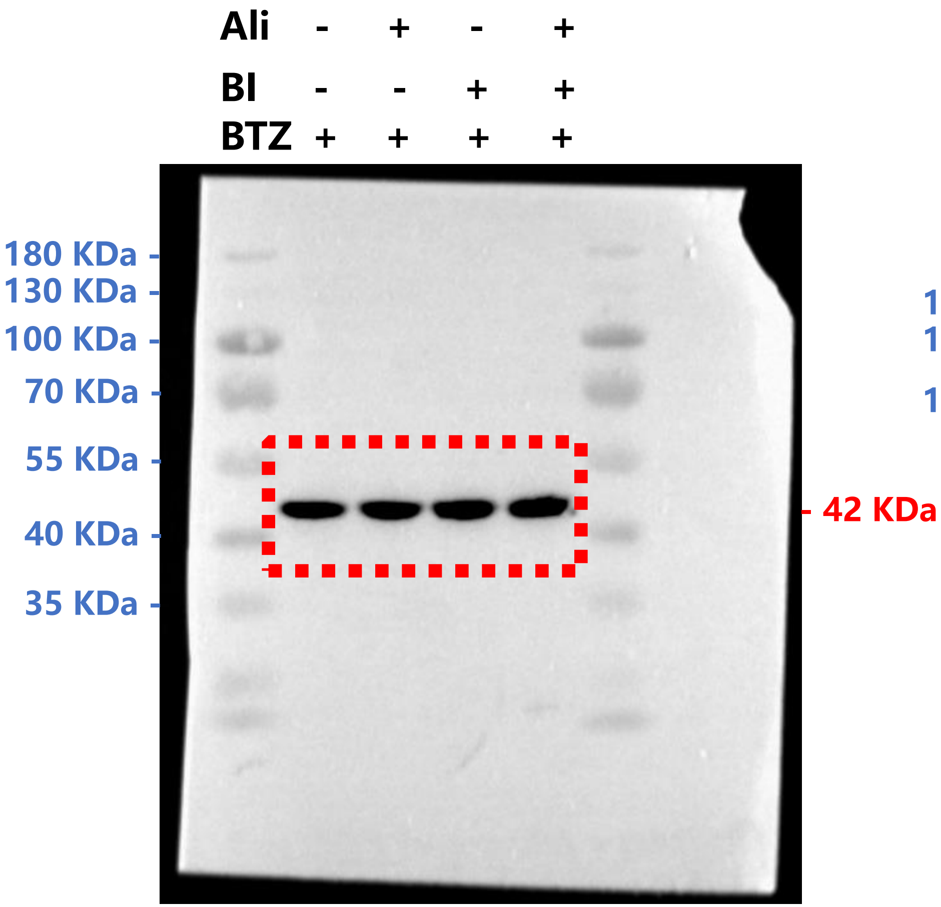

H446-Rad51

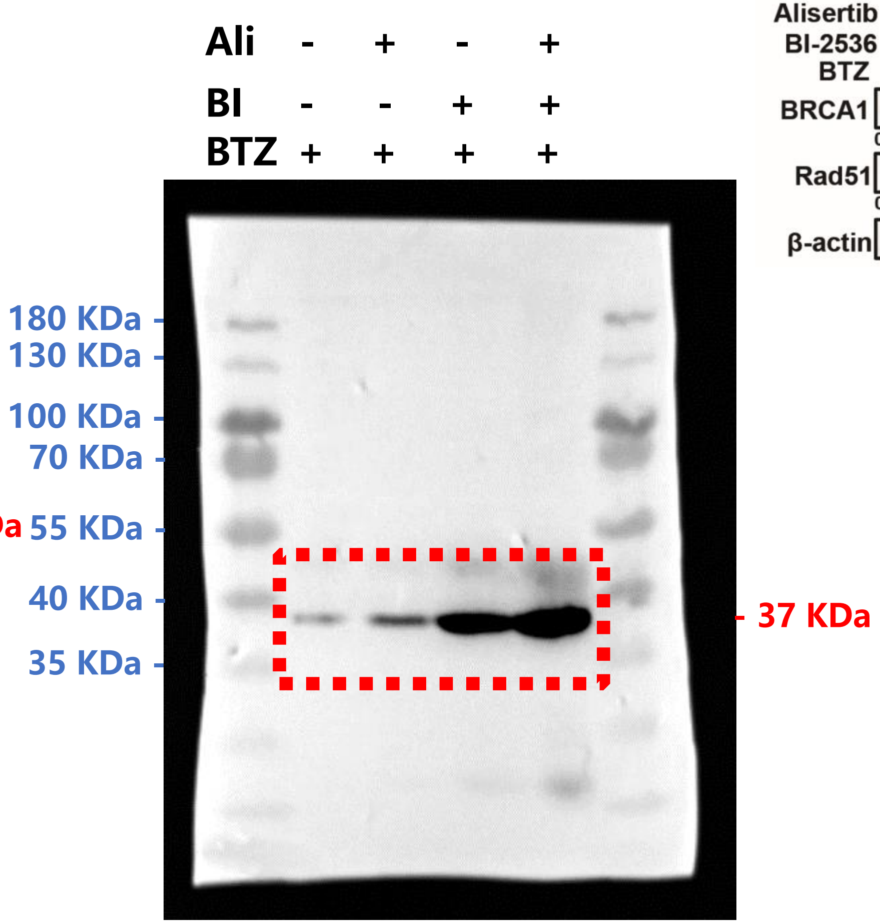

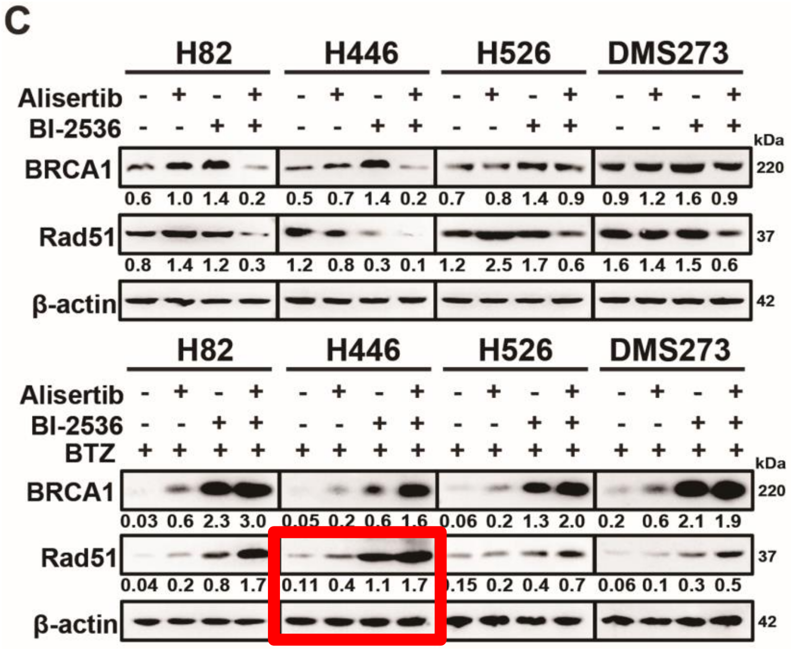

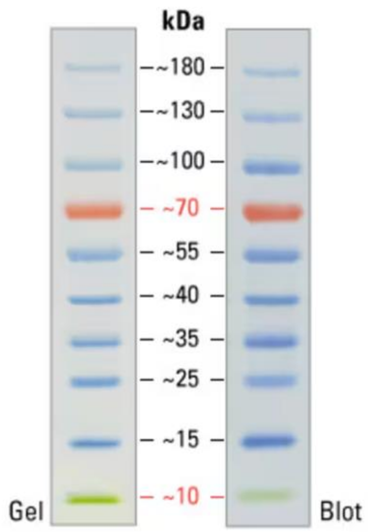

Thermo Scientific™ Marker-26617

■ Original Western Blots of Figure S5-C

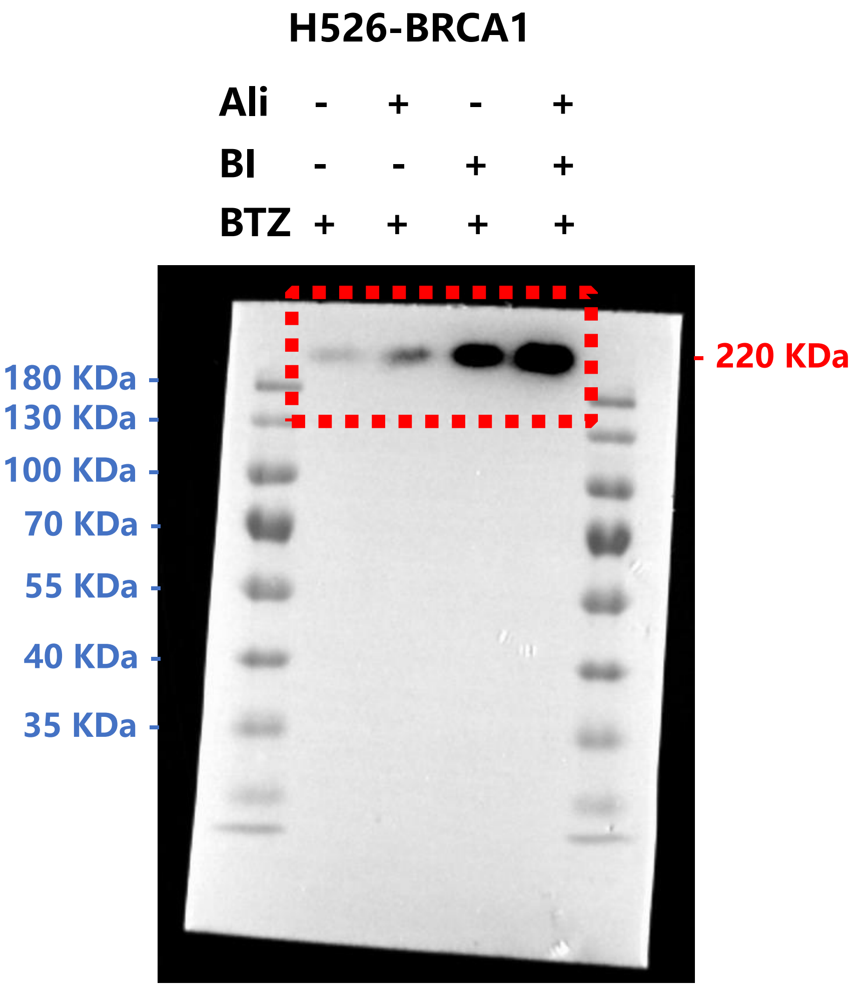

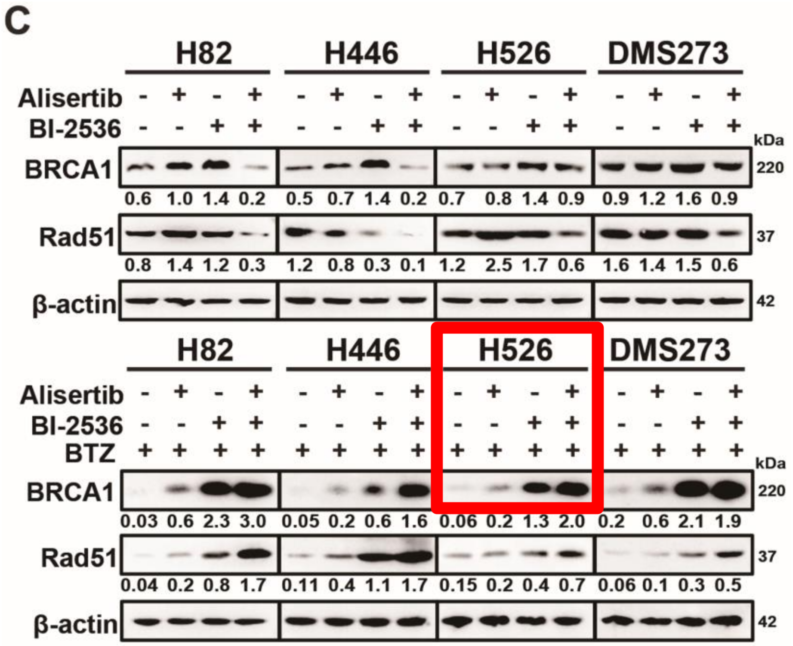

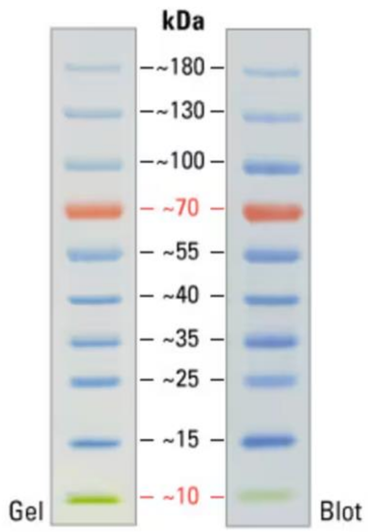

Thermo Scientific™ Marker-26617

■ Original Western Blots of Figure S5-C

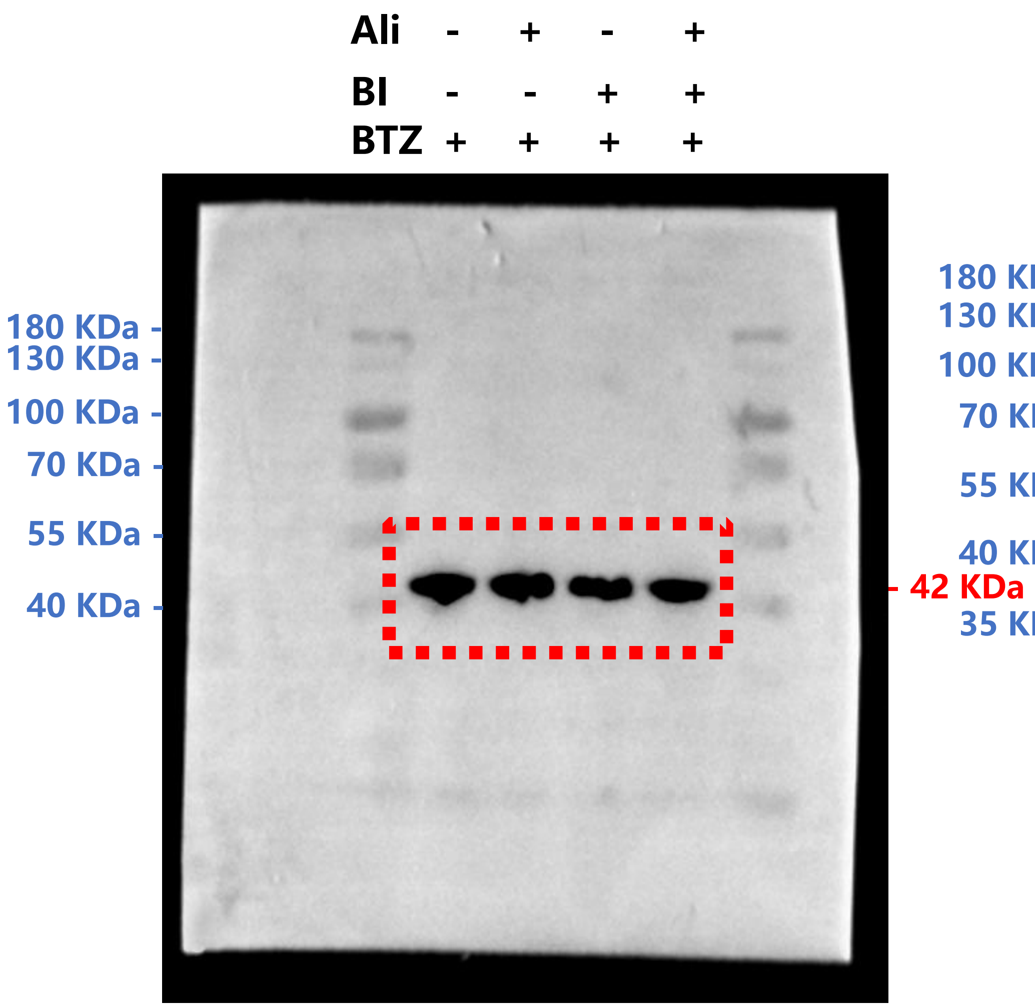

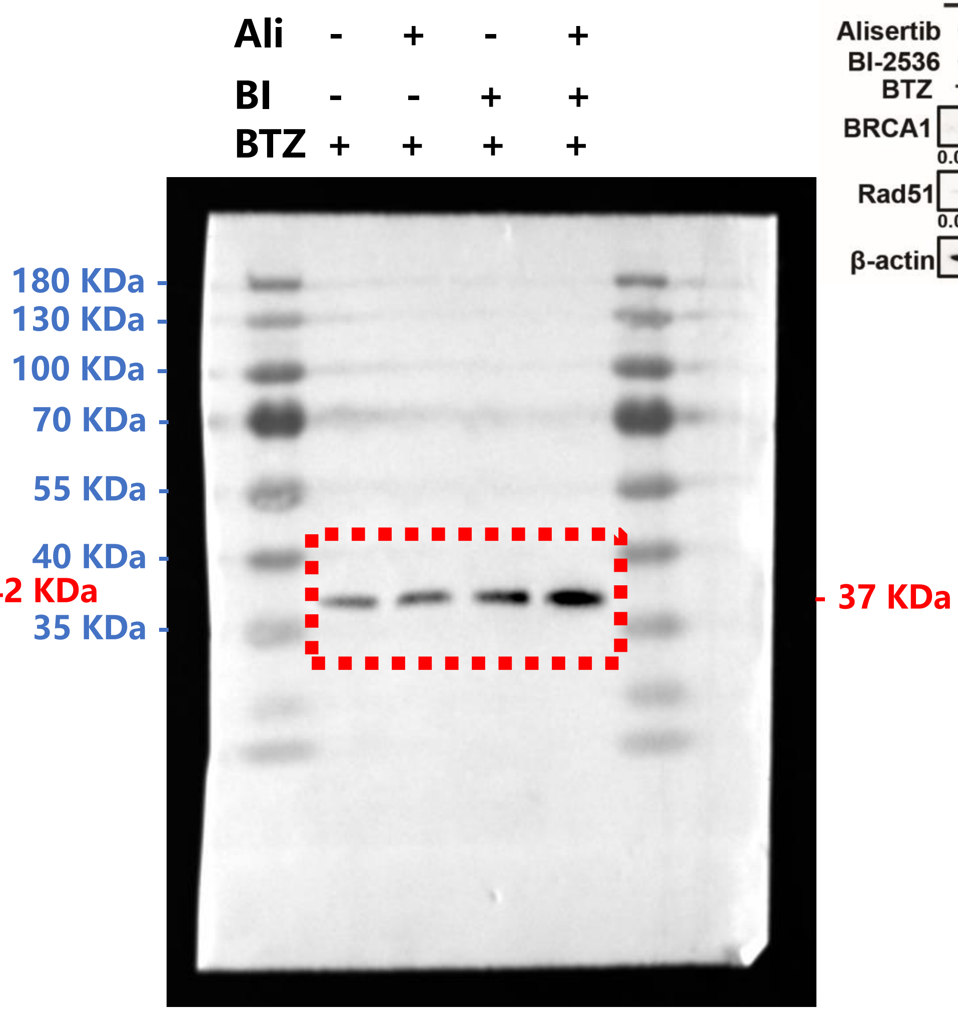

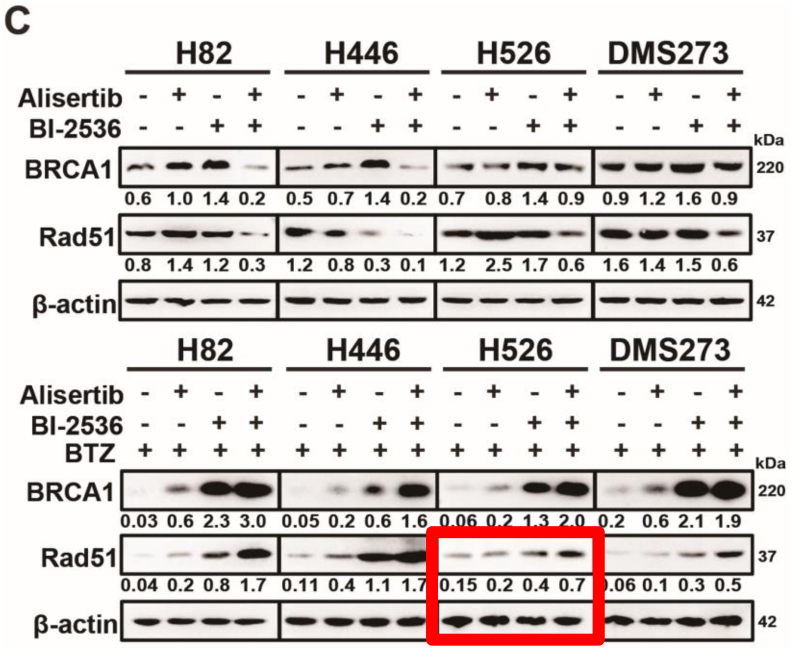

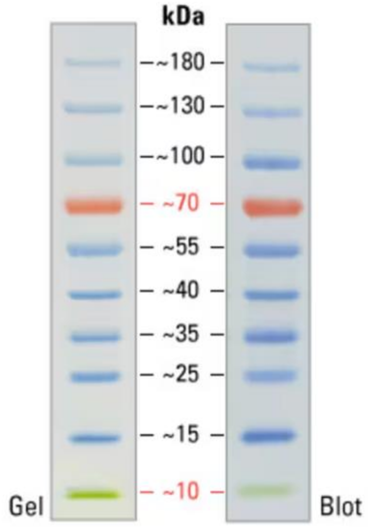

Thermo Scientific™ Marker-26617

■ Original Western Blots of Figure S5-C

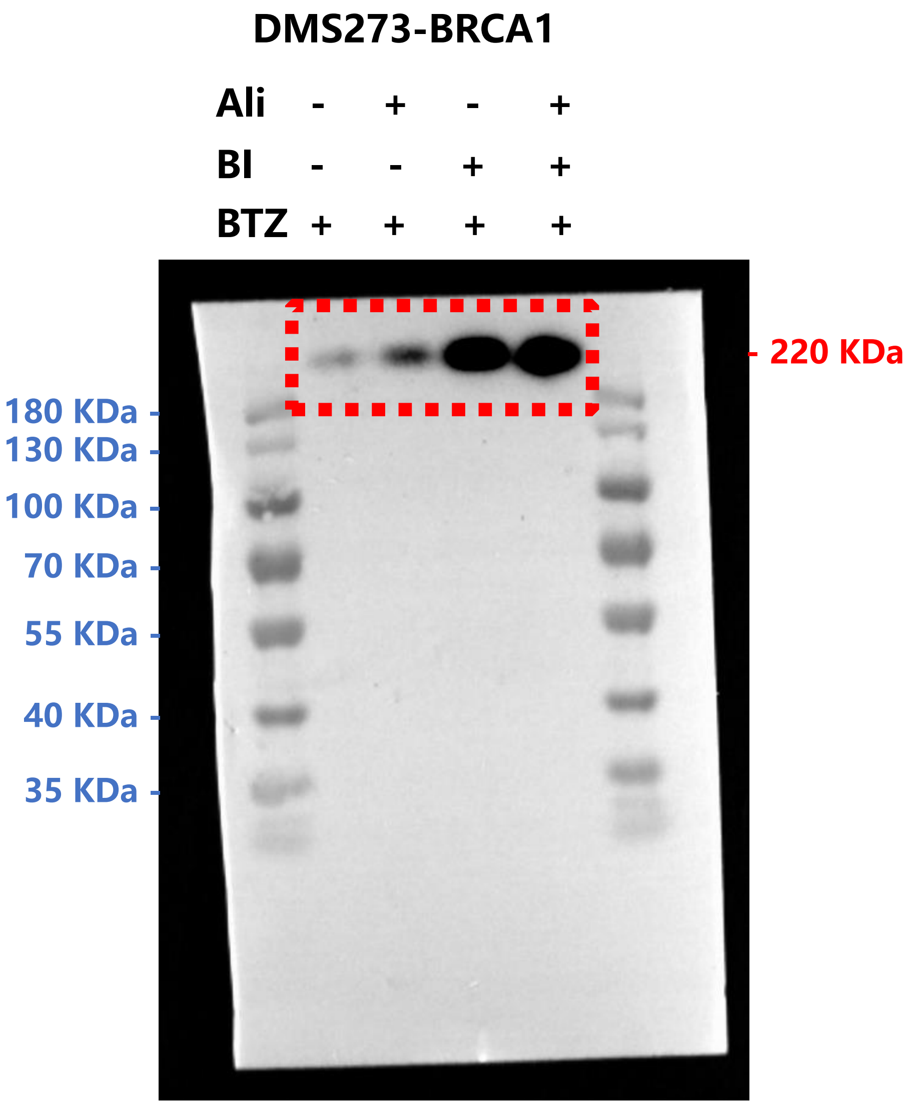

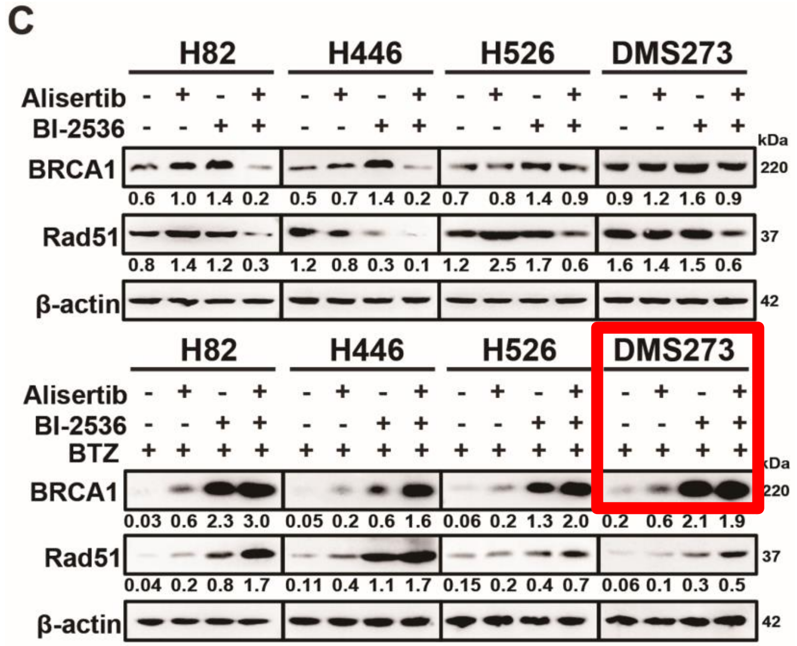

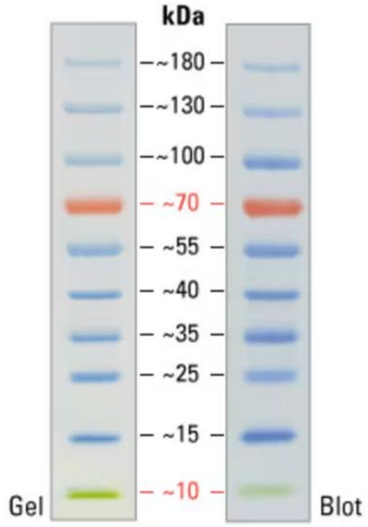

Thermo Scientific™ Marker-26617

■ Original Western Blots of Figure S5-C

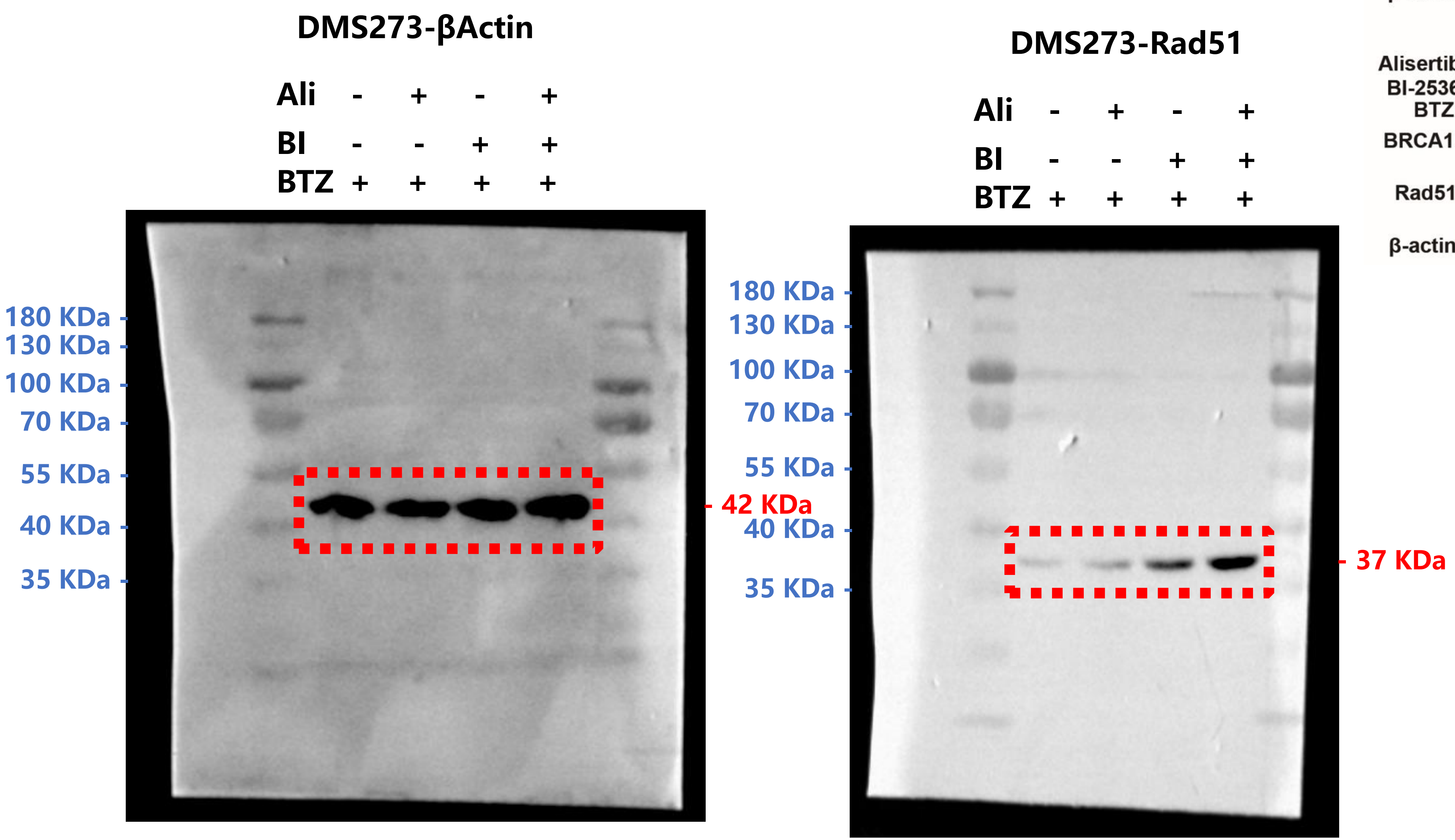

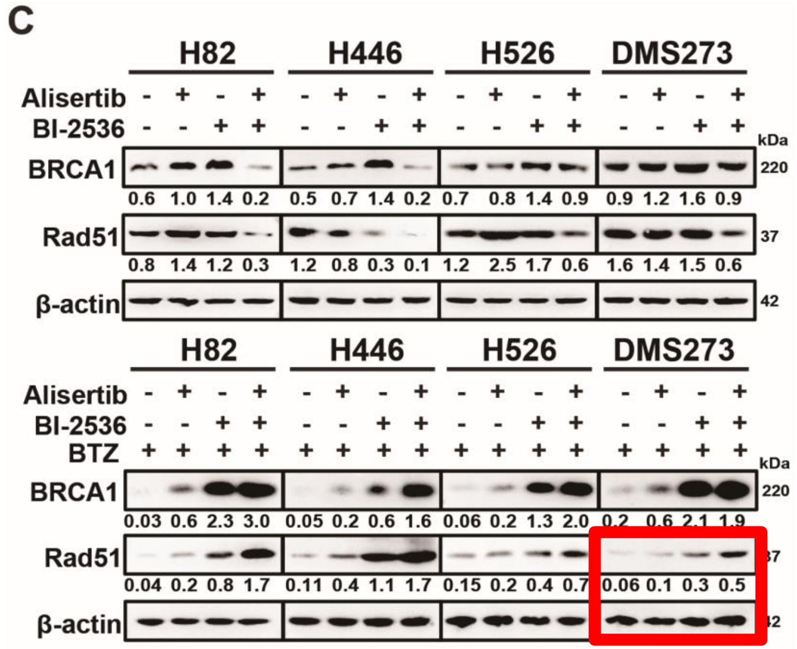

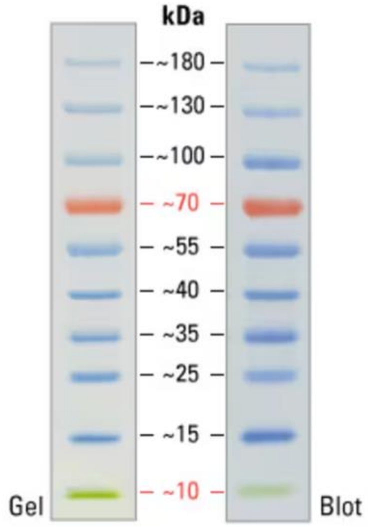

Thermo Scientific™ Marker-26617

■ Original Western Blots of Figure S9-C

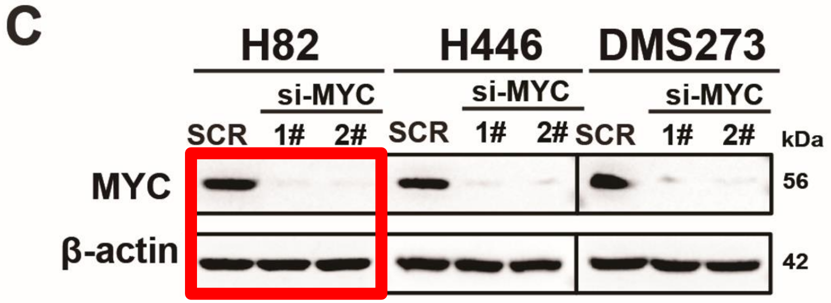

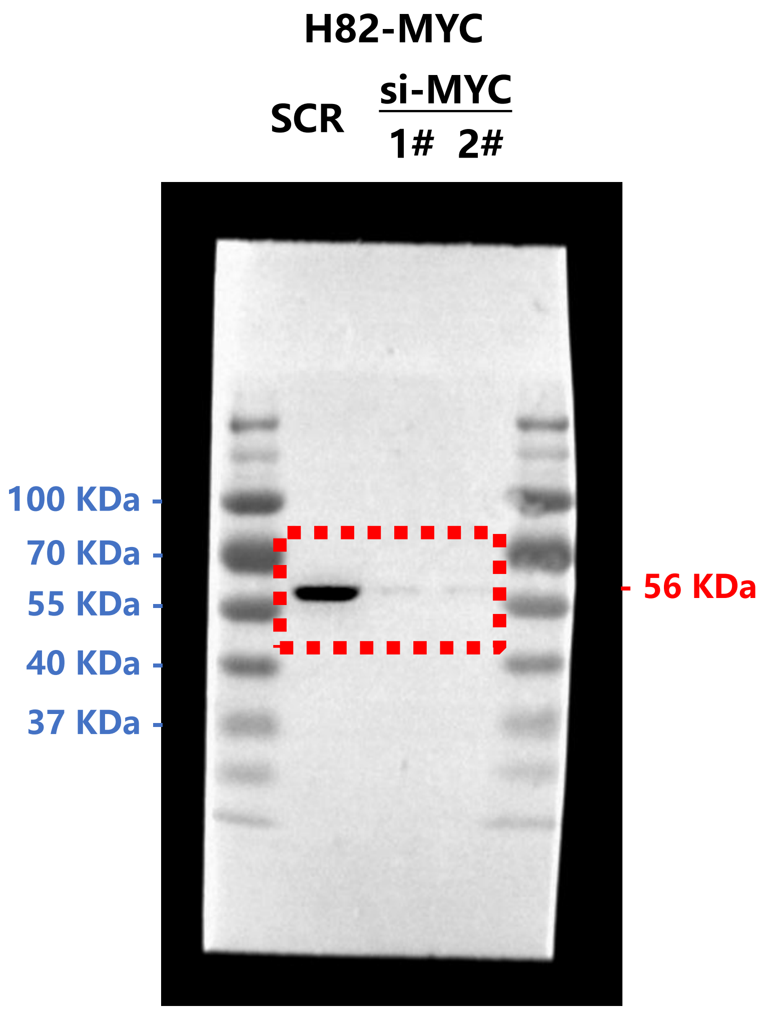

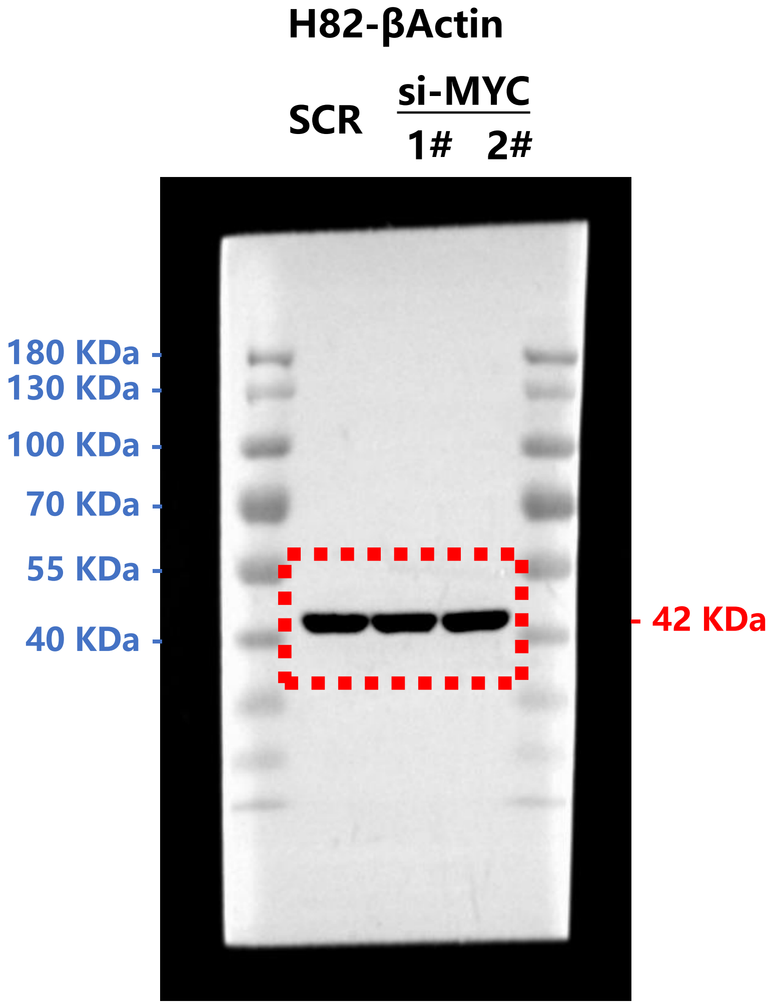

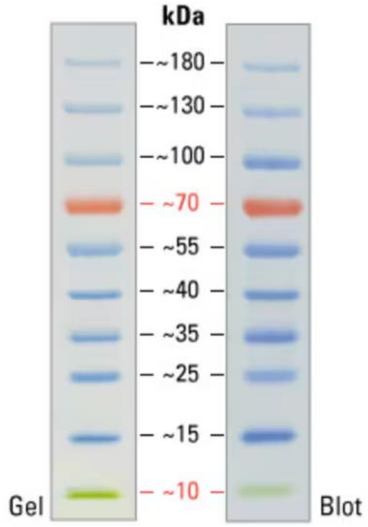

Thermo Scientific™ Marker-26617

■ Original Western Blots of Figure S9-C

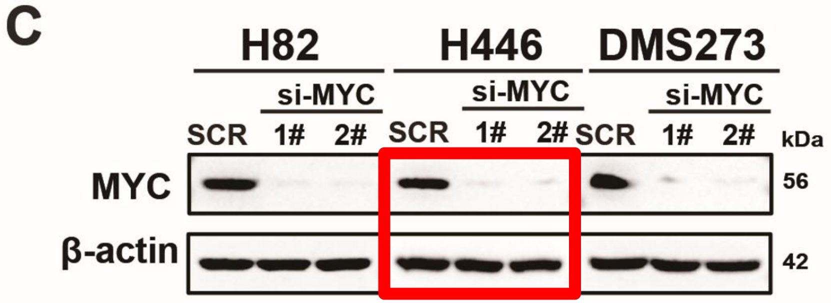

H446-MYC

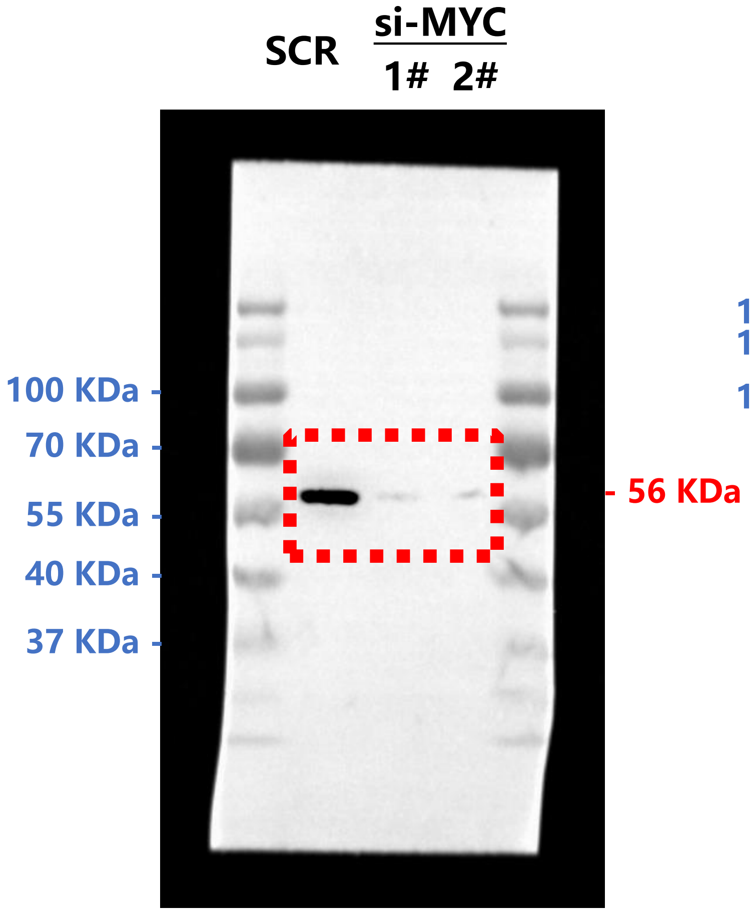

H446-βActin

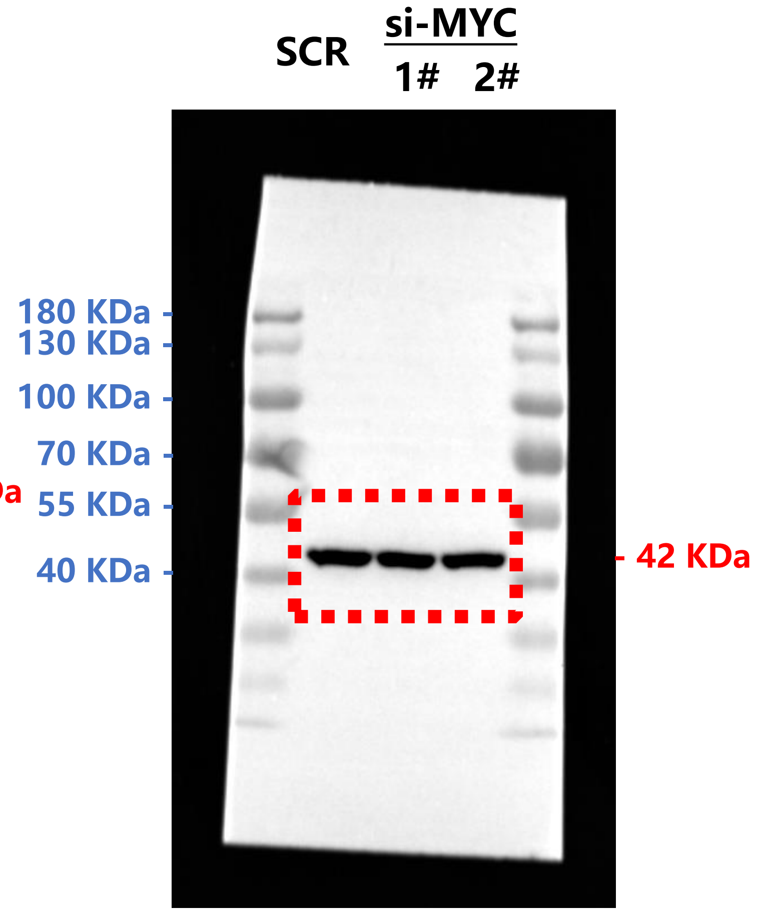

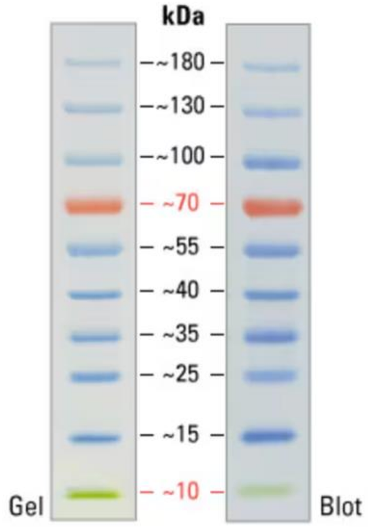

Thermo Scientific™ Marker-26617

■ Original Western Blots of Figure S9-C

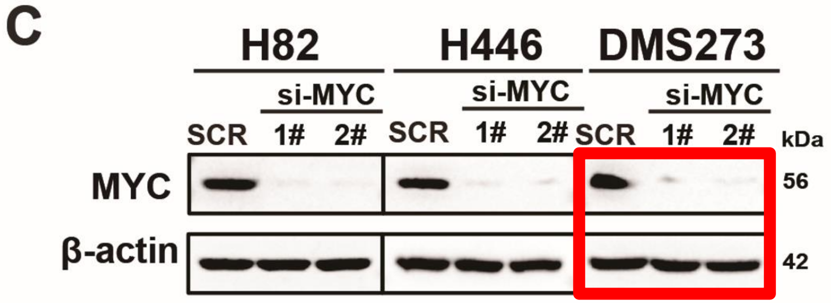

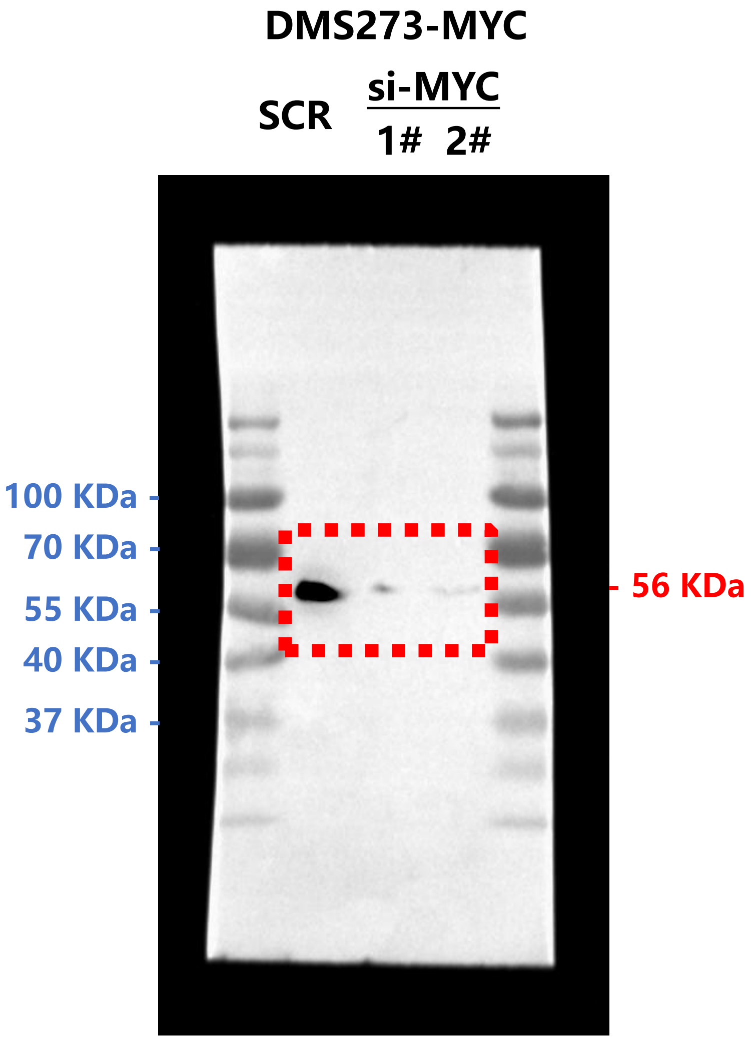

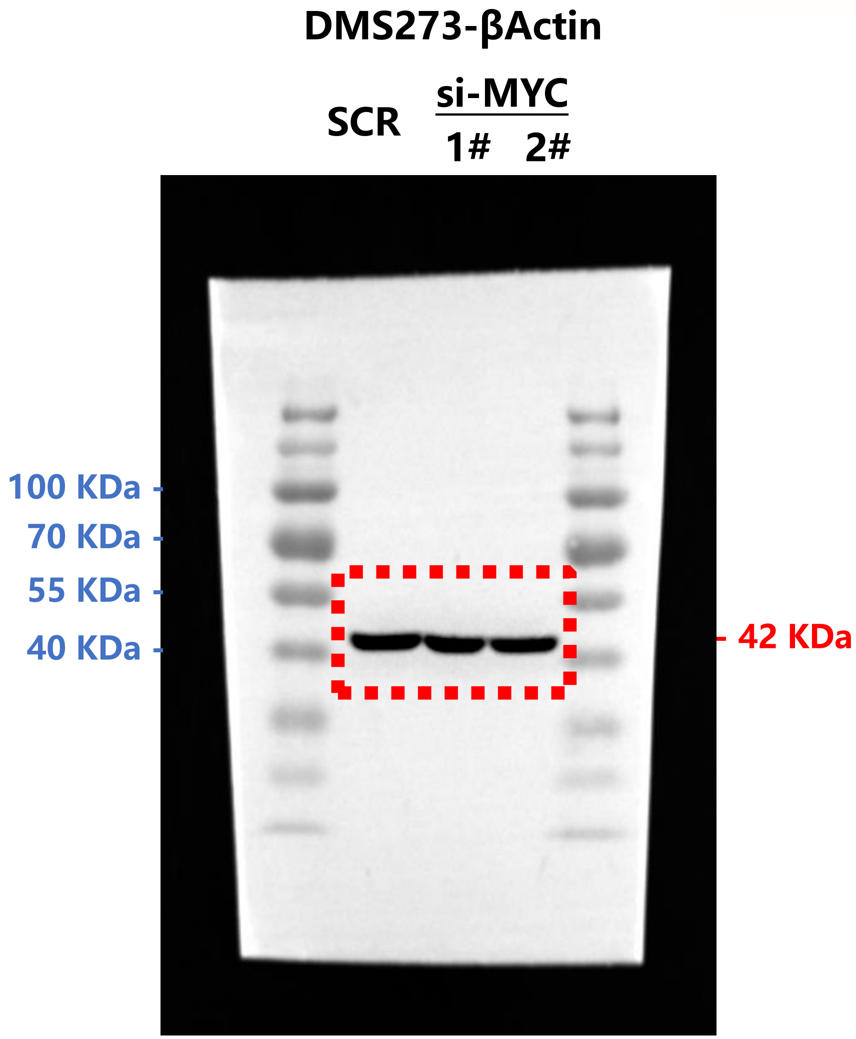

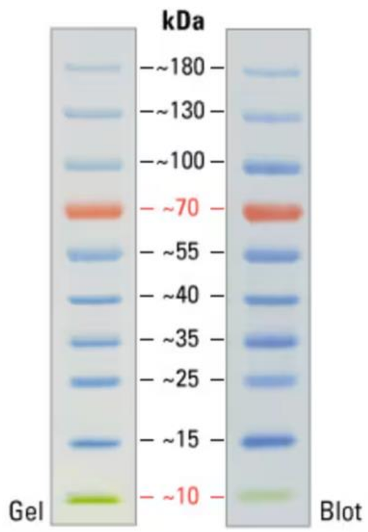

Thermo Scientific™ Marker-26617

■ Original Western Blots of Figure S9-D

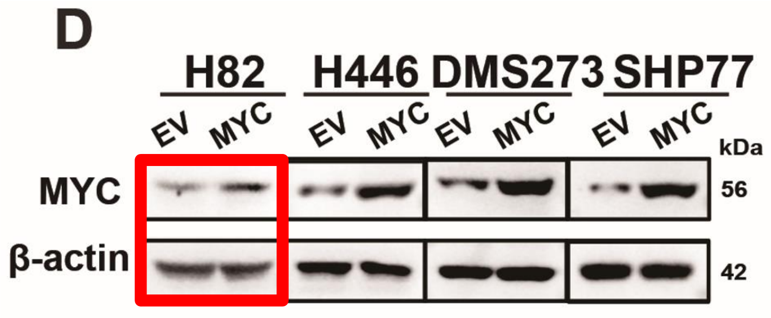

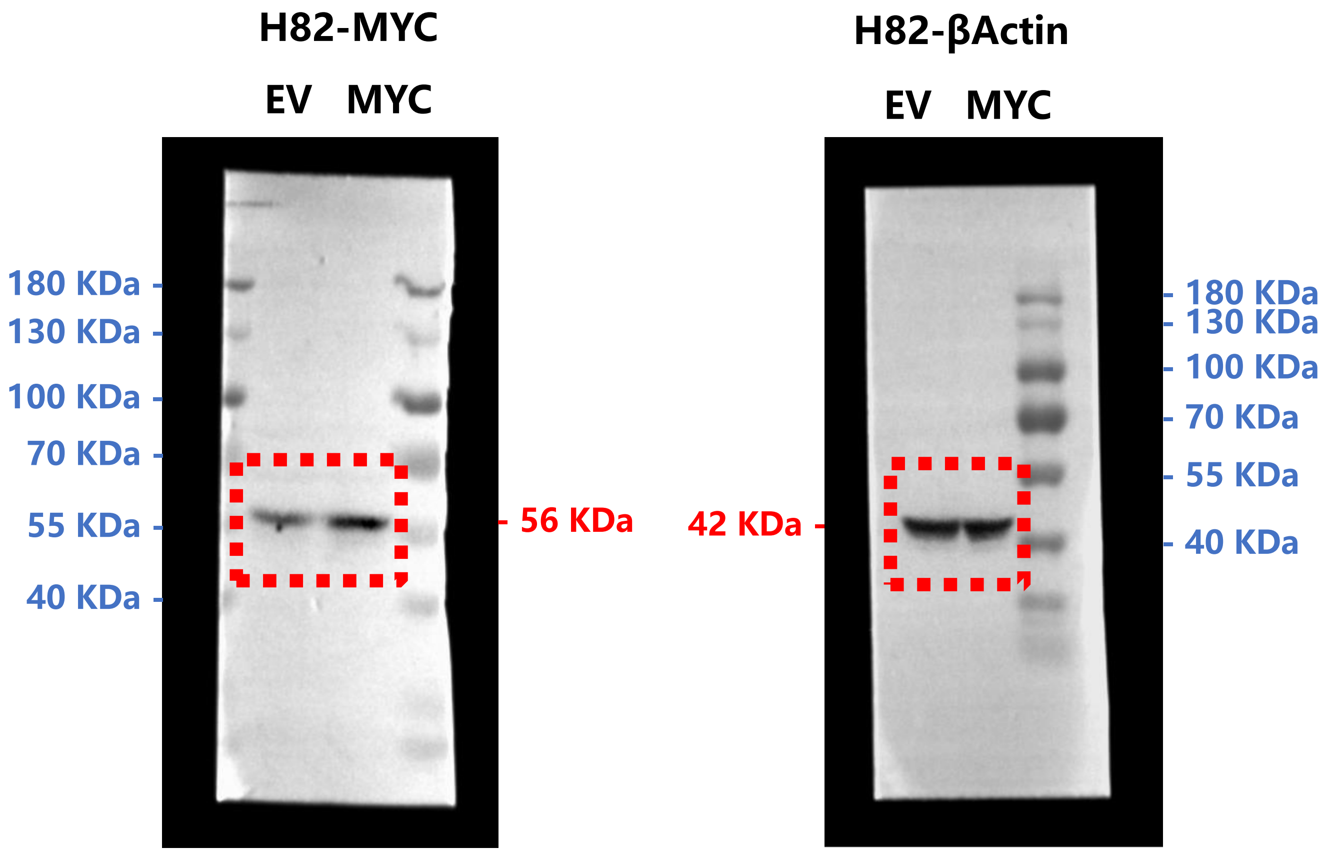

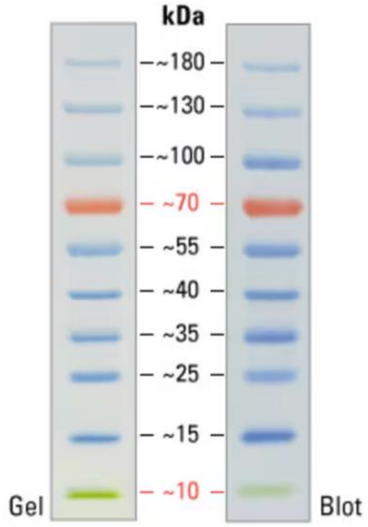

Thermo Scientific™ Marker-26617

■ **Original Western Blots of Figure S9-D**

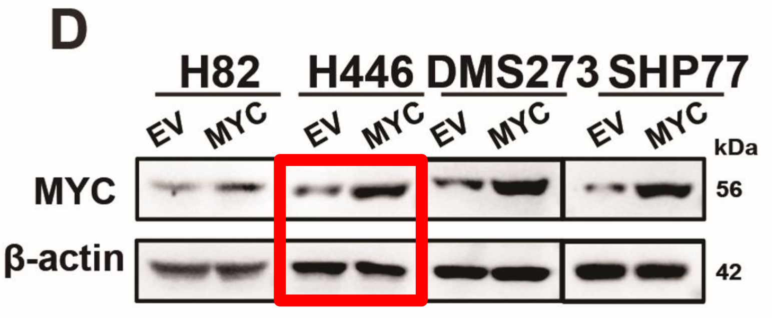

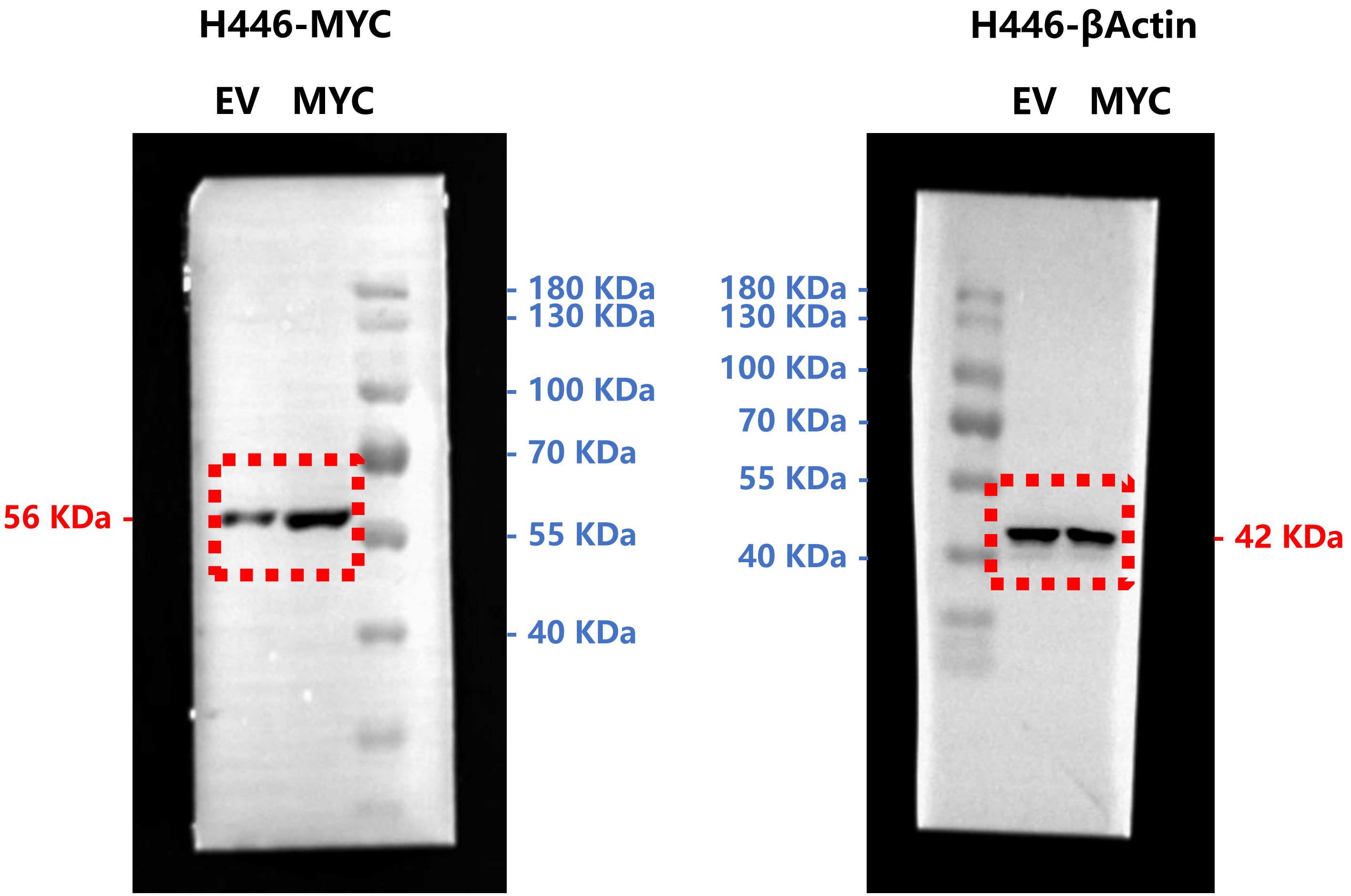

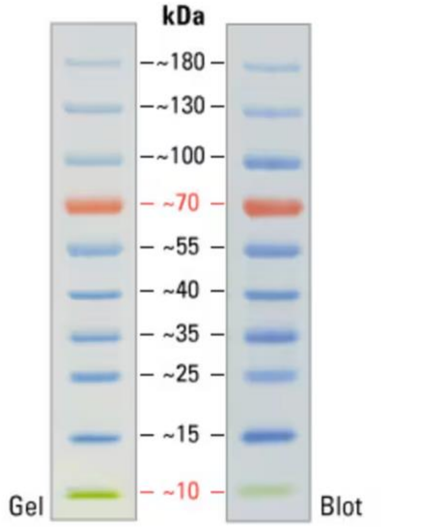

Thermo Scientific™ Marker-26617

■ Original Western Blots of Figure S9-D

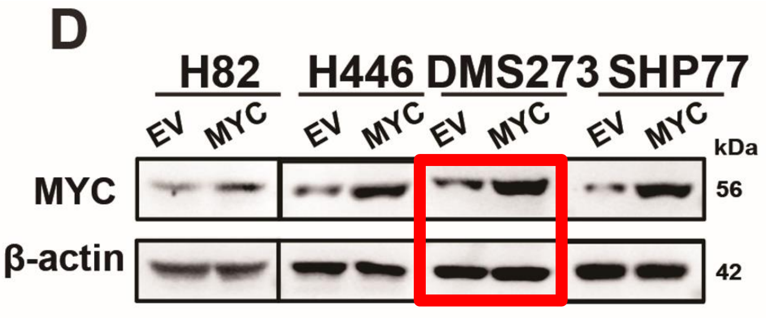

DMS273-MYC

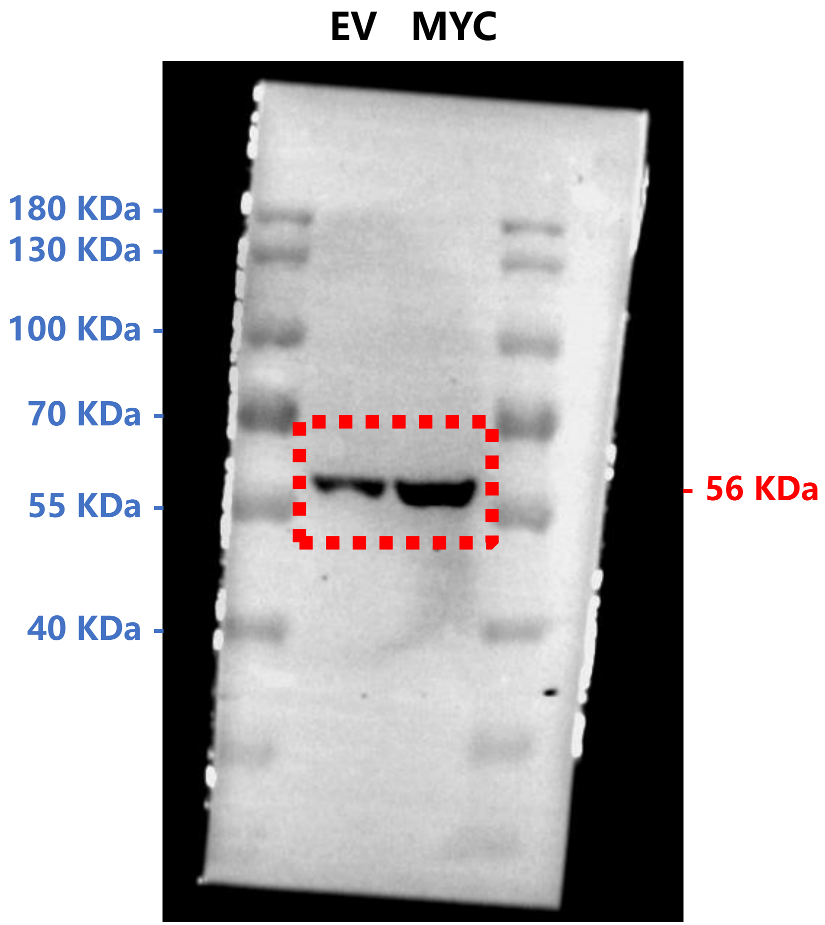

DMS273-βActin

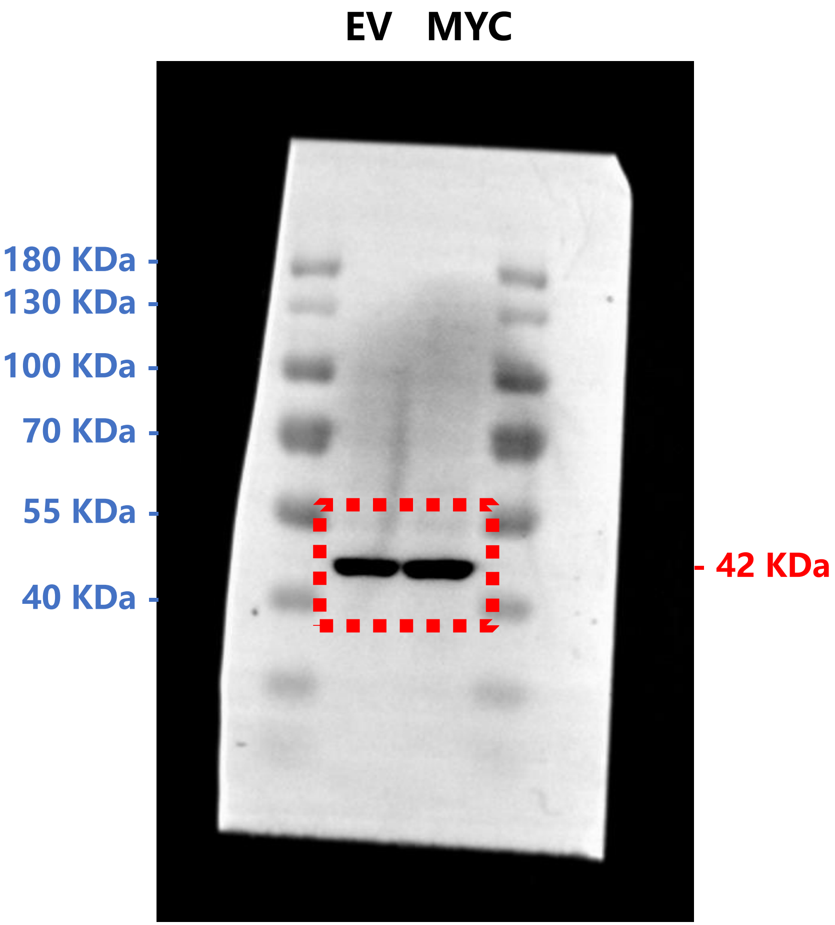

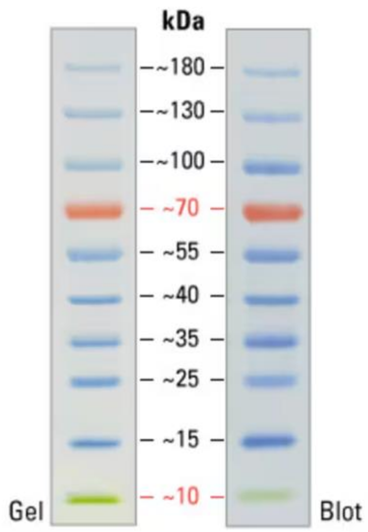

Thermo Scientific™ Marker-26617

■ Original Western Blots of Figure S9-D

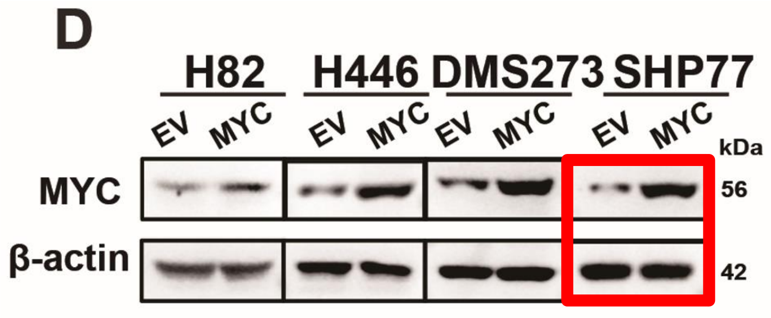

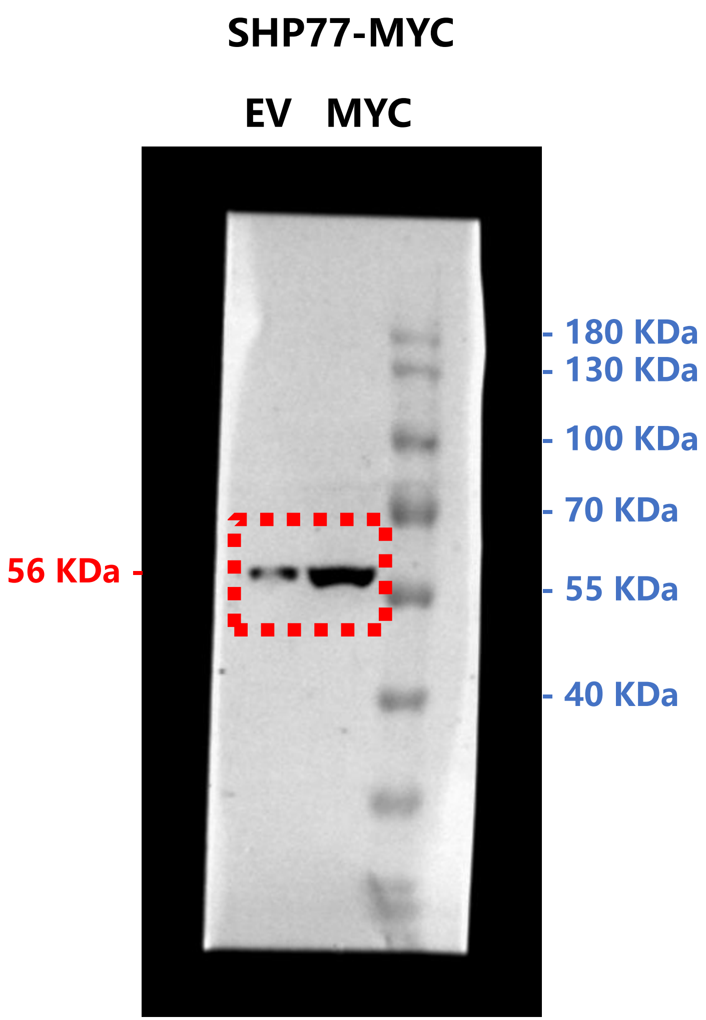

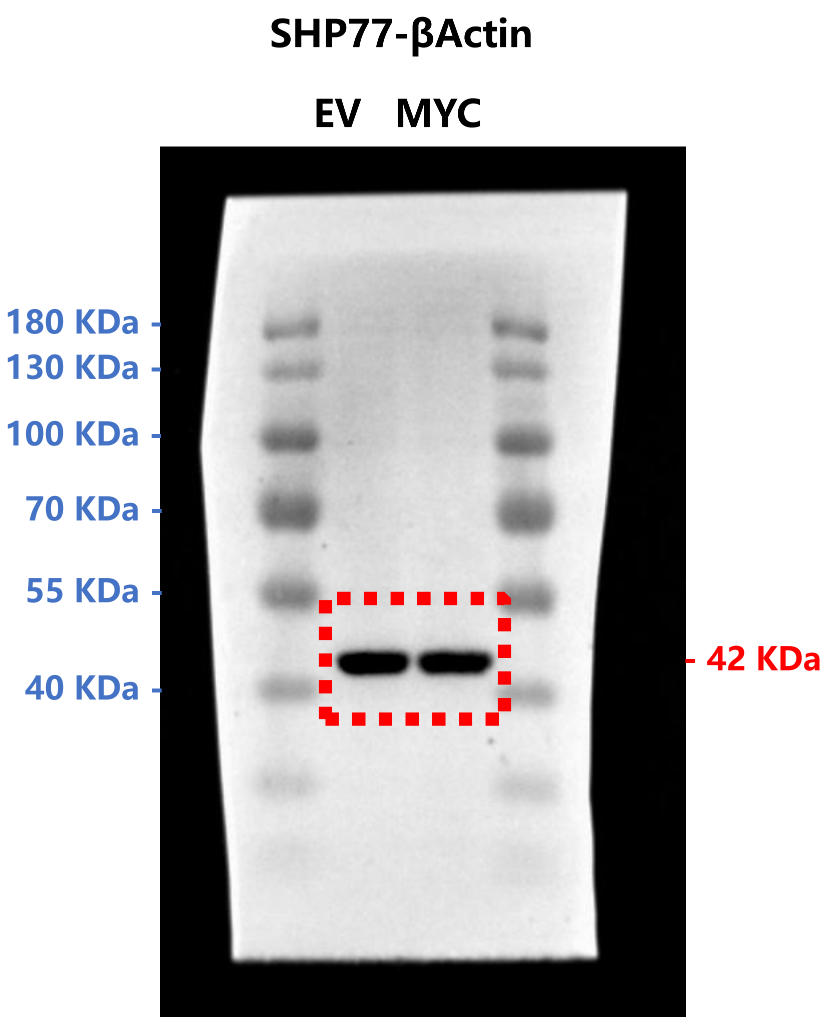

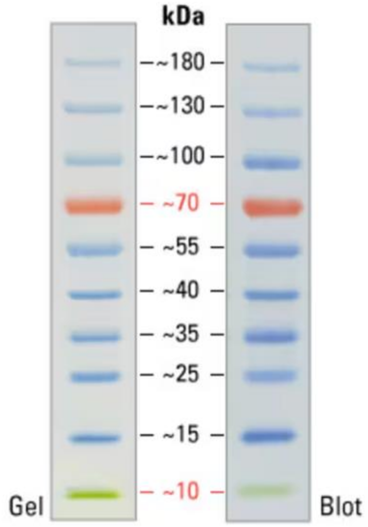

Thermo Scientific™ Marker-26617

■ **Original Western Blots of Figure S9-E**

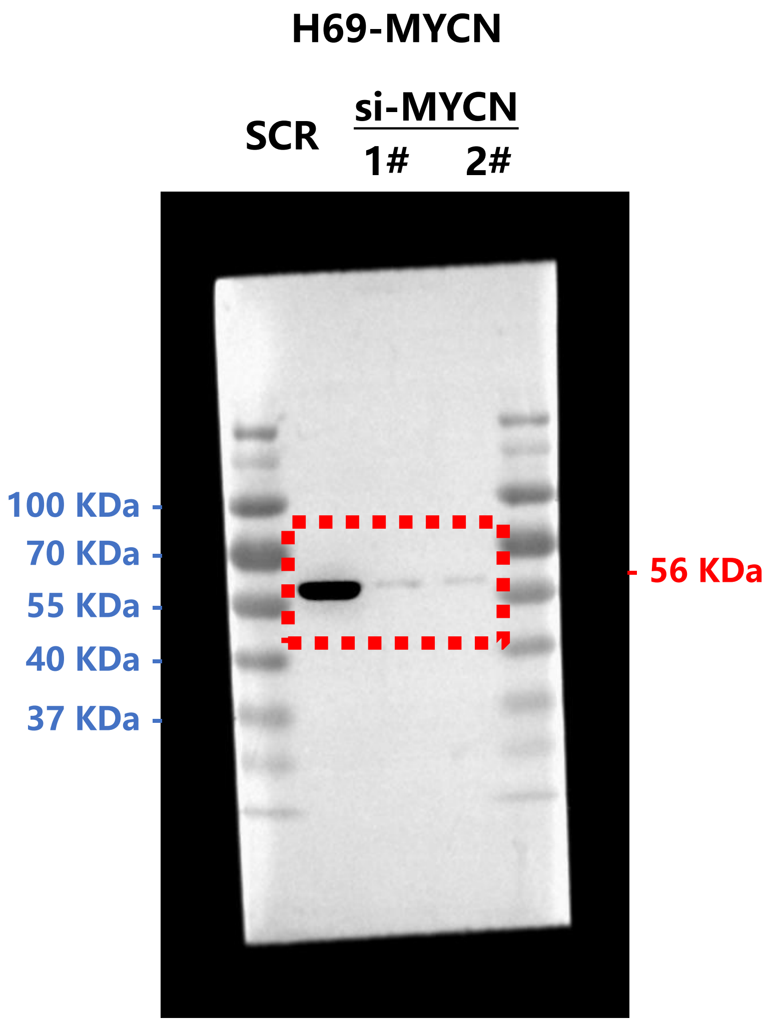

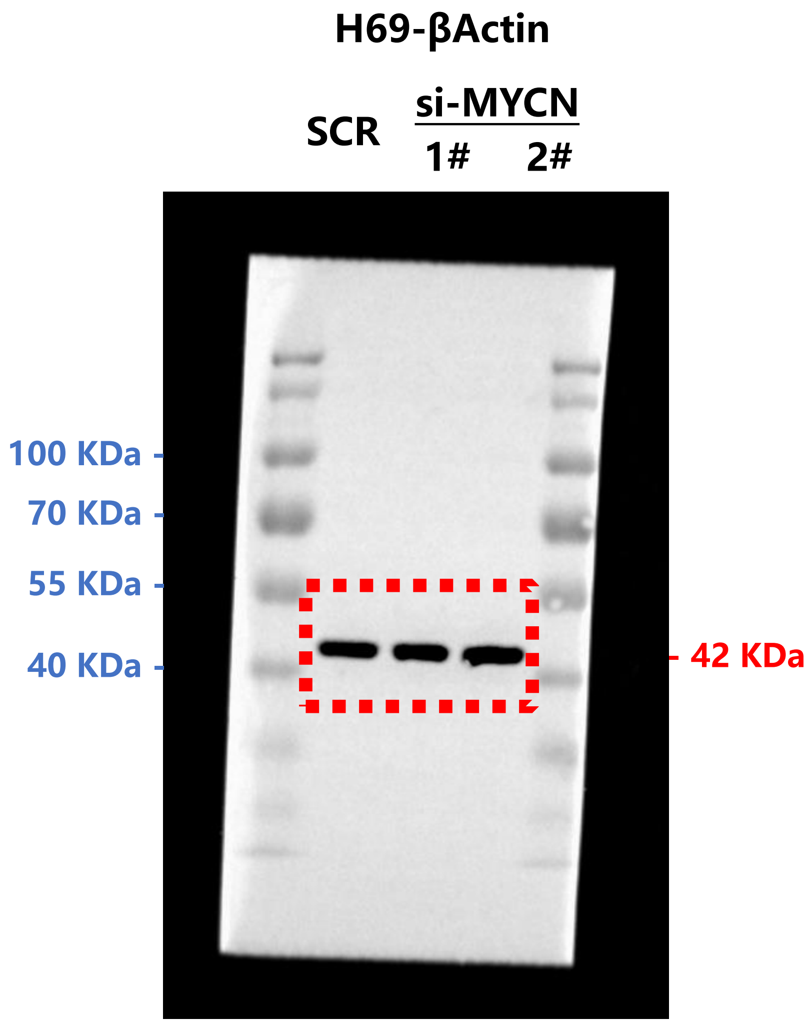

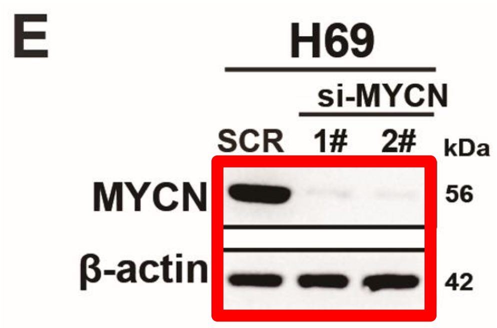

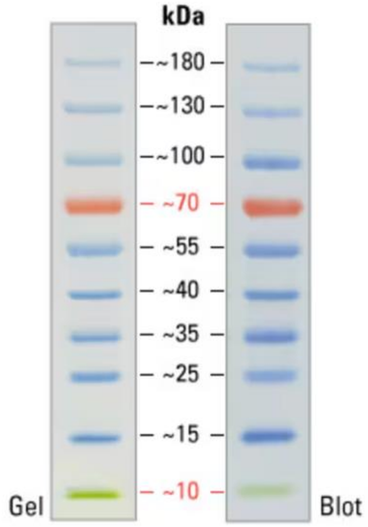

Thermo Scientific™ Marker-26617
